# Highly Enantioselective Organocatalysis with Bidentate Halogen Bond Donors

**DOI:** 10.1002/anie.202506476

**Published:** 2025-06-01

**Authors:** Julian Wolf, Meghana Poliyodath Mohanan, Raphaël Robidas, Revannath L. Sutar, Elric Engelage, Claude Y. Legault, Stefan M. Huber

**Affiliations:** ^1^ Fakultät für Chemie und Biochemie Ruhr‐Universität Bochum Universitätsstraße 150 44801 Bochum Germany; ^2^ Department of Chemistry Centre in Green Chemistry and Catalysis Université de Sherbrooke Sherbrooke Québec J1K 2R1 Canada

**Keywords:** Enantioselectivity, Halogen bonding, Noncovalent interactions, Organocatalysis

## Abstract

As the employment of “non‐classical” non‐covalent interactions like halogen bonding (XB) in asymmetric catalysis is still at a very early stage, there are significant challenges to overcome. In some reported cases, the relevance of halogen bonding to the catalytic action is unclear, while in others, catalyst activity is limited. Herein, we present the second generation of a bidentate iodine(I)‐based halogen bond donor as a modifiable and highly active chiral halogen bonding catalyst. With these modified derivatives, high stereocontrol of up to 98% ee could be achieved in a model Mukaiyama aldol reaction for a range of different substrates. Importantly, the crucial role of halogen bonding in this catalytic process was demonstrated by the low performance of the non‐iodinated variants and by DFT calculations. The latter also indicate that the stereoinduction is based on the imposed orientation of the substrates towards each other.

## Graphical abstract

In a model Mukaiyama aldol reaction with unbiased substrates, high enantioselectivity was achieved with a modifiable bidentate iodine(I)‐based halogen bonding organocatalyst. The crucial role of halogen bonding was confirmed by the low performance of the corresponding hydrogen bond donor and via DFT calculations.

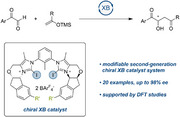

Over the course of the last few decades, the non‐covalent interaction of halogen bonding (XB)^[^ ^1^, ^2^, ^3^, ^4^, ^5^, ^6^, ^7^ ^]^ has risen from being somewhat of an obscurity to an almost routine concept in many applications, e.g. in crystal engineering,^[^ ^8^, ^9^, ^10^, ^11^, ^12^, ^13^, ^14^ ^]^ biomolecular and medicinal chemistry,^[^ ^15^, ^16^, ^17^, ^18^, ^19^ ^]^ as well as in the supramolecular recognition of Lewis bases (LB) in solution.^[^ ^20^, ^21^, ^22^, ^23^, ^24^ ^]^ Further research has also demonstrated the potential of halogen bond donors to facilitate organic reactions, providing intriguing alternatives to established organocatalysts.^[^ ^25^, ^26^, ^27^, ^28^ ^]^

Although a large variety of reactions^[^ ^29^, ^30^, ^31^, ^32^, ^33^, ^34^, ^35^, ^36^, ^37^, ^38^, ^39^, ^40^, ^41^, ^42^, ^43^, ^44^, ^45^, ^46^, ^47^ ^]^ have by now been catalyzed or activated by XB donors, achieving asymmetric induction with this interaction remains difficult.^[^ ^48^ ^]^ The intrinsic characteristics of XB, namely the high directionality of the R‐X–LB interaction as well as a large distance of the substrate to the (chiral) catalyst backbone, present significant challenges for the development of effective enantiocontrol. Consequently, successes in this area have only started to emerge in the last 5 years.

In 2020, our group introduced a chiral bis(imidazolium) based catalyst (**1^BArF^**), which achieved only moderate enantioselectivity in a Mukaiyama aldol reaction (Figure 1).^[^ ^49^ ^]^ Later in the same year, Garcia‐Mancheño and coworkers disclosed the application of a neutral, tetradentate iodotriazole‐based system for anion binding catalysis, with similarly limited selectivity.^[^ ^50^ ^]^ However, in 2023, they could achieve enantiomeric excesses of up to 90% *ee*, albeit only with particular substrates that featured additional electrophilic sites.^[^ ^51^ ^]^

**Figure 1:**
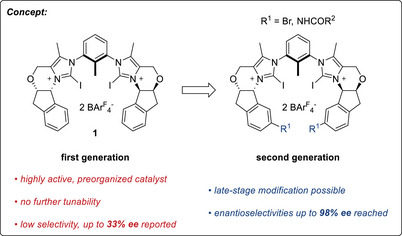
Previously reported (left) and modifiable second‐generation (right) chiral XB catalysts for effective enantioinduction.

In addition, Yoshida and coworkers have reported several examples in which bifunctional halonium(III) salts act as potent and enantioselective catalysts in the reaction of isatin derivatives with various nucleophiles. XB, however, seems to play a more complementary role in these cases.^[^ ^52^, ^53^ ^]^ High enantiomeric excesses in the same reaction have very recently been presented by Nachtsheim^[^ ^54^ ^]^ using monodentate iodine(III) derivatives.

Thus, there is currently still no precedence on the highly enantioselective activation of unbiased substrates by iodine(I)‐based catalysts, or bidentate ones in general, in which XB acts as the key driving force (“engine”). Herein, we present a first such example, in which a more potent modifiable variant of our previous catalyst motif **1^BArF^** induces enantioselectivities of more than 90%.

To this end, we reasoned that in the synthesis of a modifiable catalyst system, the derivatization of a common precursor should occur as late as possible. Due to the tolerance to different reaction conditions and a plethora of possibilities for later derivatization, the easily accessible^[^ ^55^ ^]^ (1*R*,2*S*)‐1‐Amino‐6‐bromo‐indan‐2‐ol (**2**) was thus chosen as the starting point (Scheme 1).

**Scheme 1:**
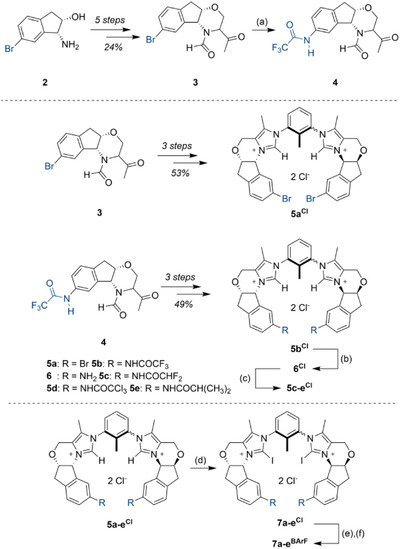
Synthesis of chiral halogen bond donors. Reaction conditions: a)  F_3_CCONH_2_, K_2_CO_3_, CuI (cat.), DMEDA (cat.), 1,4‐dioxane, 80 °C, 45 h, 75%. b) K_2_CO_3_, MeOH/H_2_O (1:1), r.t., 14 h, 85%. c) Acyl_2_O, EtOAc, r.t., 18 h, 65‐93%. d) NIS, MeCN 50 °C to 40 °C, 2 h. e) NaOTf, MeCN/MeOH (4:1), r.t., 16 h, 70%‐81% over two steps. (f) NaBAr^F^_4_, acetone, 42 °C, 2.5 h, 86%–98%.

From here, the transformation in 5 steps to the N‐formyl methyl ketone **3** was achieved in 24% yield, based on established procedures (see Supporting Information).^[^ ^56^ ^]^ This ketone was then used to form the corresponding bis(imidazolium) species **5a^Cl^** (Scheme 1) in 53% yield in a one‐pot process over three steps, analogously to the methods developed for the synthesis of **1^BArF^**.

As derivatization of this imidazolium intermediate proved difficult in orientating studies, further modification was already undertaken at the stage of the more robust ketone **3**. Here, an Ullmann‐type coupling^[^ ^57^ ^]^ introduced a trifluoroacetamide moiety as an anchor point, which allows for later modifications under mild conditions. With the resulting intermediate **4** in hand, the transformation into bis(imidazolium) derivative **5b^Cl^** was possible with 49% yield over 3 steps. After cleavage of the trifluoroacetamide, the widely functionalizable aniline **6^Cl^** was obtained in 85% yield, which should allow late‐stage modification. As an initial set of derivatives, amides like **5c‐e^Cl^** were targeted: they provide a stable linkage of the new substituents and offer a direct comparison to the original structure **5b^Cl^**. All these compounds were then smoothly transformed into halogen bond donors using N‐iodosuccinimide (NIS). Stepwise anion exchange and (partial) separation of atropisomers resulted in strong, functionalized halogen bond donors **7b‐e^Cl^**. Separation of atropisomers initially proved difficult, and a mixture was typically obtained. All screening experiments reported in the following were consequently conducted with these mixtures, in which very likely only the *syn*‐atropisomer will be the active species. A comparison of the enantiomeric excesses of the different catalysts should still be valid, as was later confirmed for **7b^BArF^** once the pure “*syn*”‐isomer could be obtained (see below).

The obtained yields, however, can only be considered an estimate of catalyst performance, as the active *syn*‐atropisomer is “diluted” to different degrees with the corresponding *anti*‐isomer.

Similar procedures for iodination and anion exchange were also applied for bromide species **5a^Cl^**, resulting in XB catalyst structure **7a^BArF^**. In order to determine the relevance of XB in relation to possible additional hydrogen bonding from the catalyst backbone, the hydrogen‐bearing analogues **5a^BArF^** and **5b^BArF^** were also prepared (see Supporting Information).

With this starting batch of modified, bidentate halogen bond donors in hand, we tested their performance in the asymmetric Mukaiyama aldol reaction of aryl glyoxals, for which we had previously attained only moderate enantioselectivity. As a model substrate, we chose the little‐explored^[^ ^58^ ^]^ trifluoromethyl‐bearing variant **8** (Table 1, top), which allows for facile reaction monitoring via ^19^F NMR. During orientating studies, the use of water‐free aryl glyoxals proved crucial for high selectivity and conversion, however, the monomeric aryl glyoxals are prone to oligomerization, resulting in lowered yields. Nevertheless, after some optimization for this substrate, the product could be obtained in well‐reproducible enantioselectivities and acceptable yields.

**Table 1:** Examination of XB catalysts, related reference compounds, and reaction conditions in a Mukaiyama aldol reaction.^a)^

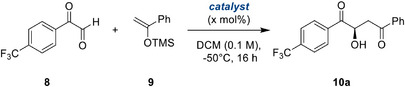				
Entry	Catalyst	Catalyst loading (mol%)	Yield (%)^b)^	ee (%)^c)^
1	**1^BArF^**	5	51	67
2	**7a^BArF^**	5	51	77
3	**7b^BArF^**	5	45	94
4	**7c^BArF^**	5	46	91
5	**7d^BArF^**	5	29	81
6	**7e^BArF^**	5	9	11
7	**5a^BArF^**	5	6	13
8	**5b^BArF^**	5	12	<2
9	**7b^BArF^**	2.5	25	94
10	**7b^BArF^**	10	50	94
11^d)^	“*syn*”‐**7b^BArF^**	5	63	94
12^d)^	“*syn*”‐**7b^BArF^**	1.7	50	94
13^d)^	“*syn*”‐**7b^BArF^**	0.8	43	93

^a)^ All reactions were conducted on a 0.09 mmol scale, using dry CH_2_Cl_2_ under argon atmosphere, employing 2 eq. of silyl enol ether **9**.

^b)^ Isolated yields.

^c)^ Determined by chiral HPLC analysis.

^d)^ A single catalyst atropisomer was used.

With 5% catalyst loading, after 16 h at −50 °C, non‐substituted catalyst **1^BArF^** provided product **10a** in acceptable enantioselectivity of 67% *ee*, while the bromide‐substituted catalyst **7a^BArF^** already yielded a higher selectivity of 77% *ee* (Table 1, entries 1 and 2). Another marked jump in stereocontrol was observed with the trifluoroacetamide moiety of **7b^BArF^**, resulting in excellent 94% *ee* (Table 1, entry 3). To the best of our knowledge, this represents the first organocatalytic approach to achieve 90%+ *ee* in such aldol reactions.^[^ ^59^, ^60^ ^]^ The comparably low difference in enantiomeric excess between bromine‐ and amide‐substituted donors **7a^BArF^** and **7b^BArF^** indicates that the key enantioinduction is exerted by the catalyst backbone, while the possibly hydrogen‐bonding amide plays at most an assisting role. Fortuitously, from these experiments, the aldol product crystallized in enantiomerically pure form, and its configuration could be determined as (*R*)‐**10a** (see SI).^[^ ^61^ ^]^

The structurally similar, but marginally less sterically demanding, catalyst **7c^BArF^** showed similar activity, with a yield of 46% after 16 h, and a slightly reduced selectivity of 91% *ee* (Table 1, entry 4). Derivative **7d^BArF^**, which offers more steric bulk compared to **7b^BArF^**, also provided lower enantioselectivity (81% *ee*, Table 1, entry 5), indicating a good steric match for the parent derivative **7b^BArF^**. Interestingly, XB donor **7e^BArF^** with its more electron‐rich isobutyramide moieties performed poorly, with only 9% of aldol product obtained after 16 h (Table 1, entry 6). Comparison with the results obtained for the non‐amide substituted catalysts **1^BArF^** and **7a^BArF^** indicates that the electron‐rich isobutyramide moieties appear to be detrimental to catalytic activity, resulting in drastically lower yields and selectivity for **7e^BArF^**.

The role of XB in both the activity as well as the selectivity of the catalyst was then further probed by the application of the non‐iodinated reference compounds **5a^BArF^** and **5b^BArF^**. The bromine‐substituted hydrogen bond donor **5a^BArF^** yielded 6% of product with an enantiomeric excess of only 13%, and the trifluoroacetamide derivative **5b^BArF^** generated 12% of racemic product (Table 1, entries 7 and 8).

All these findings clearly indicate that XB is the key interaction in this catalysis, not only in the activation of the substrate but also as essential driving force for strong asymmetric induction. The low yields observed for **5a^BArF^** and **5b^BArF^** are possibly the result of hydrogen bonding activation.

Further variation of the reaction conditions for the best catalyst **7b^BArF^** did not yield noticeable improvements: while a catalyst loading of 10% resulted in only a marginally improved amount of product, a considerably reduced yield was obtained with 2.5 mol%. In both cases, the same excellent stereoselectivity was still observed (Table 1, entries 9 and 10). As stated above, all experiments described so far were conducted with mixtures of *syn*/*anti*‐atropisomers. For **7b^BArF^**, the pure “*syn*”‐atropisomer^[^ ^62^ ^]^ could eventually be obtained in small amounts, which allowed a comparison of its performance with the one of the mixture. Although the enantioselectivities remained unaltered (Table 1, entries 11–13), the yield noticeably improved for the same overall catalyst loading (entry 11 vs. entry 3). The yield could be approximated, however, by using the equivalent amount of pure *syn*‐isomer (1.7 mol%) that would be present in the 5 mol% loading of the mixture (entry 12).^[^ ^63^ ^]^ Even when the loading of the pure catalyst is lowered below 1%, decent yields are still achieved.

Following this, our interest shifted to a screening of the silyl enol ethers used in the reaction. As we are currently mainly interested in the elucidation of the mode of enantioinduction by the halogen bond donors, the behavior of different substrates could allow to draw conclusions on the structure of the key transition state.

Surprisingly, even slight modifications, such as the addition of methyl groups towards products **10b**‐**10d**, led to noticeable drops in enantioselectivity (Scheme 2). Although the 2‐Me (**10b)** and 3‐Me (**10c**) substituted products were obtained in similar yield and selectivity, the enantiomeric excess of the 4‐Me substituted aldol product **10d** was even lower, at only 70% *ee*. Nevertheless, for methoxy‐substituted product **10e** and fluoride derivative **10f,** still 87% and 82% *ee* could be achieved, while the bromide derivative **10** **g** was obtained in reduced selectivity (Scheme 2).

**Scheme 2:**
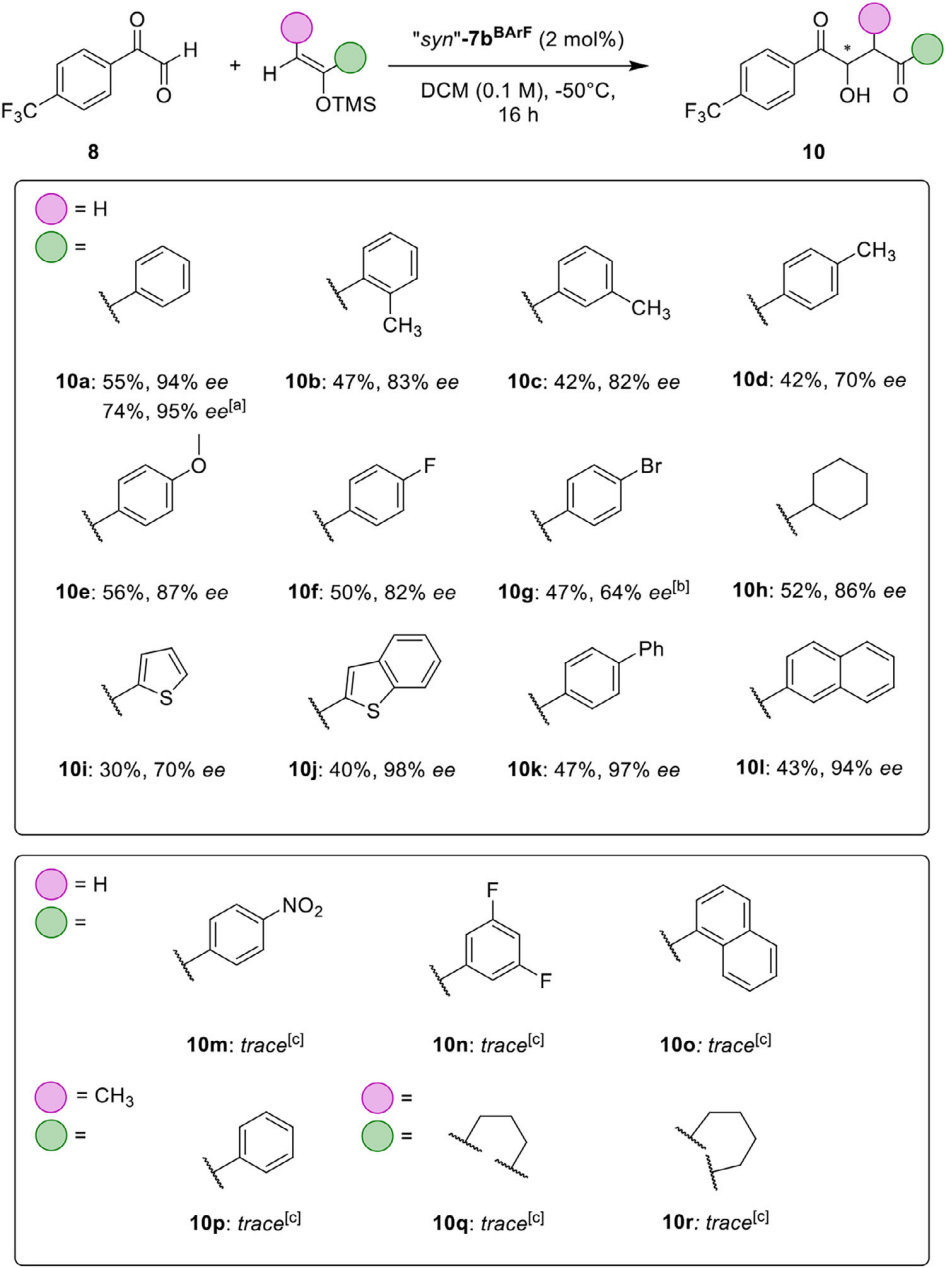
Substrate screening for the reaction of various silyl enol ethers with aryl glyoxal **8**. Reactions were conducted on a 0.09 mmol scale, using dry CH_2_Cl_2_ under argon atmosphere, employing 2 eq. of the respective silyl enol ether and 2 mol% of catalyst “*syn*”‐**7b^BArF^**, isolated yields given. Enantiomeric excess determined by chiral HPLC analysis. [a] Reaction performed at −70 °C for 64 h of reaction time. [b] 64 h of reaction time. [c] Analytical scale screening performed on 0.03 mmol scale, using 5 mol% of atropisomeric mixture of **7b^BArF^**, 40 h of reaction time, yields determined by NMR.

Interestingly, the cyclohexyl analogue **10** **h** could be accessed in moderate yield and with a significant enantioselectivity of 86% *ee*, while the thiophene derivative showed poorer selectivity (Scheme 2, **10i**). On the other hand, with extended π–systems in the cases of 2‐benzothiophenyl‐ (**10j**), 4‐biphenyl‐ (**10k**), and 2‐naphthyl‐ (**10l**) substituted aldol products, excellent enantioselectivities of up to 98% *ee* were observed. Screening of further nucleophiles revealed that highly electron‐deficient nucleophiles (**10m** and **10n**) as well as silyl enol ethers with increased steric demand near the alkene moiety were (**10o** to **10r**) were not tolerated under the optimized conditions.

Although these findings also show some limitations of the scope, they also confirm that outstanding selectivities can be achieved with other substrates, and that good base‐level enantioselectivity is observed for several less suitable substrates. The narrow requirements on the substrates to see truly exceptional enantiocontrol hint at a tight entanglement between the catalyst and substrate in the transition state.

As we turned our attention towards the evaluation of differently substituted aryl glyoxals, a similar sensitivity of the asymmetric induction to seemingly small changes in the substrate was observed. Although the bromide‐substituted glyoxal**10s** (Scheme 3) could be converted into the aldol product with 94% *ee* – displaying yet another case of very high enantioselectivity – attempts to uncover the peculiar characteristics of a further substituent which enables this level of stereocontrol proved difficult: an isopropyl substituent, which has been considered as an isostere of the trifluoromethyl group,^[^ ^64^, ^65^, ^66^, ^67^ ^]^ resulted in 51% yield but only 75% *ee* for the aldol product **10t**.

**Scheme 3:**
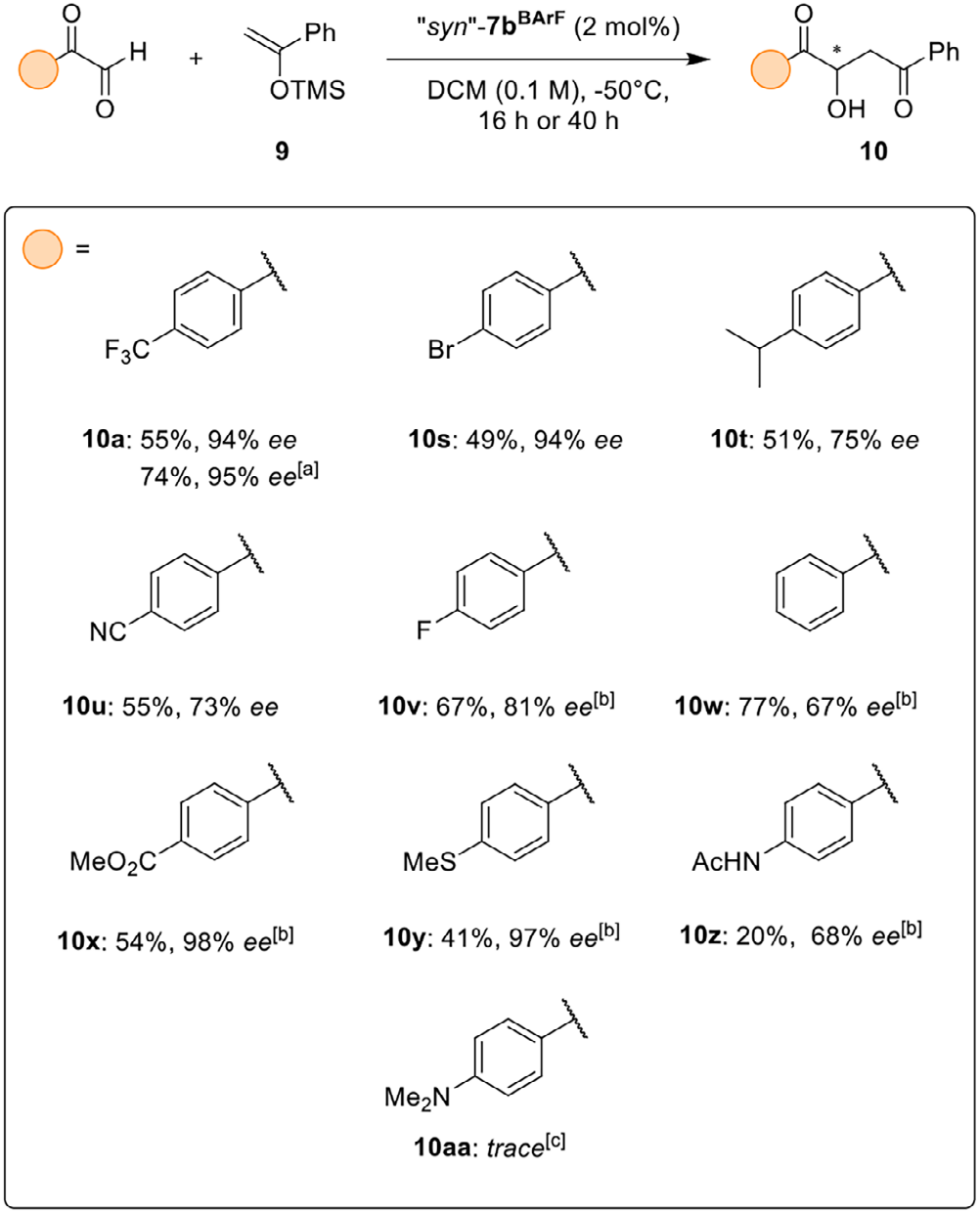
Substrate screening for the reaction of aryl glyoxals with silyl enol ether **9**. Reactions were conducted on a 0.09 mmol scale, using dry CH_2_Cl_2_ under argon atmosphere, employing 2 eq. of the respective silyl enol ether and 2 mol% of catalyst “*syn*”‐**7b^BArF^** (unless noted otherwise), isolated yields given. Enantiomeric excess determined by chiral HPLC analysis. [a] Reaction performed at −70 °C for 64 h of reaction time. [b] 40 h of reaction time. [c] Using 5 mol% of atropisomeric mixture of **7b^BArF^**, 40 h of reaction time.

Similarly, an attempt to roughly emulate the electron‐withdrawing properties^[^ ^68^ ^]^ of the trifluoromethyl group using a cyano substituent resulted in a selectivity of just 73% *ee* in product **10u**. On the other hand, puzzlingly, the fluoride‐substituted product **10v** was obtained in slightly higher selectivity while the non‐substituted product **10w** was isolated in only 67% *ee*.

Still, exceptional enantioselectivity could also be observed for ester **10x** and thioether **10y**, with enantioselectivities of 98% *ee* and 97% *ee*, respectively. Whereas the possibly competing nucleophilic ester group of **10x** had been tolerated, the introduction of an acetamide (in product **10z**) significantly impacted product formation and also resulted in lower enantioinduction. In conjunction with the incompatibility of the methylated aniline in the reaction towards **10aa**, this hints at limitations of the described process in the presence of competing nucleophiles.

Due to the complexity of the catalyst, it is not trivial to understand the key features leading to such high enantioselectivities. We thus turned to computational chemistry to gain insight into the stereoinduction process of the stereo‐determining C─C bond formation step of the aldol reaction. Substrate **8** and enol ether **9**, as well as biscationic catalyst **7b**, were used for the calculations. Extensive conformational sampling of transition structures leading to both the *(R)* and *(S)* enantiomers of **10a** was performed, using CREST.^[^ ^69^, ^70^ ^]^ Both prochiral faces of substrate **8** and both diastereotopic faces of enol ether **9** were investigated to ensure complete sampling. The various conformers were modelled at the M06‐2X/def2‐TZVP (def2‐TZVPD for I)//M06‐2X/def2‐SVP (def2‐SVPD for I) level, utilizing the SMD solvation model for dichloromethane, with revised radii for iodine.^[^ ^71^ ^]^ For each transition state leading to the *(R)* and *(S)* products, two conformers were identified as contributing to over 99% of the Boltzmann populations (refer to Supporting Information). Among these, the most stable conformers of the transition states (**TS‐1*_R_*** and **TS‐1*_S_***) each account for more than 80% of the Boltzmann populations and are depicted in Figure 2a.

**Figure 2:**
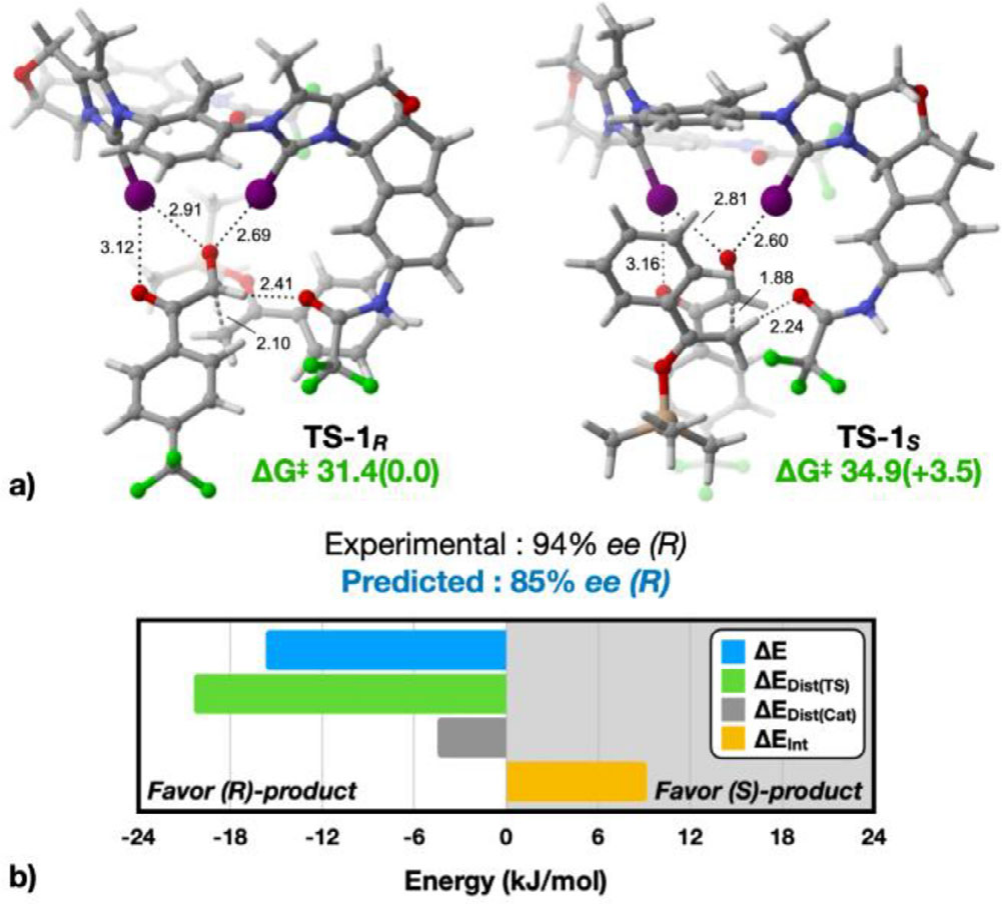
a) Lowest energy conformations of transition structures leading to *(R)* and *S)* products, respectively. Boltzmann‐averaged free energies reported (see Supporting Information); b) Relative distortion/interaction analysis of the transition structures. Energies reported in kJ mol^−1^.

The calculated free energy difference between the transition states is 3.5 kJ mol^−1^ in favor of *(R)*. At −50 °C, this represents a predicted enantioselectivity of 85% *ee*, which aligns closely with the experimentally observed selectivity of 94% *ee*. Furthermore, the barriers of the catalyzed process are 31.4 (toward *R*) and 34.9 (toward *S*) kJ mol^−1^, noticeably lower than that of the uncatalyzed process (51.2 kJ mol^−1^, see Supporting Information), in support of the accelerating effect of the catalyst. Notably, both transition structures feature the substrate being bound in the same manner, with the two iodine atoms chelating on the aldehyde carbonyl and one iodine forming an additional weaker halogen bond to the second carbonyl oxygen. In both transition states, the oxygen of one amide group acts as a hydrogen bond acceptor to either the aldehyde (**TS‐1_R_**) or the silyl enol ether (**TS‐1_S_**), but both interactions are, however, relatively weak. Despite multiple attempts, no energetically reasonable conformer was found in either case in which the amide would act as a hydrogen bond donor through the NH hydrogen.

A key difference between the two transition states is the difference in position on the reaction coordinates, as **TS‐1*_S_*** is found to be a later transition state, with a C─C distance of the forming bond of 1.88 Å versus 2.10 Å for **TS‐1*_R_***. This is also evident in the interaction of the aldehyde oxygen atom, where distances of 2.81 and 2.60 Å are observed, in contrast to the 2.91 and 2.69 Å associated with **TS‐1*_R_***. This indicates a stronger interaction, attributed to the increased electron density transferred to the oxygen atom of the aldehyde carbonyl by the incoming nucleophile. Given the intricate nature of the transition structures, identifying a specific characteristic to explain the selectivities is challenging.

As a result, we conducted a distortion/interaction (activation strain) model analysis of **TS‐1*_R_*** and **TS‐1*_S_*** to uncover a potential rationale, as illustrated in Figure 2b.^[^ ^72^ ^]^ Notably, the interaction energy favors **TS‐1*_S_***, which aligns with the late nature of the former, as well as the enhanced XB between the substrate and the catalyst in this scenario (Figure 2b, orange bar). In the distortion analysis, the transition structures were divided into the catalyst and aldol **8^…^9** transition structure fragments, yielding the **ΔE_Dist(Cat)_** and **ΔE_Dist(TS)_** energies, respectively. This reveals that the primary advantage of **TS‐1*_R_*** stems from the significantly larger **ΔE_Dist(TS)_** energy penalty associated with **TS‐1*_S_*** (Figure 2b, green bar). We therefore examined in greater detail the characteristics of the **8^…^9** aldol transition structure fragments identified in **TS‐1*_R_*** and **TS‐1*_S_***, with the results shown in Figure 3.

**Figure 3:**
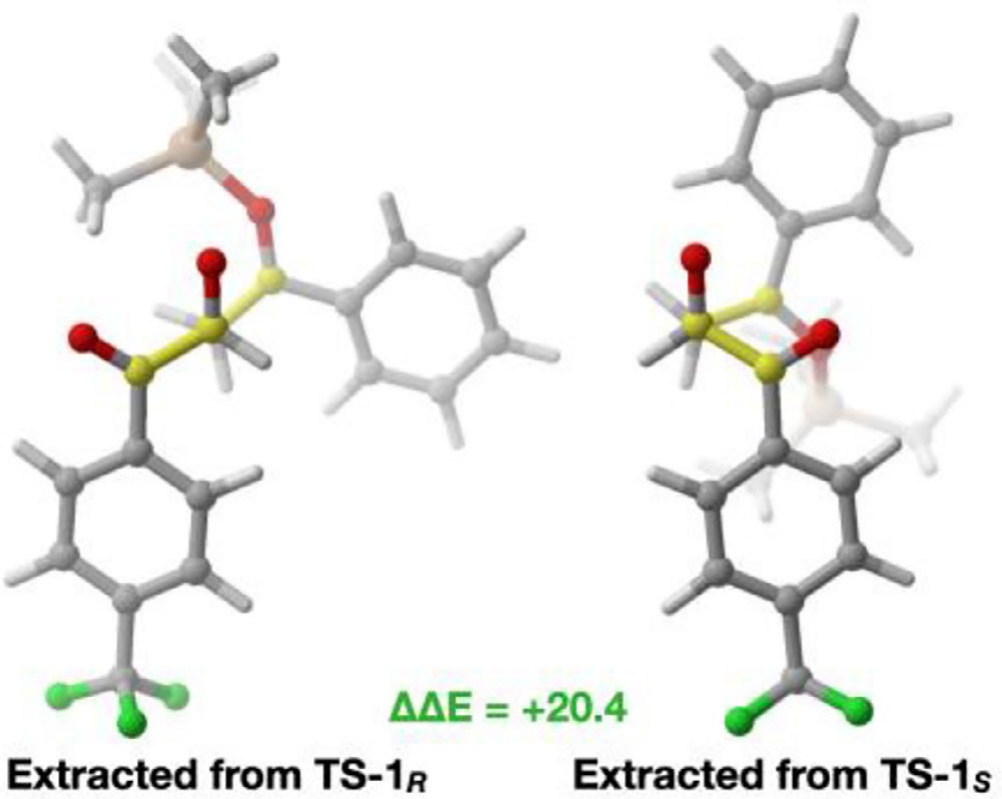
Adducts of **8** ^…^ **9** aldol transition structures fragments extracted from the lowest energy conformations of transition structures reported in Figure 2a. Energy reported in kJ mol^−1^.

For the aldol in **TS‐1*_R_***, an antiperiplanar orientation is observed between **8** and **9**, allowing the silyl group to be ideally positioned for transfer once the C─C bond is formed. This configuration minimizes steric clashes. In contrast, **TS‐1*_S_*** displays a synclinal orientation of **8** and **9**, resulting in significant steric clashes. The transition state extracted from **TS‐1*_R_*** was reoptimized to provide a true optimized, uncatalyzed transition state, resulting in a barrier of 53.2 kJ mol^−1^, very close to the lowest energy transition state found for the uncatalyzed process (51.2 kJ mol^−1^), which also presented an antiperiplanar orientation (see Supporting information).

Based on these analyses, it appears that: 1) The double XB to the aldehyde carbonyl serves as the primary activation mechanism for catalyst **7b**. Conformational sampling indicates that this binding notably lowers energy, resulting in the formation of the lowest energy transition structures. 2) Although the substrate's interaction with the catalyst does not effectively obstruct one side of the aldehyde, the overall chiral environment imposes remote limitations on the orientation of the incoming enol ether nucleophile. 3) Therefore, selectivity primarily stems from the **8** ^…^ **9** orientation in the aldol C─C bond forming step, where the major enantiomer forms through the highly favored antiperiplanar orientation. This orientation minimizes steric clashes.

It is still too early to grasp the subtle effects of the nature of substrates **8** or enol ethers **9** on selectivities. Further investigation is required to address the full stereochemical induction process and the substrate‐catalyst relationship. However, with regard to the catalyst, it seems that the improved selectivities provided by **7b** over **1** stem from the further reach of the trifluoroacetamide groups in space, providing larger steric bulk to disfavor an optimal orientation of enol ether **9** in the aldol transition state for the *(S)* enantiomer, while providing an ideal positioning of substrate **8** in the transition state leading to the *(R)* enantiomer.

In conclusion, a modifiable bidentate chiral halogen bond donor led to excellent enantioselectivities in a Mukaiyama aldol test reaction. Next to the optimal catalyst with trifluoroacetamide substituents, variants with other groups were tested as well: difluoroacetamide also provided 90%+ enantioselectivity, decent asymmetric induction was still achieved with trichloroacetamide and with bromine substituents, but very low enantiomeric excess was obtained with isobutyramide.

A substrate screening of reaction partners revealed a significant base level of stereoselectivity for different silyl enol ethers and aryl glyoxals, while even seemingly small variations led to noticeably reduced asymmetric induction. Control experiments using non‐iodinated congeners re‐confirmed the crucial role of XB in this catalyst motif, both for activity and enantioselectivity. DFT calculations showed that the preferential formation of the *(R)* product is due to an unfavorable orientation of the aldol partners in the pocket of the chiral catalyst for the *(S)* product.

Overall, this constitutes the first case in which highly asymmetric induction was achieved with an iodine(I)‐based (and bidentate) catalyst predominantly via XB and with unbiased (non‐halogen bonding) substrates. As such, it marks an important step in the further development of increasingly sophisticated organocatalyses with this interaction (even though adaptations of the catalyst structure to other types of substrates will likely be necessary).^[^ ^73^ ^]^

## Supporting Information

The authors have cited additional references within the Supporting Information.^[^ ^74^, ^75^, ^76^, ^77^, ^78^, ^79^, ^80^, ^81^, ^82^, ^83^, ^84^, ^85^, ^86^, ^87^, ^88^, ^89^, ^90^, ^91^, ^92^, ^93^, ^94^, ^95^, ^96^, ^97^, ^98^ ^]^

## Conflict of Interests

The authors declare no conflict of interest.

## Supporting information



Supporting Information

Supporting Information

Supporting Information

## Data Availability

The data that support the findings of this study are available from the corresponding author upon reasonable request.
