# Supplementary material for: Highly Enantioselective Organocatalysis with Bidentate Halogen Bond Donors
Source: Angew Chem Int Ed Engl. 2025 Jun 1;64(30):e202506476. doi: 10.1002/anie.202506476 (PMC12281084; doi:10.1002/anie.202506476)
Supplement: Supplementary file 1 — Supporting Information [file ANIE-64-e202506476-s001.pdf]

Supporting Information

Highly Enantioselective Organocatalysis with Bidentate Halogen Bond Donors

Julian Wolf[a], Meghana Poliyodath Mohanan[a], Raphaël Robidas[b], Revannath L. Sutar[a], Elric Engelage[a], Claude Y. Legault*[b], Stefan M. Huber*[a]

[a] Fakultät für Chemie und Biochemie, Ruhr-Universität Bochum, Universitätsstraße 150, 44801 Bochum, Germany

[b] Department of Chemistry, Université de Sherbrooke, Centre in Green Chemistry and Catalysis, Sherbrooke (Québec), Canada, J1K 2R1

Contents

1. Experimental Section ... 1
    1.1. General Information ... 1
    1.2. Synthesis of XB catalysts and reference structures ... 2
    1.3. Catalysis experiments ... 34
2. Crystallographic data ... 49
3. Computational Details ... 60
4. HPLC chromatograms ... 63
5. NMR spectra ... 80
6. References ... 157

1. Experimental Section

1.1. General Information

All used fine chemicals and solvents were purchased from commercial sources and were used without further purification, if not stated otherwise. Anion exchange resin (Amberlite IRA 958 Cl) was thoroughly washed with methanol before use.

Diethyl ether and *n*-pentane were purchased in technical grade and used after single distillation. Dry dichloromethane, diethyl ether and tetrahydrofuran were received from an *MBRAUN MB SPS-800*. Other dry solvents were obtained by storage over flame-dried 3 Å or 4 Å molecular sieves under argon. The water content of solvents was determined with a *Karl Fischer Titroline ®7500KF trace* from *SI Analytics* with *Honeywell Hydranal Coulomat-AD*.

All moisture or air-sensitive reactions were performed using standard Schlenk technique under argon as inert gas using dry solvents and flame-dried glassware. Thin layer chromatography was performed using *Merck TLC aluminum sheets* (silica gel 60, F254) and spots were visualized using a UV lamp at 254 nm or using $KMnO_4$ staining solution. For column chromatography, silica gel of grain size 0.04-0.063 mm (*Macherey-Nagel Si60*) was used. It was performed under atmospheric or positive pressure (by use of a hand pump or membrane pump). Used eluents and found retardation factors (R_f) are mentioned in the experiments.

NMR spectra were measured on *Bruker Avance III 300, Avance III 400 or Neo 400* spectrometers at ca. 300 K. Chemical shifts (δ) are given in parts per million (ppm) and are internally referenced to tetramethylsilane by residual solvent signals. Spectra were analyzed using *MestReNova*. Multiplicities are abbreviated as s (singlet), d (doublet), t (triplet), q (quartet), p (pentet), sept (septet), m (multiplet), and combinations of these, e.g. td (triplet of doublets). The relative integral and the coupling constant (*J* in Hz) are indicated if possible. ^{13}C NMR spectra are only ^{1}H decoupled, if not noted otherwise (in the case of additional ^{19}F decoupling). IR spectra were obtained using a *Shimadzu IR Affinity – 1S* spectrometer with a *Specac-Quest* ATR unit. Peaks are reported in $\tilde{\nu}$ = cm^{-1} and are indicated with w (weak), m (medium), s (strong) or vs (very strong). High resolution mass spectrometry (MALDI-TOF-MS or LIFDI-MS) was performed using *Bruker Ultraflex III* (MALDI-TOF-MS) or *JEOL AccuTOF GCv* (*JMS-T100GCV*) instruments (LIFDI-MS) respectively. High-performance liquid chromatography (HPLC) analysis was performed using a *Shimadzu Nexera XR* setup, employing analytical chiral HPLC columns by *Dr. Maisch* (Reprosil Chiral-NR, 8 µm, 250 mm x 4.6 mm) and *YMC (*CHIRAL ART Amylose-SA, 5 µm, 250 mm x 4.6 mm). Polarimetry was performed using an *Anton Paar MCP 500*. Optical rotations are reported as: $[\alpha]_D^T$ (c: g/100 mL, solvent), measured at 20°C using 589 nm wavelength.

Racemic aldol products were produced using achiral XB catalyst **SI-10** (see page S48, can also be found as compound **9** in manuscript reference [33]), using analogous conditions as described for the asymmetric examples, with 2 h reaction time at 0 °C (except for products **10x** and **10z**, which were synthesized at -50 °C due to poor selectivity at higher temperatures, using 5 mol% catalyst).The reaction mixtures were purified by preparative TLC and the products directly subjected to chiral HPLC analysis.

Aryl glyoxal hydrates were synthesized by Riley oxidation of the corresponding, commercially available acetophenones, according to literature procedures.[74-77]

Silyl enol ethers were synthesized from the corresponding, commercially available acetophenones using triethylamine, trimethylsilyl chloride and sodium iodide, according to literature procedures.[78-80]

1.2. Synthesis of XB catalysts and reference structures

Synthesis of *N*-((1*R*,2*S*)-6-bromo-2-((3-methyloxiran-2-yl)methoxy)-2,3-dihydro-1H-inden-1-yl)formamide (SI-3)

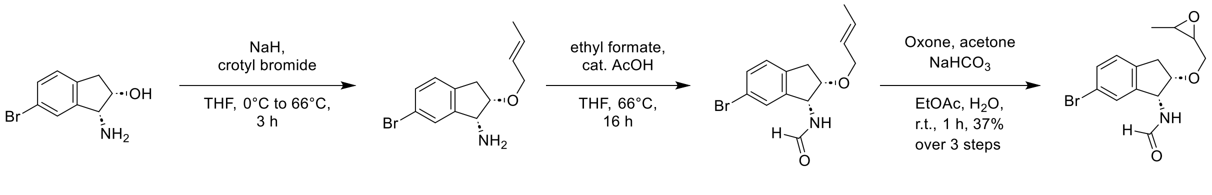

2 **SI-1** **SI-2** **SI-3**

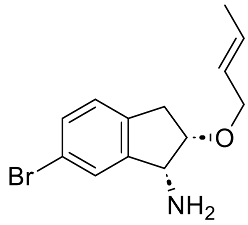

SI-1

Under an Ar atmosphere, 1.45 g of NaH (60% suspension in mineral oil, 60.5 mmol, 1.2 eq.) was added to 500 ml of dry THF. The resulting suspension was cooled to 0°C. Under stirring, 6-Bromo(1*R*,2*S*)-aminoindanol (**2**) (11.5 g, 50.4 mmol, 1.0 eq.) was added in portions over 30 minutes. The resulting mixture was stirred for 20 minutes at 0°C and then heated to reflux temperature. Next, a solution of crotyl bromide (50 vol% in THF, 8.17 g, 60.5 mmol, 1.2 eq) was added dropwise over 110 minutes to the refluxing mixture. The resulting suspension was stirred for a further 60 minutes at reflux temperature, after which it was cooled down to 0°C. The reaction mixture was quenched by slow addition of saturated aqueous NH_4Cl solution. The formed suspension was poured into a separatory funnel and mixed with 500 ml of brine. The aqueous layer was twice extracted with EtOAc (400 ml each), and the combined organic phases were dried over Na_2SO_4. Concentration under reduced pressure yielded a dark oil, which was dissolved in 500 ml of Et_2O. The solution of the crude product was cooled to 0°C and 4 M HCl in 1,4-dioxane (12.6 ml, 50.4 mmol, 1.0 eq.) was added slowly. The formed precipitate was filtered off and washed with diethyl ether.

The obtained hydrochloride salt of the crude product was then dried under vacuum. Following this, it was freebased by addition of 1 M NaOH solution and extraction of the product with diethyl ether. The aqueous layer was extracted twice with Et_2O and the combined organic layers were dried over Na_2SO_4. Removal of the solvent under reduced pressure finally yielded the crude product **SI-1** as a dark brown oil (9.83 g, 34.8 mmol, 69% crude yield). The product was not further purified and used as received in the next step.

^{1}H NMR (300 MHz, Chloroform-*d*):

See attached spectra.

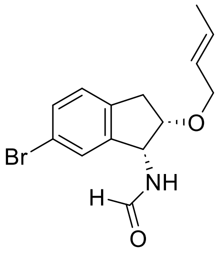

SI-2

Crotylated aminoalcohol **SI-1** (9.82 g, 34.8 mmol, 1.0 eq.), ethyl formate (20.4 g, 22.1 ml, 275 mmol, 7.9 eq.) and catalytic acetic acid (104 mg, 0.1 ml, 1.74 mmol, 0.05 eq.) were dissolved in 70 ml dry THF under Ar atmosphere and stirred at reflux temperature for 16 h. The solvent was removed *in vacuo* and the residue sonicated in the presence of *n*-pentane and the supernatant solvent decanted subsequently. The solid residue was dried *in vacuo* and then dissolved in DCM, insoluble residue was filtered off and the solvent was removed *in vacuo*. The crude product was dissolved in a minimal amount of DCM, then excess *n*-pentane was added to precipitate the product and the mixture was stored at -30°C overnight,

then the product was filtered off and the procedure of precipitation was repeated twice with the respective filtrates to precipitate all formed product. The crude product **SI-2** was thus obtained as a greyish/brownish solid (7.93 g, 25.6 mmol, 74% crude yield).

^{1}H NMR (300 MHz, Chloroform-*d*):

See attached spectra.

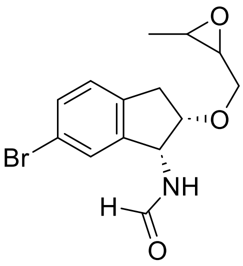

SI-3

The crude *O*-crotylated and *N*-formylated aminoindanol **SI-2** (7.30 g, 23.5 mmol, 1.0 eq.) was added to 120 ml of EtOAc and 180 ml of H_2O. $NaHCO_3$ (9.87 g, 117 mmol, 5.0 eq.) and acetone (13.7 g, 17.4 ml, 235 mmol, 10 eq.) were also added to the mixture. The biphasic mixture was then vigorously stirred at room temperature. To the stirring mixture, a solution of potassium peroxymonosulfate (as triple salt, (*Oxone*), 28.9 g, 47.1 mmol, 2.0 eq.) in 200 ml of H_2O was added dropwise over 1 hour. After complete addition, the reaction was stirred for one further hour at room temperature. The organic phase was then separated, and the aqueous phase extracted twice with EtOAc. The combined organic phases were washed with brine and subsequently dried over Na_2SO_4. The solvent was removed *in vacuo* and the resulting off-white solid was purified by column chromatography (SiO_2, ethyl acetate/*n*-pentane 9:1) to yield the product **SI-3** as a colorless solid (4.05 g, 12.4 mmol, 53%).

Additionally, starting material could be recovered (2.16 g, 6.97 mmol, 30%) after chromatography, leading to an overall process efficiency of 73% with three cycles.

R_f (EtOAc/*n*-pentane 9:1): 0.23

^{1}H NMR[1] (300 MHz, Chloroform-*d*):
δ [ppm] = 8.39 (s, 1H), 8.29* (d, *J* = 12.0 Hz, 1H), 7.56 – 7.29 (m, 2H), 7.16 – 7.00 (m, 1H), 6.71** (d, *J* = 8.9 Hz, 1H), 6.56** (d, *J* = 9.0 Hz, 1H), 6.27* (q, *J* = 12.3 Hz, 1 H), 5.56 (dt, *J* = 9.3, 4.8 Hz, 1H), 4.92* (dt, *J* = 10.7, 5.8 Hz, 1H), 4.40 – 4.20 (m, 1H), 3.95 – 3.71 (m, 1H), 3.58** (dd, *J* = 11.7, 5.0 Hz, 1H), 3.45* (ddd, *J* = 19.8, 11.8, 5.8 Hz, 1H), 3.31** (dd, J = 11.3, 6.6 Hz, 1H), 3.08 – 2.78 (m, 4H), 1.38 – 1.23 (m, 3H).

^{13}C NMR (75 MHz, Chloroform-*d*):

δ [ppm] = 161.4, 161.4, 143.5, 143.5, 138.6, 138.5, 131.3, 131.3, 127.7, 127.7, 126.7, 126.6, 120.9, 120.9, 81.7, 81.1, 70.5, 69.8, 58.0, 57.8, 54.7, 54.6, 52.2, 52.1, 36.8, 36.2, 17.3, 17.3.

ATR-IR:
$\tilde{\nu}$ [cm^{-1}] = 3262 (w), 3051 (w), 2990 (w), 2967 (w), 2911 (w), 2872 (w), 1719 (w), 1597 (vs), 1545 (m), 1472 (w), 1437 (w), 1422 (w), 1391 (m), 1356 (w), 1314 (w), 1236 (m), 1179 (w), 1153 (w), 1119 (m), 1072 (w), 1057 (w), 1036 (w), 995 (w), 937 (w), 862 (w), 851 (m), 824 (m), 800 (m), 712 (m), 652 (m), 600 (w), 556 (m), 529 (w), 466 (m).

HRMS (MALDI-TOF):

m/*z* (+) = calc. 348.0206 [M+Na]$^+$; found 348.0203 [M+Na]$^+$.

---

1 The NMR spectra show a mixture of two diastereomers in a ratio of 1:1 (as reported in manuscript reference [56] for the analogous, non-brominated species) and another, minor isomer (1:10 to the major product, similar results can be observed in the supporting information of reference [56]). Signals indicated with a star can be assigned to the minor isomer, while other signals show overlap or belong solely to the major isomer. Signals which can be solely assigned to either one of the diastereomers of the major rotamer are marked with two stars. For ^{13}C NMR spectroscopy, due to signal overlap and low intensity, only the signals of the major isomer are listed.

Synthesis of (4a*R*,9a*S*)-3-acetyl-6-bromo-2,3,9,9a-tetrahydroindeno[2,1-b][1,4]oxazine-4(4a*H*)-carbaldehyde (3)

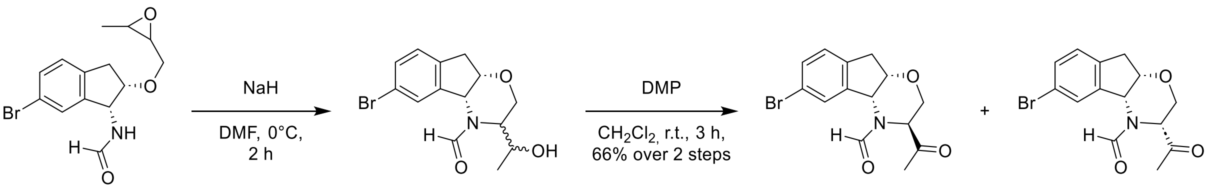

SI-3 **SI-4** **3a** **3b**

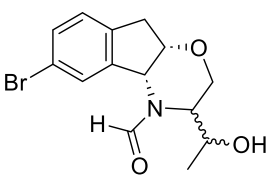

SI-4

To a flame-dried Schlenk flask, NaH (10.9 g, 60% suspension in mineral oil, 272 mmol, 10 eq.) was added. Subsequently, 340 ml of dry DMF (0.08 M) were added. The suspension was stirred vigorously and cooled to 0°C. To this suspension, a 1 M solution of the *N*-formamide epoxide starting material **SI-3** in DMF (27.2 ml, 8.87 g, 27.2 mmol, 1.0 eq.) was added dropwise over 100 minutes. The solution was stirred for a further 40 minutes at 0°C. The reaction mixture was then carefully quenched dropwise with ice cold saturated NH_4Cl solution at 0°C. The quenched solution was then poured onto another 200 ml of NH_4Cl solution. The resulting solution was extracted five times with EtOAc and the combined organic phases were washed once with half-concentrated brine and once with saturated brine solution. Afterwards, the organic phases were dried over Na_2SO_4 and the solvent was removed *in vacuo*. The remaining mineral oil was removed by washing the residue with *n*-pentane and the product was then dried under high vacuum to yield the crude product as a reddish foam.

This residue was subjected to column chromatography (SiO_2, DCM/acetone 4:1 → 2:1) and the product **SI-4** was received as a complex mixture of diastereomers and rotamers as a reddish foam (6.31 g, 19.3 mmol, 71% crude yield). The product mixture was then directly used in the subsequent oxidation reaction.

R_f (DCM/acetone 4:1): 0.30-0.24 (mixture of diastereomers)

^{1}H NMR (300 MHz, Chloroform-*d*):

See attached spectra, clear assignment difficult.

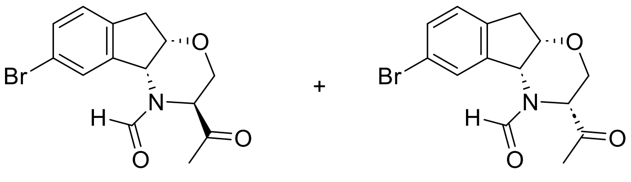

3a **3b**

In a flame-dried Schlenk flask, a suspension of Dess-Martin periodinane (17.9 g, 42.3 mmol, 1.2 eq.) in dry CH_2Cl_2 (0.195 M) was prepared under argon atmosphere at room temperature. Under stirring, a solution of the alcohol starting material **SI-4** (11.5 g, 35.3 mmol, 1.0 eq.) in dry CH_2Cl_2 (0.5 M) was added and the mixture was stirred for 3 hours at room temperature, after which full conversion to the products was observed by TLC. The mixture was then diluted with CH_2Cl_2, washed once with 1 M NaOH (400 ml), twice with H_2O (500 ml) and once with brine (400 ml). The organic phase obtained after the washing steps was then filtered through *Celite* and dried over Na_2SO_4. Finally, the solvent was removed *in vacuo* to yield the crude product. Column chromatography (SiO_2, *n*-pentane/acetone 3:2) was performed on the residue to yield the two diastereomers (**3a** (minor) and **3b** (major)) of the product as light beige solids (10.7 g, 32.9 mmol, 93% yield).

Minor diastereomer **3a**:

R_f (*n*-pentane/acetone 3:2): 0.42

^{1}H NMR[2] (400 MHz, Chloroform-*d*):
δ [ppm] = 8.36 (s, 1H), 8.30* (s, 1H), 7.49 – 7.29 (m, 2H), 7.19 (d, *J* = 8.0 Hz, 1H), 7.15* (d, *J* = 8.0 Hz, 1H), 5.73* (d, *J* = 4.6 Hz, 1H), 5.03 (d, *J* = 4.6 Hz, 1H), 4.57 (q, *J* = 4.0 Hz, 1H), 4.51* (t, *J* = 4.5 Hz, 1H), 4.29 – 4.20 (m, 1H), 3.94 – 3.85 (m, 1H), 3.79 – 3.69 (m, 1H), 3.14 – 2.93 (m, 2H), 2.34* (s, 3H), 2.32 (s, 3H).

^{13}C NMR (75 MHz, Chloroform-*d*):
δ [ppm] = 203.3*, 202.3, 163.2, 142.1*, 141.3, 139.6, 139.6*, 132.2, 131.3*, 127.5, 127.1*, 127.0*, 126.9, 121.1, 121.0*, 77.9*, 77.8, 65.9*, 64.6, 61.2*, 58.8, 57.9, 55.3*, 38.6*, 36.9, 28.6*, 28.4.

ATR-IR:
$\tilde{\nu}$ [cm^{-1}] = 3073 (w), 3005 (w), 2972 (w), 2909 (w), 2868 (w), 1719 (s), 1667 (vs), 1597 (w), 1464 (m), 1452 (m), 1437 (m), 1395 (m), 1360 (m), 1311 (m), 1298 (m), 1252 (m), 1229 (m), 1219 (m), 1182 (m), 1167 (m), 1153 (m), 1105 (m), 1092 (m), 1082 (m), 1063 (m), 1040 (m), 1001 (m), 959 (m), 939 (w), 918 (w), 866 (w), 856 (m), 839 (m), 824 (m), 801 (m), 777 (m), 745 (m), 696 (m), 660 (m), 619 (m), 600 (m).

HRMS (MALDI-TOF):

m/*z* (+) = calc. 324.0230 $[M+H]^+$; found 324.0234 $[M+H]^+$.

Major diastereomer **3b**:

R_f (*n*-pentane/acetone 3:2): two distinct spots visible, centered on R_f: 0.21 which were not separable by column chromatography.

^{1}H NMR[3] (400 MHz, Chloroform-*d*):
δ [ppm] = 8.53 (s, 1H), 8.33* (s, 1H), 7.48 (s, 1H), 7.41 – 7.27 (m, 1H), 7.12 – 7.00 (m, 1H), 5.54* (d, *J* = 4.3 Hz, 1H), 4.94 (d, *J* = 4.3 Hz, 1H), 4.73 (d, *J* = 3.9 Hz, 1H), 4.51 – 4.27 (m, 2H), 3.88* (d, *J* = 3.6 Hz, 1H), 3.80* (dd, *J* = 12.5, 3.6 Hz, 1H), 3.62 (dd, *J* = 12.2, 4.1 Hz, 1H), 3.10 – 2.87 (m, 2H), 2.11 (s, 3H), 2.06* (s, 3H).

^{13}C NMR (75 MHz, Chloroform-*d*):
δ [ppm] = 203.9*, 202.4, 164.6*, 163.5, 140.8*, 140.6, 138.9, 138.9*, 131.7, 131.1*, 129.8*, 128.4, 126.9, 126.3*, 120.4, 120.4*, 78.2*, 78.2, 65.3*, 64.2, 60.4*, 59.8, 56.6, 55.8*, 37.8*, 37.6, 27.0*, 26.8.

ATR-IR:

$\tilde{\nu}$ [cm^{-1}] = 3005 (w), 2994 (w), 2970 (w), 2907 (w), 2868 (w), 1719 (s), 1669 (vs), 1597 (w), 1466 (m), 1437 (w), 1395 (s), 1360 (m), 1312 (w), 1296 (m), 1265 (m), 1219 (m), 1167 (s), 1153 (m, 1105 (s), 1061 (w), 1042 (w), 1017 (m), 959 (m), 843 (w), 820 (w), 801 (m), 777 (w), 745 (w), 733 (w), 696 (w), 660 (w), 619 (w), 600 (s).

HRMS (MALDI-TOF):

m/*z* (+) = calc. 324.0230 $[M+H]^+$; found 324.0236 $[M+H]^+$.

---

[2] The NMR spectra show a mixture of two rotamers in a ratio of ~6:1. Signals indicated with a star can be assigned to the minor isomer, while other signals show overlap or belong solely to the major isomer.
[3] The NMR spectra show a mixture of two rotamers in a ratio of ~3:2. Signals indicated with a star can be assigned to the minor isomer, while other signals show overlap or belong solely to the major isomer.

Synthesis of *N*-((4a*R*,9a*S*)-3-acetyl-4-formyl-2,3,4,4a,9,9a-hexahydroindeno[2,1-b][1,4]oxazin-6-yl)-2,2,2-trifluoroacetamide (4)

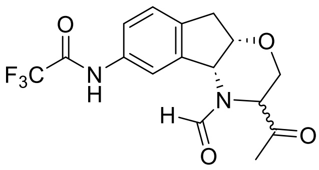

4

CuI (147 mg, 771 µmol, 0.1 eq.), K_2CO_3 (2.13 g, 15.4 mmol, 2.0 eq.), and 2 g 4 Å molecular sieves, trifluoroacetamide (1.31 g, 11.6 mmol, 1.5 eq.) and brominated ketone **3** (2.50 g, 7.71 mmol, 1.0 eq.) were added to a flame-dried Schlenk tube under argon atmosphere. The mixture was flushed with three high vacuum / argon cycles, after which 1,2-dimethylethylenediamine (68 mg, 83 µl, 771 µmol, 0.1 eq.) and 7.7 ml of dry, degassed 1,4-dioxane (1 M) were added. The mixture was then heated to 80°C under Ar and stirred at this temperature for 45 h. The reaction mixture was subsequently filtered over a SiO_2 plug (with help of EtOAc for washing) and following this, the solvent of the filtrate was removed *in vacuo*. The crude product mixture was then purified by column chromatography (SiO_2, DCM/Acetone 15:1 → 10:1 → 5:1) to yield the product **4** as a slightly beige foam (2.05 g, 5.76 mmol, 75%).

Additionally, small amounts of starting material **3b** could be recovered (203 mg, 626 µmol, 8%) after chromatography.

R_f (DCM/acetone 10:1): 0.13

^{1}H NMR[4] (400 MHz, Chloroform-*d*):
δ [ppm] = 9.26* (s, 1H), 8.87 (s, 1H), 8.56 (s, 1H), 8.37* (s, 1H), 7.88* (dd, *J* = 8.3, 2.0 Hz, 1H), 7.67 – 7.55 (m, 1H), 7.33* (t, *J* = 1.5 Hz, 1H), 7.20 (t, *J* = 8.3 Hz, 1H), 5.55* (d, *J* = 4.4 Hz, 1H), 4.96 (d, *J* = 4.3 Hz, 1H), 4.74 (d, *J* = 4.1, 1.4 Hz, 1H), 4.52 – 4.33 (m, 2H), 3.94 – 3.82 (m, 1H), 3.70 (dd, *J* = 12.4, 4.2 Hz, 1H), 3.19 – 2.93 (m, 2H), 2.16 – 2.04 (m, 3H).

^{13}C NMR (101 MHz, Chloroform-*d*):
δ [ppm] = 204.3*, 202.7, 165.4*, 163.7*, 155.1 (q, *J* = 38.5 Hz), 155.1* (q, *J* = 38.5 Hz), 139.6, 139.5*, 137.7, 137.6*, 134.9*, 134.7, 126.0, 125.4*, 121.4, 120.7*, 118.9*, 117.9, 116.0 (q, *J* = 288.7 Hz), 115.9 (q, *J* = 288.4 Hz), 78.4*, 78.4, 65.5*, 64.4, 60.6*, 60.0, 56.9, 56.1*, 37.9*, 37.7, 27.1*, 27.0.

^{19}F NMR (376 MHz, Chloroform-*d*):
δ [ppm] = -75.42* (s, 3F), -75.53 (s, 3F).

ATR-IR:
$\tilde{\nu}$ [cm^{-1}] = 3431 (w), 3165 (w), 2932 (w), 1728 (w), 1609 (w), 1541 (w), 1499 (w), 1443 (w), 1354 (m), 1273 (s), 1213 (w), 1109 (s), 1001 (w), 945 (w), 934 (w), 885 (m), 858 (w), 839 (w), 808 (w),779 (w), 762 (w), 745 (w), 712 (m), 681 (m), 669 (m), 617 (w), 579 (w), 515 (w), 449 (w).

HRMS (MALDI-TOF):

m/*z* (+) = calc. 357.1057 [M+H]$^+$; found 357.1060 [M+H]$^+$.

[4] Through epimerization, regardless of the starting material used (**3a** or **3b**), only one diastereomer of **4** is obtained in significant amounts, which consists of two amide rotamers in a ratio of ~ 45:55. Signals indicated with a star can be assigned to the minor isomer, while other signals show overlap or belong solely to the major isomer.

Synthesis of (5a*S*,5a'*S*,10b*R*,10b'*R*)-2,2'-(2-methyl-1,3-phenylene)bis(9-bromo-3-methyl-2,5a,6,10b-tetrahydro-4H-imidazo[1,5-d]indeno[2,1-b][1,4]oxazin-11-ium) chloride (5a^{Cl})

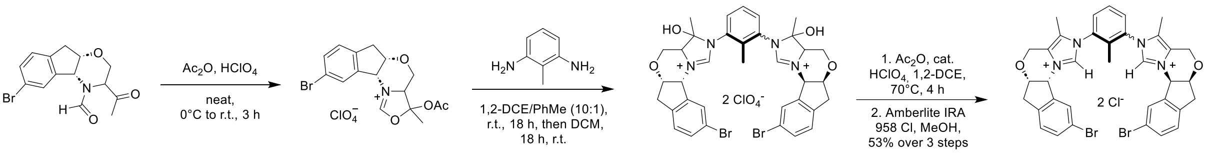

3 SI-5 SI-6 5a^{Cl}

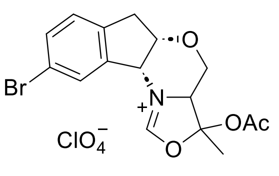

SI-5

In a flame-dried Schlenk flask, the *N*-formyl ketone **3** (2.83 g, 8.74 mmol, 1.0 eq.) was suspended in Ac_2O (14.3 g, 13.2 ml, 140 mmol, 16.0 eq.). The mixture was cooled to 0°C and $HClO_4$ (70% in H_2O, 1.50 g, 895 µl, 10.48 mmol, 1.2 eq.) was added dropwise over 20 minutes. The mixture was then warmed to room temperature and stirred at room temperature for 3 hours. The reaction mixture was triturated thrice with dry Et_2O under Ar (first with 100 ml, then 50 ml each). The supernatant was decanted each time and the resulting light brown solid dried under high vacuum at room temperature to yield the crude oxazolinium perchlorate salt **SI-5** as a brownish solid. Due to its low stability, it was used immediately in the next step.

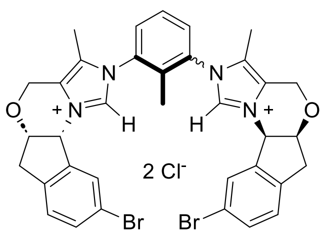

5a^{Cl}

The crude oxazolinium salt **SI-5** (4.08 g, 8.74 mmol, 2.2 eq.) and 2,6-diaminotoluene (486 mg, 3.97 mmol, 1.0 eq.) were added to a flame-dried Schlenk flask under Ar. The mixture was dissolved in 20 ml 1,2-dichloroethane and then 1.8 ml toluene were added. The mixture was stirred for 18 h at room temperature. The solvent was removed under high vacuum at room temperature and the residue was triturated twice with dry Et_2O under Ar. The residue was again dissolved in 20 ml of dry DCM and the mixture was stirred for another 18 h at room temperature. The solvent was removed under high vacuum at room temperature and the residue (crude **SI-6**) was dried under high vacuum.

The mixture of crude aminal intermediates **SI-6** was then dissolved in 1,2-dichloroethane (52 ml, 0.077 M) and Ac_2O (1.01 g, 957 µl, 9.94 mmol, 2.5 eq.) and $HClO_4$ (70% in H_2O, 114 mg, 69 µl, 795 µmol, 0.2 eq) were added. The mixture was stirred at 70 °C for two hours, during which a dark red solution was formed. Another 0.2 eq. of $HClO_4$ were added and the mixture was stirred for a further two hours at 70 °C, after which the reaction mixture was cooled down to room temperature and the solvent removed under high vacuum at room temperature. The residue was triturated thrice with Et_2O (3 x 50 ml) and the solvent decanted. The dark red/brown residue was dried under vacuum and subsequently suspended in MeOH (20 ml). 11.9 g of Amberlite IRA 958 Cl were added, and the mixture was stirred for 30 minutes, during which the solid (perchlorate salts) dissolved. The ion exchange resin was filtered off and rinsed with further MeOH. The solvent was removed *in vacuo* to receive the crude product as a red/brown solid. Purification was achieved by column chromatography (SiO_2, DCM/MeOH 8:1 → 4:1 → 1:1), yielding the product **5a^{Cl}** as a light brown solid (1.63 g, 2.12 mmol, 53% yield).

^{1}H NMR (400 MHz, DMSO-*d*$_6$):
δ [ppm] = 10.78 – 10.26 (m, 2H), 8.22 – 7.65 (m, 5H), 7.63 – 7.47 (m, 2H), 7.38 (d, *J* = 8.0 Hz, 2H), 6.27 – 6.01 (m, 2H), 5.20 – 4.74 (m, 6H), 3.47 – 3.38 (m, 2H), 3.10 (d, *J* = 17.1 Hz, 2H), 2.21 – 1.94 (m, 9H).

^{13}C NMR (101 MHz, DMSO-d_6):

δ [ppm] = 140.2, 139.8, 139.5, 136.5, 136.0, 133.5, 131.8, 131.5, 131.1, 130.8, 130.6, 128.8, 128.6, 128.3, 127.7, 127.4, 126.9, 125.1, 125.0, 124.7, 124.5, 123.1, 123.0, 122.9, 119.8, 77.2, 59.9, 59.8, 59.2, 59.0, 36.8, 12.7, 12.5, 8.1, 7.9.

ATR-IR:

$\tilde{\nu}$ [cm^{-1}] = 3383 (m), 3096 (w), 2959 (m), 2924 (m), 2345 (w), 2075 (w), 1636 (m), 1537 (vs), 1474 (s), 1447 (w), 1408 (w), 1373 (w), 1341 (w), 1317 (w), 1250 (w), 1233 (w), 1207 (w), 1186 (w), 1157 (w), 1134 (w), 1105 (s), 1084 (vs), 1067 (s), 1036 (m), 991 (w), 957 (w), 935 (w), 885 (w), 847 (w), 806 (vs), 781 (w), 754 (w), 721 (w), 696 (w), 650 (w), 633 (w), 503 (w), 490 (w).

HRMS (MALDI-TOF):

m/*z* (+) = calc. 697.0809 [M-H]$^+$; found 697.0807 [M-H]$^+$.

Synthesis of (5a*S*,5a'*S*,10b*R*,10b'*R*)-2,2'-(2-methyl-1,3-phenylene)bis(9-bromo-3-methyl-2,5a,6,10b-tetrahydro-4*H*-imidazo[1,5-d]indeno[2,1-b][1,4]oxazin-11-ium) tetrakis(3,5-bis(trifluoromethyl)phenyl)borate (5a^{BArF})

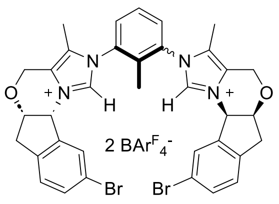

5a^{BArF}

Bis(imidazolium) chloride salt **5a^{Cl}** (120 mg, 156 µmol, 1.0 eq.) and NaBAr$^F{}_4$ (276 mg, 311 µmol, 2.0 eq.) were suspended in dry acetone (1.05 ml, 0.15 M), the formed mixture was then stirred for 2.5 h at 42°C. The reaction mixture was then cooled down to room temperature and the solvent was removed *in vacuo*. Afterwards, the obtained residue was suspended in Et_2O, and the insoluble residue filtered off (using a syringe filter, 0.22 µm). The solvent from the filtrate was evaporated *in vacuo* and the obtained residue then extracted with $CHCl_3$/DCM (1:1). The residue obtained from this evaporation of this filtrate was then precipitated multiple times from DCM by addition of *n*-pentane and the oily precipitate residue sonicated in presence of *n*-pentane. The product was then co-evaporated with DCM and dried under high vacuum at 45°C to remove residual solvent, to yield the product **5a^{BArF}** as a slightly yellowish solid (351 mg, 145 µmol, 93%).

^{1}H NMR (400 MHz, DCM-d_2):

δ [ppm] = 8.69 (s, 1H), 8.65 (s, 1H), 7.83 – 7.76 (m, 1H), 7.73 – 7.68 (m, 18H), 7.63 (d, *J* = 8.2 Hz, 1H), 7.57 – 7.53 (m, 9H), 7.42 (s, 1H), 7.36 (t, J = 7.5 Hz, 2H), 7.26 (d, *J* = 12.5 Hz, 1H), 5.75 – 5.62 (m, 2H), 4.99 – 4.76 (m, 6H), 3.45 – 3.24 (m, 4H), 2.11 – 1.93 (m, 9H).

^{13}C NMR (101 MHz, DCM-d_2):

δ [ppm] = 162.4 (dd, *J* = 49.8 Hz), 140.3, 137.2, 137.1, 136.8, 135.4, 134.6, 134.4, 133.9, 133.8, 133.5, 133.4, 132.7, 132.5, 132.4, 132.3, 132.3, 131.6, 131.4, 129.5 (qq, *J* = 31.5, 2.7 Hz), 128.9, 128.8, 127.8 – 127.4 (m), 126.9, 126.8 (d, *J* = 3.2 Hz), 126.7, 126.4, 126.2, 125.2 (q, *J* = 272.5 Hz), 121.9, 121.8, 121.8, 118.2 (p, *J* = 3.6, 2.8 Hz), 77.9, 77.7, 61.5, 61.4, 59.6, 59.6, 59.3, 37.6, 37.5, 37.1, 13.5, 13.2, 8.9, 8.7.

^{19}F NMR (376 MHz, DCM-d_2):

δ [ppm] = -62.75 (s, 48F).

ATR-IR:

$\tilde{\nu}$ [cm^{-1}] = 3163 (w), 2926 (w), 2353 (w), 1609 (w), 1541 (w), 1474 (w), 1354 (s), 1273 (vs), 1111 (vs), 1092 (vs), 1038 (w), 934 (w), 885 (m), 839 (m), 806 (m), 745 (w), 712 (s), 681 (s), 669 (s), 581 (w), 503 (w), 449 (w).

HRMS (MALDI-TOF):

m/z (+) = calc. 1565.1593 [M-BAr$^F{}_4$]$^+$; found 1565.1495 [M-BAr$^F{}_4$]$^+$.

$[\alpha]_D^{20}$ (c: 0.50, CH_2Cl_2): -45.4°

Synthesis of (5a*S*,5a'*S*,10b*R*,10b'*R*)-2,2'-((1*R*,3*S*)-2-methyl-1,3-phenylene)bis(9-bromo-1-iodo-3-methyl-2,5a,6,10b-tetrahydro-4H-imidazo[1,5-d]indeno[2,1-b][1,4]oxazin-11-ium) triflate (7a^{OTf})

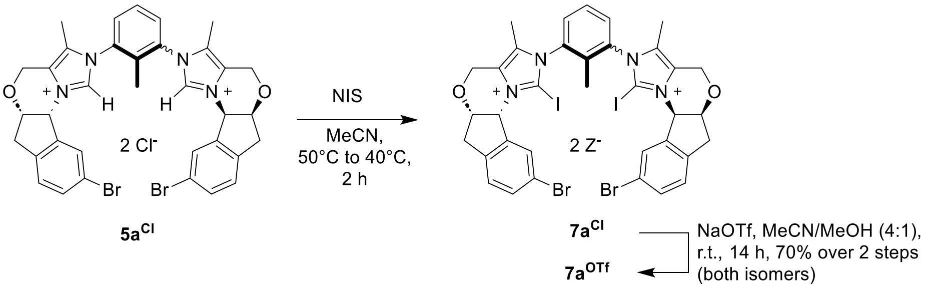

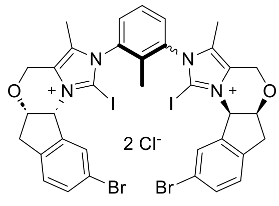

7a^{Cl}

Bis(imidazolium) chloride salt **5a^{Cl}** (400 mg, 519 µmol, 1.0 eq.) was suspended in 10.4 ml dry MeCN (0.05 M), then *N*-iodosuccinimide (257 mg, 1.14 mmol, 2.2 eq.) was added and the mixture was then heated to 50°C, after 15 minutes, it was cooled down to 40°C and stirred at this temperature for 2 hours. The solvent was then removed *in vacuo* and the obtained mixture was three times precipitated from MeCN/MeOH (4:1) by addition of Et_2O (3-4 volume equivalents) and the obtained yellowish solid (crude **7a^{Cl}**, 403 mg, 394 µmol, 76%) was finally dried under high vacuum. The crude product mixture still contained residual *N*-iodosuccinimide and solvent and was used in the next step without further purification.

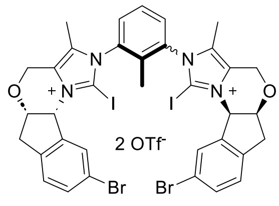

7a^{OTf}

Bis(iodoimidazolium) chloride salt **7a^{Cl}** (400 mg, 391 µmol, 1.0 eq.) was dissolved in 4.3 ml 4:1 MeCN/MeOH (0.09 M), then NaOTf (135 mg, 782 µmol, 2.0 eq.) was added and the mixture was stirred for 14 h at room temperature (immediate precipitation was observed upon addition of NaOTf). The solvent was then removed *in vacuo* and the residue resuspended in MeCN and filtered (syringe filter, 0.22 µm). The solvent of the resulting filtrate was then removed *in vacuo*. The mixture was once precipitated from a minimal amount of MeCN by addition of Et_2O (~10 volume equivalents). The precipitate was dried under high vacuum to yield the mixture of iodinated atropisomers **7a^{OTf}** (451 mg, 361 µmol, 92%) as a yellowish solid.

Partial separation of atropisomers was then achieved by the following procedure: To the mixture of isomers, 4 ml DCM were added and the suspension was sonicated, upon which a precipitate formed. The solid was separated from the supernatant filtrate and the solvent of the filtrate was removed *in vacuo*. This procedure was repeated on both the precipitate and the residue gained from the filtrate to obtain two fractions, enriched in either atropisomer. The fraction gained from the filtrates was obtained as a yellowish solid (237 mg, 189 µmol, 48% based on total starting material, referred to as fraction 1 in the following) and the fraction from the precipitates as a slightly beige solid (173 mg, 138 µmol, 35% based on total starting material, referred to as fraction 2).

Major fraction (**7a**^{OTf}, fraction 1):

^{1}H NMR (400 MHz, Acetonitrile-*d*$_3$):
δ [ppm] = 8.00 – 7.71 (m, 3H), 7.66 (s, 1H), 7.58 – 7.42 (m, 3H), 7.41 – 7.28 (m, 2H), 5.89 – 5.66 (m, 2H), 5.05 – 4.70 (m, 6H), 3.36 (d, *J* = 17.0 Hz, 2H), 3.12 (dd, *J* = 17.1, 3.5 Hz, 2H), 2.20 – 2.04 (m, 6H), 1.97 – 1.91 (m, 3H).

^{13}C NMR (101 MHz, Acetonitrile-*d*$_3$):
δ [ppm] = 141.8, 141.8, 141.71, 141.7, 140.2, 140.1, 140.1, 136.8, 136.6, 136.5, 136.4, 136.2, 135.3, 133.6, 133.3, 133.3, 133.2, 131.4, 131.1, 130.4, 130.2, 130.0, 129.6, 129.6, 129.0, 129.0, 128.9, 128.7, 121.9 (q, *J* = 320.5 Hz) 120.6, 120.5, 101.5, 101.2, 99.1, 79.4, 79.4, 63.6, 63.4, 61.04, 61.01, 37.9, 37.9, 14.1, 13.9, 10.2, 10.1, 10.0.

^{19}F NMR (376 MHz, Acetonitrile-*d*$_3$):
δ [ppm] = -79.09 (s, 6F).

ATR-IR:
ṽ [cm^{-1}] = 3088 (w), 2967 (w), 2928 (w), 2853 (w), 2372 (w), 2322 (w), 1653 (w), 1636 (w), 1474 (m), 1456 (m), 1437 (m), 1373 (w), 1339 (w), 1317 (w), 1271 (s), 1240 (s), 1223 (s), 1153 (w), 1109 (w), 1088 (w), 1067 (w), 1026 (s), 993 (w), 935 (w), 903 (w), 891 (w), 853 (w), 812 (w), 756 (w), 725 (w), 681 (w), 635 (s), 573 (w), 517 (w), 424 (w).

HRMS[5] (MALDI-TOF):

m/*z* (+) = calc. 974.9353 [M-OTf-I+H]$^+$; found 974.9353 [M-OTf-I+H]$^+$.

Minor fraction (**7a**^{OTf}, fraction 2):

^{1}H NMR (400 MHz, Acetonitrile-*d*$_3$):
δ [ppm] = 8.03 – 7.74 (m, 3H), 7.73 – 7.62 (m, 1H), 7.62 – 7.49 (m, 2H), 7.46 (s, 1H), 7.35 (d, *J* = 8.0 Hz, 2H), 5.85 – 5.69 (m, 2H), 5.03 – 4.76 (m, 6H), 3.45 – 3.28 (m, 2H), 3.12 (dd, *J* = 16.9, 4.1 Hz, 2H), 2.19 – 2.08 (m, 6H), 1.98 – 1.86 (m, 3H).

^{13}C NMR (101 MHz, Acetonitrile-*d*$_3$):
δ [ppm] = 141.8, 141.7, 141.7, 140.3, 140.1, 140.0, 136.9, 136.7, 136.5, 136.5, 136.5, 136.4, 135.3, 133.6, 133.3, 133.3, 133.2, 133.2, 133.1, 131.3, 131.2, 131.1, 130.4, 130.2, 130.0, 129.9, 129.6, 129.6, 129.5, 129.2, 129.1, 128.9, 128.9, 128.8, 128.8, 128.5, 121.9 (q, *J* = 320.6 Hz), 120.6, 120.5, 99.4, 98.9, 79.5, 79.4, 63.7, 63.6, 63.5, 63.4, 61.0, 61.0, 37.9, 37.8, 14.2, 13.9, 13.8, 10.2, 10.0, 10.0.

^{19}F NMR (376 MHz, Acetonitrile-*d*$_3$):
δ [ppm] = -79.13 (s, 6F).

ATR-IR:
ṽ [cm^{-1}] = 3102 (w), 2928 (w), 2311 (w), 1749 (w), 1636 (w), 1474 (w), 1447 (w), 1437 (w), 1373 (w), 1339 (w), 1317 (w), 1273 (s), 1242 (s), 1223 (s), 1155 (s), 1107 (m), 1088 (m), 1067 (w), 1026 (vs), 993 (w), 937 (w), 891 (w), 853 (w), 814 (w), 785 (w), 758 (w), 745 (w), 723 (w), 679 (w), 635 (vs), 573 (w), 517 (m).

[5] Under the conditions used for mass spectrometry, one iodine was lost to proto-deiodination, this resulted in the peak for the proto-deiodinated cation with one anion for this fraction and the deprotonated proto-deiodinated cation for the minor fraction. Similar behavior was observed for many of the halogen bond donors described in this report.

HRMS (MALDI-TOF):

m/*z* (+) = calc. 824.9755 [M-2OTf-I]$^+$; found 824.9752 [M-2OTf-I]$^+$.

Synthesis of (5a*S*,5a'*S*,10b*R*,10b'*R*)-2,2'-((1*R*,3*S*)-2-methyl-1,3-phenylene)bis(9-bromo-1-iodo-3-methyl-2,5a,6,10b-tetrahydro-4H-imidazo[1,5-d]indeno[2,1-b][1,4]oxazin-11-ium) tetrakis(3,5-bis(trifluoromethyl)phenyl)borate (7a^{BArF})

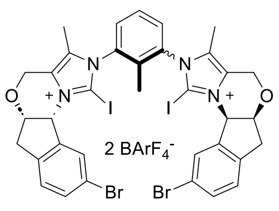

7a^{BArF}

Bis(iodoimidazolium) triflate salt **7a^{OTf}** (fraction 1) (312 mg, 237 µmol, 1 eq.) and NaBAr$^F_4$ (421 mg, 475 µmol, 2.0 eq.) were suspended in 2.4 ml dry acetone (0.1 M) and the mixture was stirred for 2.5 h at 42°C. The mixture was cooled to room temperature, the solvent was removed *in vacuo* and the residue dried under high vacuum. The residue was then extracted with Et_2O and the solvent of the filtrate removed *in vacuo*. The obtained residue was afterwards extracted with $CHCl_3$/DCM (1:1). The solvent of the filtrate thus obtained was removed *in vacuo* and the gained residue was precipitated with *n*-pentane from DCM. The product was then co-evaporated with DCM and dried under high vacuum at 45°C to remove residual solvent, yielding the product **7a^{BArF}** (fraction 1) as a slightly amber colored solid (636 mg, 231 µmol, 98%).

^{1}H NMR (400 MHz, DCM-*d*$_2$):
δ [ppm] = 7.97 – 7.78 (m, 2H), 7.71 (s, 16H), 7.67 – 7.49 (m, 12H), 7.40 – 7.26 (m, 3H), 5.70 (dd, *J* = 18.2, 3.6 Hz, 2H), 4.95 – 4.74 (m, 6H), 3.46 – 3.34 (m, 2H), 3.34 – 3.23 (m, 2H), 2.21 – 2.09 (m, 6H), 1.98 – 1.81 (m, 3H).

^{13}C NMR (101 MHz, DCM-*d*$_2$):
δ [ppm] = 162.4 (dd, *J* = 49.8 Hz), 140.6, 140.6, 140.6, 137.6, 137.4, 135.9, 135.8, 135.8, 134.6, 134.2, 134.1, 134.0, 134.0, 133.4, 133.2, 133.2, 132.5, 132.3, 131.1, 130.9, 130.7, 130.6, 130.5, 129.6 (qq, *J* = 31.8, 2.7 Hz), 128.8, 128.8, 128.7, 128.3, 127.9, 127.8, 125.2 (q, *J* = 272.5 Hz) 121.4, 121.4, 121.3, 118.2 (p, *J* = 3.9 Hz), 96.2, 96.1, 93.6, 78.7, 78.7, 78.6, 63.9, 63.9, 63.8, 60.0, 60.0, 59.9, 37.8, 37.8, 13.9, 13.8, 10.5, 10.3, 10.2.

^{19}F NMR (376 MHz, DCM-*d*$_2$):
δ [ppm] = -62.74 (s, 48F).

ATR-IR:
$\tilde{\nu}$ [cm^{-1}] = 3032 (w), 2934 (w), 2376 (w), 2311 (w), 1609 (w), 1474 (w), 1354 (s), 1317 (w), 1273 (vs), 1111 (vs), 1092 (vs), 934 (w), 885 (m), 853 (w), 839 (m), 810 (w), 764 (w), 745 (s), 712 (s), 681 (s), 669 (s), 619 (w), 581 (w), 503 (w), 447 (w).

HRMS (MALDI-TOF):

m/*z* (+) = calc. 1687.0502 [M-BAr$^F_4$-I]$^+$; found 1687.0513 [M-BAr$^F_4$-I]$^+$.

$[\alpha]_D^{20}$ (c: 0.56, CH_2Cl_2): -152.9°

Synthesis of (5a*S*,5a'*S*,10b*R*,10b'*R*)-2,2'-(2-methyl-1,3-phenylene)bis(9-bromo-3-methyl-2,5a,6,10b-tetrahydro-4H-imidazo[1,5-d]indeno[2,1-b][1,4]oxazin-11-ium) chloride ($5a^{Cl}$)

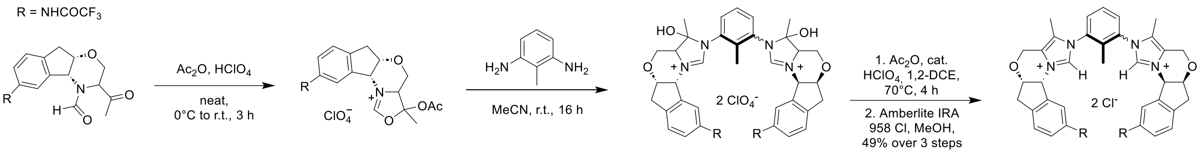

4 **SI-7** **SI-8** $\mathbf{5b^{Cl}}$

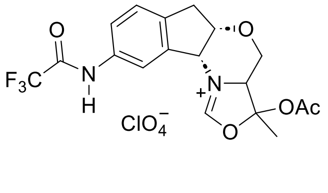

SI-7

In a flame-dried Schlenk flask, the *N*-formyl ketone **4** (3.00 g, 8.42 mmol, 1.0 eq.) was suspended in Ac_2O (13.8 g, 12.7 ml, 135 mmol, 16.0 eq.). The mixture was cooled to 0°C and $HClO_4$ (70% in H_2O, 1.45 g, 863 µl, 10.1 mmol, 1.2 eq.) was added dropwise over 20 minutes. The mixture was then warmed to room temperature and stirred at room temperature for 3 hours. The reaction mixture was triturated thrice with dry Et_2O under Ar (first with 100 ml, then 50 ml each). The supernatant was decanted each time and the resulting light brown solid dried under high vacuum at room temperature to yield the crude oxazolinium perchlorate salt **SI-7** as a brownish solid. Due its low stability, it was used immediately in the next step.

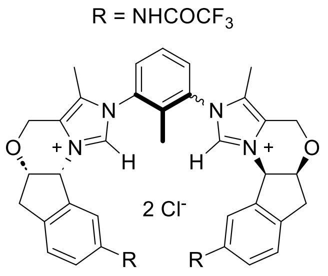

$\mathbf{5b^{Cl}}$

The crude oxazolinium salt **SI-7** (4.20 g, 8.42 mmol, 2.2 eq.) and 2,6-diaminotoluene (468 mg, 3.83 mmol, 1.0 eq.) were added to a flame-dried Schlenk flask under Ar. The mixture was dissolved in 19 ml dry MeCN and stirred for 16 h at room temperature. The solvent was removed under high vacuum at room temperature and the residue was triturated twice with dry Et_2O under Ar, after which the residue (crude **SI-8**) was dried under high vacuum at room temperature.

The mixture of crude aminal intermediates **SI-8** was then suspended in 1,2-dichloroethane (50 ml, 0.077 M) and Ac_2O (977 mg, 904 µl, 9.57 mmol, 2.5 eq.) and $HClO_4$ (70% in H_2O, 110 mg, 65 µl, 765 µmol, 0.2 eq) were added. The mixture was stirred at 70 °C for two hours, after which, another 0.2 eq. of $HClO_4$ were added and the mixture was stirred for a further two hours at 70 °C. Subsequently, the reaction mixture was cooled down to room temperature and the solvent removed under high vacuum at room temperature. The residue was triturated thrice with Et_2O (3 x 50 ml) and the solvent decanted. The dark red/brown residue was dried under vacuum and subsequently dissolved in MeOH (20 ml). 11.5 g of Amberlite IRA 958 Cl were added, and the mixture was stirred for 1 hour. The ion exchange resin was filtered off and rinsed with further MeOH. The solvent was removed *in vacuo* to receive the crude product as a brownish solid. Purification was achieved by column chromatography (SiO_2, DCM/MeOH 8:1 → 4:1 → 1:1), yielding the product $\mathbf{5b^{Cl}}$ as a beige solid (1.58 g, 1.89 mmol, 49% yield).

^{1}H NMR (400 MHz, DMSO-*d*$_6$):
δ [ppm] = 11.62 – 11.38 (m, 2H), 10.39 – 10.21 (m, 2H), 8.18 – 7.80 (m, 5H), 7.69 – 7.39 (m, 4H), 6.10 – 5.94 (m, 2H), 5.09 (t, *J* = 14.0 Hz, 2H), 4.94 (t, *J* = 3.9 Hz, 2H), 4.85 (d, *J* = 14.8 Hz, 2H), 3.43 (d, *J* = 17.2 Hz, 2H), 3.13 (d, *J* = 17.0 Hz, 2H), 2.17 – 1.93 (m, 9H).

^{13}C NMR {^{1}H, ^{19}F} (101 MHz, Methanol-*d*$_4$):
δ [ppm] = 156.9, 156.7, 139.6, 139.4, 139.0, 138.9, 137.2, 137.1, 136.9, 136.5, 136.0, 135.7, 135.4, 135.2, 135.1, 132.7, 132.6, 132.4, 130.3, 130.2, 130.1, 127.4, 127.3, 127.1, 127.0, 125.6, 125.5, 125.3, 123.6, 123.5, 123.2, 118.0, 117.5, 117.4, 117.2, 117.1, 79.0, 79.0, 62.1, 60.5, 60.5, 38.3, 38.2, 13.4, 8.4, 8.3.

^{19}F NMR[6] (376 MHz, DMSO-d_6):
δ [ppm] = 73.50 (s, 3F), -73.61 (s, 3F), -73.69 (s, 3F), -73.77 (s, 3F).

ATR-IR:
$\tilde{\nu}$ [cm^{-1}] = 3379 (w), 3013 (w), 2922 (w), 2862 (w), 2789 (w), 1713 (s), 1609 (w), 1539 (m), 1495 (m), 1437 (w), 1373 (w), 1341 (w), 1319 (w), 1288 (w), 1244 (w), 1192 (s), 1144 (vs), 1101 (s), 1082 (s), 1038 (m), 993 (w), 897 (w), 858 (w), 810 (m), 752 (w), 723 (m), 656 (w), 617 (w), 517 (m), 482 (w), 430 (w).

HRMS (MALDI-TOF):
m/z (+) = calc. 763.2462 [M-2Cl-H]$^+$; found 763.2451 [M-2Cl-H]$^+$.

Synthesis of (5a*S*,5a'*S*,10b*R*,10b'*R*)-2,2'-(2-methyl-1,3-phenylene)bis(3-methyl-9-(2,2,2-trifluoroacetamido)-2,5a,6,10b-tetrahydro-4*H*-imidazo[1,5-d]indeno[2,1-b][1,4]oxazin-11-ium) tetrakis(3,5 bis(trifluoromethyl)phenyl)borate (5b^{BArF})

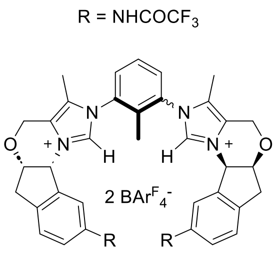

5b^{BArF}

Bis(imidazolium) chloride salt **5b^{Cl}** (74.0 mg, 88.6 µmol, 1.0 eq.) and NaBAr$^F{}_4$ (161 mg, 182 µmol, 2.05 eq.) were suspended in dry acetone (0.59 ml, 0.15 M), the formed mixture was then stirred for 2.5 h at 42°C. The reaction mixture was then cooled down to room temperature and the solvent was removed *in vacuo*. Afterwards, the obtained residue was suspended in Et_2O, and the insoluble residue filtered off (using a syringe filter, 0.22 µm). The solvent from the filtrate was evaporated *in vacuo* and the obtained residue then extracted with $CHCl_3$/DCM (1:1). The residue obtained from this evaporation of this filtrate was then precipitated multiple times from DCM by addition of *n*-pentane and the oily precipitate residue sonicated in presence of *n*-pentane. The product was then co-evaporated with DCM and dried under high vacuum at 45°C to remove residual solvent, to yield the product **5b^{BArF}** as a slightly yellowish solid (185 mg, 74.4 µmol, 84%).

^{1}H NMR (400 MHz, DCM-d_2):
δ [ppm] = 8.91 – 8.76 (m, 2H), 8.24 – 8.00 (m, 4H), 7.82 – 7.63 (m, 19H), 7.54 (s, 8H), 7.45 – 7.40 (m, 2H), 7.22 – 6.99 (m, 2H), 5.79 – 5.54 (m, 2H), 5.00 – 4.90 (m, 4H), 4.79 (d, *J* = 15.1 Hz, 2H), 3.45 (dd, *J* = 17.8, 4.4 Hz, 2H), 3.39 – 3.26 (m, 2H), 2.24 – 1.77 (m, 9H).

^{13}C NMR (101 MHz, DCM-d_2):
δ [ppm] = 162.4 (dd, *J* = 49.8 Hz), 156.0 (q, *J* = 38.1 Hz), 155.9 (q, *J* = 38.1 Hz), 139.2, 138.9, 136.8, 136.3, 135.8, 135.8 – 135.6 (m), 135.5, 134.0, 133.3, 133.0, 132.8, 132.4, 131.9, 131.3, 130.9, 129.5 (qq, *J* = 32.0, 2.9 Hz), 128.2, 127.9, 126.0, 125.3 (q, *J* = 272.4 Hz), 123.0, 122.5, 122.3, 118.2 (p, *J* = 3.8 Hz), 117.4, 115.9, 114.7, 114.5, 78.2, 77.8, 61.8, 59.8, 59.4, 38.1, 37.3, 13.5, 13.0, 8.8, 8.7, 8.4.

^{19}F NMR (376 MHz, DCM-d_2):
δ [ppm] = -62.82 (s, 48F), -76.19 – -76.68 (m, 6F).

[6] The trifluoromethyl fluorine atoms of **5b^{Cl}** give rise to four signals, as a mixture of two atropisomers with two amide rotamers each is present. This continues to be the case for the compounds deriving from this, although the varying shifts in some cases lead to signals overlapping.

ATR-IR:

ṽ [cm^{-1}] = 3431 (w), 3165 (w), 2932 (w), 1728 (w), 1609 (w), 1541 (w), 1499 (w), 1443 (w), 1354 (m), 1273 (s), 1213 (w), 1109 (s), 945 (w), 934 (w) , 885 (m), 808 (w), 762 (w), 745 (w), 725 (w), 712 (m), 681 (m), 669 (m), 617 (w), 604 (w), 579 (w), 515 (w), 449 (w).

HRMS (MALDI-TOF):

m/*z* (+) = calc. 1627.3190 [M-BAr$^F_4$]$^+$; found 1627.3231 [M-BAr$^F_4$]$^+$.

[α]$_D^{20}$ (c: 0.53, CH_2Cl_2): -52.2°

Synthesis of (5a*S*,5a'*S*,10b*R*,10b'*R*)-2,2'-(2-methyl-1,3-phenylene)bis(1-iodo-3-methyl-9-(2,2,2-trifluoroacetamido)-2,5a,6,10b-tetrahydro-4H-imidazo[1,5-d]indeno[2,1-b][1,4]oxazin-11-ium) chloride (7b^{Cl})

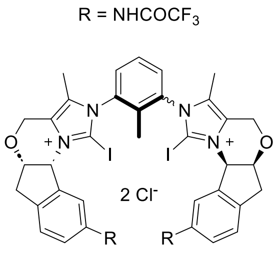

7b^{Cl}

Bis(imidazolium) chloride salt **5b^{Cl}** (500 mg, 598 µmol, 1.0 eq.) was suspended in 12 ml dry MeCN (0.05 M), then *N*-iodosuccinimide (296 mg, 1.32 mmol, 2.2 eq.) was added and the mixture was then heated to 50°C, after 15 minutes, it was cooled down to 40°C and stirred at this temperature for 2 hours. The solvent was then removed *in vacuo* and the obtained mixture was precipitated five times from MeCN/MeOH (4:1) by addition of Et_2O (3-4 volume equivalents) and the obtained yellowish solid (**7b^{Cl}**, 530 mg, 486 µmol, 81%) was finally dried under high vacuum.

^{1}H NMR (400 MHz, DMSO-*d*$_6$):

δ [ppm] = 11.56 – 11.21 (m, 2H), 8.06 – 7.80 (m, 5H), 7.76 – 7.57 (m, 2H), 7.52 – 7.41 (m, 2H), 6.11 – 5.87 (m, 2H), 5.15 – 4.69 (m, 6H), 3.44 (d, *J* = 16.5 Hz, 2H), 3.06 (d, *J* = 16.5 Hz, 2H), 2.15 – 1.85 (m, 9H).

^{13}C NMR (101 MHz, DMSO-*d*$_6$):

δ [ppm] = 155.1 – 153.6 (m), 138.2, 138.2, 138.1, 138.0, 137.9, 135.5, 135.5, 135.4, 135.1, 135.1, 134.8, 134.7, 133.8, 132.0, 131.9, 131.8, 129.8, 129.7, 129.6, 127.7, 127.5, 126.8, 126.6, 126.2, 126.1, 126.0, 125.9, 125.7, 125.6, 121.5, 121.5, 121.4, 121.1, 117.9, 117.7, 117.2, 117.1, 116.9, 116.6, 116.3, 114.3, 114.2, 114.2, 114.1, 113.1, 109.8, 107.9, 78.3, 62.1, 61.9, 61.9, 61.8, 59.5, 59.4, 59.3, 59.2, 36.8, 36.7, 13.3, 13.0, 12.6, 9.3, 9.2, 9.1, 9.0.

^{19}F NMR (376 MHz, DMSO-*d*$_6$):

δ [ppm] = -73.30 – -73.93 (m, 6F).

ATR-IR:

ṽ [cm^{-1}] = 3375 (m), 3215 (m), 3038 (m), 2957 (m), 2916 (m), 1713 (s), 1611 (m), 1557 (m), 1489 (m), 1479 (m), 1435 (m), 1371 (w), 1342 (w), 1319 (w), 1287 (m), 1242 (m), 1204 (s), 1165 (vs), 1146 (vs), 1101 (s), 1082 (m), 1038 (m), 991 (w), 901 (w), 864 (w), 843 (w), 818 (m), 785 (w), 752 (w), 725 (m), 617 (w), 517 (w).

HRMS (MALDI-TOF):

m/*z* (+) = calc. 1015.0395 [M-2Cl-H]$^+$; found 1015.0349 [M-2Cl-H]$^+$.

calc. 889.1429 [M-2Cl-H-I]$^+$; found 889.1425 [M-2Cl-H-I]$^+$.

Synthesis of (5a*S*,5a'*S*,10b*R*,10b'*R*)-2,2'-(2-methyl-1,3-phenylene)bis(1-iodo-3-methyl-9-(2,2,2-trifluoroacetamido)-2,5a,6,10b-tetrahydro-4H-imidazo[1,5-d]indeno[2,1-b][1,4]oxazin-11-ium) triflate ($7b^{OTf}$)

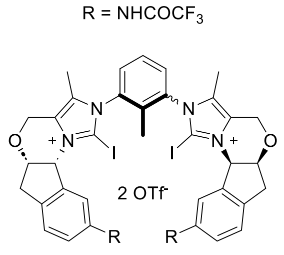

$7b^{OTf}$

Bis(iodoimidazolium) chloride salt **$7b^{Cl}$** (420 mg, 386 µmol, 1.0 eq.) was dissolved in 4.3 ml of 4:1 MeCN/MeOH (0.09 M), then NaOTf (133 mg, 773 µmol, 2.0 eq.) was added and the mixture was stirred for 14 h at room temperature (immediate precipitation was observed upon addition of NaOTf). The solvent was then removed *in vacuo* and the residue was resuspended in MeCN and solids filtered off (syringe filter, 0.22 µm). The solvent of the resulting filtrate was then removed *in vacuo*.

The obtained residue was once precipitated from a minimal amount of MeCN by addition of Et_2O (~10 volume equivalents). The precipitate was dried under high vacuum, yielding **$7b^{OTf}$** as a mixture of atropisomers which was obtained as slightly yellowish solid (482 mg, 366 µmol, 95% for both isomers).

The mixture of atropisomers was then extracted with THF to achieve partial separation: 2 ml of THF was added, the obtained suspension was subjected to sonication. Following sonication, almost all of the mixture had dissolved, then, an additional 6 ml of THF were added and the mixture was stored at 4°C overnight. After this time, a precipitate had formed which was filtered off and washed with additional THF. The solvent was removed from the filtrate and the procedure of treatment with THF and filtration was repeated to precipitate further product.

After this, two fractions had been obtained, both of which were then precipitated once again from minimal MeCN by addition of Et_2O to yield two fractions enriched in either atropisomer. The products were then coevaporated once with acetonitrile and dried for two days at 45°C under high vacuum.

The fraction soluble in THF, **$7b^{OTf}$** (fraction 1)**,** was obtained as a slightly yellowish solid (331 mg, 251 µmol, 65% based on sum of isomers). The fraction insoluble in THF, **$7b^{OTf}$** (fraction 2, “*syn*”-**$7b^{OTf}$**) was obtained as a colorless solid (97 mg, 73.8 µmol, 19% based on sum of isomers).

Although no clear crystallographic evidence could be obtained yet, the tentative assignment of isomers was made based on NMR spectroscopic properties and trends regarding catalytic activity of the two fractions; indicating fraction 1 to consist of a ~2:1 mixture in favor of the *anti* atropisomer, while fraction 2 was determined to consist of essentially pure *syn* atropisomer.

Major fraction (**$7b^{OTf}$**, fraction 1):

^{1}H NMR (400 MHz, Acetonitrile-*d*$_3$):
δ [ppm] = 9.47 – 9.18 (m, 2H), 8.01 – 7.53 (m, 6H), 7.53 – 7.37 (m, 3H), 5.89 – 5.69 (m, 2H), 5.03 – 4.90 (m, 2H), 4.90 – 4.72 (m, 4H), 3.42 (dt, *J* = 16.9, 3.7 Hz, 2H), 3.16 (dt, *J* = 17.0, 2.4 Hz, 2H), 2.17 – 1.87 (m, 9H).

^{1}H NMR (300 MHz, DMSO-*d*$_6$):
δ [ppm] = 11.43 – 11.26 (m, 2H), 8.19 – 7.85 (m, 5H), 7.69 – 7.40 (m, 4H), 6.11 – 5.88 (m, 2H), 5.18 – 4.99 (m, 2H), 4.93 – 4.73 (m, 4H), 3.44 (d, *J* = 17.4 Hz, 2H), 3.10 (dd, *J* = 16.7, 3.8 Hz, 2H), 2.18 – 2.03 (m, 6H), 1.96 – 1.83 (m, 3H).

^{13}C NMR (101 MHz, Acetonitrile-*d*$_3$):
δ [ppm] = 156.1 (q, *J* = 37.4 Hz), 155.9 (q, *J* = 37.4 Hz), 139.8, 139.8, 139.7, 138.7, 138.6, 138.5, 138.4, 136.9, 136.8, 136.6, 136.6, 136.5, 136.5, 136.2, 136.1, 135.8, 135.8, 135.5, 133.6, 133.4, 133.3, 133.2, 131.4, 131.2, 131.1, 130.4, 130.3, 130.3, 130.2, 129.6, 129.3, 129.0, 128.8, 127.5, 127.3, 123.3, 123.2, 123.0, 122.8, 121.9 (q, *J* = 320.4 Hz) 121.2, 119.7, 119.5, 115.5, 115.4, 115.3, 112.7, 112.5, 101.1, 100.6, 98.8, 98.2, 79.5, 79.5, 64.0, 63.9, 63.8, 63.7, 61.0, 60.9, 37.9, 37.9, 14.0, 13.9, 13.6, 10.2, 10.1, 10.0.

^{19}F NMR[7] (376 MHz, Acetonitrile-d_3):
δ [ppm] = -75.96* (s, 3F), -76.10* (s, 3F), -76.13 (s, 3F), -76.21 (s, 3F), -79.31 (s, 6F).

ATR-IR:
$\tilde{\nu}$ [cm^{-1}] = 3275 (w), 3136 (w), 3094 (w), 2932 (w), 1717 (m), 1684 (m), 1653 (m), 1647 (w), 1636 (w), 1616 (w), 1609 (w), 1558 (m), 1541 (m), 1522 (w), 1506 (w), 1489 (m), 1481 (m), 1456 (w), 1437 (m), 1279 (m), 1242 (s), 1223 (s), 1211 (s), 1150 (vs), 1103 (m), 1084 (m), 1026 (vs), 899 (w), 820 (w), 787 (w), 756 (w), 725 (m), 635 (s), 573 (m), 515 (m), 492 (w).

HRMS (MALDI-TOF):

m/*z* (+) = calc. 1015.0395 [M-2OTf-H]$^+$; found 1015.0350 [M-2OTf-H]$^+$.

Minor fraction (**7b^{OTf}**, fraction 2 or "*syn*"-**7b^{OTf}**):

^{1}H NMR (400 MHz, Acetonitrile-d_3):
δ [ppm] = 9.47 – 9.26 (m, 2H), 7.89 (t, *J* = 8.0 Hz, 1H), 7.84 – 7.75 (m, 2H), 7.74 (s, 1H), 7.71 – 7.63 (m, 2H), 7.54 (d, *J* = 8.2 Hz, 1H), 7.44 (d, *J* = 8.2 Hz, 2H), 5.87 – 5.68 (m, 2H), 5.05 – 4.69 (m, 6H), 3.40 (dd, *J* = 17.1, 3.9 Hz, 2H), 3.14 (dd, *J* = 17.1, 2.6 Hz, 2H), 2.20 – 2.14 (m, 9H).

^{1}H NMR (400 MHz, DMSO-d_6):
δ [ppm] = 11.45 – 11.28 (m, 2H), 8.10 – 7.84 (m, 5H), 7.69 (d, *J* = 8.0 Hz, 1H), 7.57 (d, *J* = 8.0 Hz, 1H), 7.52 – 7.40 (m, 2H), 5.99 (dd, *J* = 29.4, 3.2 Hz, 2H), 5.14 – 4.94 (m, 2H), 4.94 – 4.74 (m, 4H), 3.44 (dd, *J* = 17.0, 3.4 Hz, 2H), 3.08 (dd, *J* = 16.6, 4.5 Hz, 2H), 2.22 – 2.05 (m, 6H), 2.05 – 1.92 (m, 3H).

^{13}C NMR (101 MHz, Acetonitrile-d_3):
δ [ppm] = 154.5 (q, *J* = 36.9 Hz), 154.4 (q, *J* = 37.0 Hz), 138.4, 138.4, 138.1, 137.9, 135.6, 135.5, 135.4, 135.1, 134.8, 132.1, 129.8, 128.1, 127.9, 126.5, 126.5, 126.3, 126.1, 121.6, 121.6, 120.7 (q, *J* = 322.3 Hz), 120.1, 120.0, 118.0, 117.3, 117.2, 116.6, 114.4, 114.3, 111.5, 111.4, 107.5, 105.7, 78.3, 62.2, 62.1, 59.6, 59.4, 36.8, 36.8, 13.0, 9.2, 9.1.

^{19}F NMR (376 MHz, Acetonitrile-d_3):
δ [ppm] = - 75.92 (s, 3F), -76.08 (s, 3F), -79.34 (s, 6F).

ATR-IR:
$\tilde{\nu}$ [cm^{-1}] = 3269 (w), 3142 (w), 3044 (w), 2928 (w), 1717 (m), 1701 (m), 1684 (m), 1653 (m), 1647 (w), 1636 (w), 1616 (w), 1609 (w), 1558 (m), 1541 (m), 1533 (w), 1522 (w), 1506 (w), 1489 (m), 1481 (m), 1456 (w), 1437 (m), 1420 (w), 1373 (w), 1279 (m), 1244 (m), 1209 (m), 1148 (s), 1107 (s), 1084 (m), 1070 (m), 1028 (s), 899 (w), 864 (w), 843 (w), 816 (w), 787 (w), 746 (w), 725 (w), 637 (s), 573 (w), 517 (m).

HRMS (MALDI-TOF):
m/*z* (+) = calc. 1015.0395 [M-2OTf-H]$^+$; found 1015.0317 [M-2OTf-H]$^+$.

---

[7] Here, two sets of signals (two rotamers each) of the two atropisomers are visible, the signals of the minor atropisomer (tentatively *syn* atropisomer) are marked with a star.

Synthesis of (5a*S*,5a'*S*,10b*R*,10b'*R*)-2,2'-(2-methyl-1,3-phenylene)bis(1-iodo-3-methyl-9-(2,2,2-trifluoroacetamido)-2,5a,6,10b-tetrahydro-4H-imidazo[1,5-d]indeno[2,1-b][1,4]oxazin-11-ium) tetrakis(3,5-bis(trifluoromethyl)phenyl)borate ($7b^{BArF}$, fraction 1)

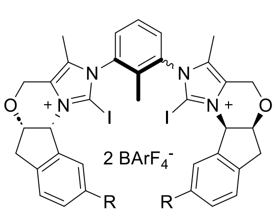

$7b^{BArF}$

Bis(iodoimidazolium) triflate salt **$7b^{OTf}$** (fraction 1) (312 mg, 237 µmol, 1.0 eq.) and $NaBAr^F_4$ (421 mg, 475 µmol, 2.0 eq.) were suspended in 2.4 ml dry acetone (0.1 M) and the mixture was stirred for 2.5 h at 42°C. The mixture was cooled to room temperature, the solvent was removed *in vacuo* and the residue dried under high vacuum. The residue was then extracted with Et_2O and the solvent of the filtrate removed *in vacuo*. The obtained residue was afterwards extracted with $CHCl_3$/DCM (1:1). The solvent of the filtrate thus obtained was removed *in vacuo* and the gained residue was precipitated with *n*-pentane from DCM. The product was then co-evaporated with DCM and dried under high vacuum at 45°C to remove residual solvent, yielding the product **$7b^{BArF}$** (fraction 1) as a beige solid (636 mg, 232 µmol, 98%).

^{1}H NMR (400 MHz, DCM-*d_2*):
δ [ppm] = 8.51 – 8.26 (m, 2H), 8.17 – 7.79 (m, 4H), 7.80 – 7.70 (m, 17H), 7.58 (s, 8H), 7.56 – 7.43 (m, 2H), 7.24 (d, 2H), 7.09 (d, *J* = 8.1 Hz, 2H), 5.78 – 5.66 (m, 2H), 5.01 – 4.79 (m, 6H), 3.56 – 3.44 (m, 2H), 3.34 (dd, *J* = 17.0, 5.2 Hz, 2H), 2.31 – 2.14 (m, 6H), 2.00 – 1.84 (m, 3H).

^{13}C{^{1}H,^{19}F} NMR (101 MHz, DCM-*d_2*):
δ [ppm] = 162.4 (dd, *J* = 49.8 Hz), 155.7, 155.5, 155.4, 139.1, 139.1, 139.0, 138.9, 136.9, 136.6, 136.5, 136.0, 135.9, 135.9, 135.8, 135.7, 135.7, 135.5, 135.2, 135.2, 134.7, 133.5, 133.4, 133.2, 133.1, 132.9, 132.3, 131.8, 131.1, 130.8, 130.7, 130.6, 130.5, 129.5 (q, *J* = 2.8 Hz), 127.9, 127.8, 125.2, 122.5, 122.5, 121.7, 121.7, 118.2, 117.5, 117.2, 116.2, 116.1, 94.1, 94.1, 93.8, 93.4, 79.0, 78.9, 78.8, 78.8, 64.2, 63.9, 60.1, 60.0, 37.9, 37.8, 13.9, 13.3, 10.5, 10.3, 10.3, 10.3.

^{19}F NMR (376 MHz, DCM-*d_2*):
δ [ppm] = -62.77 (s, 48F), -76.08 – -76.53 (m, 6F).

ATR-IR:
$\tilde{\nu}$ [cm^{-1}] = 3431 (w), 3100 (w), 2936 (w), 1748 (w), 1734 (w), 1717 (w), 1705 (w), 1699 (w), 1684 (w), 1653 (w), 1647 (w), 1636 (w), 1609 (w), 1558 (w), 1549 (w), 1541 (w), 1533 (w), 1526 (w), 1522 (w), 1506 (w), 1497 (w), 1489 (w), 1481 (w), 1474 (w), 1456 (w) 1437 (w), 1418 (w), 1354 (s), 1273 (vs), 1211 (w), 1107 (vs), 1001 (w), 932 (w), 885 (m), 839 (m), 818 (w), 810 (w), 787 (w), 745 (m), 725 (w), 712 (s), 681 (s), 669 (s), 581 (w), 517 (w), 449 (w).

HRMS (MALDI-TOF):

m/*z* (+) = calc. 1015.0395 $[M\text{-}2BAr^F_4\text{-}H]^+$; found 1015.0406 $[M\text{-}2BAr^F_4\text{-}H]^+$.

calc. 1753.2156 $[M\text{-}BAr^F_4\text{-}I]^+$; found 1753.2151 $[M\text{-}BAr^F_4\text{-}I]^+$.

$[\alpha]_D^{20}$ (c: 0.51, CH_2Cl_2): -147.5°

Synthesis of (5a*S*,5a'*S*,10b*R*,10b'*R*)-2,2'-(2-methyl-1,3-phenylene)bis(1-iodo-3-methyl-9-(2,2,2-trifluoroacetamido)-2,5a,6,10b-tetrahydro-4H-imidazo[1,5-d]indeno[2,1-b][1,4]oxazin-11-ium) tetrakis(3,5-bis(trifluoromethyl)phenyl)borate ("*syn*"-7b^{BArF})

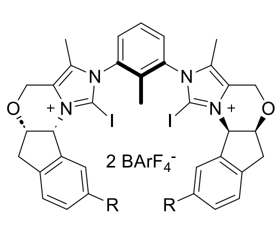

7b^{BArF}

Bis(iodoimidazolium) triflate salt "*syn*"-**7b^{OTf}** (60 mg, 45.6 µmol, 1.0 eq.) and NaBAr$^F_4$ (80.9 mg, 91.3 µmol, 2.0 eq.) were suspended in 0.46 ml dry acetone (0.1 M) and the mixture was stirred for 2.5 h at 42°C. The mixture was cooled to room temperature, the solvent was removed *in vacuo* and the residue dried under high vacuum. The residue was then extracted with Et_2O and the solvent of the filtrate removed *in vacuo*. The obtained residue was afterwards extracted with $CHCl_3$/DCM (1:1). The solvent of the filtrate thus obtained was removed *in vacuo* and the gained residue was precipitated with *n*-pentane from DCM. The product was then co-evaporated with DCM and dried under high vacuum at 45°C to remove residual solvent, yielding the product "*syn*"-**7b^{BArF}** as a colorless solid (122 mg, 44.4 µmol, 97%).

^{1}H NMR (300 MHz, DCM-*d*$_2$):
δ [ppm] = 8.37 (s, 1H), 8.28 (s, 1H), 8.08 (s, 2H), 7.95 (t, *J* = 8.0 Hz, 1H), 7.79 – 7.64 (m, 18H), 7.54 (s, 8H), 7.46 (t, *J* = 8.2 Hz, 2H), 7.21 (dd, *J* = 8.1, 1.6 Hz, 1H), 7.06 (dd, *J* = 8.0, 1.8 Hz, 1H), 5.69 (dd, *J* = 10.9, 3.6 Hz, 2H), 4.95 – 4.73 (m, 6H), 3.45 (dd, *J* = 17.4, 4.1 Hz, 2H), 3.29 (dd, *J* = 17.0, 2.0 Hz, 2H), 2.19 – 2.05 (m, 6H), 1.96 (s, 3H).

^{13}C{^{1}H,^{19}F} NMR (101 MHz, DCM-*d*$_2$):
δ [ppm] = 162.4 (dd, *J* = 49.8 Hz), 155.7, 155.5, 139.2, 139.0, 136.9, 136.7, 136.0, 135.9, 135.6, 135.4, 135.3, 135.3, 133.2, 133.0, 132.2, 130.5, 130.3, 129.5 (q, *J* = 2.8 Hz), 127.8, 127.7, 125.2, 122.6, 121.8, 118.1, 117.6, 116.2, 116.2, 116.0, 95.5, 78.9, 78.8, 64.1, 63.8, 60.1, 60.1, 37.9, 37.8, 14.0, 10.3, 10.2.

^{19}F NMR (376 MHz, DCM-*d*$_2$):
δ [ppm] = -62.74 (s, 48F), -76.28 (s, 3F), -76.35 (s, 3F).

ATR-IR:
$\tilde{\nu}$ [cm^{-1}] = 3431 (w), 2970 (w), 2934 (w), 2322 (w), 1732 (w), 1719 (w), 1609 (w) 1558 (w), 1541 (w), 1481 (w), 1456 (w), 1439 (w), 1354 (s), 1273 (vs), 1107 (vs), 932 (w), 885 (m), 839 (m), 816 (w), 766 (w), 745 (w), 725 (w), 712 (s), 681 (s), 669 (s), 611 (w), 581 (w), 515 (w), 486 (w), 449 (w).

HRMS (MALDI-TOF):

m/*z* (+) = calc. 1753.2156 [M-BAr$^F_4$-I]$^+$; found 1753.2160 [M-BAr$^F_4$-I]$^+$.

[α]$_D^{20}$ (c: 0.45, CH_2Cl_2): -173.0°

Synthesis of (5a*S*,5a'*S*,10b*R*,10b'*R*)-2,2'-(2-methyl-1,3-phenylene)bis(9-amino-3-methyl-2,5a,6,10b-tetrahydro-4*H*-imidazo[1,5-d]indeno[2,1-b][1,4]oxazin-11-ium) chloride (6^{Cl})

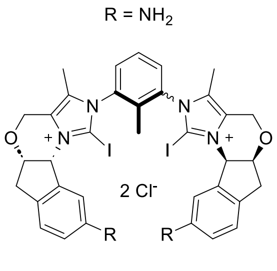

6^{Cl}

Bis(imidazolium) chloride salt $\mathbf{5b^{Cl}}$ (348 mg, 417 µmol,1.0 eq.) was dissolved in 4.2 ml MeOH/H_2O (1:1, 0.1 M) and then K_2CO_3 (115 mg, 833 µmol, 2.0 eq.) was added. The mixture was stirred at room temperature overnight (14 hours). Then, excess MeOH was added (20 ml) and the mixture was filtered. The solvent was removed *in vacuo* (to remove residual water, the mixture was co-evaporated three times with absolute EtOH) and the crude product was then purified by column chromatography (SiO_2, DCM/MeOH 8:1 → 4:1 → 1:1). The product thus received was dissolved in MeOH and stirred in the presence of 2 g Amberlite IRA 958 Cl for a few hours. The resin was then filtered off and rinsed with MeOH. The solvent of the filtrate was removed *in vacuo* and the described procedure was repeated 3 times, after which the anion exchange was found to be complete. The product $\mathbf{6^{Cl}}$ was thus obtained as a slightly beige solid (229 mg, 355 µmol, 85%).

^{1}H NMR (400 MHz, DMSO-*d*$_6$):
δ [ppm] = 10.60 – 10.05 (m, 2H), 8.17 – 7.74 (m, 3H), 7.09 – 6.96 (m, 2H), 6.83 – 6.62 (m, 2H), 6.62 – 6.47 (m, 2H), 5.94 – 5.75 (m, 2H), 5.25 – 4.93 (m, 6H), 4.88 – 4.75 (m, 4H), 3.22 (dd, *J* = 16.9, 4.5 Hz, 2H), 2.94 (dd, *J* = 16.5, 7.2 Hz, 2H), 2.18 – 1.96 (m, 9H).

^{13}C NMR (101 MHz, Methanol-*d*$_4$):
δ [ppm] = 149.1, 149.0, 148.8, 138.6, 138.5, 136.5, 135.7, 135.2, 135.1, 132.6, 132.5, 132.4, 132.2, 130.3, 130.2, 130.1, 130.0, 127.1, 126.9, 126.7, 126.6, 125.7, 125.5, 125.4, 117.7, 117.7, 117.5, 111.2, 110.6, 110.4, 79.0, 78.9, 62.1, 60.2, 37.7, 37.6, 37.4, 13.6, 13.3, 8.6, 8.4, 8.3, 8.2.

ATR-IR:
$\tilde{\nu}$ [cm^{-1}] = 3366 (m), 3227 (w), 3107 (w), 2955 (w), 2920 (w), 2849 (w), 2367 (w), 2322 (w), 1647 (m), 1636 (s), 1622 (s), 1616 (s), 1541 (s), 1497 (vs), 1481 (m), 1474 (m), 1456 (m), 1449 (m), 1437 (m), 1375 (w), 1331 (m), 1288 (w), 1250 (w), 1231 (w), 1202 (w), 1186 (m), 1167 (m), 1098 (s), 1080 (s), 1067 (s), 1034 (m), 991 (w), 849 (w), 812 (vs), 762 (w), 719 (w), 615 (w), 527 (w), 473 (w), 432 (w).

HRMS (MALDI-TOF):

m/*z* (+) = calc. 571.2816 [M-2Cl-H]$^+$; found 571.2805 [M-2Cl-H]$^+$.

Synthesis of (5a*S*,5a'*S*,10b*R*,10b'*R*)-2,2'-(2-methyl-1,3-phenylene)bis(9-(2,2-difluoroacetamido)-3-methyl-2,5a,6,10b-tetrahydro-4*H*-imidazo[1,5-d]indeno[2,1-b][1,4]oxazin-11-ium) chloride ($5c^{Cl}$)

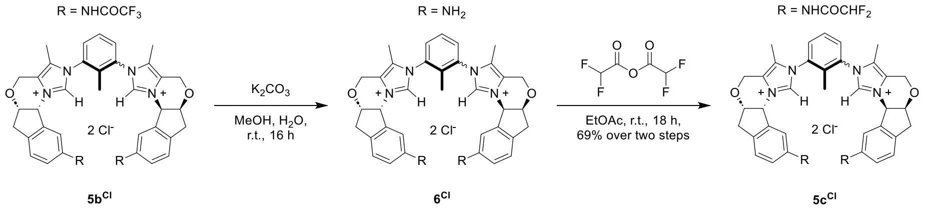

Bis(imidazolium) chloride salt $\mathbf{5b^{Cl}}$ (250 mg, 299 µmol,1.0 eq.) was dissolved in 3.0 ml MeOH/H_2O (1:1, 0.1 M) and then K_2CO_3 (82.7 mg, 598 µmol, 2.0 eq.) was added. The mixture was stirred at room temperature overnight (16 hours). Then, excess MeOH was added (20 ml) and the mixture was filtered. The solvent was then removed *in vacuo* (to remove residual water, the mixture was co-evaporated three times with absolute EtOH). Full conversion

to the aniline intermediate was confirmed *via* ^{1}H NMR and the product mixture subsequently used as received in the next step.

The crude product mixture containing **6^{Cl}** was then suspended in EtOAc (1.2 ml, 0.25 M), then difluoroacetic anhydride (520 mg, 325 µl, 2.99 mmol, 10 eq.) was slowly added and the mixture was stirred at room temperature for 18 h. Then, Et_2O (30 ml) was added and the formed precipitate separated by filtration and afterwards washed with excess Et_2O. The obtained solid was dried under high vacuum and then purified by column chromatography (SiO_2, DCM/MeOH 8:1 → 4:1 → 1:1). The obtained solid was then dissolved in minimal MeOH and stirred with Amberlite IRA 958 Cl (1 g) for a few hours at room temperature. The resin was filtered off, rinsed with further MeOH and the solvent of the filtrate was subsequently removed *in vacuo*. This procedure was repeated two further times until the anion exchange was complete, yielding product **5c^{Cl}** as a light beige solid (164 mg, 205 µmol, 69% over two steps).

^{1}H NMR (400 MHz, DMSO-*d*$_6$):
δ [ppm] = 11.50 – 11.10 (m, 2H), 10.31 (s, 2H), 8.16 – 7.77 (m, 5H), 7.72 – 7.47 (m, 2H), 7.47 – 7.35 (m, 2H), 6.71 – 6.19 (m, 2H), 6.07 – 5.93 (m, 2H), 5.19 – 4.99 (m, 2H), 4.99 – 4.77 (m, 4H), 3.42 (d, *J* = 16.5 Hz, 2H), 3.11 (d, *J* = 16.7 Hz, 2H), 2.21 – 1.91 (m, 9H).

^{13}C{^{1}H,^{19}F} NMR (101 MHz, Methanol-*d*$_4$):
δ [ppm] = 163.0, 162.9, 162.8, 138.9, 138.7, 138.6, 137.9, 137.5, 136.9, 136.9, 136.8, 136.5, 136.3, 136.2, 135.5, 135.3, 135.2, 132.7, 132.6, 132.4, 132.4, 130.3, 130.2, 127.5, 127.2, 127.1, 125.5, 125.2, 123.2, 122.9, 122.7, 117.5, 116.7, 116.5, 109.9, 109.8, 109.7, 79.0, 79.0, 62.1, 60.5, 38.2, 38.1, 30.7, 13.5, 13.4, 8.4.

^{19}F NMR (376 MHz, DCM-*d*$_2$):
δ [ppm] = -124.71 – -125.33 (m, 4F).

ATR-IR:
$\tilde{\nu}$ [cm^{-1}] = 3393 (w), 3048 (w), 2990 (w), 2930 (w), 2857 (w), 2297 (w), 1697 (m), 1616 (w), 1557 (w), 1541 (m), 1491 (m), 1437 (w), 1373 (w), 1329 (w), 1306 (w), 1248 (w), 1186 (w), 1152 (w), 1099 (m), 1082 (m), 1057 (s), 974 (w), 918 (w), 860 (w), 810 (m), 762 (w), 750 (w), 721 (w), 656 (w), 617 (w), 583 (w), 519 (w), 473 (w).

HRMS (MALDI-TOF):

m/*z* (+) = calc. 726.2656 $[M\text{-}2Cl\text{-}H]^+$; found 726.2656 $[M\text{-}2Cl\text{-}H]^+$.

Synthesis of (5a*S*,5a'*S*,10b*R*,10b'*R*)-2,2'-(2-methyl-1,3-phenylene)bis(9-(2,2-difluoroacetamido)-1-iodo-3-methyl-2,5a,6,10b-tetrahydro-4H-imidazo[1,5-d]indeno[2,1-b][1,4]oxazin-11-ium) trifluoromethanesulfonate ($7c^{OTf}$)

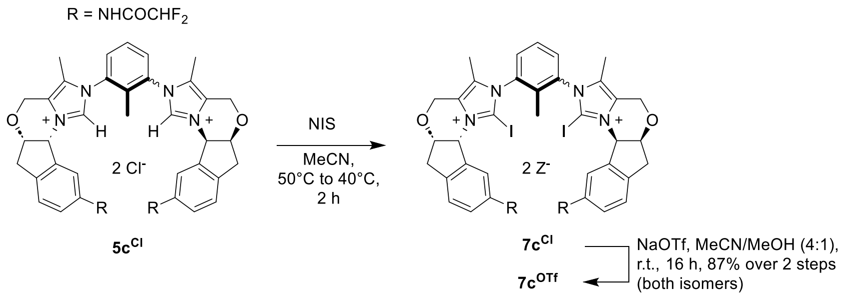

Bis(imidazolium) chloride salt **$5c^{Cl}$** (150 mg, 188 µmol, 1.0 eq.) was suspended in 3.8 ml dry MeCN (0.05 M), then *N*-iodosuccinimide (92.9 mg, 413 µmol, 2.2 eq.) was added and the mixture was then heated to 50°C, after 15 minutes, it was cooled down to 40°C and stirred at this temperature for 2 hours. The solvent was then removed *in vacuo* and the obtained mixture was precipitated three times from MeCN/MeOH (4:1) by addition of Et_2O (3-4 volume equivalents) and the obtained yellowish solid (crude **$7c^{Cl}$**, 184 mg, 175 µmol, 93%) was finally dried under high vacuum. The crude product mixture still contained residual *N*-iodosuccinimide and solvent and was used in the next step without further purification.

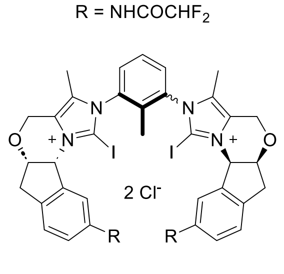

$7c^{Cl}$

Bis(iodoimidazolium) chloride salt **$7c^{Cl}$** (184 mg, 175 µmol, 1.0 eq.) was dissolved in 1.9 ml 4:1 MeCN/MeOH (0.09 M), then NaOTf (57.3 mg, 333 µmol, 2.0 eq.) was added and the mixture was stirred for 16 h at room temperature (immediate precipitation was observed upon addition of NaOTf). The solvent was then removed *in vacuo* and the residue resuspended in MeCN and filtered (syringe filter, 0.22 µm). The solvent of the resulting filtrate was then removed *in vacuo*. The mixture was once precipitated from a minimal amount of MeCN by addition of Et_2O (~10 volume equivalents). The precipitate was dried under high vacuum to yield the mixture of iodinated atropisomers **$7c^{OTf}$** (200 mg, 156 µmol, 94%) as a yellowish solid.

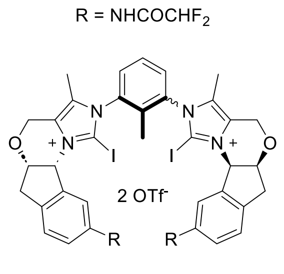

$7c^{OTf}$

The mixture of atropisomers was then extracted with THF to achieve partial separation: 2 ml of THF was added, then obtained suspension was subjected to sonication. Following sonication, almost all of the mixture had dissolved, then, an additional 3 ml of THF were added and the mixture was stored at 4°C overnight. After this time, a precipitate had formed which was filtered off and washed with additional THF. The solvent was removed from the filtrate and the procedure of treatment with THF and filtration was repeated to check for precipitation of further product from the filtrate, which did not occur.

After this, two fractions had been obtained, both of which were then precipitated once again from minimal MeCN by addition of Et_2O to yield two fractions enriched in either atropisomer. The products were then coevaporated once with acetonitrile and dried for two days at 45°C under high vacuum.

The fraction soluble in THF, **$7c^{OTf}$** (fraction 1)**,** was obtained as a slightly yellowish solid (118 mg, 92.0 µmol, 55% based on sum of isomers). The fraction insoluble in THF, **$7c^{OTf}$** (fraction 2) was obtained as a slightly yellowish solid (66.3 mg, 51.9 µmol, 31% based on sum of isomers).

Major fraction (**$7c^{OTf}$**, fraction 1):

^{1}H NMR (400 MHz, Acetonitrile-*d_3*):
δ [ppm] = 9.15 – 8.86 (m, 2H), 8.04 – 7.71 (m, 5H), 7.65 – 7.52 (m, 1H), 7.50 – 7.36 (m, 3H), 6.30 – 5.84 (m, 2H), 5.82 – 5.71 (m, 2H), 5.05 – 4.74 (m, 6H), 3.49 – 3.34 (m, 2H), 3.15 (dd, *J* = 16.8, 3.2 Hz, 2H), 2.18 – 1.87 (m, 9H).

^{13}C{^{1}H,^{19}F} NMR (101 MHz, Acetonitrile-*d_3*):
δ [ppm] = 161.9, 161.8, 161.8, 139.0, 138.9, 138.8, 138.6, 138.5, 138.3, 137.0, 137.0, 136.9, 136.7, 136.7, 136.7, 136.6, 136.6, 136.5, 136.4, 135.6, 133.6, 133.4, 133.3, 133.2, 131.3, 131.2, 131.1, 130.4, 130.3, 130.2, 130.1, 129.5, 129.3, 129.0, 128.9, 127.4, 127.2, 122.6, 122.6, 122.4, 122.3, 119.0, 118.9, 117.7, 109.6, 109.5, 109.4, 101.0, 100.5, 98.8, 98.1, 79.5, 79.5, 64.0, 63.9, 63.8, 63.8, 60.9, 60.9, 37.9, 37.9, 14.1, 13.8, 13.6, 10.2, 10.1, 10.0.

^{19}F NMR (376 MHz, Acetonitrile-*d_3*):
δ [ppm] = -79.30 (s, 6F), -126.89 – -127.33 (m, 4F).

ATR-IR:
$\tilde{\nu}$ [cm^{-1}] = 3289 (w), 3213 (w), 3096 (w), 2930 (w), 2853 (w), 2372 (w), 2313 (w), 1697 (m), 1616 (w), 1609 (w), 1558 (w), 1541 (w), 1489 (w), 1481 (w), 1456 (w), 1437 (w), 1373 (w), 1339 (w), 1273 (s), 1242 (s), 1225 (s), 1159 (m), 1103 (m), 1082 (m), 1067 (m), 1026 (vs), 993 (w), 891 (w), 866 (w), 816 (m), 760 (w), 723 (w), 637 (s), 573 (w), 515 (m).

HRMS (MALDI-TOF):

m/*z* (+) = calc. 1129.0182 [M-OTf]$^+$; found 1129.0184 [M-OTf]$^+$.

calc. 979.0589 [M-2OTf-H]$^+$; found 979.0574 [M-2OTf-H]$^+$.

Minor fraction (**$7c^{OTf}$**, fraction 2):

^{1}H NMR (400 MHz, Acetonitrile-*d_3*):
δ [ppm] = 9.15 – 8.94 (m, 2H), 7.99 – 7.83 (m, 2H), 7.83 – 7.74 (m, 2H), 7.67 (d, J = 7.9 Hz, 2H), 7.60 – 7.46 (m, 1H), 7.46 – 7.36 (m, 2H), 6.28 – 5.95 (m, 2H), 5.82 – 5.72 (m, 2H), 5.01 – 4.74 (m, 6H), 3.39 (dd, J = 17.2, 3.9 Hz, 2H), 3.14 (dd, J = 16.9, 3.0 Hz, 2H), 2.18 – 2.14 (m, 8H), 1.97 – 1.96 (m, 2H).

^{19}F NMR (376 MHz, Acetonitrile-*d_3*):
δ [ppm] = -79.33 (s, 6F), -126.86 – -127.35 (m, 4F).

ATR-IR:
$\tilde{\nu}$ [cm^{-1}] = 3281 (w), 3213 (w), 3075 (w), 2926 (w), 2849 (w), 2367 (w), 2315 (w), 1701 (m), 1616 (w), 1609 (w), 1558 (w), 1551 (w), 1541 (w), 1489 (w), 1481 (w), 1437 (w), 1371 (w), 1339 (w), 1273 (s), 1246 (s), 1225 (s), 1155 (m), 1105 (s), 1082 (m), 1063 (m), 1028 (vs), 891 (w), 866 (w), 814 (w), 637 (v), 573 (w), 517 (w).

HRMS (MALDI-TOF):
m/*z* (+) = calc. 853.1617 [M-2OTf-I-H]$^+$; found 853.1615 [M-2OTf-I-H]$^+$.

Synthesis of (5a*S*,5a'*S*,10b*R*,10b'*R*)-2,2'-(2-methyl-1,3-phenylene)bis(9-(2,2-difluoroacetamido)-1-iodo-3-methyl-2,5a,6,10b-tetrahydro-4H-imidazo[1,5-d]indeno[2,1-b][1,4]oxazin-11-ium) tetrakis(3,5-bis(trifluoromethyl)phenyl)borate ($7c^{BArF}$, fraction 1)

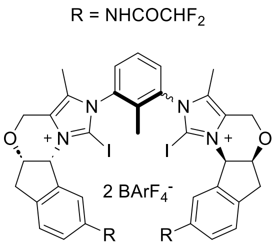

$7c^{BArF}$

Bis(iodoimidazolium) triflate salt $\mathbf{7c^{OTf}}$ (fraction 1) (101 mg, 79.0 µmol, 1.0 eq.) and $NaBAr^F{}_4$ (140 mg, 158 µmol, 2.0 eq.) were suspended in 0.79 ml dry acetone (0.1 M) and the mixture was stirred for 2.5 h at 42°C. The mixture was cooled to room temperature, the solvent was removed *in vacuo* and the residue dried under high vacuum. The residue was then extracted with Et_2O and the solvent of the filtrate removed *in vacuo*. The obtained residue was afterwards extracted with $CHCl_3$/DCM (1:1). The solvent of the filtrate thus obtained was removed *in vacuo* and the gained residue was precipitated with *n*-pentane from DCM. The product was then co-evaporated with DCM and dried under high vacuum at 45°C to remove residual solvent, yielding the product $\mathbf{7c^{BArF}}$ (fraction 1) as a slightly yellowish solid (184 mg, 68.1 µmol, 86%).

^{1}H NMR (400 MHz, DCM-*d*$_2$):
δ [ppm] = 8.45 – 8.27 (m, 2H), 8.13 – 7.79 (m, 3H), 7.72 – 7.68 (m, 17H), 7.56 – 7.53 (m, 9H), 7.48 – 7.38 (m, 2H), 7.21 (dd, *J* = 8.2, 1.2 Hz, 1H), 7.03 (dd, *J* = 8.2, 1.2 Hz, 1H), 6.18 – 5.74 (m, 2H), 5.74 – 5.62 (m, 2H), 5.00 – 4.73 (m, 6H), 3.51 – 3.38 (m, 2H), 3.36 – 3.22 (m, 2H), 2.26 – 1.94 (m, 9H).

^{13}C{^{1}H,^{19}F} NMR (101 MHz, DCM-*d*$_2$):
δ [ppm] = 162.4 (dd, *J* = 49.8 Hz), 161.6, 161.3, 161.2, 138.9, 138.8, 138.4, 138.3, 136.8, 136.7, 136.6, 136.4, 136.3, 136.3, 136.0, 136.0, 135.9, 135.9, 135.9, 135.7, 135.5, 135.4, 135.2, 134.8, 133.5, 133.2, 133.2, 133.1, 132.7, 132.2, 131.9, 131.1, 130.9, 130.8, 130.7, 130.6, 130.5, 130.5, 130.2 – 128.9 (m), 127.7, 127.7, 127.0, 126.8, 126.6, 126.5, 124.2, 124.0, 124.0, 123.8, 123.6, 122.6, 121.7, 121.3, 118.2, 117.6, 117.2, 116.3, 116.21, 108.9, 108.9, 108.7, 94.8, 94.5, 93.8, 93.4, 79.1, 79.0, 78.8, 78.8, 64.3, 64.2, 64.0, 64.0, 60.2, 60.2, 60.0, 59.9, 37.9, 37.8, 31.5, 14.0, 13.9, 13.4, 10.4, 10.3, 10.2.

^{19}F NMR (376 MHz, DCM-*d*$_2$):
δ [ppm] = -62.79 (s, 48F), -126.50 – -127.09 (m, 4F).

ATR-IR:
$\tilde{\nu}$ [cm^{-1}] = 3428 (w), 3096 (w), 2926 (w), 2369 (w), 2315 (w), 1715 (w), 1609 (w), 1549 (w), 1541 (w), 1479 (w), 1437 (w), 1354 (s), 1273 (vs), 1109 (vs), 932 (w), 885 (m), 839 (m), 812 (w), 745 (w), 712 (s), 681 (s), 669 (s), 617 (w), 581 (w), 503 (w), 449 (w).

HRMS (MALDI-TOF):

m/*z* (+) = calc. 1717.2344 $[M\text{-}BAr^F{}_4\text{-}I]^+$; found 1717.2350 $[M\text{-}BAr^F{}_4\text{-}I]^+$.

$[\alpha]_D^{20}$ (c: 0.54, CH_2Cl_2): -128.7°

Synthesis of (5a*S*,5a'*S*,10b*R*,10b'*R*)-2,2'-(2-methyl-1,3-phenylene)bis(9-(2,2-difluoroacetamido)-1-iodo-3-methyl-2,5a,6,10b-tetrahydro-4H-imidazo[1,5-d]indeno[2,1-b][1,4]oxazin-11-ium) tetrakis(3,5-bis(trifluoromethyl)phenyl)borate (7c^{BArF}, fraction 2)

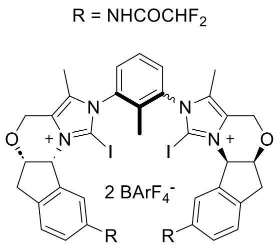

7c^{BArF}

Bis(iodoimidazolium) triflate salt **7c**^{OTf} (fraction 2) (58.5 mg, 45.8 µmol, 1.0 eq.) and $NaBAr^{F}_4$ (81.1 mg, 91.5 µmol, 2.0 eq.) were suspended in 0.46 ml dry acetone (0.1 M) and the mixture was stirred for 2.5 h at 42°C. The mixture was cooled to room temperature, the solvent was removed *in vacuo* and the residue dried under high vacuum. The residue was then extracted with Et_2O and the solvent of the filtrate removed *in vacuo*. The obtained residue was afterwards extracted with $CHCl_3$/DCM (1:1). The solvent of the filtrate thus obtained was removed *in vacuo* and the gained residue was precipitated with *n*-pentane from DCM. The product was then co-evaporated with DCM and dried under high vacuum at 45°C to remove residual solvent, yielding the product **7c**^{BArF} (fraction 2) as a slightly yellowish solid (117 mg, 43.3 µmol, 95%).

^{1}H NMR (400 MHz, DCM-*d*$_2$):
δ [ppm] = 8.49 – 8.28 (m, 2H), 8.18 – 8.02 (m, 2H), 7.99 – 7.84 (m, 1H), 7.82 – 7.71 (m, 18H), 7.59 (s, 8H), 7.48 – 7.36 (m, 2H), 7.24 (d, *J* = 8.1 Hz, 1H), 7.04 (d, *J* = 8.1 Hz, 1H), 6.18 – 5.78 (m, 2H), 5.78 – 5.66 (m, 2H), 4.97 – 4.74 (m, 6H), 3.45 (dd, *J* = 17.1, 3.9 Hz, 2H), 3.30 (dd, *J* = 17.5, 4.2 Hz, 2H), 2.35 – 2.13 (m, 6H), 2.08 – 1.94 (m, 3H).

^{13}C{^{1}H,^{19}F} NMR (101 MHz, DCM-*d*$_2$):
δ [ppm] = 162.3 (dd, *J* = 49.8 Hz), 161.3, 161.2, 161.1, 138.4, 138.3, 138.3, 138.1, 136.7, 136.5, 136.4, 136.3, 136.3, 136.1, 136.0, 136.0, 135.9, 135.4, 135.3, 133.5, 133.3, 133.1, 133.1, 132.2, 131.8, 131.0, 130.8, 130.8, 130.6, 130.5, 130.4, 129.8 – 129.2 (m), 127.7, 127.6, 126.5 , 126.5, 124.1, 124.0, 123.9, 123.7, 123.5, 122.3, 121.6, 121.3, 117.4, 117.1, 116.1, 108.9, 108.7, 94.9, 94.6, 93.9, 93.5, 79.0, 79.0, 78.8, 64.2, 63.9, 63.9, 60.2, 60.1, 60.0, 59.9, 37.9, 37.8, 14.0, 13.9, 13.3, 10.4, 10.3, 10.3, 10.2.

^{19}F NMR (376 MHz, DCM-*d*$_2$):
δ [ppm] = -62.71 (s, 48F), -126.28 – -127.45 (m, 4F).

ATR-IR:
$\tilde{\nu}$ [cm^{-1}] = 3428 (w), 3069 (w), 2920 (w), 2315 (w), 2207 (w), 1717 (w), 1609 (w), 1541 (w), 1495 (w), 1479 (w), 1439 (w), 1352 (s), 1271 (vs), 1109 (vs), 932 (w), 885 (m), 839 (m), 812 (w), 745 (w), 712 (s), 681 (s), 669 (s), 581 (w), 503 (w), 484 (w), 449 (w).

HRMS (MALDI-TOF):

m/*z* (+) = calc. 1717.2344 [M-BAr^{F}_4-I]$^{+}$; found 1717.2339 [M-BAr^{F}_4-I]$^{+}$.

$[\alpha]_D^{20}$ (c: 0.53, CH_2Cl_2): -172.9°

Synthesis of (5a*S*,5a'*S*,10b*R*,10b'*R*)-2,2'-(2-methyl-1,3-phenylene)bis(3-methyl-9-(2,2,2-trichloroacetamido)-2,5a,6,10b-tetrahydro-4*H*-imidazo[1,5-d]indeno[2,1-b][1,4]oxazin-11-ium) chloride ($5d^{Cl}$)

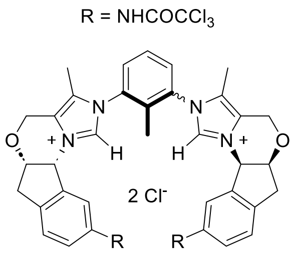

$5d^{Cl}$

Bis(imidazolium) aniline chloride salt **6^{Cl}** (190 mg, 295 µmol, 1.0 eq.) was suspended in EtOAc (1.2 ml, 0.25 M) and the suspension was cooled to 0°C. Then, trichloroacetic anhydride (911 mg, 539 µl, 2.95 mmol, 10 eq.) was slowly added dropwise and the mixture was stirred at 0°C for 15 minutes. Afterwards, it was warmed up to room temperature and stirred for a further 14 h. Then, Et_2O (30 ml) was added and the formed precipitate separated by filtration and afterwards washed with excess Et_2O. The obtained solid was dried under high vacuum and then purified by column chromatography (SiO_2, DCM/MeOH 8:1 → 4:1 → 1:1). The obtained solid was then stirred with Amberlite IRA 958 Cl (2 g) in a minimal amount of MeOH for 8 h, the resin was filtered off and the solvent removed *in vacuo*, this procedure was repeated once, confirming that the anion exchange was complete. The product **$5d^{Cl}$** was thus obtained as a slightly beige solid (180 mg, 193 µmol, 65%).

^{1}H NMR (400 MHz, DMSO-d_6):
δ [ppm] = 11.16 – 10.68 (m, 2H), 10.37 (s, 2H), 8.21 – 8.06 (m, 1H), 8.06 – 7.59 (m, 5H), 7.57 – 7.29 (m, 3H), 6.11 – 5.91 (m, 2H), 5.20 – 5.01 (m, 2H), 5.01 – 4.75 (m, 4H), 3.43 (d, *J* = 16.8 Hz, 2H), 3.16 – 3.08 (m, 2H), 2.20 – 1.85 (m, 9H).

^{13}C NMR (101 MHz, Methanol-d_4):
δ [ppm] = 162.0, 161.6, 139.4, 139.1, 138.9, 138.7, 138.0, 137.7, 136.4, 135.2, 135.2, 132.6, 132.1, 130.3, 130.2, 130.1, 127.4, 127.2, 127.1, 125.7, 125.5, 125.1, 124.1, 123.6, 118.4, 118.1, 117.1, 94.2, 94.1, 79.1, 62.2, 62.1, 60.5, 60.4, 38.3, 38.1, 14.2, 13.5, 13.4, 8.4.

ATR-IR:
$\tilde{\nu}$ [cm^{-1}] = 3393 (m), 3107 (w), 2924 (w), 2855 (w), 2295 (w), 1699 (s), 1622 (w), 1603 (w), 1539 (s), 1533 (w), 1491 (w), 1431 (w), 1420 (w), 1373 (w), 1331 (w), 1306 (w), 1242 (w), 1213 (w), 1186 (w), 1101 (m), 1082 (m), 1070 (w), 1038 (w), 991 (w), 920 (w), 843 (s), 812 (w), 758 (w), 725 (w), 665 (m), 611 (w), 527 (w), 482 (w), 430 (w).

HRMS (MALDI-TOF):

m/*z* (+) = calc. 861.0660 [M-2Cl-H]$^+$; found 861.0663 [M-2Cl-H]$^+$.

Synthesis of (5a*S*,5a'*S*,10b*R*,10b'*R*)-2,2'-(2-methyl-1,3-phenylene)bis(1-iodo-3-methyl-9-(2,2,2-trichloroacetamido)-2,5a,6,10b-tetrahydro-4*H*-imidazo[1,5-d]indeno[2,1-b][1,4]oxazin-11-ium) trifluoromethanesulfonate (7d^{OTf})

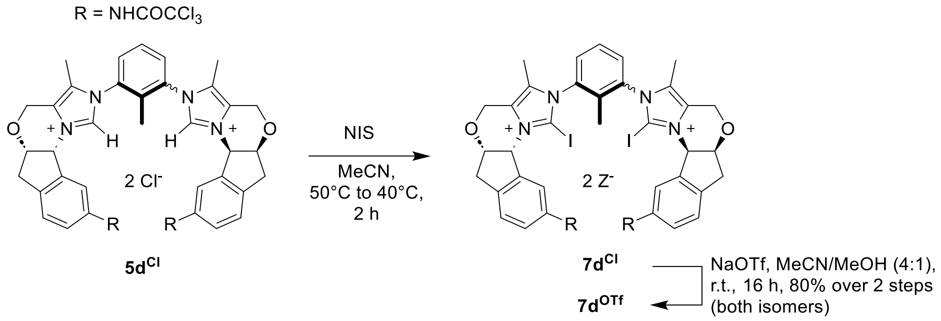

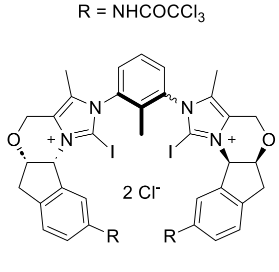

7d^{Cl}

Bis(imidazolium) chloride salt **5d^{Cl}** (166 mg, 178 µmol, 1.0 eq.) was suspended in 3.6 ml dry MeCN (0.05 M), then *N*-iodosuccinimide (87.9 mg, 391 µmol, 2.2 eq.) was added and the mixture was then heated to 50°C, after 15 minutes, it was cooled down to 40°C and stirred at this temperature for 2 hours. The solvent was then removed *in vacuo* and the obtained mixture was precipitated three times from MeCN/MeOH (4:1) by addition of Et_2O (3-4 volume equivalents) and the obtained yellowish solid (crude **7d^{Cl}**, 181 mg, 153 µmol, 86%) was finally dried under high vacuum. The crude product mixture still contained residual *N*-iodosuccinimide and solvent and was used in the next step without further purification.

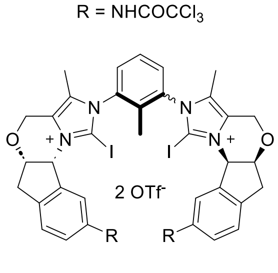

7d^{OTf}

Bis(iodoimidazolium) chloride salt **7d^{Cl}** (172 mg, 145 µmol, 1.0 eq.) was dissolved in 1.6 ml 4:1 MeCN/MeOH (0.09 M), then NaOTf (49.9 mg, 290 µmol, 2.0 eq.) was added and the mixture was stirred for 16 h at room temperature (immediate precipitation was observed upon addition of NaOTf). The solvent was then removed *in vacuo* and the residue resuspended in MeCN and filtered (syringe filter, 0.22 µm). The solvent of the resulting filtrate was then removed *in vacuo*. The mixture was once precipitated from a minimal amount of MeCN by addition of Et_2O (~10 volume equivalents). The precipitate was dried under high vacuum to yield the mixture of iodinated atropisomers **7d^{OTf}** (190 mg, 134 µmol, 93%) as a yellowish solid.

The mixture of atropisomers was then extracted with THF to achieve partial separation: 2 ml of THF was added, the obtained suspension was subjected to sonication. Following sonication, almost all of the mixture had dissolved, a few minutes after which, precipitation started, after which the suspension was stored at -30°C for two hours. The formed precipitate was then filtered off and washed with additional THF.

After this, two fractions had been obtained, both of which were then precipitated once again from minimal MeCN by addition of Et_2O to yield two fractions enriched in either atropisomer. The products were then coevaporated once with acetonitrile and dried for two days at 45°C under high vacuum.

The fraction soluble in THF, **7d^{OTf}** (fraction 1)**,** was obtained as a yellowish solid (79.2 mg, 56.0 µmol, 39% based on sum of isomers). The fraction insoluble in THF, **7d^{OTf}** (fraction 2) was obtained as a slightly beige solid (87.4 mg, 61.8 µmol, 43% based on sum of isomers).

Minor fraction (**7d^{OTf}**, fraction 1):

^{1}H NMR (400 MHz, Acetonitrile-*d*$_3$):
δ [ppm] = 9.36 – 9.05 (m, 2H), 8.03 – 7.69 (m, 5H), 7.61 – 7.33 (m, 4H), 5.86 – 5.68 (m, 2H), 5.04 – 4.71 (m, 6H), 3.43 (dd, *J* = 17.0, 3.7 Hz, 2H), 3.16 (dd, *J* = 17.1, 2.5 Hz, 2H), 2.19 – 1.86 (m, 9H).

^{13}C NMR (75 MHz, Acetonitrile-*d*$_3$):
δ [ppm] = 161.1, 161.1, 161.1, 160.7, 139.8, 139.7, 139.4, 138.7, 138.6, 138.5, 138.3, 136.8, 136.8, 136.7, 136.6, 136.5, 136.5, 136.4, 133.3, 133.2, 133.0, 131.4, 131.2, 131.1, 130.5, 130.3, 130.2, 130.1, 129.3, 129.2, 128.8, 128.7, 128.2, 127.4, 127.2, 127.2, 124.0, 123.9, 123.9, 123.8, 123.0, 120.1, 120.0, 119.7, 119.0, 101.9, 101.2, 99.4, 98.3, 93.7, 93.6, 93.4, 79.5, 79.5, 64.0, 63.9, 63.8, 63.6, 60.9, 60.9, 60.8, 60.6, 38.0, 37.9, 37.9, 14.1, 13.9, 10.2, 10.1, 10.1, 10.0.

^{19}F NMR (376 MHz, Acetonitrile-*d*$_3$):
δ [ppm] = -79.29 (s, 6F).

ATR-IR:
$\tilde{\nu}$ [cm^{-1}] = 3283 (w), 3061 (w), 2932 (w), 2857 (w), 2295 (w), 2099 (w), 1705 (m), 1636 (w), 1603 (w), 1541 (w), 1489 (w), 1435 (w), 1373 (w), 1339 (w), 1246 (s), 1225 (s), 1159 (s), 1105 (w), 1084 (w), 1026 (vs), 991 (w), 926 (w), 891 (w), 847 (m), 818 (s), 758 (w), 725 (w), 669 (w), 637 (s), 573 (w), 515 (w), 486 (w), 419 (w).

HRMS (MALDI-TOF):

m/*z* (+) = calc. 1262.8191 [M-OTf]$^+$; found 1262.8208 [M-OTf]$^+$.

calc. 1136.9225 [M-OTf-I]$^+$; found 1136.9228 [M-OTf-I]$^+$.

Major fraction (**7d^{OTf}**, fraction 2):

^{1}H NMR (400 MHz, Acetonitrile-*d*$_3$):
δ [ppm] = 9.32 – 8.97 (m, 2H), 8.02 – 7.66 (m, 5H), 7.61 – 7.30 (m, 4H), 5.88 – 5.71 (m, 2H), 5.01 – 4.74 (m, 6H), 3.49 – 3.37 (m, 2H), 3.22 – 3.10 (m, 2H), 2.18 – 2.15 (m, 5H), 2.13 – 1.96 (m, 4H).

^{13}C NMR (75 MHz, Acetonitrile-*d*$_3$):
δ [ppm] = 161.1, 160.7, 139.7, 139.7, 139.4, 138.7, 138.6, 138.5, 136.9, 136.8, 136.6, 136.5, 136.5, 135.9, 133.3, 133.0, 131.3, 131.0, 130.5, 130.0, 130.0, 129.1, 128.6, 128.5, 127.4, 127.2, 123.8, 123.8, 123.0, 121.9 (q, *J* = 320.6 Hz) 119.9, 119.0, 117.7, 99.8, 93.6, 93.4, 79.5, 63.8, 63.6, 60.9, 60.8, 60.6, 38.0, 14.2, 13.9, 10.1, 10.0, 10.0.

^{19}F NMR (376 MHz, Acetonitrile-*d*$_3$):
δ [ppm] = -79.30 (s, 6F).

ATR-IR:
$\tilde{\nu}$ [cm^{-1}] = 3275 (w), 3078 (w), 2930 (w), 2843 (w), 2290 (w), 2089 (w), 1705 (m), 1603 (w), 1533 (w), 1489 (w), 1435 (w), 1373 (w), 1339 (w), 1273 (s), 1240 (s), 1223 (s), 1159 (m), 1103 (w), 1084 (w), 1026 (vs), 926 (w), 891 (w), 843 (m), 816 (s), 789 (w), 758 (w), 746 (w), 725 (w), 669 (w), 635 (vs), 573 (w), 515 (w), 492 (w), 424 (w).

HRMS (MALDI-TOF):

m/*z* (+) = calc. 1262.8191 [M-OTf]$^+$; found 1262.8198 [M-OTf]$^+$.

Synthesis of (5a*S*,5a'*S*,10b*R*,10b'*R*)-2,2'-(2-methyl-1,3-phenylene)bis(1-iodo-3-methyl-9-(2,2,2-trichloroacetamido)-2,5a,6,10b-tetrahydro-4*H*-imidazo[1,5-d]indeno[2,1-b][1,4]oxazin-11-ium) tetrakis(3,5-bis(trifluoromethyl)phenyl)borate (7d^{BArF}, fraction 1)

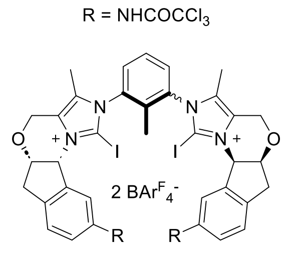

7d^{BArF}

Bis(iodoimidazolium) triflate salt **7d^{OTf}** (fraction 1) (70.0 mg, 49.5 µmol, 1.0 eq.) and NaBAr$^F_4$ (87.8 mg, 99.1 µmol, 2.0 eq.) were suspended in 0.5 ml dry acetone (0.1 M) and the mixture was stirred for 2.5 h at 42°C. The mixture was cooled to room temperature, the solvent was removed *in vacuo* and the residue dried under high vacuum. The residue was then extracted with Et_2O and the solvent of the filtrate removed *in vacuo*. The obtained residue was afterwards extracted with $CHCl_3$/DCM (1:1). The solvent of the filtrate thus obtained was removed *in vacuo* and the gained residue was precipitated with *n*-pentane from DCM. The product was then co-evaporated with DCM and dried under high vacuum at 45°C to remove residual solvent, yielding the product **7d^{BArF}** (fraction 1) as a yellowish solid (130 mg, 45.7 µmol, 92%).

^{1}H NMR (300 MHz, DCM-*d*$_2$):
δ [ppm] = 8.58 – 8.03 (m, 4H), 8.01 – 7.77 (m, 2H), 7.71 (s, 17H), 7.55 (s, 8H), 7.51 – 7.41 (m, 2H), 7.21 (dt, *J* = 8.1, 1.6 Hz, 1H), 7.13 – 7.00 (m, 1H), 5.78 – 5.63 (m, 2H), 5.01 – 4.68 (m, 6H), 3.54 – 3.39 (m, 2H), 3.39 – 3.24 (m, 2H), 2.30 – 2.09 (m, 6H), 1.94 – 1.69 (m, 3H).

^{13}C NMR (101 MHz, DCM-*d*$_2$):
δ [ppm] = 162.3 (dd, *J* = 49.8 Hz), 160.3, 160.3, 160.1, 159.8, 138.9, 138.7, 138.7, 138.6, 137.0, 136.9, 136.5, 136.4, 136.4, 136.3, 136.3, 136.2, 136.1, 136.0, 135.9, 135.8, 135.4, 134.7, 133.5, 133.2, 133.0, 132.9, 132.7, 132.2, 131.8, 131.3, 130.8, 130.8, 130.6, 130.4, 130.4, 130.2, 129.5 (qq, *J* = 31.5, 2.9 Hz), 127.8, 127.8, 127.7, 127.6, 125.2 (q, *J* = 272.4 Hz) 122.8, 122.6, 121.9, 121.1, 118.1 (p, *J* = 4.1 Hz), 117.3, 116.9, 115.8, 115.3, 95.3, 95.2, 94.3, 93.7, 93.1, 93.0, 92.8, 79.0, 79.0, 78.8, 78.7, 64.3, 64.2, 63.9, 63.9, 60.2, 60.0, 59.9, 38.0, 37.9, 37.8, 14.0, 14.0, 13.9, 10.5, 10.4, 10.3, 10.2.

^{19}F NMR (376 MHz, DCM-*d*$_2$):
δ [ppm] = -62.68 (s, 48F).

ATR-IR:
$\tilde{\nu}$ [cm^{-1}] = 3420 (w), 2926 (w), 2857 (w), 2376 (w), 2311 (w), 1734 (w), 1717 (w), 1609 (w), 1541 (w), 1526 (w), 1437 (w), 1354 (s), 1271 (vs), 1109 (vs), 932 (w), 887 (m), 839 (m), 818 (w), 810 (w), 745 (w), 712 (s), 681 (s), 669 (s), 581 (w), 534 (w), 490 (w), 447 (w).

HRMS (MALDI-TOF):

m/*z* (+) = calc. 1851.0353 [M-2BAr$^F_4$-I]$^+$; found 1851.0359 [M-2BAr$^F_4$-I]$^+$.

[α]$_D^{20}$ (c: 0.50, CH_2Cl_2): -163.4°

Synthesis of (5a*S*,5a'*S*,10b*R*,10b'*R*)-2,2'-(2-methyl-1,3-phenylene)bis(1-iodo-3-methyl-9-(2,2,2-trichloroacetamido)-2,5a,6,10b-tetrahydro-4*H*-imidazo[1,5-d]indeno[2,1-b][1,4]oxazin-11-ium) tetrakis(3,5-bis(trifluoromethyl)phenyl)borate ($7d^{BArF}$, fraction 2)

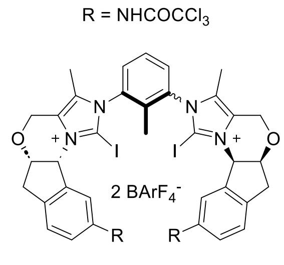

$7d^{BArF}$

Bis(iodoimidazolium) triflate salt $7d^{OTf}$ (fraction 2) (62.0 mg, 43.9 µmol, 1.0 eq.) and $NaBAr^F_4$ (77.8 mg, 87.7 µmol, 2.0 eq.) were suspended in 0.45 ml dry acetone (0.1 M) and the mixture was stirred for 2.5 h at 42°C. The mixture was cooled to room temperature, the solvent was removed *in vacuo* and the residue dried under high vacuum. The residue was then extracted with Et_2O and the solvent of the filtrate removed *in vacuo*. The obtained residue was afterwards extracted with $CHCl_3$/DCM (1:1). The solvent of the filtrate thus obtained was removed *in vacuo* and the gained residue was precipitated with *n*-pentane from DCM. The product was then co-evaporated with DCM and dried under high vacuum at 45°C to remove residual solvent, yielding the product $7d^{BArF}$ (fraction 2) as a very slightly beige solid (116 mg, 40.7 µmol, 93%).

^{1}H NMR (400 MHz, DCM-d_2):
δ [ppm] = 8.57 – 8.19 (m, 4H), 8.01 – 7.81 (m, 2H), 7.80 – 7.73 (m, 17H), 7.59 (s, 8H), 7.51 – 7.37 (m, 2H), 7.27 – 7.00 (m, 2H), 5.77 – 5.65 (m, 2H), 4.98 – 4.74 (m, 6H), 3.54 – 3.39 (m, 2H), 3.39 – 3.24 (m, 2H), 2.35 – 1.69 (m, 9H).

^{13}C NMR (101 MHz, DCM-d_2):
δ [ppm] = 162.4 (dd, *J* = 49.8 Hz), 160.3, 160.1, 159.8, 138.8, 138.7, 138.6, 137.0, 136.8, 136.5, 136.4, 136.3, 136.1, 136.0, 135.9, 135.8, 135.4, 135.3, 133.3, 133.1, 132.7, 132.3, 131.8, 131.3, 130.8, 130.7, 130.5, 130.5, 130.5, 130.2, 129.5 (qq, *J* = 31.5, 2.7 Hz), 127.8, 127.6, 125.2 (q, *J* = 272.4 Hz), 122.7, 121.9, 118.1 (p, *J* = 3.6 Hz), 117.2, 115.7, 115.3, 94.5, 94.3, 93.1, 93.0, 92.8, 79.0, 79.0, 78.8, 64.2, 63.9, 60.2, 60.0, 59.9, 38.0, 37.9, 37.8, 14.0, 13.9, 10.4, 10.3, 10.2.

^{19}F NMR (376 MHz, DCM-d_2):
δ [ppm] = -62.68 (s, 48F).

ATR-IR:
$\tilde{\nu}$ [cm^{-1}] = 3420 (w), 3102 (w), 2922 (w), 2376 (w), 2320 (w), 1730 (w), 1717 (w), 1609 (w), 1528 (w), 1522 (w), 1437 (w), 1352 (s), 1271 (vs), 1109 (vs), 930 (w), 887 (m), 839 (m), 818 (m), 810 (m), 791 (w), 745 (w), 712 (s), 681 (s), 669 (s), 615 (w), 581 (w), 536 (w), 447 (w).

HRMS (MALDI-TOF):
m/*z* (+) = calc. 1726.1421 [M-BAr^F_4-2I]$^+$; found 1726.1668 [M-BAr^F_4-2I]$^+$.

$[\alpha]_D^{20}$ (c: 0.51, CH_2Cl_2): -134.5°

Synthesis of (5a*S*,5a'*S*,10b*R*,10b'*R*)-2,2'-(2-methyl-1,3-phenylene)bis(9-isobutyramido-3-methyl-2,5a,6,10b-tetrahydro-4*H*-imidazo[1,5-d]indeno[2,1-b][1,4]oxazin-11-ium) chloride (5e^{Cl})

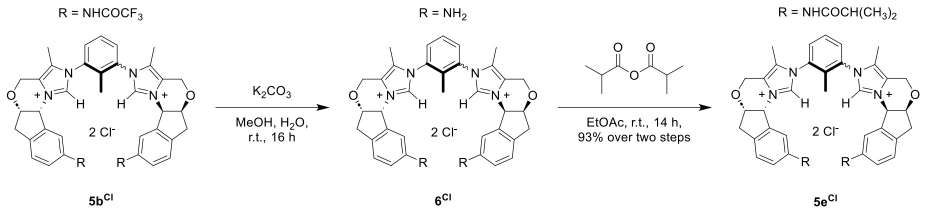

Bis(imidazolium) chloride salt **5b^{Cl}** (250 mg, 299 µmol,1.0 eq.) was dissolved in 3.0 ml MeOH/H_2O (1:1, 0.1 M) and then K_2CO_3 (82.7 mg, 598 µmol, 2.0 eq.) was added. The mixture was stirred at room temperature overnight (16 hours). Then, excess MeOH was added (20 ml) and the mixture was filtered. The solvent was then removed *in vacuo* (to remove residual water, the mixture was co-evaporated three times with absolute EtOH). Full conversion to the aniline intermediate was confirmed via ^{1}H NMR and the product mixture subsequently used as received in the next step.

The crude product mixture containing **6^{Cl}** was then suspended in EtOAc (1.2 ml, 0.25 M), then isobutyric acid anhydride (473 mg, 497 µl, 2.99 mmol, 10 eq.) was slowly added and the mixture was stirred at room temperature for 18 h. Then, Et_2O (30 ml) was added and the formed precipitate separated by filtration and afterwards washed with excess Et_2O. The obtained solid was dried under high vacuum and then purified by column chromatography (SiO_2, DCM/MeOH 8:1 → 4:1 → 1:1). The obtained solid was then dissolved in minimal MeOH and stirred with Amberlite IRA 958 Cl (1 g) for a few hours at room temperature. The resin was filtered off, rinsed with further MeOH and the solvent of the filtrate was subsequently removed *in vacuo*, yielding product **5e^{Cl}** as a beige solid (216 mg, 276 µmol, 93% over two steps).

^{1}H NMR (300 MHz, DMSO-*d*$_6$):
δ [ppm] = 10.41 – 10.05 (m, 4H), 8.20 – 7.80 (m, 5H), 7.69 – 7.22 (m, 4H), 6.04 – 5.85 (m, 2H), 5.14 – 4.77 (m, 6H), 3.43 – 3.34 (m, 2H), 3.14 – 2.97 (m, 2H), 2.77 – 2.53 (m, 2H), 2.22 – 1.88 (m, 9H), 1.13 – 0.78 (m, 12H).

^{13}C NMR (75 MHz, Methanol-*d*$_4$):
δ [ppm] = 178.7, 178.4, 139.8, 139.4, 138.6, 138.3, 137.3, 136.9, 136.3, 135.5, 135.3, 135.3, 132.7, 132.2, 130.1, 127.3, 127.0, 126.9, 125.6, 125.2, 122.6, 121.9, 116.8, 115.6, 79.1, 79.0, 62.3, 62.2, 60.4, 60.4, 38.2, 38.0, 36.9, 36.7, 20.0, 19.9, 19.9, 19.8, 13.8, 13.4, 13.3, 8.4, 8.3.

ATR-IR:
$\tilde{\nu}$ [cm^{-1}] = 3410 (w), 3117 (w), 2970 (w), 2932 (w), 2874 (w), 2293 (w), 2104 (w), 1670 (s), 1601 (m), 1541 (s), 1506 (m), 1493 (w), 1474 (w), 1429 (w), 1425 (w), 1373 (w), 1327 (w), 1300 (w), 1250 (w), 1200 (m), 1169 (m), 1126 (m), 1099 (m), 1082 (m), 1070 (m), 1038 (w), 991 (w), 937 (w), 858 (w), 812 (m), 800 (m), 750 (w), 718 (m), 656 (w), 613 (w), 519 (w).

HRMS (MALDI-TOF):

m/*z* (+) = calc. 571.2816 [M-2Cl-H]$^+$; found 571.2805 [M-2Cl-H]$^+$.

Synthesis of (5a*S*,5a'*S*,10b*R*,10b'*R*)-2,2'-(2-methyl-1,3-phenylene)bis(1-iodo-9-isobutyramido-3-methyl-2,5a,6,10b-tetrahydro-4*H*-imidazo[1,5-d]indeno[2,1-b][1,4]oxazin-11-ium) (7e^{BArF})

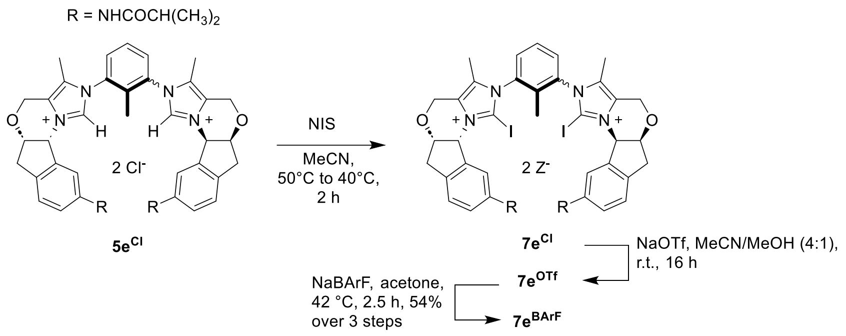

R = NHCOCH(CH$_3$)$_2$

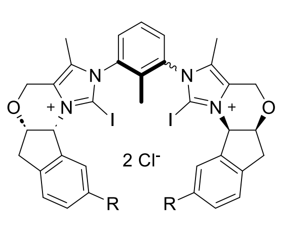

7e^{Cl}

Bis(imidazolium) chloride salt **5e^{Cl}** (202 mg, 258 µmol, 1.0 eq.) was suspended in 5.2 ml dry MeCN (0.05 M), then *N*-iodosuccinimide (128 mg, 567 µmol, 2.2 eq.) was added and the mixture was then heated to 50°C, after 15 minutes, it was cooled down to 40°C and stirred at this temperature for 2 hours. The solvent was then removed *in vacuo* and the obtained mixture was precipitated three times from MeCN/MeOH (4:1) by addition of Et_2O (3-4 volume equivalents) and the obtained yellowish solid (crude **7e^{Cl}**, 200 mg, 193 µmol, 75% crude yield) was finally dried under high vacuum. The crude product mixture still contained residual *N*-iodosuccinimide and solvent and was used in the next step without further purification.

R = NHCOCH(CH$_3$)$_2$

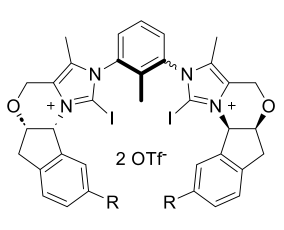

7e^{OTf}

Crude bis(iodoimidazolium) chloride salt **7e^{Cl}** (185 mg, 179 µmol, 1.0 eq.) was dissolved in 2.0 ml 4:1 MeCN/MeOH (0.09 M), then NaOTf (61.5 mg, 357 µmol, 2.0 eq.) was added and the mixture was stirred for 20 h at room temperature (immediate precipitation was observed upon addition of NaOTf). The solvent was then removed *in vacuo* and the residue resuspended in MeCN and filtered (syringe filter, 0.22 µm). The solvent of the resulting filtrate was then removed *in vacuo*. The mixture was once precipitated from a minimal amount of MeCN by addition of Et_2O (~10 volume equivalents). The precipitate was dried under high vacuum to yield the mixture of iodinated atropisomers **7e^{OTf}** (177 mg, 140 µmol, 78%) as a yellowish solid. Even though repeated attempts were made, using different solvents and conditions, no separation of the atropisomers could be achieved until now. Thus, the mixture was used as received in the next step for the final ion exchange to $BAr^F{}_4{}^-$.

R = NHCOCH(CH$_3$)$_2$

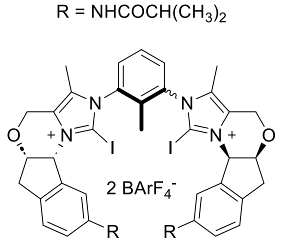

7e^{BArF}

Bis(iodoimidazolium) triflate salt **7e^{OTf}** (168 mg, 133 µmol, 1.0 eq.) and $NaBAr^F{}_4$ (236 mg, 266 µmol, 2.0 eq.) were suspended in 1.35 ml dry acetone (0.1 M) and the mixture was stirred for 2.5 h at 42°C. The mixture was cooled to room temperature, the solvent was removed *in vacuo* and the residue dried under high vacuum. The residue was then extracted with Et_2O and the solvent of the filtrate removed *in vacuo*. The obtained residue was afterwards extracted with $CHCl_3$/DCM (1:1). The solvent of the filtrate thus obtained was removed *in vacuo* and the gained residue was precipitated with *n*-pentane from DCM. The product was then co-evaporated with DCM and dried under high vacuum at 45°C to remove residual solvent, yielding the product **7e^{BArF}** as a beige solid (330 mg, 123 µmol, 92%, 54% over three steps).

^{1}H NMR (300 MHz, DCM-d_2):
δ [ppm] = 8.50 – 8.23 (m, 1H), 8.00 – 7.89 (m, 1H), 7.81 – 7.73 (m, 19H), 7.62 – 7.55 (m, 9H), 7.41 – 7.23 (m, 4H), 6.98 – 6.87 (m, 1H), 5.77 – 5.57 (m, 2H), 5.00 – 4.68 (m, 6H), 3.49 – 3.13 (m, 4H), 2.54 – 2.34 (m, 2H), 2.32 – 2.03 (m, 6H), 2.01 – 1.79 (m, 3H), 1.20 – 0.82 (m, 12H).

^{13}C NMR (75 MHz, DCM-d_2):
δ [ppm] = 177.5, 177.2, 176.9, 176.2, 162.4 (dd, *J* = 49.8 Hz), 138.6, 138.0, 137.9, 137.6, 137.3, 137.1, 136.8, 136.5, 136.4, 136.2, 136.2, 136.2, 136.1, 136.0, 135.9, 135.9, 135.5, 133.4, 133.0, 132.9, 131.9, 131.6, 130.9, 130.8, 130.3, 130.0, 130.0, 129.5 (qq, *J* = 31.5, 2.7 Hz), 127.2, 125.2 (q, J = 272.5 Hz), 122.9, 122.4, 121.4, 120.7, 118.2 (p, *J* = 3.9 Hz), 117.1, 115.6, 94.4, 93.8, 79.0, 79.0, 78.8, 78.7, 64.3, 64.1, 63.9, 60.1, 60.0, 59.9, 59.8, 37.8, 37.7, 37.3, 37.2, 37.1, 19.9, 19.8, 19.7, 19.6, 19.6, 19.6, 14.1, 13.4, 10.4, 10.3, 10.2, 10.1.

^{19}F NMR (377 MHz, DCM-d_2):
δ [ppm] = -62.77 (s, 48F).

ATR-IR:
$\tilde{\nu}$ [cm^{-1}] = 3445 (w), 2980 (w), 2938 (w), 2361 (w), 2322 (w), 1734 (w), 1674 (w), 1609 (w), 1522 (w), 1474 (w), 1437 (w), 1354 (s), 1273 (vs), 1109 (vs), 1001 (w), 932 (w), 885 (m), 839 (m), 818 (w), 744 (w), 712 (m), 681 (m), 669 (m), 617 (w), 581 (w), 449 (w).

HRMS (MALDI-TOF):

m/*z* (+) = calc. 1701.3347 [M-2BAr$^{F}_{4}$-I]$^{+}$; found 1701.3334 [M-2BAr$^{F}_{4}$-I]$^{+}$.

[α]$_{D}^{20}$ (c: 0.50, CH_2Cl_2): -138.1°

Synthesis of *anti*-2-trifluoromethyl-1,3-phenylene)bis(2-iodo-3-octyl-1H-benzo[d]imidazol-3-ium) tetrakis(3,5-bis(trifluoromethyl)phenyl)borate (SI-9^{BArF})

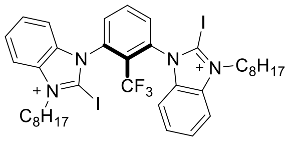

2 BArF$_4^-$

SI-9^{BArF}

Anti-bis(iodobenzimidazolium) triflate XB donor **SI-9^{OTf}** (200 mg, 173 µmol, 1.0 eq.) was dissolved in dry acetone (1.7 ml, 0.1 M) under an Ar atmosphere, after which NaBAr$^{F}_{4}$ (307 mg, 346 µmol, 2.0 eq.) was added. The mixture was stirred at 40 °C for 2 hours. The mixture was cooled to room temperature, the solvent was removed *in vacuo* and the residue dried under high vacuum. The residue was then extracted with Et_2O and the solvent of the filtrate removed *in vacuo*. The obtained residue was afterwards extracted with $CHCl_3$/DCM (1:1). The solvent of the filtrate thus obtained was removed *in vacuo* and the gained residue was precipitated with *n*-pentane from DCM. The obtained solid was co-evaporated with DCM and dried under high vacuum to yield the final product as a colorless foam (396 mg, 153 µmol, 88%).

^{1}H NMR (400 MHz, DCM-d_2):
δ [ppm] = 8.31 (t, *J* = 8.3 Hz, 1H), 7.97 (d, *J* = 8.1 Hz, 2H), 7.90 (d, *J* = 8.5 Hz, 2H), 7.77 (t, *J* = 7.7 Hz, 2H), 7.74 – 7.63 (m, 18H), 7.53 (s, 8H), 7.28 (d, *J* = 8.4 Hz, 2H), 4.72 – 4.52 (m, 4H), 2.04 (p, *J* = 7.2 Hz, 4H), 1.49 – 1.22 (m, 20H), 0.85 (t, *J* = 6.6 Hz, 6H).

^{13}C NMR (101 MHz, DCM-d_2):
δ [ppm] = 162.4 (dd, *J* = 49.8 Hz), 138.8, 136.6, 136.0, 135.4, 134.4, 133.3, 130.5, 129.9, 129.5 (qq, *J* = 31.6, 2.8 Hz), 127.9 (q, *J* = 31.9 Hz), 125.2 (q, *J* = 272.4 Hz), 118.1 (p, *J* = 3.8 Hz), 114.2, 113.3, 108.7, 52.8, 32.1, 29.7, 29.4, 29.4, 27.1, 23.1, 14.2.

^{19}F NMR (376 MHz, DCM-d_2):
δ [ppm] = -56.02 (s, 3F), -62.77 (s, 48F).

ATR-IR:
$\tilde{\nu}$ [cm^{-1}] = 2966 (w), 2942 (w), 2864 (w), 1610 (w), 1507 (w), 1477 (w), 1472 (w), 1465 (w), 1437 (w), 1435 (w), 1392 (w), 1353 (s), 1272 (vs), 1111 (vs), 1044 (w),1012 (w), 931 (w), 886 (m), 839 (s), 801 (s), 744 (s), 712 (s), 681 (vs), 668 (vs) , 638 (w), 618 (w), 616 (w), 580 (w), 452 (w), 448 (w), 423 (w).

HRMS (LIFDI-MS):
m/*z* (+) = calc. 1719.2329 [M-BAr$^F_4$]$^+$; found 1719.2329 [M-BAr$^F_4$]$^+$.

1.3. Catalysis experiments

General procedure for XB catalyzed Mukaiyama aldol reaction

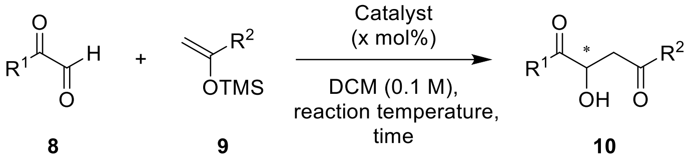

Aryl glyoxal hydrate (360 µmol, 4.0 eq.) and 600 mg of freshly activated, powdered 4 Å molecular sieves were added to a flame-dried Schlenk tube under argon atmosphere. Then 3.6 ml of dry DCM were added and the mixture was stirred for 90 minutes at room temperature, during which the suspension turned a yellow color, indicating the progress of dehydration of the glyoxal hydrate. Afterwards, the suspension was filtered via syringe filter and 0.9 ml (containing 90 µmol, 1.0 eq. of the monomeric, water-free glyoxal) of the solution (0.1 M) were added to another Schlenk tube containing catalyst (equivalents indicated in procedure) and the obtained solution was cooled to the reaction temperature. The solution was subsequently stirred for 30 minutes at this temperature. Then, silyl enol ether (180 µmol, 2 eq.) was added dropwise and the reaction mixture was stirred at the reaction temperature for the indicated time.

Then, 3 ml of THF/1 M HCl (2:1) were added at the reaction temperature and the mixture was warmed to room temperature immediately after addition. The resulting mixture was stirred vigorously for 30 minutes, after which it was added to 10 ml saturated aqueous $NaHCO_3$ solution and then extracted three times with DCM (3 x 10 ml). The combined organic extracts were dried over $MgSO_4$, and the solvent removed *in vacuo*. The crude product mixture was then purified by column chromatography (conditions: SiO_2, DCM → DCM/EtOAc (1% EtOAc), unless indicated differently) to yield the product.

2-Hydroxy-4-phenyl-1-(4-(trifluoromethyl)phenyl)butane-1,4-dione (10a)

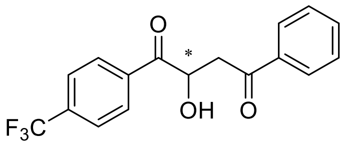

10a

According to the general procedure, (4-(trifluoromethyl)phenyl)glyoxal (18.2 mg, 90 µmol, 1.0 eq.) and trimethyl((1-phenylvinyl)oxy)silane (34.6 mg, 37 µl, 180 µmol, 2.0 eq.) were reacted using catalyst "*syn*"-**7b**$^{\mathbf{BArF}}$ (4.9 mg, 1.8 µmol, 2 mol%) in DCM (0.9 ml, 0.1 M) at -70°C for 64 h, yielding product **10a** after column chromatography as a colorless solid (21.4 mg, 66.4 µmol, 74%). The enantiomeric ratio was determined to be 97.5:2.5 (95% *ee*) by chiral HPLC analysis (CHIRAL ART Amylose-SA (YMC), *n*-heptane/*i*PrOH 93:7, 1 ml/min, 30°C, λ = 240 nm, t_R = 20.1 min (minor), t_R = 23.0 min (major)).

R_f (DCM/EtOAc (1%)): 0.30

^{1}H NMR (400 MHz, Chloroform-*d*):
δ [ppm] = 8.12 (d, *J* = 8.1 Hz, 2H), 7.94 (d, *J* = 7.4 Hz, 2H), 7.76 (d, *J* = 8.2 Hz, 2H), 7.59 (t, *J* = 7.4 Hz, 1H), 7.47 (t, *J* = 7.8 Hz, 2H), 5.57 (td, *J* = 5.9, 5.9, 4.6 Hz, 1H), 4.01 (d, *J* = 6.3 Hz, 1H), 3.55 – 3.42 (m, 2H).

^{13}C {^{1}H,^{19}F} NMR (101 MHz, DCM-*d*$_2$):
δ [ppm] = 200.6, 198.0, 137.8, 137.0, 135.1, 134.3, 129.7, 129.3, 128.7, 126.4, 124.2, 71.3, 43.3.

^{19}F NMR (376 MHz, Chloroform-*d*):
δ [ppm] = -63.27 (s, 3F).

ATR-IR:

$\tilde{\nu}$ [cm^{-1}] = 3466 (m), 3059 (w), 2955 (w), 2920 (w), 2853 (w), 1697 (w), 1674 (vs), 1597 (w), 1582 (w), 1510 (w), 1450 (w), 1427 (w), 1410 (w), 1395 (w), 1369 (w), 1331 (s), 1314 (s), 1306 (s), 1261 (m), 1202 (m), 1169 (m), 1157 (w), 1134 (m), 1117 (vs), 1069 (s), 1047 (w), 1005 (s), 962 (w), 935 (w), 880 (m), 854 (m), 843 (m), 806 (w), 772 (m), 756 (vs), 733 (m), 704 (m), 691 (s), 673 (m), 637 (w), 631 (w), 586 (m), 546 (w), 500 (w), 482 (w), 409 (w).

HRMS (MALDI-TOF):

m/*z* (+) = calc. 345.0709 [M+Na]$^+$; found 345.0714 [M+Na]$^+$.

$[\alpha]_D^{20}$ (c: 0.31, CH_2Cl_2): -8.0°

2-Hydroxy-4-(*o*-tolyl)-1-(4-(trifluoromethyl)phenyl)butane-1,4-dionedione (10b)

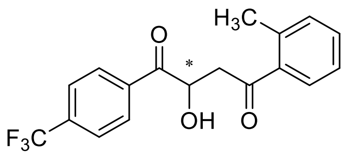

10b

According to the general procedure, (4-(trifluoromethyl)phenyl)glyoxal (18.2 mg, 90 µmol, 1.0 eq.) and trimethyl((1-(*o*-tolyl)vinyl)oxy)silane (37.1 mg, 41 µl, 180 µmol, 2.0 eq.) were reacted using catalyst "*syn*"-**7b**$^{\mathbf{BArF}}$ (4.9 mg, 1.8 µmol, 2 mol%) in DCM (0.9 ml, 0.1 M) at -50°C for 16 h, yielding product **10b** after column chromatography as a colorless oil (14.1 mg, 41.9 µmol, 47%). The enantiomeric ratio was determined to be 91.5:8.5 (83% *ee*) by chiral HPLC analysis (CHIRAL ART Amylose-SA (YMC), *n*-heptane/*i*PrOH 93:7, 1 ml/min, 30°C, λ = 240 nm, t_R = 15.6 min (minor), t_R = 17.4 min (major)).

R_f (DCM/EtOAc (1%)): 0.32

^{1}H NMR (300 MHz, DCM-*d*$_2$):

δ [ppm] = 8.10 (d, *J* = 8.1 Hz, 2H), 7.78 (d, *J* = 8.2 Hz, 2H), 7.71 – 7.56 (m, 1H), 7.47 – 7.35 (m, 1H), 7.27 (t, *J* = 7.4, 6.5 Hz, 2H), 5.51 (td, *J* = 5.7, 3.8 Hz, 1H), 3.95 (d, *J* = 6.3 Hz, 1H), 3.51 – 3.26 (m, 2H), 2.46 (s, 3H).

^{13}C NMR (75 MHz, DCM-*d*$_2$):

δ [ppm] = 201.5, 200.7, 139.2, 137.8, 137.6, 135.1 (q, *J* = 32.9 Hz), 132.6, 132.5, 129.7, 129.5, 126.4 (q, *J* = 3.8 Hz), 126.3, 71.7, 46.1, 21.6.

^{19}F NMR (376 MHz, DCM-*d*$_2$):

δ [ppm] = -63.57 (s, 3F).

ATR-IR:

$\tilde{\nu}$ [cm^{-1}] = 3447 (w), 3067 (w), 2970 (w), 2926 (w), 2851 (w), 1701 (w), 1676 (s), 1663 (s), 1601 (w), 1566 (m), 1510 (m), 1487 (m), 1454 (m), 1410 (m), 1325 (vs), 1288 (m), 1223 (m), 1206 (m), 1161 (s), 1126 (s), 1113 (m), 1069 (s), 1042 (s), 1016 (m), 999 (m), 980 (s), 959 (m), 945 (m), 860 (s), 824 (w), 795 (m), 775 (m), 766 (m), 754 (m), 737 (m), 716 (w), 700 (w), 658 (w), 590 (w), 575 (m), 561 (w), 525 (w), 501 (w), 490 (w), 471 (w), 442 (w), 409 (w).

HRMS (MALDI-TOF):

m/*z* (+) = calc. 359.0866 [M+Na]$^+$; found 359.0870 [M+Na]$^+$.

$[\alpha]_D^{20}$ (c: 0.28, CH_2Cl_2): +4.2°

2-Hydroxy-4-(*m*-tolyl)-1-(4-(trifluoromethyl)phenyl)butane-1,4-dionedione (10c)

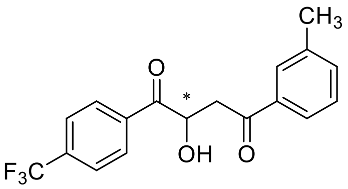

10c

According to the general procedure, (4-(trifluoromethyl)phenyl)glyoxal (18.2 mg, 90 µmol, 1.0 eq.) and trimethyl((1-(*m*-tolyl)vinyl)oxy)silane (37.1 mg, 40 µl, 180 µmol, 2.0 eq.) were reacted using catalyst "*syn*"-**7b**^{BArF} (4.9 mg, 1.8 µmol, 2 mol%) in DCM (0.9 ml, 0.1 M) at -50°C for 16 h, yielding product **10c** after column chromatography as a colorless solid (12.6 mg, 37.5 µmol, 42%). The enantiomeric ratio was determined to be 91:9 (82% *ee*) by chiral HPLC analysis (CHIRAL ART Amylose-SA (YMC), *n*-heptane/*i*PrOH 93:7, 1 ml/min, 30°C, λ = 240 nm, t_R = 16.0 min (minor), t_R = 19.5 min (major)).

R_f (DCM/EtOAc (1%)): 0.33

^{1}H NMR (400 MHz, DCM-*d₂*):
δ [ppm] = 8.10 (d, *J* = 8.2 Hz, 2H), 7.83 – 7.68 (m, 4H), 7.42 (d, *J* = 7.5 Hz, 1H), 7.36 (t, *J* = 7.6 Hz, 1H), 5.54 (td, *J* = 6.0, 4.3 Hz, 1H), 3.92 (d, *J* = 6.3 Hz, 1H), 3.53 – 3.36 (m, 2H), 2.40 (s, 3H).

^{13}C NMR {^{1}H,^{19}F} (101 MHz, DCM-d_2):
δ [ppm] = 200.7, 198.2, 139.3, 137.8, 137.1, 135.0, 135.0, 129.7, 129.2, 129.1, 126.4, 125.9, 124.2, 71.3, 43.3, 21.6.

^{19}F NMR (376 MHz, DCM-d_2):
δ [ppm] = -63.57 (s, 3F).

ATR-IR:
$\tilde{\nu}$ [cm^{-1}] = 3429 (w), 2955 (w), 2924 (w), 2853 (w), 1697 (w), 1682 (w), 1668 (s), 1647 (w), 1601 (w), 1508 (w), 1412 (w), 1337 (s), 1315 (s), 1306 (s), 1263 (w), 1234 (w), 1217 (w), 1163 (s), 1136 (s), 1119 (vs), 1070 (w), 1040 (w), 1015 (w), 984 (w), 924 (w), 893 (w), 858 (w), 851 (w), 829 (w), 789 (w), 764 (w), 725 (w), 716 (w), 702 (m), 689 (m), 675 (w), 637 (w), 588 (w), 556 (w), 467 (w), 419 (w).

HRMS (MALDI-TOF):

m/*z* (+) = calc. 359.0866 [M+Na]$^+$; found 359.0862 [M+Na]$^+$.

$[\alpha]_D^{20}$ (c: 0.26, CH_2Cl_2): -5.5°

2-Hydroxy-4-(*p*-tolyl)-1-(4-(trifluoromethyl)phenyl)butane-1,4-dionedione (10d)

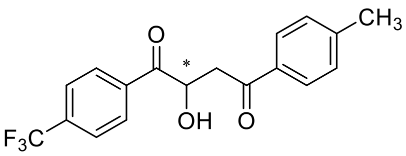

10d

According to the general procedure, (4-(trifluoromethyl)phenyl)glyoxal (18.2 mg, 90 µmol, 1.0 eq.) and trimethyl((1-(*p*-tolyl)vinyl)oxy)silane (37.1 mg, 41 µl, 180 µmol, 2.0 eq.) were reacted using catalyst "*syn*"-**7b**^{BArF} (4.9 mg, 1.8 µmol, 2 mol%) in DCM (0.9 ml, 0.1 M) at -50°C for 16 h, yielding product **10d** after column chromatography as a colorless solid (12.7 mg, 37.8 µmol, 42%). The enantiomeric ratio was determined to be 85:15 (70% *ee*) by chiral HPLC analysis (CHIRAL ART Amylose-SA (YMC), *n*-heptane/*i*PrOH 93:7, 1 ml/min, 30°C, λ = 240 nm, t_R = 21.8 min (minor), t_R = 26.1 min (major)).

R_f (DCM/EtOAc (1%)): 0.29

^{1}H NMR (400 MHz, DCM-d_2):
δ [ppm] = 8.10 (d, *J* = 8.1 Hz, 2H), 7.82 (d, *J* = 8.2 Hz, 2H), 7.77 (d, *J* = 8.2 Hz, 2H), 7.28 (d, *J* = 8.0 Hz, 2H), 5.56 – 5.50 (m, 1H), 3.93 (d, *J* = 6.3 Hz, 1H), 3.53 – 3.36 (m, 2H), 2.41 (s, 3H).

^{13}C NMR {^{1}H,^{19}F} (101 MHz, DCM-d_2):
δ [ppm] = 200.7, 197.6, 145.5, 137.8, 135.0, 134.6, 129.9, 129.7, 128.8, 126.4, 124.2, 71.4, 43.1, 22.0.

^{19}F NMR (376 MHz, DCM-d_2):
δ [ppm] = -63.57 (s, 3F).

ATR-IR:
$\tilde{\nu}$ [cm^{-1}] = 3462 (w), 3073 (w), 3032 (w), 2918 (w), 2853 (w), 1695 (w), 1674 (vs), 1653 (w), 1647 (w), 1603 (m), 1508 (w), 1410 (w), 1325 (s), 1314 (s), 1306 (s), 1200 (w), 1163 (s), 1134 (s), 1121 (vs), 1067 (s), 1009 (s), 957 (w), 881 (m), 853 (m), 843 (s), 814 (s), 802 (s), 766 (s), 723 (s), 712 (s), 702 (s), 654 (w), 584 (m), 546 (m), 484 (w), 463 (w), 409 (w).

HRMS (MALDI-TOF):

m/*z* (+) = calc. 359.0866 [M+Na]$^+$; found 359.0867 [M+Na]$^+$.

$[\alpha]_D^{20}$ (c: 0.25, CH_2Cl_2): -3.9°

2-Hydroxy-4-(4-methoxyphenyl)-1-(4-(trifluoromethyl)phenyl)butane-1,4-dione (10e)

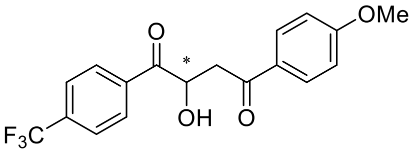

10e

According to the general procedure, (4-(trifluoromethyl)phenyl)glyoxal (18.2 mg, 90 µmol, 1 eq.) and ((1-(4-methoxyphenyl)vinyl)oxy)-trimethylsilane (40.0 mg, 42 µl, 180 µmol, 2 eq.) were reacted using catalyst "*syn*"-**7b**$^{\mathbf{BArF}}$ (4.9 mg, 1.8 µmol, 2 mol%) in DCM (0.9 ml, 0.1 M) at -50°C for 16 h, yielding product **10e** after column chromatography as a colorless solid (17.8 mg, 50.5 µmol, 56%). The enantiomeric ratio was determined to be 93.5:6.5 (87% *ee*) by chiral HPLC analysis (CHIRAL ART Amylose-SA (YMC), *n*-heptane/*i*PrOH 93:7, 1 ml/min, 30°C, λ = 240 nm, t_R = 35.7 min (minor), t_R = 41.8 min (major)).

R_f (DCM/EtOAc (1%)): 0.16

^{1}H NMR (400 MHz, DCM-d_2):
δ [ppm] = 8.11 (d, *J* = 8.1 Hz, 2H), 7.91 (dt, *J* = 8.9, 2.8, 2.0 Hz, 2H), 7.77 (d, *J* = 8.2 Hz, 2H), 6.95 (dt, *J* = 8.9, 2.8, 2.0 Hz, 2H), 5.52 (td, *J* = 6.2, 4.2 Hz, 1H), 3.95 (d, *J* = 6.3 Hz, 1H), 3.86 (s, 3H), 3.48 – 3.35 (m, 2H).

^{13}C NMR {^{1}H,^{19}F} (101 MHz, DCM-d_2):
δ [ppm] = 200.8, 196.4, 164.7, 137.9, 135.0, 131.1, 130.1, 129.7, 126.3, 124.2, 114.4, 71.5, 56.1, 42.8.

^{19}F NMR (376 MHz, DCM-d_2):
δ [ppm] = -63.57 (s, 3F).

ATR-IR:

$\tilde{\nu}$ [cm^{-1}] = 3447 (w), 3078 (w), 3015 (w), 2918 (w), 2845 (w), 1697 (m), 1670 (vs), 1599 (s), 1578 (m), 1512 (s), 1464 (w), 1410 (w), 1369 (w), 1323 (s), 1312 (s), 1261 (s), 1204 (w), 1165 (s), 1113 (vs), 1067 (s), 1030 (m), 1013 (s), 1003 (m), 881 (m), 854 (m), 829 (s), 804 (s), 777 (w), 764 (m), 723 (m), 700 (m), 652 (w), 633 (w), 583 (s), 546 (m), 511 (w), 503 (w), 484 (w), 413 (w).

HRMS (MALDI-TOF):

m/z (+) = calc. 353.0996 $[M+H]^+$; found 353.0996 $[M+H]^+$.

$[\alpha]_D^{20}$ (c: 0.25, CH_2Cl_2): -3.1°

4-(4-Fluorophenyl)-2-hydroxy-1-(4-(trifluoromethyl)phenyl)butane-1,4-dione (10f)

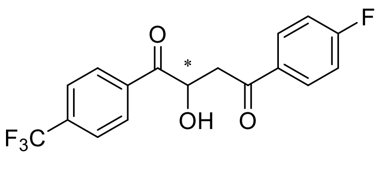

10f

According to the general procedure, (4-(trifluoromethyl)phenyl)glyoxal (18.2 mg, 90 µmol, 1.0 eq.) and ((1-(4-fluorophenyl)vinyl)oxy)trimethylsilane (37.9 mg, 39 µl, 180 µmol, 2.0 eq.) were reacted using catalyst "*syn*"-**7b**$^{\mathbf{BArF}}$ (4.9 mg, 1.8 µmol, 2 mol%) in DCM (0.9 ml, 0.1 M) at -50°C for 16 h, yielding product **10f** after column chromatography as a colorless solid (15.4 mg, 45.3 µmol, 50%). The enantiomeric ratio was determined to be 91:9 (82% *ee*) by chiral HPLC analysis (CHIRAL ART Amylose-SA (YMC), *n*-heptane/*i*PrOH 93:7, 1 ml/min, 30°C, λ = 240 nm, t_R = 21.3 min (minor), t_R = 24.7 min (major)).

R_f (DCM/EtOAc (1%)): 0.29

^{1}H NMR (300 MHz, DCM-*d*$_2$):

δ [ppm] = 8.10 (d, *J* = 8.1 Hz, 2H), 8.04 – 7.90 (m, 2H), 7.77 (d, *J* = 8.2 Hz, 2H), 7.22 – 7.07 (m, 2H), 5.54 (td, *J* = 6.4, 4.2 Hz, 1H), 3.92 (d, *J* = 6.3 Hz, 1H), 3.54 – 3.36 (m, 2H).

^{13}C NMR (75 MHz, DCM-*d*$_2$):

δ [ppm] = 200.5, 196.5, 166.7 (d, *J* = 255.1 Hz), 137.7 (d, *J* = 1.0 Hz), 135.1 (q, *J* = 32.7 Hz), 133.6 (d, *J* = 2.9 Hz), 131.6 (d, *J* = 9.5 Hz), 129.7, 126.4 (q, *J* = 3.8 Hz), 124.2 (q, *J* = 272.7 Hz), 116.5 (d, *J* = 22.1 Hz), 71.3, 43.2.

^{19}F NMR (376 MHz, DCM-*d*$_2$):

δ [ppm] = -63.59 (s, 3F), -104.97 (s, 1F).

ATR-IR:

$\tilde{\nu}$ [cm^{-1}] = 3453 (w), 3078 (w), 2961 (w), 2924 (w), 2853 (w), 1674 (vs), 1647 (w), 1597 (m), 1508 (m), 1410 (w), 1329 (s), 1315 (s), 1260 (w), 1248 (m), 1198 (m), 1179 (s), 1157 (s), 1150 (s), 1132 (vs), 1107 (m), 1070 (s), 1045 (w), 1009 (s), 881 (w), 858 (w), 839 (s), 822 (s), 768 (m), 727 (m), 704 (m), 652 (w), 583 (s), 548 (m), 494 (m), 480 (w), 409 (w).

HRMS (MALDI-TOF):

m/z (+) = calc. 363.0615 $[M+Na]^+$; found 363.0611 $[M+Na]^+$.

$[\alpha]_D^{20}$ (c: 0.26, CH_2Cl_2): -4.8°

4-(4-Bromophenyl)-2-hydroxy-1-(4-(trifluoromethyl)phenyl)butane-1,4-dione (10g)

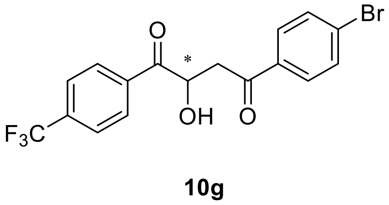

10g

According to the general procedure, (4-(trifluoromethyl)phenyl)glyoxal (18.2 mg, 90 µmol, 1.0 eq.) and ((1-(4-bromophenyl)vinyl)oxy)-trimethylsilane (48.8 mg, 42 µl, 180 µmol, 2.0 eq.) were reacted using catalyst "*syn*"-**7b**$^{\mathbf{BArF}}$ (4.9 mg, 1.8 µmol, 2 mol%) in DCM (0.9 ml, 0.1 M) at -50°C for 64 h, yielding product **10g** after column chromatography as a colorless solid (17.1 mg,

42.6 µmol, 47%). The enantiomeric ratio was determined to be 82:18 (64% *ee*) by chiral HPLC analysis (CHIRAL ART Amylose-SA (YMC), *n*-heptane/*i*PrOH 93:7, 1 ml/min, 30°C, λ = 240 nm, t_R = 25.1 min (minor), t_R = 31.1 min (major)).

R_f (DCM/EtOAc (1%)): 0.37

^{1}H NMR (400 MHz, DCM-*d*$_2$):
δ [ppm] = 8.10 (d, *J* = 8.2 Hz, 2H), 7.86 – 7.73 (m, 4H), 7.63 (d, *J* = 8.6 Hz, 2H), 5.54 (td, *J* = 6.4, 4.0 Hz, 1H), 3.89 (d, *J* = 6.3 Hz, 1H), 3.50 – 3.35 (m, 2H).

^{13}C NMR {^{1}H,^{19}F} (101 MHz, DCM-*d*$_2$):
δ [ppm] = 200.5, 197.1, 137.7, 135.9, 135.2, 132.6, 130.3, 129.7, 129.4, 126.4, 124.2, 71.2, 43.3.

^{19}F NMR (376 MHz, DCM-*d*$_2$):
δ [ppm] = -63.59 (s, 3F).

ATR-IR:

$\tilde{\nu}$ [cm^{-1}] = 3458 (w), 2955 (w), 2920 (w), 2853 (w), 1674 (vs), 1584 (s), 1566 (m), 1510 (w), 1487 (w), 1408 (w), 1398 (w), 1369 (m), 1314 (s), 1263 (m), 1177 (s), 1128 (s), 1113 (s), 1069 (s), 1045 (m), 1015 (s), 1003 (s), 978 (m), 959 (m), 883 (m), 858 (m), 841 (m), 818 (s), 772 (s), 741 (m), 702 (s), 644 (w), 627 (w), 584 (w), 546 (w), 496 (w), 482 (w), 457 (w), 411 (w).

HRMS (MALDI-TOF):

m/*z* (+) = calc. 422.9815 [M+Na]$^+$; found 422.9886 [M+Na]$^+$.

$[\alpha]_D^{20}$ (c: 0.26, CH_2Cl_2): +0.8°

4-Cyclohexyl-2-hydroxy-1-(4-(trifluoromethyl)phenyl)butane-1,4-dione (10h)

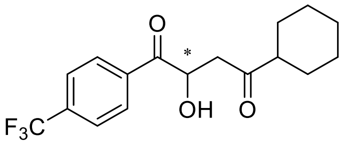

10h

According to the general procedure, (4-(trifluoromethyl)phenyl)glyoxal (18.2 mg, 90 µmol, 1.0 eq.) and ((1-cyclohexylvinyl)oxy)trimethylsilane (35.7 mg, 40 µl, 180 µmol, 2.0 eq.) were reacted using catalyst "*syn*"-**7b^{BArF}** (4.9 mg, 1.8 µmol, 2 mol%) in DCM (0.9 ml, 0.1 M) at -50°C for 16 h, yielding product **10h** after column chromatography as a colorless solid (15.5 mg, 47.2 µmol, 52%). The enantiomeric ratio was determined to be 93:7 (86% *ee*) by chiral HPLC analysis (CHIRAL ART Amylose-SA (YMC), *n*-heptane/*i*PrOH 93:7, 1 ml/min, 30°C, λ = 240 nm, t_R = 11.9 min (minor), t_R = 14.0 min (major)).

R_f (DCM/EtOAc (1%)): 0.37

^{1}H NMR (300 MHz, DCM-*d*$_2$):
δ [ppm] = 8.04 (d, *J* = 8.1 Hz, 2H), 7.76 (d, *J* = 8.2 Hz, 2H), 5.39 – 5.33 (m, 1H), 3.85 (d, *J* = 6.3 Hz, 1H), 3.01 – 2.80 (m, 2H), 2.42 – 2.25 (m, 1H), 1.87 – 1.58 (m, 5H), 1.35 – 1.11 (m, 5H).

^{13}C NMR (75 MHz, DCM-*d*$_2$):
δ [ppm] = 211.9, 200.7, 137.7, 135.0 (q, *J* = 32.6 Hz), 129.6, 126.32 (q, *J* = 3.8 Hz), 124.2 (q, *J* = 272.7 Hz), 71.3, 51.8, 45.0, 28.6, 28.6, 26.4, 26.1, 26.1.

^{19}F NMR (376 MHz, DCM-*d*$_2$):
δ [ppm] = -63.58 (s, 3F).

ATR-IR:
$\tilde{\nu}$ [cm^{-1}] = 3458 (w), 2955 (w), 2920 (w), 2853 (w), 1674 (vs), 1584 (s), 1566 (m), 1510 (w), 1487 (w), 1408 (w), 1398 (w), 1369 (m), 1314 (s), 1263 (m), 1177 (s), 1128 (s), 1113 (s), 1069 (s), 1045 (m), 1015 (s), 1003 (s), 978 (m), 959 (m), 883 (m), 858 (m), 841 (m), 818 (s), 772 (s), 741 (m), 702 (s), 644 (w), 627 (w), 584 (w), 546 (w), 496 (w), 482 (w), 457 (w), 411 (w).

HRMS (MALDI-TOF):

m/*z* (+) = calc. 351.1179 [M+Na]$^+$; found 351.1255 [M+Na]$^+$.

[α]$_D^{20}$ (c: 0.30, CH_2Cl_2): +2.9°

2-Hydroxy-4-(thiophen-2-yl)-1-(4-(trifluoromethyl)phenyl)-2λ3-butane-1,4-dione (10i)

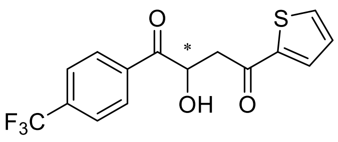

10i

According to the general procedure, (4-(trifluoromethyl)phenyl)glyoxal (18.2 mg, 90 µmol, 1.0 eq.) and trimethyl((1-(thiophen-2-yl)vinyl)oxy)silane (35.7 mg, 35 µl, 180 µmol, 2.0 eq.) were reacted using catalyst "*syn*"-**7b**$^{\mathbf{BArF}}$ (4.9 mg, 1.8 µmol, 2 mol%) in DCM (0.9 ml, 0.1 M) at -50°C for 16 h, yielding product **10i** after column chromatography as a colorless solid (8.8 mg, 26.8 µmol, 30%). The enantiomeric ratio was determined to be 85:15 (70% *ee*) by chiral HPLC analysis (CHIRAL ART Amylose-SA (YMC), *n*-heptane/*i*PrOH 93:7, 1 ml/min, 30°C, λ = 240 nm, t_R = 27.7 min (minor), t_R = 30.7 min (major)).

R_f (DCM/EtOAc (1%)): 0.23

^{1}H NMR (400 MHz, DCM-*d*$_2$):
δ [ppm] = 8.10 (d, *J* = 8.2 Hz, 2H), 7.78 (d, *J* = 8.2 Hz, 2H), 7.73 (t, *J* = 5.3, 4.1 Hz, 2H), 7.16 (t, *J* = 4.6, 4.2 Hz, 1H), 5.53 (td, *J* = 6.4, 3.7 Hz, 1H), 3.91 (d, *J* = 6.2 Hz, 1H), 3.47 – 3.32 (m, 2H).

^{13}C NMR {^{1}H,^{19}F} (101 MHz, DCM-*d*$_2$):
δ [ppm] = 200.4, 190.6, 144.2, 137.6, 135.4, 135.1, 133.6, 129.7, 129.0, 126.4, 124.2, 71.3, 44.0.

^{19}F NMR (376 MHz, DCM-*d*$_2$):
δ [ppm] = -63.59 (s, 3F).

ATR-IR:
$\tilde{\nu}$ [cm^{-1}] = 3468 (w), 3121 (w), 3086 (w), 2959 (w), 2922 (w), 1697 (w), 1686 (m), 1661 (s), 1651 (s), 1582 (w), 1514 (w), 1412 (s), 1333 (s), 1314 (s), 1304 (s), 1260 (m), 1238 (w), 1204 (m), 1173 (m), 1136 (m), 1107 (s), 1082 (w), 1070 (m), 1061 (m), 1047 (m), 1024 (m), 1015 (m), 989 (m), 961 (w), 941 (w), 918 (w), 872 (m), 860 (m), 837 (s), 800 (m), 764 (w), 729 (s), 718 (vs), 702 (s), 667 (w), 635 (w), 631 (w), 604 (w), 588 (w), 554 (w), 534 (w), 496 (w), 478 (w), 453 (w), 409 (w).

HRMS (MALDI-TOF):

m/*z* (+) = calc. 351.0274 [M+Na]$^+$; found 351.0266 [M+Na]$^+$.

[α]$_D^{20}$ (c: 0.26, CH_2Cl_2): -0.4°

4-(Benzo[b]thiophen-2-yl)-2-hydroxy-1-(4-(trifluoromethyl)phenyl)butane-1,4-dione (10j)

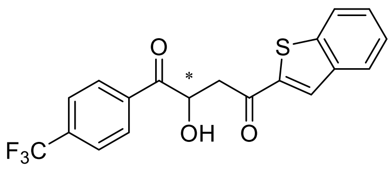

10j

According to the general procedure, (4-(trifluoromethyl)phenyl)glyoxal (18.2 mg, 90 µmol, 1.0 eq.) and ((1-(benzo[b]thiophen-2-yl)vinyl)oxy)trimethylsilane (44.7 mg, 42 µl, 180 µmol, 2.0 eq.) were reacted using catalyst "*syn*"-**7b^{BArF}** (4.9 mg, 1.8 µmol, 2 mol%) in DCM (0.9 ml, 0.1 M) at -50°C for 16 h, yielding product **10j** after column chromatography as a colorless solid (13.6 mg, 35.9 µmol, 40%). The enantiomeric ratio was determined to be 99:1 (98% *ee*) by chiral HPLC analysis (CHIRAL ART Amylose-SA (YMC), *n*-heptane/*i*PrOH 93:7, 1 ml/min, 30°C, λ = 240 nm, t_R = 32.7 min (minor), t_R = 41.7 min (major)).

R_f (DCM/EtOAc (1%)): 0.42

^{1}H NMR (400 MHz, DCM-*d$_2$*):
δ [ppm] = 8.12 (d, *J* = 8.1 Hz, 2H), 8.00 (s, 1H), 7.91 (t, J = 9.5, 8.7 Hz, 2H), 7.79 (d, *J* = 8.1 Hz, 2H), 7.50 (t, *J* = 7.9, 7.2 Hz, 1H), 7.43 (t, *J* = 7.9, 7.1 Hz, 1H), 5.62 – 5.52 (m, 1H), 3.92 (d, *J* = 6.2 Hz, 1H), 3.61 – 3.40 (m, 2H).

^{13}C NMR {^{1}H,^{19}F} (101 MHz, DCM-*d$_2$*):
δ [ppm] = 200.3, 192.2, 143.6, 143.3, 139.6, 137.5, 135.2, 131.0, 129.7, 128.5, 126.8, 126.5, 125.8, 124.2, 123.5, 71.3, 43.9.

^{19}F NMR (376 MHz, DCM-*d$_2$*):
δ [ppm] = -63.60 (s, 3F).

ATR-IR:
$\tilde{\nu}$ [cm^{-1}] = 3441 (w), 3067 (w), 2953 (w), 2922 (w), 2853 (w), 1697 (m), 1651 (vs), 1593 (w), 1578 (w), 1558 (w), 1516 (m), 1508 (m), 1458 (w), 1429 (w), 1410 (w), 1331 (s), 1314 (s), 1261 (w), 1254 (w), 1233 (w), 1213 (w), 1169 (s), 1136 (s), 1121 (vs), 1069 (s), 1047 (w), 1015 (w), 986 (w), 962 (w), 934 (w), 883 (m), 860 (m), 833 (s), 762 (m), 746 (s), 727 (s), 708 (s), 698 (s), 638 (w), 590 (w), 581 (w), 569 (w), 550 (w), 536 (w), 467 (w).

HRMS (MALDI-TOF):

m/*z* (+) = calc. 401.0430 [M+Na]$^+$; found 401.0601 [M+Na]$^+$.

[α]$_D^{20}$ (c: 0.17, CH_2Cl_2): +9.5°

4-([1,1'-Biphenyl]-4-yl)-2-hydroxy-1-(4-(trifluoromethyl)phenyl)butane-1,4-dione (10k)

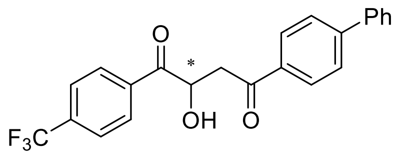

10k

According to the general procedure, (4-(trifluoromethyl)phenyl)glyoxal (18.2 mg, 90 µmol, 1.0 eq.) and ((1-([1,1'-biphenyl]-4-yl)vinyl)oxy)trimethylsilane (48.3 mg, 50 µl, 180 µmol, 2.0 eq.) were reacted using catalyst "*syn*"-**7b^{BArF}** (4.9 mg, 1.8 µmol, 2 mol%) in DCM (0.9 ml, 0.1 M) at -50°C for 16 h, yielding product **10k** after column chromatography as a colorless solid (16.7 mg, 41.9 µmol, 47%). The enantiomeric ratio was determined to be 98.5:1.5 (97% *ee*) by chiral HPLC analysis (CHIRAL ART Amylose-SA (YMC), *n*-heptane/*i*PrOH 93:7, 1 ml/min, 30°C, λ = 240 nm, t_R = 35.3 min (minor), t_R = 46.1 min (major)).

R_f (DCM/EtOAc (1%)): 0.4

^{1}H NMR (400 MHz, DMSO-d_6):
δ [ppm] = 8.22 (d, *J* = 8.1 Hz, 2H), 8.07 (d, *J* = 8.3 Hz, 2H), 7.93 (d, *J* = 8.2 Hz, 2H), 7.84 (d, *J* = 8.3 Hz, 2H), 7.76 (d, *J* = 7.6 Hz, 2H), 7.52 (t, *J* = 7.3 Hz, 2H), 7.44 (t, *J* = 7.3 Hz, 1H), 5.97 (d, *J* = 7.4 Hz, 1H), 5.47 (dt, *J* = 6.9, 6.4 Hz, 1H), 3.64 (dd, *J* = 17.3, 6.5 Hz, 1H), 3.46 (dd, *J* = 17.3, 6.0 Hz, 1H).

^{13}C NMR {^{1}H,^{19}F} (101 MHz, DMSO-d_6):
δ [ppm] = 198.9, 197.4, 144.7, 138.9, 138.7, 135.3, 132.4, 129.6, 129.1, 128.8, 128.4, 127.0, 126.9, 125.6, 123.8, 69.1, 42.1.

^{19}F NMR (376 MHz, DMSO-d_6):
δ [ppm] = -61.58 (s, 3F).

ATR-IR:
ṽ [cm^{-1}] = 3422 (w), 3096 (w), 3040 (w), 2920 (w), 2851 (w), 1697 (m), 1684 (m), 1663 (s), 1647 (m), 1603 (m), 1558 (w), 1533 (w), 1508 (w), 1489 (w), 1406 (m), 1373 (w), 1337 (s), 1319 (s), 1306 (s), 1261 (m), 1234 (w), 1177 (w), 1161 (w), 1136 (m), 1121 (s), 1070 (s), 1043 (m), 1015 (m), 1003 (m), 984 (w), 887 (m), 858 (m), 849 (m), 843 (m), 833 (m), 773 (w), 762 (vs), 748 (m), 718 (m), 694 (m), 687 (m), 658 (w), 625 (w), 588 (m), 552 (w), 473 (w), 442 (w), 411 (w).

HRMS (MALDI-TOF):
m/z (+) = calc. 421.1022 [M+Na]$^+$; found 421.1019 [M+Na]$^+$.

$[\alpha]_D^{20}$ (c: 0.14, CH_2Cl_2): +4.9°

2-Hydroxy-4-(naphthalen-2-yl)-1-(4-(trifluoromethyl)phenyl)butane-1,4-dione (10l)

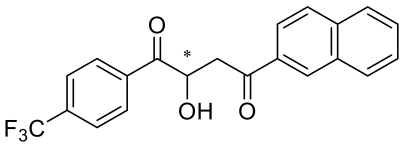

10l

According to the general procedure, (4-(trifluoromethyl)phenyl)glyoxal (18.2 mg, 90 µmol, 1.0 eq.) and trimethyl((1-(naphthalen-2-yl)vinyl)oxy)silane (43.6 mg, 44 µl, 180 µmol, 2.0 eq.) were reacted using catalyst “*syn*”-**7b^{BArF}** (4.9 mg, 1.8 µmol, 2 mol%) in DCM (0.9 ml, 0.1 M) at -50°C for 16 h, yielding product **10l** after column chromatography as a colorless solid (14.4 mg, 38.7 µmol, 43%). The enantiomeric ratio was determined to be 97:3 (94% *ee*) by chiral HPLC analysis (CHIRAL ART Amylose-SA (YMC), *n*-heptane/*i*PrOH 93:7, 1 ml/min, 30°C, λ = 240 nm, t_R = 28.6 min (minor), t_R = 40.8 min (major)).

R_f (DCM/EtOAc (1%)): 0.28

^{1}H NMR (400 MHz, DCM-d_2):
δ [ppm] = 8.47 (s, 1H), 8.14 (d, *J* = 8.2 Hz, 2H), 8.04 – 7.95 (m, 2H), 7.91 (t, *J* = 8.0, 6.9 Hz, 2H), 7.79 (d, *J* = 8.2 Hz, 2H), 7.68 – 7.53 (m, 2H), 5.65 – 5.57 (m, 1H), 3.96 (d, *J* = 6.3 Hz, 1H), 3.68 – 3.55 (m, 2H).

^{13}C NMR {^{1}H,^{19}F} (101 MHz, DCM-d_2):
δ [ppm] = 200.7, 197.9, 137.8, 136.4, 135.1, 134.4, 133.0, 130.9, 130.2, 129.7, 129.4, 129.1, 128.3, 127.6, 126.4, 124.2, 124.1, 71.4, 43.4.

^{19}F NMR (376 MHz, DCM-d_2):
δ [ppm] = -63.57 (s, 3F).

ATR-IR:
$\tilde{\nu}$ [cm^{-1}] = 3420 (w), 3291 (w), 3065 (w), 2961 (w), 2922 (w), 2853 (w), 1697 (w), 1678 (vs), 1670 (vs), 1653 (m), 1622 (m), 1508 (m), 1472 (w), 1410 (w), 1333 (vs), 1314 (vs), 1304 (vs), 1275 (w), 1265 (w), 1234 (w), 1225 (w), 1173 (m), 1150 (m), 1119 (vs), 1069 (s), 1045 (w), 1032 (w), 1015 (w), 1007 (w), 991 (w), 982 (w), 968 (w), 961 (w), 943 (w), 922 (w), 887 (w), 858 (m), 849 (m), 829 (m), 818 (vs), 762 (m), 746 (m), 721 (m), 702 (m), 660 (w), 650 (w), 592 (w), 571 (w), 559 (w), 546 (w), 488 (w), 478 (w), 461 (w).

HRMS (MALDI-TOF):

m/z (+) = calc. 395.0866 $[M+Na]^+$; found 395.0861 $[M+Na]^+$.

$[\alpha]_D^{20}$ (c: 0.27, CH_2Cl_2): -0.7°

1-(4-Bromophenyl)-2-hydroxy-4-phenylbutane-1,4-dione (10s)

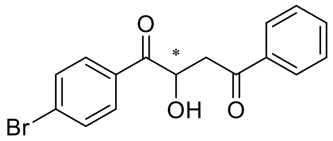

10s

According to the general procedure, 4-bromophenylglyoxal (19.2 mg, 90 µmol, 1.0 eq.) and trimethyl((1-phenylvinyl)oxy)silane (34.6 mg, 37 µl, 180 µmol, 2.0 eq.) were reacted using catalyst "*syn*"-**7b^{BArF}** (4.9 mg, 1.8 µmol, 2 mol%) in DCM (0.9 ml, 0.1 M) at -50°C for 16 h, yielding product **10s** after column chromatography as a colorless solid (14.7 mg, 44.1 µmol, 49%). The enantiomeric ratio was determined to be 97:3 (94% *ee*) by chiral HPLC analysis (Reprosil Chiral-NR (Dr. Maisch), *n*-heptane/*i*PrOH 93:7, 1 ml/min, 30°C, λ = 240 nm, t_R = 26.7 min (minor), t_R = 34.3 min (major)).

R_f (DCM/EtOAc (1%)): 0.29

^{1}H NMR (400 MHz, Chloroform-*d*):
δ [ppm] = δ 7.94 (d, *J* = 7.5 Hz, 2H), 7.87 (d, *J* = 8.5 Hz, 2H), 7.64 (d, *J* = 8.5 Hz, 2H), 7.58 (t, *J* = 7.4 Hz, 1H), 7.46 (t, *J* = 7.7 Hz, 2H), 5.56 (td, *J* = 6.9, 3.6 Hz, 1H), 4.00 (d, *J* = 6.2 Hz, 1H), 3.54 – 3.32 (m, 2H).

$[\alpha]_D^{20}$ (c: 0.29, CH_2Cl_2): +6.2°

Spectroscopic data was found to be in accordance with literature.[59]

2-Hydroxy-1-(4-isopropylphenyl)-4-phenylbutane-1,4-dione (10t)

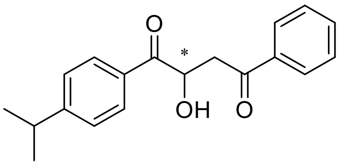

10t

According to the general procedure, (4-(isopropyl)phenyl)glyoxal (15.9 mg, 90 µmol, 1.0 eq.) and trimethyl((1-phenylvinyl)oxy)silane (34.6 mg, 37 µl, 180 µmol, 2.0 eq.) were reacted using catalyst "*syn*"-**7b^{BArF}** (4.9 mg, 1.8 µmol, 2 mol%) in DCM (0.9 ml, 0.1 M) at -50°C for 16 h, yielding product **10t** after column chromatography as a colorless oil (13.6 mg, 45.9 µmol, 51%). The enantiomeric ratio was determined to be 87.5:12.5 (75% *ee*) by chiral HPLC analysis (CHIRAL ART Amylose-SA (YMC), *n*-heptane/*i*PrOH 93:7, 1 ml/min, 30°C, λ = 240 nm, t_R = 21.1 min (minor), t_R = 22.5 min (major)).

R_f (DCM/EtOAc (1%)): 0.24

^{1}H NMR (400 MHz, DCM-d_2):
δ [ppm] = 7.92 (t, *J* = 8.3, 7.3 Hz, 4H), 4H), 7.59 (t, *J* = 7.4 Hz, 1H), 7.47 (t, *J* = 7.8 Hz, 2H), 7.37 (d, *J* = 8.3 Hz, 2H), 5.69 – 5.54 (m, 1H), 3.93 (d, *J* = 6.0 Hz, 1H), 3.45 – 3.32 (m, 2H), 2.98 (hept, *J* = 6.9 Hz, 1H), 1.27 (d, *J* = 6.9 Hz, 6H).

^{13}C NMR (101 MHz, DCM-d_2):
δ [ppm] = 201.0, 197.6, 156.3, 137.4, 134.0, 131.9, 129.4, 129.2, 128.7, 127.6, 70.4, 44.4, 34.9, 23.9, 23.9.

ATR-IR:
$\tilde{\nu}$ [cm^{-1}] = 3456 (w), 3071 (w), 3051 (w), 3036 (w), 2961 (w), 2924 (w), 2866 (w), 1690 (m), 1674 (vs), 1649 (m), 1638 (w), 1605 (m), 1597 (m), 1570 (w), 1558 (w), 1464 (w), 1458 (w), 1447 (m), 1416 (w), 1362 (w), 1341 (w), 1310 (w), 1300 (m), 1290 (m), 1260 (m), 1227 (m), 1202 (m), 1113 (w), 1055 (w), 1024 (w), 1007 (m), 976 (w), 930 (w), 874 (w), 837 (w), 777 (w), 748 (s), 723 (w), 689 (s), 646 (w), 584 (m), 548 (w), 532 (w).

HRMS (MALDI-TOF):

m/z (+) = calc. 319.1305 [M+Na]$^+$; found 319.1305 [M+Na]$^+$.

$[\alpha]_D^{20}$ (c: 0.27, CH_2Cl_2): +2.0°

4-(2-Hydroxy-4-oxo-4-phenylbutanoyl)benzonitrile (10u)

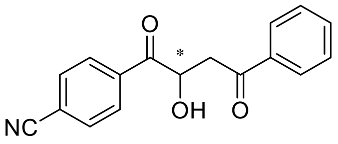

10u

According to the general procedure, (4-(cyano)phenyl)glyoxal (14.3 mg, 90 µmol, 1.0 eq.) and trimethyl((1-phenylvinyl)oxy)silane (34.6 mg, 37 µl, 180 µmol, 2.0 eq.) were reacted using catalyst "*syn*"-**7b$^{\mathbf{BArF}}$** (4.9 mg, 1.8 µmol, 2 mol%) in DCM (0.9 ml, 0.1 M) at -50°C for 16 h, yielding product **10u** after column chromatography as a colorless solid (13.8 mg, 49.4 µmol, 55%). The enantiomeric ratio was determined to be 86.5:13.5 (73% *ee*) by chiral HPLC analysis (Reprosil Chiral-NR (Dr. Maisch), *n*-heptane/*i*PrOH 93:7, 1 ml/min, 30°C, λ = 240 nm, t_R = 55.4 min (minor), t_R = 60.6 min (major)).

R_f (DCM/EtOAc (1%)): 0.20

^{1}H NMR (400 MHz, DCM-d_2):
δ [ppm] = 8.08 (d, *J* = 8.0 Hz, 2H), 7.93 (d, *J* = 7.6 Hz, 2H), 7.80 (d, *J* = 8.0 Hz, 2H), 7.61 (t, *J* = 7.2 Hz, 1H), 7.49 (t, *J* = 7.5 Hz, 2H), 5.56 – 5.44 (m, 1H), 4.01 – 3.83 (m, 1H), 3.60 – 3.41 (m, 2H).

^{13}C NMR (101 MHz, DCM-d_2):
δ [ppm] = 200.3, 198.2, 138.1, 137.0, 134.3, 133.2, 129.7, 129.3, 128.7, 118.4, 117.3, 71.4, 43.0.

ATR-IR:
$\tilde{\nu}$ [cm^{-1}] = 3447 (w), 3096 (w), 3049 (w), 2970 (w), 2928 (w), 2853 (w), 2230 (m), 1694 (m), 1670 (vs), 1655 (m), 1647 (m), 1638 (w), 1595 (m), 1447 (m), 1404 (w), 1379 (m), 1321 (s), 1314 (s), 1306 (s), 1294 (s), 1261 (w), 1198 (s), 1184 (s), 1173 (s), 1103 (s), 1047 (w), 1007 (m), 980 (w), 930 (w), 880 (m), 839 (m), 756 (vs), 718 (w), 689 (s), 637 (w), 584 (m), 567 (m), 544 (m), 532 (m).

HRMS (MALDI-TOF):

m/z (+) = calc. 302.0788 [M+Na]$^+$; found 302.0774 [M+Na]$^+$.

$[\alpha]_D^{20}$ (c: 0.27, CH_2Cl_2): -3.0°

1-(4-Fluorophenyl)-2-hydroxy-4-phenylbutane-1,4-dione (10v)

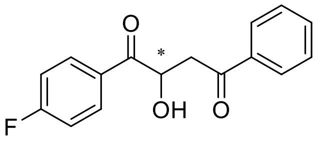

10v

According to the general procedure, 4-fluorophenylglyoxal (13.7 mg, 90 µmol, 1.0 eq.) and trimethyl((1-phenylvinyl)oxy)silane (34.6 mg, 37 µl, 180 µmol, 2.0 eq.) were reacted using catalyst "*syn*"-**7b**$^{\mathbf{BArF}}$ (4.9 mg, 1.8 µmol, 2 mol%) in DCM (0.9 ml, 0.1 M) at -50°C for 40 h, yielding product **10v** after column chromatography as a colorless solid (16.5 mg, 60.6 µmol, 67%). The enantiomeric ratio was determined to be 90.5:9.5 (81% *ee*) by chiral HPLC analysis (Reprosil Chiral-NR (Dr. Maisch), *n*-heptane/*i*PrOH 93:7, 1 ml/min, 30°C, λ = 240 nm, t_R = 25.1 min (minor), t_R = 31.0 min (major)).

R_f (DCM/EtOAc (1%)): 0.25

^{1}H NMR (400 MHz, Chloroform-*d*):
δ [ppm] = 8.10 – 8.01 (m, 2H), 7.95 (d, *J* = 7.4 Hz, 2H), 7.58 (t, *J* = 7.4 Hz, 1H), 7.46 (t, *J* = 7.7 Hz, 2H), 7.17 (t, *J* = 8.5 Hz, 2H), 5.60 (td, *J* = 6.9, 3.3 Hz, 1H), 4.01 (d, *J* = 6.3 Hz, 1H), 3.52 – 3.30 (m, 2H).

^{19}F NMR (376 MHz, Chloroform-*d*):
δ [ppm] = -103.23 (s, 1F).

Spectroscopic data was found to be in accordance with literature.[59]

$[\alpha]_D^{20}$ (c: 0.27, CH_2Cl_2): -18.9°

2-Hydroxy-1,4-diphenylbutane-1,4-dione (10w)

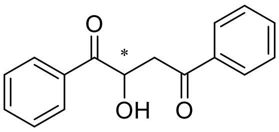

10w

According to the general procedure, phenylglyoxal (12.1 mg, 90 µmol, 1.0 eq.) and trimethyl((1-phenylvinyl)oxy)silane (34.6 mg, 37 µl, 180 µmol, 2.0 eq.) were reacted using catalyst "*syn*"-**7b**$^{\mathbf{BArF}}$ (4.9 mg, 1.8 µmol, 2 mol%) in DCM (0.9 ml, 0.1 M) at -50°C for 40 h, yielding product **10w** after column chromatography as a colorless solid (17.6 mg, 69.2 µmol, 77%). The enantiomeric ratio was determined to be 83.5:16.5 (67% *ee*) by chiral HPLC analysis (Reprosil Chiral-NR (Dr. Maisch), *n*-heptane/*i*PrOH 93:7, 1 ml/min, 30°C, λ = 240 nm, t_R = 31.1 min (minor), t_R = 42.5 min (major)).

R_f (DCM/EtOAc (1%)): 0.22

^{1}H NMR (400 MHz, Chloroform-*d*):
δ [ppm] = 8.00 (d, *J* = 7.3 Hz, 2H), 7.94 (d, *J* = 7.3 Hz, 2H), 7.65 – 7.54 (m, 2H), 7.54 – 7.41 (m, 4H), 5.68 (td, *J* = 6.6, 3.5 Hz, 1H), 4.01 (d, *J* = 6.1 Hz, 1H), 3.49 – 3.32 (m, 2H).

$[\alpha]_D^{20}$ (c: 0.31, CH_2Cl_2): -8.4°

Spectroscopic data was found to be in accordance with literature.[59]

Methyl 4-(2-hydroxy-4-oxo-4-phenyl-2λ3-butanoyl)benzoate (10x)

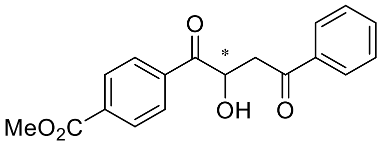

10x

According to the general procedure, (4-(carboxymethyl)phenyl)glyoxal (18.9 mg, 90 µmol, 1.0 eq.) and trimethyl((1-phenylvinyl)oxy)silane (34.6 mg, 37 µl, 180 µmol, 2.0 eq.) were reacted using catalyst "*syn*"-**7b**$^{\mathbf{BArF}}$ (4.9 mg,

1.8 µmol, 2 mol%) in DCM (0.9 ml, 0.1 M) at -50°C for 16 h, yielding product **10x** after column chromatography (generally, SiO_2, DCM → DCM/EtOAc (5% EtOAc) as a colorless solid (15.1 mg, 48.3 µmol, 54%). The enantiomeric ratio was determined to be 99:1 (98% *ee*) by chiral HPLC analysis (CHIRAL ART Amylose-SA (YMC), *n*-heptane/*i*PrOH 93:7, 1 ml/min, 30°C, λ = 240 nm, t_R = 41.9 min (minor), t_R = 44.7 min (major)).

R_f (DCM/EtOAc (5%)): 0.25

^{1}H NMR (400 MHz, DCM-*d₂*):
δ [ppm] = 8.13 (d, *J* = 8.3 Hz, 2H), 8.03 (d, *J* = 8.4 Hz, 2H), 7.96 – 7.86 (m, 2H), 7.65 – 7.55 (m, 1H), 7.53 – 7.42 (m, 2H), 5.57 (td, *J* = 6.3, 4.0 Hz, 1H), 3.96 – 3.88 (m, 4H), 3.53 – 3.38 (m, 2H).

^{13}C NMR (101 MHz, DCM-d_2):
δ [ppm] = 201.0, 197.8, 166.4, 138.0, 137.1, 135.1, 134.2, 130.4, 129.2, 129.2, 128.7, 71.1, 53.0, 43.5.

ATR-IR:
$\tilde{\nu}$ [cm^{-1}] = 3470 (w), 3442 (w), 3069 (w), 2947 (w), 2921 (w), 2849 (w), 1710 (vs), 1692 (s), 1674 (vs), 1597 (w), 1572 (w), 1503 (w), 1454 (w), 1439 (m), 1408 (w), 1379 (w), 1324 (w), 1312 (w), 1281 (vs), 1196 (m), 1186 (m), 1175 (w), 1118 (m), 1110 (s), 1054 (w), 1010 (m), 966 (w), 935 (w), 888 (w), 862 (w), 851 (w), 829 (w), 789 (w), 760 (m), 752 (s), 719 (vs), 691 (vs), 672 (w), 627 (w), 585 (m), 556 (m), 505 (w), 502 (w), 500 (w), 497 (w), 485 (w).

HRMS (MALDI-TOF):

m/*z* (+) = calc. 335.0890 $[M+Na]^+$; found 335.0885 $[M+Na]^+$.

$[\alpha]_D^{20}$ (c: 0.24, CH_2Cl_2): +3.7°

2-Hydroxy-1-(4-(methylthio)phenyl)-4-phenylbutane-1,4-dione (10y)

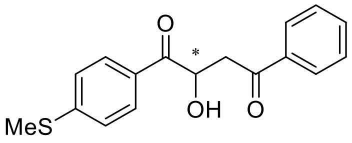

10y

According to the general procedure, (4-(thiomethyl)phenyl)glyoxal (17.8 mg, 90 µmol, 1.0 eq.) and trimethyl((1-phenylvinyl)oxy)silane (34.6 mg, 37 µl, 180 µmol, 2.0 eq.) were reacted using catalyst "*syn*"-**7b^{BArF}** (4.9 mg, 1.8 µmol, 2 mol%) in DCM (0.9 ml, 0.1 M) at -50°C for 16 h, yielding product **10y** after column chromatography as a colorless solid (11.0 mg, 36.6 µmol, 41%). The enantiomeric ratio was determined to be 98.5:1.5 (97% *ee*) by chiral HPLC analysis (CHIRAL ART Amylose-SA (YMC), *n*-heptane/*i*PrOH 93:7, 1 ml/min, 30°C, λ = 240 nm, t_R = 40.2 min (minor), t_R = 42.5 min (major)).

R_f (DCM/EtOAc (5%)): 0.34

^{1}H NMR (300 MHz, DCM-d_2):
δ [ppm] = 7.99 – 7.84 (m, 4H), 7.64 – 7.54 (m, 1H), 7.53 – 7.41 (m, 2H), 7.31 (dt, *J* = 8.6, 1.8 Hz, 2H), 5.58 (td, *J* = 6.2, 4.6 Hz, 1H), 3.92 (d, *J* = 6.3 Hz, 1H), 3.46 – 3.31 (m, 2H), 2.52 (s, 3H).

^{13}C NMR (101 MHz, DCM-d_2):
δ [ppm] = 200.2, 197.7, 148.0, 137.3, 134.0, 130.2, 129.6, 129.2, 128.7, 125.6, 70.4, 44.3, 15.1.

ATR-IR:
$\tilde{\nu}$ [cm^{-1}] = 3497 (w), 3357 (w), 3057 (w), 2914 (w), 1691 (m), 1684 (m), 1673 (vs), 1663 (vs), 1654 (vs), 1646 (w), 1645 (w), 1588 (s), 1576 (w), 1575 (w), 1558 (w), 1555 (w), 1491 (w), 1447 (w), 1437 (w), 1399 (w), 1355 (w), 1322 (w), 1304 (w), 1265 (w), 1237 (w), 1203 (w), 1187 (w), 1107 (w), 1088 (m), 1034 (w), 1002 (w), 998 (w), 977 (w),

931 (w), 881 (w), 827 (m), 773 (w), 753 (w), 747 (m), 735 (m), 688 (s), 675 (w), 588 (w), 567 (w), 563 (w), 556 (w), 548 (w), 545 (w), 536 (w), 521 (w), 481 (w), 479 (w), 475 (w), 473 (w), 418 (w).

HRMS (MALDI-TOF):

m/*z* (+) = calc. 323.0712 $[M+Na]^+$; found 323.0712 $[M+Na]^+$.

$[\alpha]_D^{20}$ (c: 0.26, CH_2Cl_2): +17.1°

_N_-(4-(2-Hydroxy-4-oxo-4-phenylbutanoyl)phenyl)acetamide (10z)

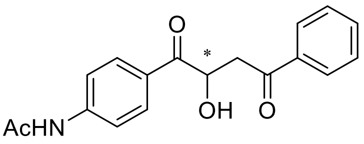

10z

According to the general procedure, (4-(acetamido)phenyl)glyoxal (18.8 mg, 90 µmol, 1.0 eq.) and trimethyl((1-phenylvinyl)oxy)silane (34.6 mg, 37 µl, 180 µmol, 2.0 eq.) were reacted using catalyst "*syn*"-**7b**$^{\mathbf{BArF}}$ (4.9 mg, 1.8 µmol, 2 mol%) in DCM (0.9 ml, 0.1 M) at -50°C for 16 h, yielding product **10z** after column chromatography as a colorless solid (5.5 mg, 17.6 µmol, 20%). The enantiomeric ratio was determined to be 84:16 (68% *ee*) by chiral HPLC analysis (CHIRAL ART Amylose-SA (YMC), *n*-heptane/*i*PrOH 93:7, 1.5 ml/min, 40°C, λ = 240 nm, t_R = 95.6 min (major), t_R = 132.9 min (minor)).

R_f (*n*-pentane/EtOAc (1:2)): 0.22

^{1}H NMR (300 MHz, DCM-*d*₂):
δ [ppm] = 8.01 – 7.87 (m, 4H), 7.71 – 7.41 (m, 6H), 5.62 – 5.55 (m, 1H), 3.92 (d, *J* = 6.3 Hz, 1H), 3.42 – 3.34 (m, 2H), 2.17 (s, 3H).

^{13}C NMR (101 MHz, DCM-d_2):
δ [ppm] = 199.8, 197.7, 169.0, 143.8, 137.3, 134.0, 130.7, 129.4, 129.2, 128.7, 119.4, 70.4, 44.4, 25.1.

ATR-IR:
$\tilde{\nu}$ [cm^{-1}] = 3328 (w), 3266 (w), 3113 (w), 3058 (w), 2927 (w), 2862 (w), 1700 (w), 1695 (m), 1684 (s), 1675 (s), 1662 (vs), 1543 (m), 1525 (s), 1520 (s), 1506 (m), 1448 (w), 1405 (w), 1373 (w), 1368 (w), 1261 (w), 1203 (w), 1176 (m), 1097 (w), 1040 (w), 1002 (m), 926 (w), 883 (w), 835 (w), 749 (m), 686 (m), 676 (w), 674 (w), 638 (w), 581 (w), 508 (w), 502 (w), 418 (w).

HRMS (MALDI-TOF):

m/*z* (+) = calc. 334.1050 $[M+Na]^+$; found 334.1033 $[M+Na]^+$.

$[\alpha]_D^{20}$ (c: 0.14, CH_2Cl_2): +9.8°

Additional data on catalysis experiments

Table S1. Additional results from catalytic reactions using XB donor catalysts.

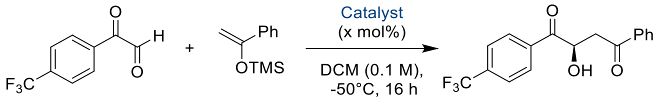

8 **9** **10a**

Entry	Catalyst	Catalyst loading [mol%]	Yield [%][b]	*ee* [%][c]
1	**7b**$^{\mathbf{BArF}}$ (fraction 1)	5	45	94
2	**7b**$^{\mathbf{BArF}}$ (fraction 2)	5	63	94
3	**7c**$^{\mathbf{BArF}}$ (fraction 1)	5	30	89
4	**7c**$^{\mathbf{BArF}}$ (fraction 2)	5	46	91
5	**7d**$^{\mathbf{BArF}}$ (fraction 1)	5	29	81
6	**7d**$^{\mathbf{BArF}}$ (fraction 2)	5	20	78
7	**SI-9**$^{\mathbf{BArF}}$	2	7	*rac.*
8	**SI-10**$^{\mathbf{BArF}}$	2	47	*rac.*

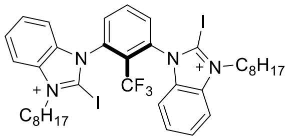

2 $BArF_4^-$

SI-9$^{\mathbf{BArF}}$

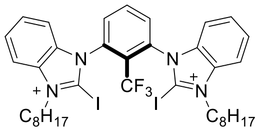

2 $BArF_4^-$

SI-10$^{\mathbf{BArF}}$

As can be seen in the comparison of the different catalyst fractions, the different amounts in atropisomers may lead to significant differences in yield (as different catalyst loadings of the "active" species are present), however, the differences in enantioselectivity observed are slight or negligible. This supports our previously described hypothesis that the preorganized, *syn*-isomers based on such backbone structures[81] are considerably more active and also, that the other atropisomers do not significantly compete as catalysts under suitable conditions.

This could be further supported by the employment of preorganized, achiral XB catalysts **SI-9**$^{\mathbf{BArF}}$ and **SI-10**$^{\mathbf{BArF}}$ in comparison experiments. Herein, the *syn*-preorganized catalyst yielded significantly higher amounts of the (racemic) aldol product **10a** than the *anti*-preorganized system, which exhibited poor activity.

2. Crystallographic data

Crystal structure determination was carried out on a *Rigaku Synergy* dual source device, with Cu micro focus sealed tubes (Cu-K$_{\alpha}$) using mirror monochromators and a *HyPix-6000HE* Hybrid Photon Counting X-ray detector. Crystals were mounted in *Hampton CryoLoops* using *Parabar/Paratone* or *GE/Bayer* silicone grease. Data was recorded and reduced using the *CrysalisPro* Software.[82] Structures were solved using *WinGX*[83] in combination with *ShelXT* and refined with *shelXle* and *ShelXL*.[84,85] Tables for the publication were generated using a modified version of *Ciftab*. Analysis and visualization of the structures was done with *Diamond 4*.[86]

Crystallographic data for 10a

From the asymmetric catalysis experiments using **7b**$^{\mathbf{BArF}}$, a sample of aldol product **10a** was obtained, which upon slow evaporation of $CDCl_3$, gave crystals suitable for analysis by X-ray diffraction.

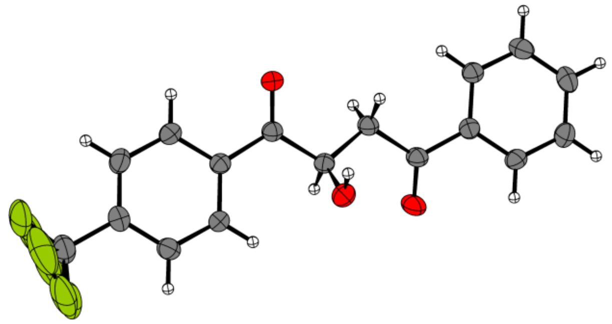

Figure S1. Crystal structure of (*R*)-**10a**. The structure shows disorder in the trifluoromethyl substituent.

Table S2. Crystal data and structure refinement for (*R*)-**10a**.

Compound	(*R*)-**10a**
CCDC number	2364334
Empirical formula	C17 H13 F3 O3
Formula weight [g/mol]	322.27
Crystal system	Orthorhombic
Space group	$P2_12_12_1$ (19)
Lattice parameters [Å]	
a	5.6330(2)
b	7.5988(3)
c	33.1297(11)
α	90
β	90
γ	90
Density [g/cm^3]	1.509
Crystal size [mm^3]	0.410 x 0.270 x 0.140
Volume [Å^3]	1418.08(9)
Z	4
Temperature [K]	170.00(10)
Diffraction Device	XtaLAB Synergy, Dualflex, HyPix
Radiation Type	1.54184 Å (Cu K/ micro-focus sealed X-ray tube)
F(000)	664
Absorption coefficient [mm^{-1}]	1.112
Absorption correction	Gaussian
Measurement range	2.7 - 66.5
Index range	-4 < h < 6
	-8 < k < 9
	-39 < l < 39
Measured reflexes	12274
Independent	2482
Observed	2402
R(int)	0.0411
Completeness (%) / theta (°)	100.0 / 66.497
Transmission (min / max)	0.313 / 1.000
R1 (observed/all)	0.0348 / 0.0356
wR2 (observed/all)	0.0903 / 0.0910
GooF = S	1.055
Rest electron density max./min. [e-/Å^3]	-0.224 / 0.169

Table S3. Atomic coordinates (x 10^4) and equivalent isotropic displacement parameters (Å^{2}x 10^3) for **10a**.

	x	y	z	U(eq)	S.O.F.
C(1A)	0.3388(5)	0.6795(4)	0.28265(7)	0.0439(6)	0.867(3)
F(1A)	0.5174(4)	0.6846(6)	0.25652(6)	0.0815(9)	0.867(3)
F(2A)	0.2018(7)	0.5498(5)	0.26889(7)	0.0872(10)	0.867(3)
F(3A)	0.2200(10)	0.8242(5)	0.27743(7)	0.1134(16)	0.867(3)
C(1B)	0.3388(5)	0.6795(4)	0.28265(7)	0.0439(6)	0.133(3)
F(2B)	0.371(7)	0.555(4)	0.2605(5)	0.1134(16)	0.133(3)
F(1B)	0.427(5)	0.811(4)	0.2686(5)	0.0872(10)	0.133(3)
F(3B)	0.113(3)	0.702(4)	0.2806(4)	0.0815(9)	0.133(3)
O(1)	0.8180(3)	0.4642(3)	0.45223(5)	0.0381(4)	1
O(2)	0.3790(3)	0.7086(2)	0.48745(5)	0.0322(4)	1
O(3)	0.1310(3)	0.4699(3)	0.54407(5)	0.0489(5)	1
C(2)	0.4152(5)	0.6476(3)	0.32536(7)	0.0327(5)	1
C(3)	0.6277(4)	0.5626(3)	0.33301(7)	0.0353(5)	1
C(4)	0.6913(4)	0.5246(3)	0.37239(7)	0.0318(5)	1
C(5)	0.5403(4)	0.5686(3)	0.40437(6)	0.0254(5)	1
C(6)	0.3272(4)	0.6546(3)	0.39622(6)	0.0296(5)	1
C(7)	0.2656(4)	0.6946(3)	0.35683(7)	0.0325(5)	1
C(8)	0.6172(4)	0.5166(3)	0.44609(6)	0.0256(5)	1
C(9)	0.4356(4)	0.5282(3)	0.48060(6)	0.0253(4)	1
C(10)	0.5244(4)	0.4385(3)	0.51862(6)	0.0284(5)	1
C(11)	0.3401(4)	0.4449(3)	0.55189(7)	0.0295(5)	1
C(12)	0.4155(4)	0.4158(3)	0.59447(6)	0.0269(5)	1
C(13)	0.6327(4)	0.3402(3)	0.60447(7)	0.0304(5)	1
C(14)	0.6865(4)	0.3041(3)	0.64457(7)	0.0359(5)	1
C(15)	0.5282(5)	0.3484(3)	0.67488(7)	0.0367(6)	1
C(16)	0.3150(4)	0.4299(3)	0.66523(7)	0.0369(6)	1
C(17)	0.2580(4)	0.4617(3)	0.62526(7)	0.0313(5)	1
H(2)	0.509(5)	0.768(2)	0.4913(9)	0,048	1
H(3)	0,729396	0,530604	0,311346	0,042	1
H(4)	0,838441	0,468236	0,37775	0,038	1
H(6)	0,224087	0,685928	0,417743	0,036	1
H(7)	0,120877	0,754276	0,351342	0,039	1
H(9)	0,287573	0,46674	0,471721	0,03	1
H(10A)	0,563391	0,314288	0,512507	0,034	1
H(10B)	0,671289	0,497336	0,527944	0,034	1
H(13)	0,744304	0,313226	0,58387	0,037	1
H(14)	0,832574	0,248699	0,651209	0,043	1
H(15)	0,565443	0,323178	0,702242	0,044	1
H(16)	0,208439	0,463848	0,686043	0,044	1
H(17)	0,110429	0,515092	0,618716	0,038	1

Table S4. Anisotropic displacement parameters ($Å^2$x 10^3) for **10a**.

	U^{11}	U^{22}	U^{33}	U^{23}	U^{13}	U^{12}
C(1A)	0.0539(16)	0.0466(15)	0.0312(12)	0.0036(11)	-0.0005(12)	0.0029(13)
F(1A)	0.0695(14)	0.144(3)	0.0308(10)	0.0221(14)	0.0100(10)	-0.0002(17)
F(2A)	0.108(2)	0.111(2)	0.0427(12)	0.0059(13)	-0.0291(14)	-0.0469(19)
F(3A)	0.206(4)	0.099(2)	0.0353(11)	0.0067(13)	-0.0141(17)	0.100(3)
C(1B)	0.0539(16)	0.0466(15)	0.0312(12)	0.0036(11)	-0.0005(12)	0.0029(13)
F(2B)	0.206(4)	0.099(2)	0.0353(11)	0.0067(13)	-0.0141(17)	0.100(3)
F(1B)	0.108(2)	0.111(2)	0.0427(12)	0.0059(13)	-0.0291(14)	-0.0469(19)
F(3B)	0.0695(14)	0.144(3)	0.0308(10)	0.0221(14)	0.0100(10)	-0.0002(17)
O(1)	0.0275(8)	0.0563(11)	0.0304(8)	0.0017(7)	0.0004(7)	0.0121(8)
O(2)	0.0333(8)	0.0287(8)	0.0345(8)	-0.0025(6)	0.0024(7)	0.0011(7)
O(3)	0.0224(9)	0.0904(16)	0.0340(9)	0.0140(9)	-0.0006(7)	0.0040(9)
C(2)	0.0385(12)	0.0324(11)	0.0270(11)	0.0016(9)	-0.0015(9)	-0.0029(10)
C(3)	0.0372(13)	0.0400(13)	0.0286(11)	0.0010(10)	0.0072(10)	0.0018(11)
C(4)	0.0269(11)	0.0374(12)	0.0311(12)	0.0014(9)	0.0035(9)	0.0021(10)
C(5)	0.0236(10)	0.0256(10)	0.0271(10)	-0.0010(8)	-0.0001(8)	-0.0018(8)
C(6)	0.0266(11)	0.0356(12)	0.0266(10)	-0.0001(9)	0.0021(9)	0.0039(10)
C(7)	0.0307(11)	0.0344(12)	0.0324(11)	0.0030(10)	-0.0025(9)	0.0024(10)
C(8)	0.0241(10)	0.0255(10)	0.0273(11)	-0.0008(8)	-0.0005(8)	0.0000(9)
C(9)	0.0217(9)	0.0281(10)	0.0262(10)	-0.0014(8)	0.0000(8)	-0.0025(9)
C(10)	0.0231(10)	0.0352(12)	0.0269(10)	0.0046(9)	0.0001(8)	0.0005(9)
C(11)	0.0224(10)	0.0366(12)	0.0295(11)	0.0041(10)	-0.0005(9)	-0.0013(9)
C(12)	0.0248(10)	0.0286(11)	0.0271(11)	0.0009(8)	0.0009(8)	-0.0043(9)
C(13)	0.0251(11)	0.0366(12)	0.0296(11)	0.0016(9)	0.0005(9)	-0.0005(9)
C(14)	0.0288(11)	0.0409(13)	0.0380(12)	0.0057(11)	-0.0057(10)	-0.0009(10)
C(15)	0.0417(14)	0.0429(13)	0.0254(11)	0.0037(10)	-0.0034(10)	-0.0065(11)
C(16)	0.0375(13)	0.0449(14)	0.0284(11)	-0.0008(10)	0.0063(10)	0.0002(11)
C(17)	0.0256(11)	0.0372(13)	0.0311(11)	0.0023(9)	0.0034(9)	0.0017(10)

Crystallographic data for SI-3

Additionally, the intermediate epoxide **SI-3** could be crystallized using vapor diffusion of *n*-heptane into an EtOAc solution of **SI-3**, yielding colorless needles suitable for analysis by X-ray diffraction.

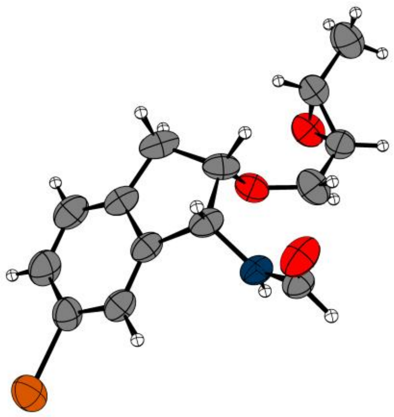

Figure S2. Crystal structure of **SI-3**. The structure shows disorder in the epoxide-containing side chain, which has been omitted in this depiction for the sake of clarity.

Table S5. Crystal data and structure refinement for **SI-3**.

Compound	**SI-3**
CCDC number	2366022
Empirical formula	C14 H16 Br N O3
Formula weight [g/mol]	326.19
Crystal system	Monoclinic
Space group	C2 (5)
Lattice parameters [Å]	
a	35.6233(5)
b	4.54156(7)
c	17.7645(2)
α	90
β	96.9299(13)
γ	90
Density [g/cm3]	1.519
Crystal size [mm3]	0.030 x 0.020 x 0.020
Volume [Å3]	2853.04(7)
Z	8
Temperature [K]	170.00(10)
Diffraction Device	XtaLAB Synergy. Dualflex. HyPix
Radiation Type	1.54184 Å (Cu K/ micro-focus sealed X-ray tube)
F(000)	1328
Absorption coefficient [mm-1]	3.969
Absorption correction	Gaussian
Measurement range	3.3 - 66.5
Index range	-41 < h < 42
	-5 < k < 5
	-21 < l < 21
Measured reflexes	20782
Independent	4963
Observed	4797
R(int)	0.0364
Completeness (%) / theta (°)	99.9 / 66.496
Transmission (min / max)	0.501 / 1.000
R1 (observed/all)	0.0392 / 0.0403
wR2 (observed/all)	0.1038 / 0.1047
GooF = S	1.075
Rest electron density max./min. [e-/Å^3]	-0.417 / 0.546

Table S6. Atomic coordinates (x 10^4) and equivalent isotropic displacement parameters (Å^{2}x 10^3) for **SI-3**.

	x	y	z	U(eq)	S.O.F.
Br(2)	0.43859(2)	0.50981(16)	0.93403(4)	0.0636(2)	1
O(4)	0.61177(12)	1.3341(9)	0.9360(2)	0.0548(10)	1
N(1)	0.71855(15)	0.1045(10)	0.3479(3)	0.0451(10)	1
C(1)	0.74774(15)	0.4876(14)	0.1341(3)	0.0469(11)	1
N(2)	0.58699(12)	0.9024(10)	0.8888(2)	0.0410(9)	1
C(2)	0.71699(16)	0.5149(15)	0.0795(3)	0.0517(12)	1
C(3)	0.68333(17)	0.3749(14)	0.0894(3)	0.0516(14)	1
C(4)	0.68055(15)	0.2094(12)	0.1549(3)	0.0450(11)	1
C(5)	0.71189(15)	0.1881(13)	0.2095(3)	0.0418(10)	1
C(6)	0.74556(15)	0.3253(13)	0.2000(3)	0.0456(12)	1
C(7)	0.64807(17)	0.0371(15)	0.1773(3)	0.0551(14)	1
C(8)	0.65920(16)	-0.0284(12)	0.2622(3)	0.0509(12)	1
C(9)	0.70283(16)	-0.0072(13)	0.2742(3)	0.0463(11)	1
C(10)	0.72976(17)	-0.0628(12)	0.4072(3)	0.0476(13)	1
C(15)	0.46713(14)	0.6435(15)	0.8570(3)	0.0490(12)	1
C(16)	0.45277(17)	0.6041(15)	0.7823(3)	0.0594(16)	1
C(17)	0.47333(18)	0.7016(18)	0.7257(3)	0.0613(15)	1
C(18)	0.50821(17)	0.8306(14)	0.7446(3)	0.0501(12)	1
C(19)	0.52234(15)	0.8680(12)	0.8211(3)	0.0425(11)	1
C(20)	0.50228(15)	0.7766(12)	0.8779(3)	0.0445(12)	1
C(21)	0.56080(14)	1.0149(12)	0.8265(3)	0.0426(10)	1
C(22)	0.57312(17)	0.9550(12)	0.7476(3)	0.0493(13)	1
C(23)	0.53602(19)	0.9458(14)	0.6951(3)	0.0587(15)	1
C(24)	0.60986(14)	1.0659(11)	0.9362(3)	0.0403(11)	1
Br(1)	0.79411(2)	0.67144(19)	0.11873(4)	0.0656(2)	1
O(1)	0.72995(16)	-0.3297(10)	0.4080(2)	0.0734(14)	1
O(2A)	0.64260(13)	0.1901(12)	0.3054(2)	0.0625(10)	0.675(8)
C(11A)	0.6346(3)	0.119(3)	0.3767(5)	0.061(2)	0.675(8)
C(12A)	0.5964(7)	0.206(4)	0.3918(13)	0.058(4)	0.675(8)
C(13A)	0.5870(3)	0.228(2)	0.4678(5)	0.061(2)	0.675(8)
C(14A)	0.5479(4)	0.192(10)	0.4913(19)	0.088(5)	0.675(8)
O(3A)	0.5913(6)	0.495(3)	0.4234(6)	0.060(3)	0.675(8)
O(5A)	0.59026(11)	0.6701(10)	0.7468(2)	0.0535(9)	0.457(8)
O(6A)	0.634(6)	0.35(2)	0.640(5)	0.066(2)	0.457(8)
C(25A)	0.6297(5)	0.633(12)	0.7611(14)	0.060(6)	0.457(8)
C(26A)	0.6520(4)	0.561(3)	0.6980(7)	0.0525(19)	0.457(8)
C(27A)	0.6396(4)	0.653(4)	0.6211(8)	0.059(2)	0.457(8)
C(28A)	0.6668(7)	0.704(8)	0.5633(16)	0.077(4)	0.457(8)
O(2B)	0.64260(13)	0.1901(12)	0.3054(2)	0.0625(10)	0.325(8)
C(11B)	0.6133(6)	0.045(6)	0.3367(11)	0.061(2)	0.325(8)
C(12B)	0.6048(17)	0.169(10)	0.406(3)	0.058(4)	0.325(8)
C(13B)	0.5671(6)	0.219(5)	0.4254(11)	0.061(2)	0.325(8)
C(14B)	0.5593(13)	0.19(2)	0.499(4)	0.088(5)	0.325(8)
O(3B)	0.5900(13)	0.466(7)	0.4036(18)	0.060(3)	0.325(8)
O(5B)	0.59026(11)	0.6701(10)	0.7468(2)	0.0535(9)	0.543(8)
O(6B)	0.634(5)	0.355(19)	0.634(5)	0.066(2)	0.543(8)
C(25B)	0.6291(4)	0.683(10)	0.7420(10)	0.060(6)	0.543(8)
C(26B)	0.6338(3)	0.664(3)	0.6605(7)	0.0525(19)	0.543(8)
C(27B)	0.6672(3)	0.533(3)	0.6345(7)	0.059(2)	0.543(8)
C(28B)	0.6792(6)	0.632(6)	0.5603(13)	0.077(4)	0.543(8)
H(1)	0.720(2)	0.28(2)	0.356(4)	0.068	1
H(4)	0.5904(19)	0.70(2)	0.895(4)	0.061	1
H(2)	0.718839	0.629068	0.035295	0.062	1
H(3)	0.662142	0.391469	0.051679	0.062	1

H(6)	0.766811	0.309341	0.237654	0.055	1
H(7A)	0.644532	-0.147705	0.147714	0.066	1
H(7B)	0.624409	0.153324	0.169299	0.066	1
H(8)	0.650658	-0.229719	0.275306	0.061	1
H(9)	0.713539	-0.207512	0.267156	0.056	1
H(10)	0.73845	0.03477	0.453304	0.057	1
H(16)	0.42897	0.510911	0.769652	0.071	1
H(17)	0.46338	0.679476	0.67399	0.074	1
H(20)	0.511887	0.802621	0.929673	0.053	1
H(21)	0.557661	1.231799	0.833101	0.051	1
H(22)	0.590308	1.112665	0.732448	0.059	1
H(23A)	0.528742	1.144724	0.675733	0.07	1
H(23B)	0.537932	0.812784	0.651525	0.07	1
H(24)	0.626253	0.964902	0.973762	0.048	1
H(11A)	0.65343	0.215577	0.414387	0.074	0.675(8)
H(11B)	0.637413	-0.09654	0.383797	0.074	0.675(8)
H(12A)	0.575422	0.13851	0.353209	0.07	0.675(8)
H(13A)	0.608297	0.186359	0.508447	0.073	0.675(8)
H(14A)	0.52896	0.223967	0.447325	0.132	0.675(8)
H(14B)	0.544259	0.335757	0.530847	0.132	0.675(8)
H(14C)	0.54518	-0.00766	0.510969	0.132	0.675(8)
H(25A)	0.634564	0.475468	0.799589	0.073	0.457(8)
H(25B)	0.640336	0.817044	0.784893	0.073	0.457(8)
H(26A)	0.679862	0.539096	0.711919	0.063	0.457(8)
H(27A)	0.61628	0.77728	0.614486	0.071	0.457(8)
H(28A)	0.655329	0.634245	0.513566	0.116	0.457(8)
H(28B)	0.690271	0.595745	0.578416	0.116	0.457(8)
H(28C)	0.672305	0.914753	0.560461	0.116	0.457(8)
H(11C)	0.620539	-0.164026	0.345519	0.074	0.325(8)
H(11D)	0.590276	0.049095	0.299479	0.074	0.325(8)
H(12B)	0.624088	0.127174	0.450061	0.07	0.325(8)
H(13B)	0.546205	0.157436	0.385863	0.073	0.325(8)
H(14D)	0.555961	-0.020308	0.510011	0.132	0.325(8)
H(14E)	0.535988	0.29589	0.505265	0.132	0.325(8)
H(14F)	0.58024	0.268946	0.533527	0.132	0.325(8)
H(25C)	0.642279	0.517759	0.770244	0.073	0.543(8)
H(25D)	0.639751	0.870531	0.7637	0.073	0.543(8)
H(26B)	0.619351	0.80909	0.625903	0.063	0.543(8)
H(27B)	0.688213	0.48638	0.675149	0.071	0.543(8)
H(28D)	0.656851	0.662324	0.523338	0.116	0.543(8)
H(28E)	0.69538	0.480713	0.541445	0.116	0.543(8)
H(28F)	0.693386	0.816761	0.567775	0.116	0.543(8)

Table S7. Anisotropic displacement parameters ($Å^2$x 10^3) for **SI-3**.

	U^{11}	U^{22}	U^{33}	U^{23}	U^{13}	U^{12}
Br(2)	0.0529(3)	0.0801(5)	0.0581(3)	-0.0132(3)	0.0084(2)	-0.0169(3)
O(4)	0.067(2)	0.030(2)	0.062(2)	0.0024(18)	-0.0153(18)	0.0006(18)
N(1)	0.069(3)	0.028(2)	0.038(2)	-0.0036(16)	0.0039(19)	0.0048(17)
C(1)	0.054(3)	0.047(3)	0.040(2)	-0.003(2)	0.009(2)	0.010(3)
N(2)	0.051(2)	0.025(2)	0.045(2)	0.0062(16)	-0.0008(18)	-0.0018(16)
C(2)	0.068(3)	0.049(3)	0.038(2)	-0.001(3)	0.005(2)	0.018(3)
C(3)	0.066(3)	0.051(3)	0.034(3)	-0.003(2)	-0.008(2)	0.017(3)
C(4)	0.057(3)	0.033(3)	0.043(3)	-0.009(2)	-0.002(2)	0.011(2)
C(5)	0.057(3)	0.031(2)	0.037(2)	-0.003(2)	0.003(2)	0.015(2)
C(6)	0.053(3)	0.042(3)	0.041(3)	-0.001(2)	0.002(2)	0.015(2)
C(7)	0.061(3)	0.045(3)	0.057(3)	-0.017(3)	-0.004(2)	0.008(3)

C(8)	0.066(3)	0.027(3)	0.060(3)	-0.005(2)	0.005(3)	-0.005(2)
C(9)	0.069(3)	0.026(2)	0.042(2)	-0.002(2)	0.000(2)	0.007(2)
C(10)	0.070(3)	0.033(3)	0.038(3)	-0.002(2)	0.000(2)	0.001(2)
C(15)	0.042(2)	0.055(3)	0.049(3)	-0.009(3)	0.001(2)	-0.003(2)
C(16)	0.052(3)	0.070(5)	0.052(3)	-0.011(3)	-0.009(2)	-0.007(3)
C(17)	0.067(3)	0.069(4)	0.043(3)	-0.006(3)	-0.014(2)	0.000(3)
C(18)	0.063(3)	0.047(3)	0.038(3)	0.002(2)	-0.005(2)	0.004(3)
C(19)	0.051(3)	0.032(3)	0.042(3)	0.002(2)	-0.005(2)	0.002(2)
C(20)	0.050(3)	0.042(3)	0.039(2)	-0.006(2)	-0.005(2)	0.001(2)
C(21)	0.057(3)	0.027(2)	0.041(2)	0.003(2)	-0.004(2)	-0.004(2)
C(22)	0.069(3)	0.034(3)	0.046(3)	0.009(2)	0.010(2)	-0.007(2)
C(23)	0.084(4)	0.050(4)	0.040(3)	0.008(2)	-0.002(3)	-0.001(3)
C(24)	0.045(3)	0.032(3)	0.042(3)	0.0060(19)	0.000(2)	0.0000(19)
Br(1)	0.0636(4)	0.0746(4)	0.0609(4)	0.0090(4)	0.0170(3)	0.0061(3)
O(1)	0.137(4)	0.028(2)	0.048(2)	-0.0002(19)	-0.019(2)	0.002(3)
O(2A)	0.074(3)	0.056(2)	0.063(2)	-0.008(2)	0.028(2)	-0.009(2)
C(11A)	0.060(5)	0.073(6)	0.052(5)	0.019(4)	0.012(3)	0.007(4)
C(12A)	0.065(13)	0.052(7)	0.059(11)	0.010(6)	0.014(7)	0.002(6)
C(13A)	0.058(5)	0.072(6)	0.052(5)	0.009(4)	0.008(3)	0.000(4)
C(14A)	0.049(12)	0.127(8)	0.093(9)	0.020(8)	0.033(11)	0.005(16)
O(3A)	0.074(3)	0.062(4)	0.044(7)	0.000(5)	0.009(6)	0.003(3)
O(5A)	0.061(2)	0.043(2)	0.058(2)	0.003(2)	0.0137(17)	-0.009(2)
O(6A)	0.070(3)	0.056(3)	0.073(10)	-0.011(6)	0.013(10)	-0.011(2)
C(25A)	0.059(4)	0.051(14)	0.073(10)	-0.011(12)	0.015(5)	-0.014(4)
C(26A)	0.052(5)	0.049(5)	0.056(5)	0.006(4)	0.004(4)	-0.004(4)
C(27A)	0.051(4)	0.063(6)	0.064(5)	0.006(5)	0.010(4)	-0.003(5)
C(28A)	0.062(14)	0.093(14)	0.082(6)	0.002(8)	0.030(8)	0.006(8)
O(2B)	0.074(3)	0.056(2)	0.063(2)	-0.008(2)	0.028(2)	-0.009(2)
C(11B)	0.060(5)	0.073(6)	0.052(5)	0.019(4)	0.012(3)	0.007(4)
C(12B)	0.065(13)	0.052(7)	0.059(11)	0.010(6)	0.014(7)	0.002(6)
C(13B)	0.058(5)	0.072(6)	0.052(5)	0.009(4)	0.008(3)	0.000(4)
C(14B)	0.049(12)	0.127(8)	0.093(9)	0.020(8)	0.033(11)	0.005(16)
O(3B)	0.074(3)	0.062(4)	0.044(7)	0.000(5)	0.009(6)	0.003(3)
O(5B)	0.061(2)	0.043(2)	0.058(2)	0.003(2)	0.0137(17)	-0.009(2)
O(6B)	0.070(3)	0.056(3)	0.073(10)	-0.011(6)	0.013(10)	-0.011(2)
C(25B)	0.059(4)	0.051(14)	0.073(10)	-0.011(12)	0.015(5)	-0.014(4)
C(26B)	0.052(5)	0.049(5)	0.056(5)	0.006(4)	0.004(4)	-0.004(4)
C(27B)	0.051(4)	0.063(6)	0.064(5)	0.006(5)	0.010(4)	-0.003(5)
C(28B)	0.062(14)	0.093(14)	0.082(6)	0.002(8)	0.030(8)	0.006(8)

Crystallographic data for 3a

The minor diastereomer of intermediate methyl ketone **3** could be crystallized using vapor diffusion of *n*-heptane into an acetone solution of **3a**, yielding colorless needles suitable for analysis by X-ray diffraction.

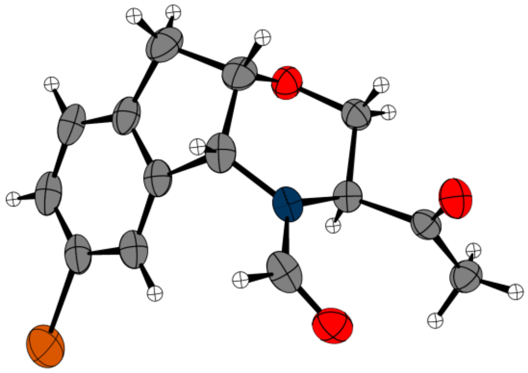

Figure S3. Crystal structure of **3a**.

Table S8. Crystal data and structure refinement for **3a**.

Compound	**3a**
CCDC number	2366021
Empirical formula	C14 H14 Br N O3
Formula weight [g/mol]	324.17
Crystal system	Orthorhombic
Space group	$P2_12_12_1$ (19)
Lattice parameters [Å]	
a	4.85650(10)
b	14.5869(2)
c	19.1323(3)
α	90
β	90
γ	90
Density [g/cm^3]	1.589
Crystal size [mm^3]	0.460 x 0.080 x 0.050
Volume [Å^3]	1355.36(4)
Z	4
Temperature [K]	170.01(10)
Diffraction Device	XtaLAB Synergy. Dualflex. HyPix
Radiation Type	1.54184 Å (Cu K/ micro-focus sealed X-ray tube)
F(000)	656
Absorption coefficient [mm^{-1}]	4.177
Absorption correction	Gaussian
Measurement range	3.8 - 66.4
Index range	-5 < h < 4
	-17 < k < 17
	-22 < l < 22
Measured reflexes	7119
Independent	2389
Observed	2277
R(int)	0.0338
Completeness (%) / theta (°)	100.1 / 66.355
Transmission (min / max)	0.475 / 1.000
R1 (observed/all)	0.0296 / 0.0309
wR2 (observed/all)	0.0954 / 0.0973
GooF = S	0.819
Rest electron density max./min. [e-/Å^3]	-1.40960452

Table S9. Atomic coordinates (x 10^4) and equivalent isotropic displacement parameters ($Å^2$x 10^3) for **3a**.

	x	y	z	U(eq)
Br(1)	0.73937(12)	0.56858(3)	0.05669(2)	0.0674(2)
O(1)	0.5676(5)	0.86341(17)	0.31820(13)	0.0369(5)
O(2)	0.0442(4)	0.70582(18)	0.44674(12)	0.0367(5)
O(3)	0.0380(5)	0.57687(18)	0.32520(14)	0.0425(6)
N(1)	0.1956(5)	0.72017(18)	0.30076(14)	0.0292(5)
C(1)	0.6571(7)	0.6817(3)	0.10051(17)	0.0404(8)
C(2)	0.7881(7)	0.7606(3)	0.07723(18)	0.0433(8)
C(3)	0.7323(7)	0.8445(3)	0.10878(17)	0.0414(8)
C(4)	0.5443(7)	0.8472(2)	0.16376(17)	0.0355(7)
C(5)	0.4549(8)	0.9277(3)	0.2079(2)	0.0442(8)
C(6)	0.3391(7)	0.8810(2)	0.2732(2)	0.0373(7)
C(7)	0.2234(6)	0.7902(2)	0.24635(17)	0.0335(7)
C(8)	0.4160(6)	0.7673(2)	0.18649(17)	0.0313(7)
C(9)	0.4678(7)	0.6835(3)	0.15538(17)	0.0356(7)
C(10)	0.4815(7)	0.8141(2)	0.37815(17)	0.0346(7)
C(11)	0.3922(5)	0.7170(2)	0.35902(16)	0.0267(6)
C(12)	0.2579(6)	0.67453(19)	0.42435(16)	0.0280(6)
C(13)	0.4185(7)	0.6011(2)	0.46046(19)	0.0349(7)
C(14)	0.0396(6)	0.6458(3)	0.28876(18)	0.0369(7)
H(2)	0.915986	0.757351	0.039739	0.052
H(3)	0.820582	0.898923	0.093143	0.05
H(5A)	0.312058	0.96446	0.183865	0.053
H(5B)	0.612846	0.967771	0.219609	0.053
H(6)	0.194833	0.919146	0.296579	0.045
H(7)	0.036502	0.802177	0.226393	0.04
H(9)	0.378062	0.62931	0.170764	0.043
H(10A)	0.325925	0.846598	0.40057	0.041
H(10B)	0.634774	0.811162	0.412184	0.041
H(11)	0.556892	0.680073	0.345212	0.032
H(13A)	0.313941	0.578605	0.500768	0.052
H(13B)	0.594977	0.626135	0.476388	0.052
H(13C)	0.45201	0.550446	0.427916	0.052
H(14)	-0.078033	0.646719	0.249091	0.044

Table S10. Anisotropic displacement parameters ($Å^2$x 10^3) for **3a**.

	U^{11}	U^{22}	U^{33}	U^{23}	U^{13}	U^{12}
Br(1)	0.0938(4)	0.0627(3)	0.0458(3)	-0.00961(19)	0.0210(2)	0.0188(3)
O(1)	0.0392(11)	0.0381(12)	0.0334(12)	0.0024(11)	0.0018(10)	-0.0063(10)
O(2)	0.0265(11)	0.0491(12)	0.0345(11)	-0.0015(11)	0.0071(9)	0.0046(9)
O(3)	0.0373(11)	0.0418(13)	0.0483(14)	-0.0059(12)	0.0025(11)	-0.0107(11)
N(1)	0.0204(11)	0.0394(13)	0.0278(12)	-0.0028(10)	0.0002(9)	0.0004(9)
C(1)	0.0424(17)	0.054(2)	0.0244(15)	0.0004(15)	0.0008(13)	0.0144(15)
C(2)	0.0357(16)	0.067(2)	0.0271(15)	0.0094(16)	0.0039(14)	0.0132(17)
C(3)	0.0359(16)	0.0569(19)	0.0313(15)	0.0159(15)	0.0006(14)	0.0062(16)
C(4)	0.0307(14)	0.0466(18)	0.0292(15)	0.0099(15)	-0.0029(13)	0.0105(14)
C(5)	0.0460(18)	0.0389(18)	0.048(2)	0.0118(17)	0.0055(16)	0.0122(15)
C(6)	0.0353(15)	0.0353(16)	0.0412(19)	0.0035(14)	0.0057(14)	0.0142(13)
C(7)	0.0222(13)	0.0488(17)	0.0295(14)	0.0030(14)	-0.0007(12)	0.0105(14)
C(8)	0.0224(13)	0.0453(18)	0.0262(14)	0.0025(14)	-0.0037(12)	0.0057(12)
C(9)	0.0311(14)	0.0486(18)	0.0271(15)	0.0006(14)	-0.0006(13)	0.0053(14)
C(10)	0.0407(17)	0.0340(15)	0.0290(15)	-0.0034(13)	0.0003(13)	-0.0065(13)
C(11)	0.0200(12)	0.0327(15)	0.0274(14)	0.0006(12)	-0.0011(11)	0.0009(10)
C(12)	0.0259(13)	0.0298(13)	0.0284(13)	-0.0039(11)	-0.0002(12)	-0.0013(11)
C(13)	0.0360(15)	0.0339(15)	0.0349(16)	0.0029(14)	-0.0003(13)	0.0016(13)
C(14)	0.0208(13)	0.055(2)	0.0346(16)	-0.0111(17)	0.0030(12)	-0.0039(13)

3. Computational Details

To ensure that the lowest conformers were used in the calculations, conformational sampling was performed using xTB 6.5.1[87-90] and CREST 2.12.[69,70] The 10 most stable conformers, according to CREST (with GFN2-xTB energies), for each diastereomeric transition state, were remodelled at the M06-2X/def2-TZVP (def2-TZVPD for I)//M06-2X/def2-SVP (def2-SVPD for I) level. This was performed to be certain to truly identify the most favored conformers. Only two conformers per diastereomeric transition state proved significant (i.e. Boltzmann weight above 1%). Thus, for the sake of simplicity, only the two lowest energy conformers of each transition state, leading to *(R)* and *(S)* products, respectively, were used and are provided.

The geometry optimizations were performed using the ORCA 6.0.0 software package[91] with the M06-2X[92] density functional, in combination with the def2-SVP[93] basis set for all atoms except iodine, for which def2-SVPD basis set and related pseudopotential were applied.[94] The denser integration grid DefGrid3 was used throughout to improve results quality. The structures were optimized using the SMD[95] solvation model for dichloromethane, using the optimized radii for iodine.[71] Harmonic vibrational frequencies were computed for all optimized structures to verify that they were transition states, possessing one imaginary frequency. To further improve the free energies computed, single point energy calculations were performed on the optimized structures with the M06-2X density functional, in combination with the def2-TZVP basis set for all atoms except iodine, for which def2-TZVPD basis set and related pseudopotential were applied, including SMD solvation for dichloromethane, using the parameters described above. The free energies were calculated from the unscaled harmonic vibrational frequencies and include the vibrational entropy correction scheme for low-lying (< 100 cm^{-1}) vibrational modes described by Grimme.[96] All thermodynamic corrections were calculated for a state of 223 K (-50 °C) and 1 mol/L.

The structures of the optimized geometries are provided as a zip file (Optimized_Structures.zip)

Table S11. Computed energetic values.

Structure	SCF_{TZVP}	SCF_{SVP}	G_{corr} (1M)	G_{TZVP} (1M)	$Ratio_{223K}$		ΔG_{223K}	$\Delta\Delta G^{‡}_{223K}$	*e.r.*	*ee*
	Ha	*Ha*	*Ha*	*Ha*	%	*Ha*	*kJ/mol*	*kJ/mol*	X:1	%
**8**	-795.975923	-795.055117	0.101781	-795.874142						
**9**	-793.555339	-792.884612	0.213567	-793.341772						
**7b**	-3328.963728	-3325.881363	0.629702	-3328.334026						
**7b•8**	-4124.959758	-4120.974135	0.751485	-4124.208273			**-0.3**			
**TS1-uncat**	-1589.513838	-1587.927364	0.317434	-1589.196404			**51.2**			
**TS2-uncat**	-1589.513855	-1587.927403	0.318219	-1589.195636			**53.2**			
**TS-1*R***	-4918.525665	-4913.875988	0.987446	-4917.538220	**79%**	-3884.769901	31.0			
**TS-2*R***	-4918.522546	-4913.871431	0.985428	-4917.537118	**21%**	-1032.768087	33.9			
					Boltz. avg	**-4917.537988**	**31.4**			
								**3.5**	**6.6**	**85%**
**TS-1*S***	-4918.519672	-4913.867994	0.982799	-4917.536872	**83%**	-4073.111821	34.6			
**TS-2*S***	-4918.522761	-4913.874504	0.987132	-4917.535628	**17%**	-844.424837	37.9			
					Boltz. avg	**-4917.536659**	**34.9**			

TS1-uncat: Transition state for the uncatalyzed aldol reaction, lowest conformation found.
TS2-uncat: Transition state for the uncatalyzed aldol reaction, optimized from **TS-1*R* -** TS **8**···**9** fragment.

Table S12. Computed Distortion/Interaction values for **TS-1*R*** and **TS-1*S***.

Structure	SCF_{TZVP}	Property	Absolute values	Property	Relative values
	Ha		*kJ/mol*		*kJ/mol*
**TS-1*R***	-4918.525665				
**TS-1*R*** - Catalyst **7b** fragment	-3328.962149	**$\Delta E_{Dist(Cat)}$**	**4.1**		
**TS-1*R*** - TS **8**···**9** fragment	-1589.519890	**$\Delta E_{Dist(TS)}$**	**29.9**		
		**ΔE_{Int}**	**-114.5**		
**TS-1*S***	-4918.519672			**ΔΔE**	**-15.7**
**TS-1*S*** - Catalyst **7b** fragment	-3328.960441	**$\Delta E_{Dist(Cat)}$**	**8.6**	**$\Delta\Delta E_{Dist(Cat)}$**	**-4.5**
**TS-1*S*** - TS **8**···**9** fragment	-1589.512117	**$\Delta E_{Dist(TS)}$**	**50.3**	**$\Delta\Delta E_{Dist(TS)}$**	**-20.4**
		**ΔE_{Int}**	**-123.7**	**$\Delta\Delta E_{Int}$**	**9.2**

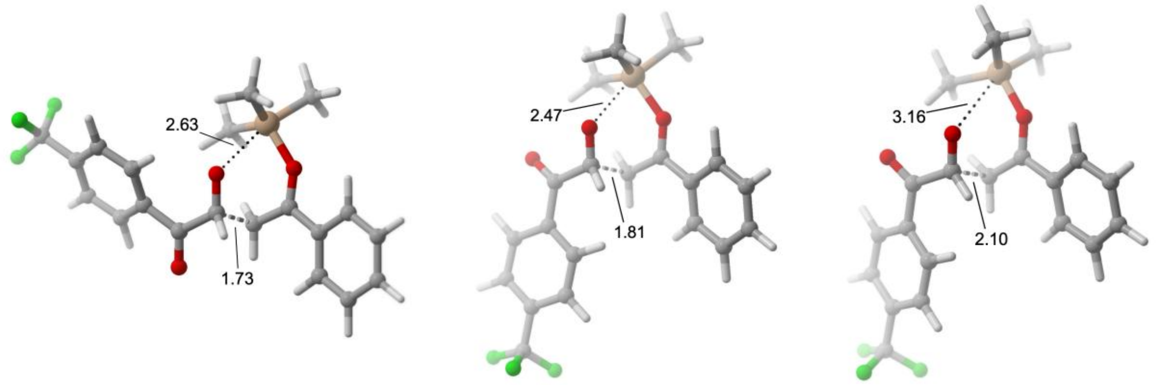

TS1-uncat **TS2-uncat** **TS-1*R*** - TS **8**···**9** fragment

Figure S4. Transition structures for the uncatalyzed aldol reaction.

Evaluation of different theory models

The evaluation of the different theory models shows good consistency in reproducing the selectivities and the preference for the main conformer of each transition structure, including with a range-separated hybrid functional (ωB97X-V)[97] and one of the best-performing double-hybrid functional (revDSD-PBEP86-D3(BJ)).[98]

Table S13. Evaluation of different theory models (single-point calculations on the geometries obtained with M06-2X/def2-SVP (def2-SVPD for I) with SMD(CH_2Cl_2); see Table S14 for details on the models)

TS	$SCF_{Method\ \#}$	SCF_{SVP}	G_{corr} (1M)	G_{TZVP} (1M)	Ratio(223K)		$\Delta\Delta G^{\ddagger}_{223K}$	*e.r.*	*ee*
	Ha	*Ha*	*Ha*	*Ha*	*%*	*Ha*	*kJ/mol*	*X:1*	*%*
**TS-1*R***	-4918.525665	-4913.875988	0.987446	-4917.538220	**79%**	-3884.769901			
**TS-2*R***	-4918.522546	-4913.871431	0.985428	-4917.537118	**21%**	-1032.768087			
					Boltz. avg	**-4917.537988**			
							**3.5**	**6.6**	**85%**
**TS-1*S***	-4918.519672	-4913.867994	0.982799	-4917.536872	**83%**	-4073.111821			
**TS-2*S***	-4918.522761	-4913.874504	0.987132	-4917.535628	**17%**	-844.424837			
**Method 1**					*Boltz. avg*	**-4917.536659**			
**TS-1*R***	-4918.546065	-4913.875988	0.987446	-4917.558619	**81%**	-3982.869183			
**TS-2*R***	-4918.542933	-4913.871431	0.985428	-4917.557505	**19%**	-934.689224			
					Boltz. avg	**-4917.558407**			
							**3.1**	**5.2**	**81%**
**TS-1*S***	-4918.540219	-4913.867994	0.982799	-4917.557420	**87%**	-4288.052814			
**TS-2*S***	-4918.543126	-4913.874504	0.987132	-4917.555994	**13%**	-629.504423			
**Method 2**					*Boltz. avg*	**-4917.557237**			

**TS-1$_R$**	-4918.853217	-4913.875988	0.987446	-4917.865772	**81%**	-4000.529031			
**TS-2$_R$**	-4918.850160	-4913.871431	0.985428	-4917.864732	**19%**	-917.336547			
					Boltz. avg	**-4917.865578**			
							**1.3**	**2.0**	**51%**
**TS-1$_S$**	-4918.847962	-4913.867994	0.982799	-4917.865163	**96%**	-4743.080228			
**TS-2$_S$**	-4918.849964	-4913.874504	0.987132	-4917.862832	**4%**	-174.784852			
**Method 3**					*Boltz. avg*	**-4917.865080**			
**TS-1$_R$**	-4919.040394	-4913.875988	0.987446	-4918.052948	**85%**	-4190.627715			
**TS-2$_R$**	-4919.037140	-4913.871431	0.985428	-4918.051712	**15%**	-727.425051			
					Boltz. avg	**-4918.052765**			
							**3.5**	**6.7**	**85%**
**TS-1$_S$**	-4919.034321	-4913.867994	0.982799	-4918.051522	**58%**	-2876.272561			
**TS-2$_S$**	-4919.038412	-4913.874504	0.987132	-4918.051280	**42%**	-2041.778861			
**Method 4**					*Boltz. avg*	**-4918.051421**			
**TS-1$_R$**	-4912.387783	-4913.875988	0.987446	-4911.400337	**80%**	-3943.462819			
**TS-2$_R$**	-4912.384773	-4913.871431	0.985428	-4911.399345	**20%**	-967.937322			
					Boltz. avg	**-4911.400142**			
							**1.6**	**2.3**	**57%**
**TS-1$_S$**	-4912.382536	-4913.867994	0.982799	-4911.399736	**82%**	-4013.950797			
**TS-2$_S$**	-4912.385811	-4913.874504	0.987132	-4911.398678	**18%**	-897.448746			
**Method 5**					*Boltz. avg*	**-4911.399543**			

Table S14. Summary of the evaluation of the different theory models

**Method**	**Functional**	**Basis Set**	**$\Delta\Delta G^{\ddagger}_{223K}$ (kJ/mol)**	***ee* (%)**
**1**	M06-2X	def2-TZVP + def2-TZVPD(I)	3.5	85 (*R*)
**2**	M06-2X	def2-TZVPP + def2-TZVPPD(I)	3.1	81 (*R*)
**3**	M06-2X	def2-QZVP + def2-QZVPD(I)	1.3	51 (*R*)
**4**	ωB97X-V	def2-TZVP + def2-TZVPD(I)	3.5	85 (*R*)
**5**	revDSD-PBEP86-D3(BJ)	def2-TZVPP + def2-TZVPPD(I)	1.6	57 (*R*)

4. HPLC chromatograms

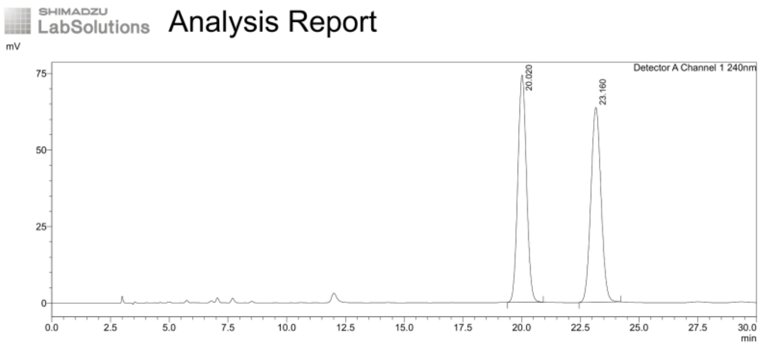

Detector A Channel 1 240nm

Peak#	Ret. Time	Area	Height	Area%
1	20.020	1906408	74185	49.963
2	23.160	1909223	63628	50.037
Total		3815631	137813	100.000

Figure S5. HPLC chromatogram of product **10a** (racemic synthesis).

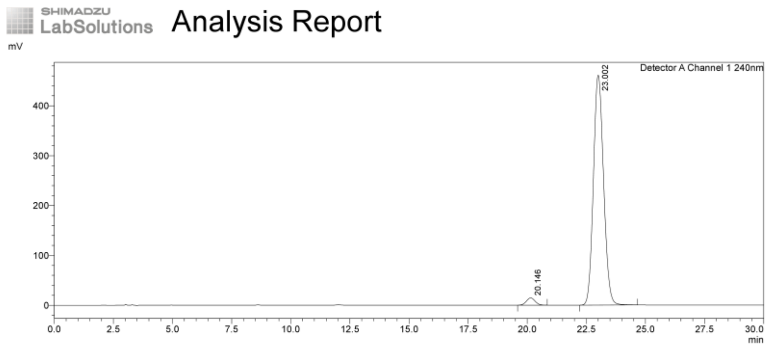

Detector A Channel 1 240nm

Peak#	Ret. Time	Area	Height	Area%
1	20.146	369642	14541	2.606
2	23.002	13813233	461066	97.394
Total		14182875	475608	100.000

Figure S6. HPLC chromatogram of product **10a** (enantioselective synthesis).

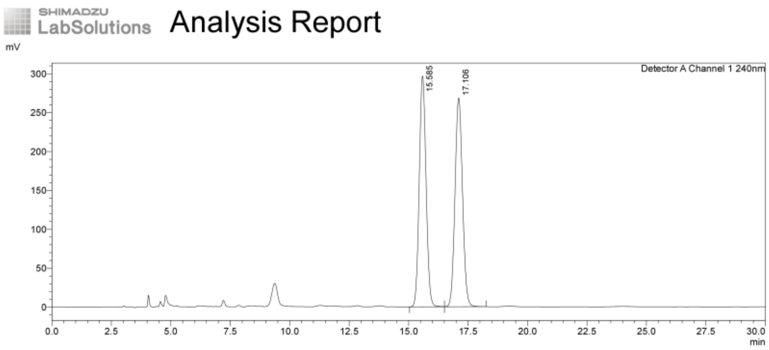

Detector A Channel 1 240nm

Peak#	Ret. Time	Area	Height	Area%
1	15.585	5920278	296696	50.035
2	17.106	5912022	268432	49.965
Total		11832300	565128	100.000

Figure S7. HPLC chromatogram of product **10b** (racemic synthesis).

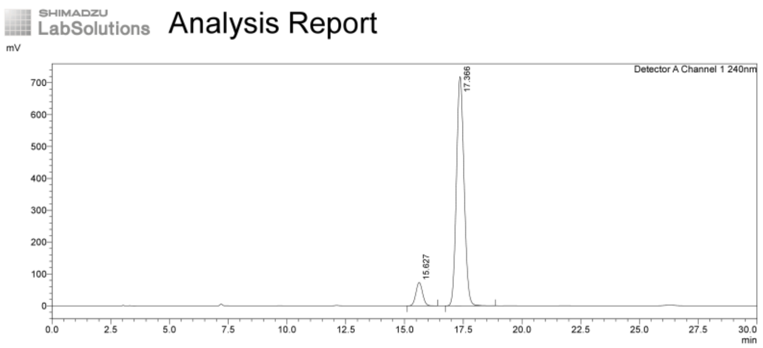

Detector A Channel 1 240nm

Peak#	Ret. Time	Area	Height	Area%
1	15.627	1456640	73419	8.267
2	17.366	16163680	719058	91.733
Total		17620320	792477	100.000

Figure S8. HPLC chromatogram of product **10b** (enantioselective synthesis).

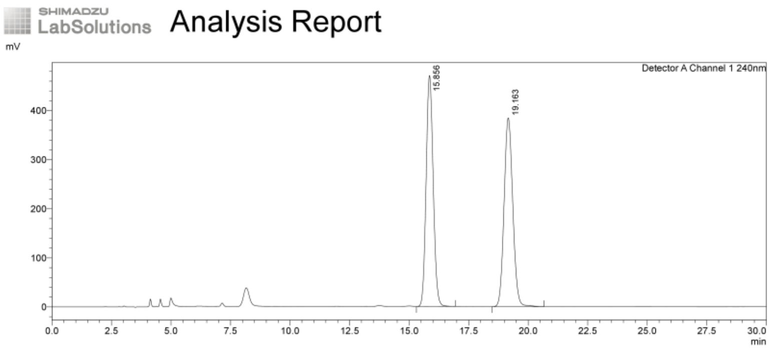

Detector A Channel 1 240nm

Peak#	Ret. Time	Area	Height	Area%
1	15.856	9710289	470587	49.853
2	19.163	9767358	384473	50.147
Total		19477647	855060	100.000

Figure S9. HPLC chromatogram of product **10c** (racemic synthesis).

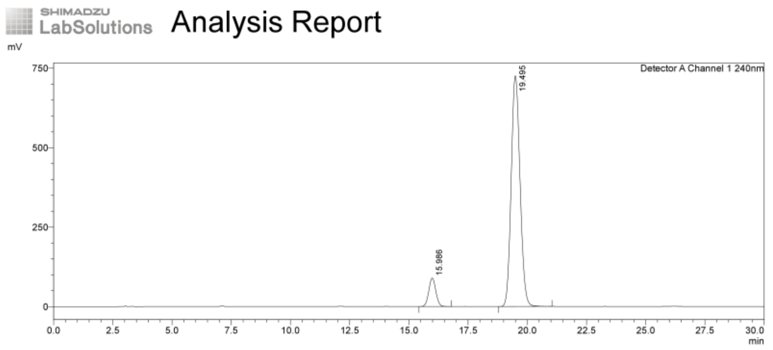

Detector A Channel 1 240nm

Peak#	Ret. Time	Area	Height	Area%
1	15.986	1851753	89577	8.960
2	19.495	18816212	725928	91.040
Total		20667965	815505	100.000

Figure S10. HPLC chromatogram of product **10c** (enantioselective synthesis).

SHIMADZU LabSolutions **Analysis Report**

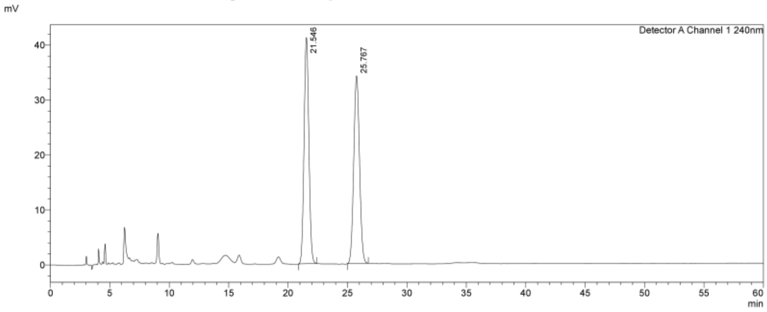

Detector A Channel 1 240nm

Peak#	Ret. Time	Area	Height	Area%
1	21.546	1149507	41163	50.049
2	25.767	1147260	34164	49.951
Total		2296768	75327	100.000

Figure S11. HPLC chromatogram of product **10d** (racemic synthesis).

SHIMADZU LabSolutions **Analysis Report**

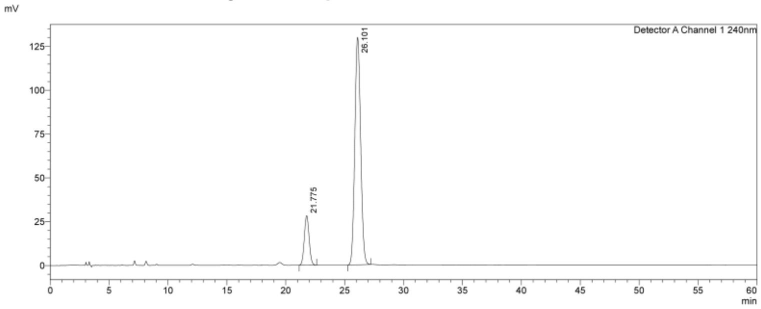

Detector A Channel 1 240nm

Peak#	Ret. Time	Area	Height	Area%
1	21.775	796325	28237	15.176
2	26.101	4450859	129776	84.824
Total		5247184	158013	100.000

Figure S12. HPLC chromatogram of product **10d** (enantioselective synthesis).

SHIMADZU LabSolutions **Analysis Report**

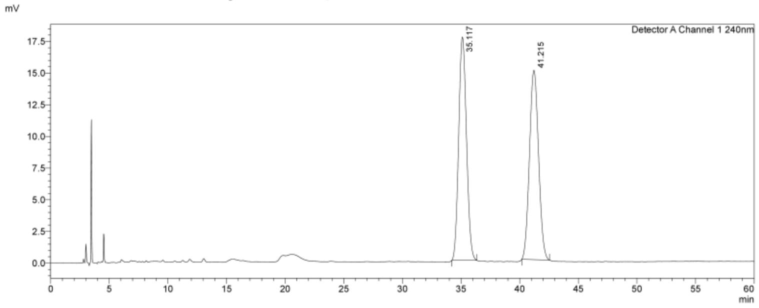

Detector A Channel 1 240nm

Peak#	Ret. Time	Area	Height	Area%
1	35.117	813867	17630	50.256
2	41.215	805584	14949	49.744
Total		1619451	32578	100.000

Figure S13. HPLC chromatogram of product **10e** (racemic synthesis).

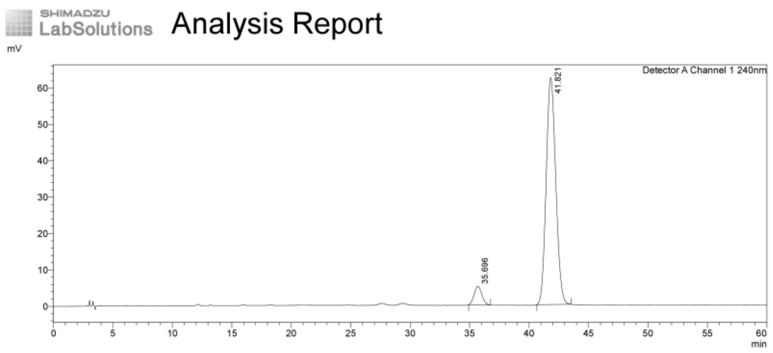

Detector A Channel 1 240nm

Peak#	Ret. Time	Area	Height	Area%
1	35.696	232208	5092	6.262
2	41.821	3476112	62390	93.738
Total		3708320	67482	100.000

Figure S14. HPLC chromatogram of product **10e** (enantioselective synthesis).

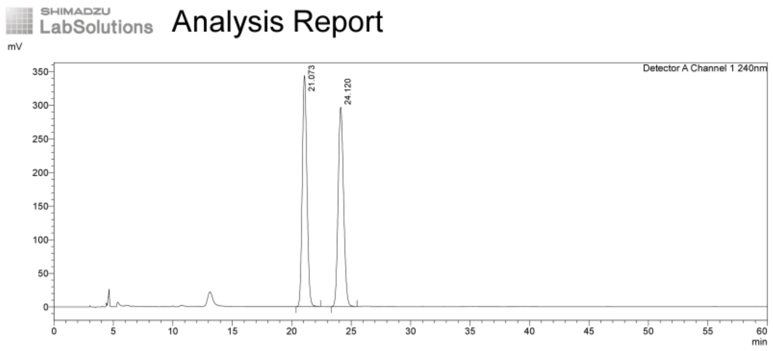

Detector A Channel 1 240nm

Peak#	Ret. Time	Area	Height	Area%
1	21.073	9399394	343519	49.984
2	24.120	9405243	296504	50.016
Total		18804637	640023	100.000

Figure S15. HPLC chromatogram of product **10f** (racemic synthesis).

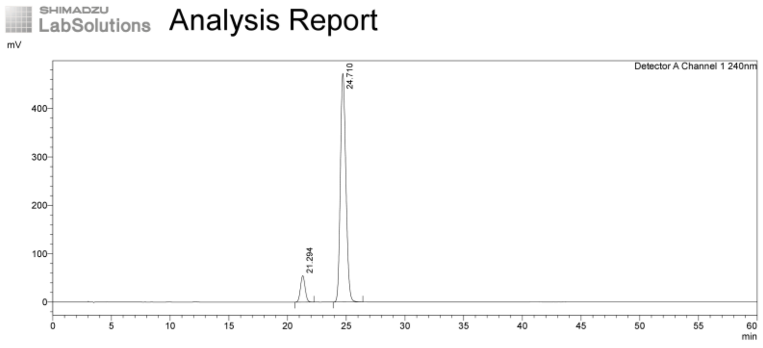

Detector A Channel 1 240nm

Peak#	Ret. Time	Area	Height	Area%
1	21.294	1493323	54561	8.764
2	24.710	15545531	472383	91.236
Total		17038854	526944	100.000

Figure S16. HPLC chromatogram of product **10f** (enantioselective synthesis).

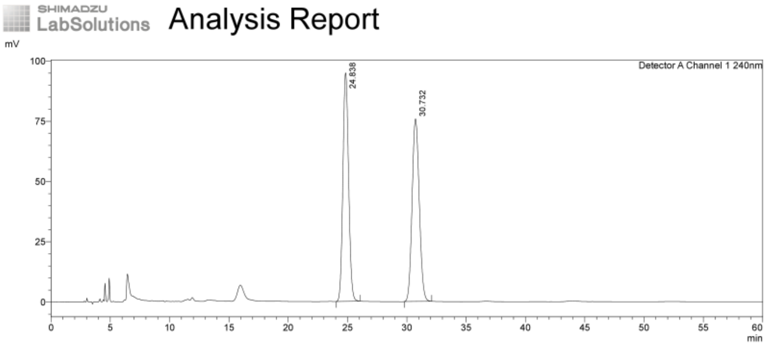

Detector A Channel 1 240nm

Peak#	Ret. Time	Area	Height	Area%
1	24.838	3158071	94759	49.996
2	30.732	3158629	75709	50.004
Total		6316700	170467	100.000

Figure S17. HPLC chromatogram of product **10g** (racemic synthesis).

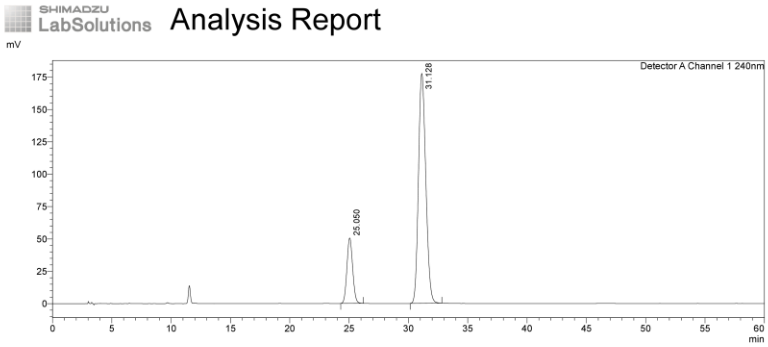

Detector A Channel 1 240nm

Peak#	Ret. Time	Area	Height	Area%
1	25.050	1697265	50524	18.182
2	31.128	7637421	177540	81.818
Total		9334686	228064	100.000

Figure S18. HPLC chromatogram of product **10g** (enantioselective synthesis).

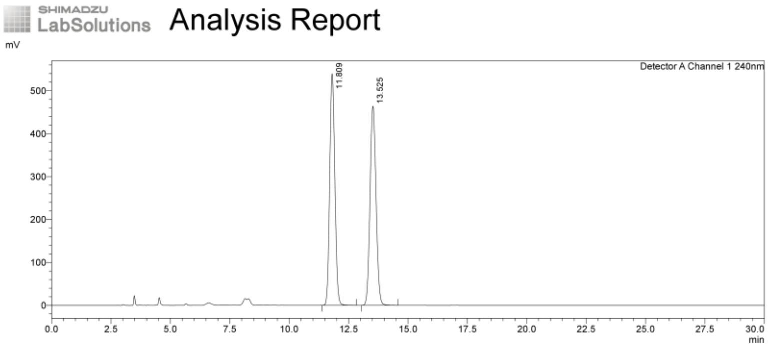

Detector A Channel 1 240nm

Peak#	Ret. Time	Area	Height	Area%
1	11.809	8137934	539397	49.994
2	13.525	8139962	464008	50.006
Total		16277896	1003405	100.000

Figure S19. HPLC chromatogram of product **10h** (racemic synthesis).

SHIMADZU LabSolutions **Analysis Report**

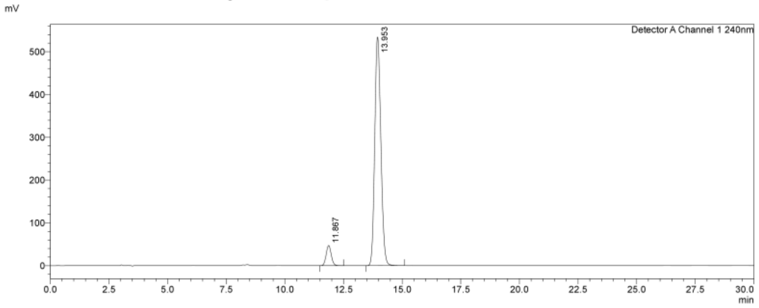

Detector A Channel 1 240nm

Peak#	Ret. Time	Area	Height	Area%
1	11.867	713028	47216	6.829
2	13.953	9727531	534091	93.171
Total		10440559	581307	100.000

Figure S20. HPLC chromatogram of product **10h** (enantioselective synthesis).

SHIMADZU LabSolutions **Analysis Report**

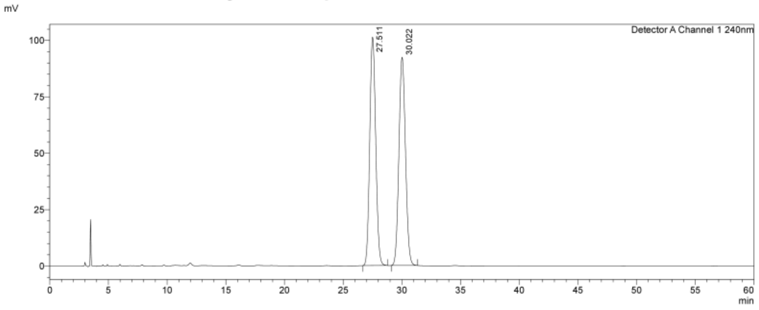

Detector A Channel 1 240nm

Peak#	Ret. Time	Area	Height	Area%
1	27.511	3574776	101222	49.976
2	30.022	3578177	92254	50.024
Total		7152953	193476	100.000

Figure S21. HPLC chromatogram of product **10i** (racemic synthesis).

SHIMADZU LabSolutions **Analysis Report**

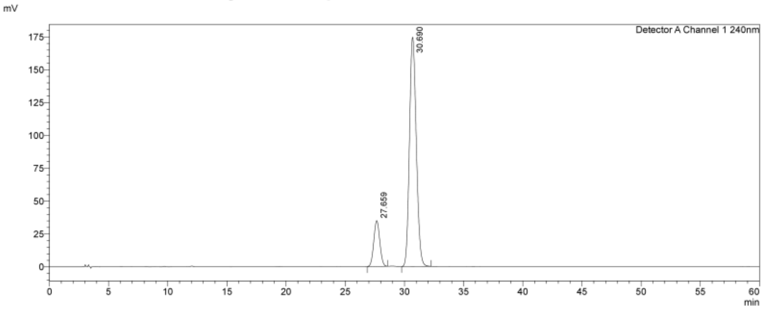

Detector A Channel 1 240nm

Peak#	Ret. Time	Area	Height	Area%
1	27.659	1226447	34815	14.900
2	30.690	7004587	174421	85.100
Total		8231034	209236	100.000

Figure S22. HPLC chromatogram of product **10i** (enantioselective synthesis).

SHIMADZU LabSolutions **Analysis Report**

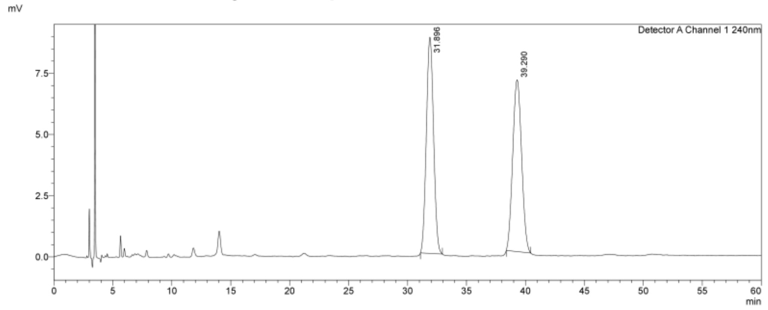

Detector A Channel 1 240nm

Peak#	Ret. Time	Area	Height	Area%
1	31.896	375994	8831	50.668
2	39.290	366085	7018	49.332
Total		742079	15849	100.000

Figure S23. HPLC chromatogram of product **10j** (racemic synthesis).

SHIMADZU LabSolutions **Analysis Report**

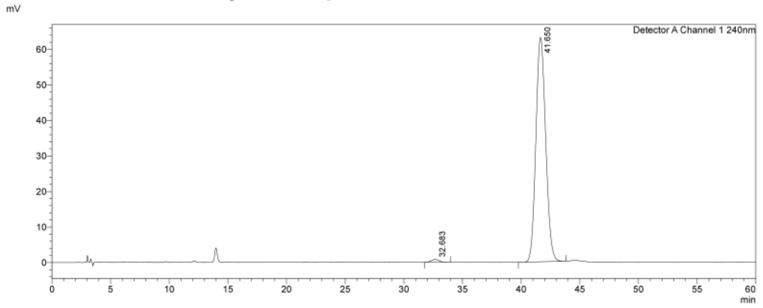

Detector A Channel 1 240nm

Peak#	Ret. Time	Area	Height	Area%
1	32.683	35444	769	0.962
2	41.650	3649453	63139	99.038
Total		3684897	63908	100.000

Figure S24. HPLC chromatogram of product **10j** (enantioselective synthesis).

SHIMADZU LabSolutions **Analysis Report**

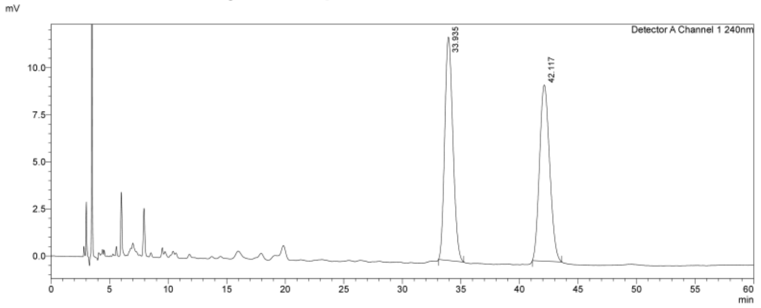

Detector A Channel 1 240nm

Peak#	Ret. Time	Area	Height	Area%
1	33.935	573916	11868	50.499
2	42.117	562582	9358	49.501
Total		1136498	21226	100.000

Figure S25. HPLC chromatogram of product **10k** (racemic synthesis).

SHIMADZU LabSolutions **Analysis Report**

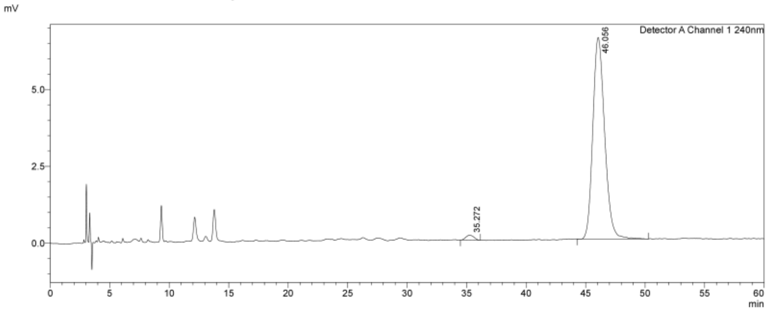

Detector A Channel 1 240nm

Peak#	Ret. Time	Area	Height	Area%
1	35.272	7328	160	1.590
2	46.056	453657	6558	98.410
Total		460985	6718	100.000

Figure S26. HPLC chromatogram of product **10k** (enantioselective synthesis).

SHIMADZU LabSolutions **Analysis Report**

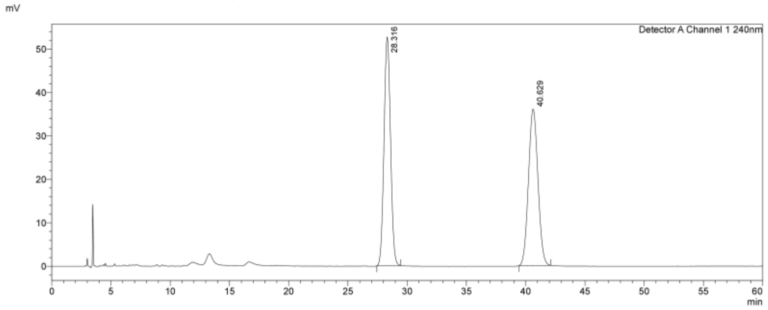

Detector A Channel 1 240nm

Peak#	Ret. Time	Area	Height	Area%
1	28.316	2009740	52670	50.086
2	40.629	2002808	36040	49.914
Total		4012548	88710	100.000

Figure S27. HPLC chromatogram of product **10l** (racemic synthesis).

SHIMADZU LabSolutions **Analysis Report**

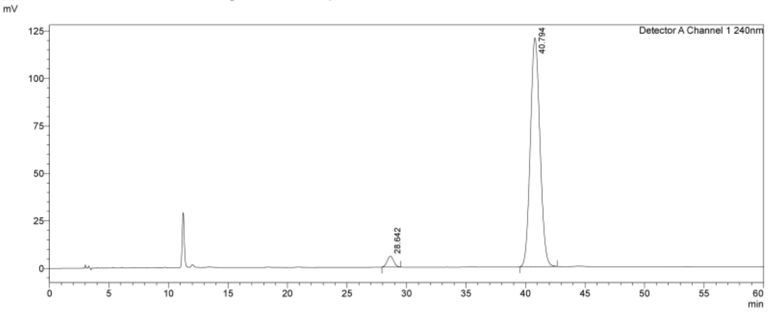

Detector A Channel 1 240nm

Peak#	Ret. Time	Area	Height	Area%
1	28.642	218431	5741	3.128
2	40.794	6765716	120480	96.872
Total		6984147	126221	100.000

Figure S28. HPLC chromatogram of product **10l** (enantioselective synthesis).

SHIMADZU LabSolutions **Analysis Report**

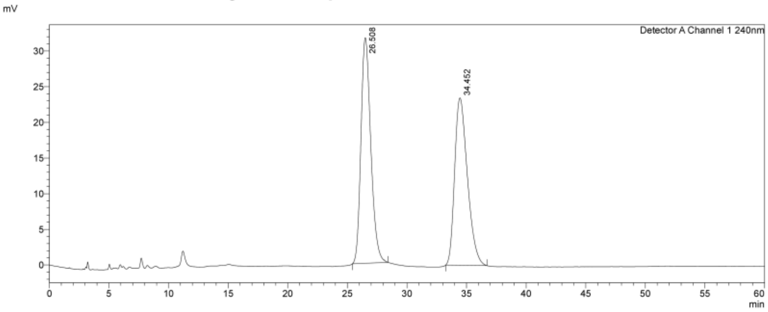

Detector A Channel 1 240nm

Peak#	Ret. Time	Area	Height	Area%
1	26.508	1806783	31618	50.906
2	34.452	1742463	23446	49.094
Total		3549245	55064	100.000

Figure S29. HPLC chromatogram of product **10s** (racemic synthesis).

SHIMADZU LabSolutions **Analysis Report**

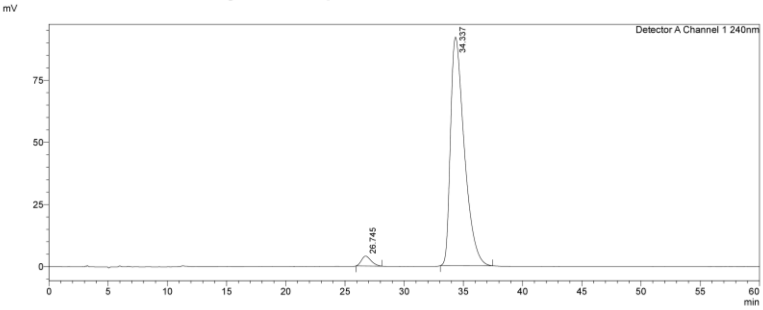

Detector A Channel 1 240nm

Peak#	Ret. Time	Area	Height	Area%
1	26.745	224125	3969	2.920
2	34.337	7451956	91923	97.080
Total		7676082	95892	100.000

Figure S30. HPLC chromatogram of product **10s** (enantioselective synthesis).

SHIMADZU LabSolutions **Analysis Report**

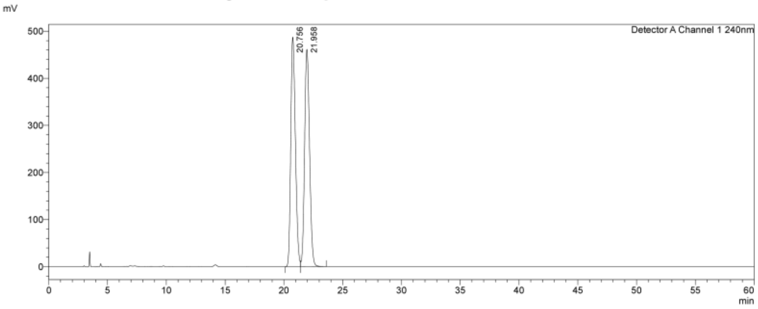

Detector A Channel 1 240nm

Peak#	Ret. Time	Area	Height	Area%
1	20.756	13344189	487384	49.789
2	21.958	13457293	461567	50.211
Total		26801481	948951	100.000

Figure S31. HPLC chromatogram of product **10t** (racemic synthesis).

Analysis Report

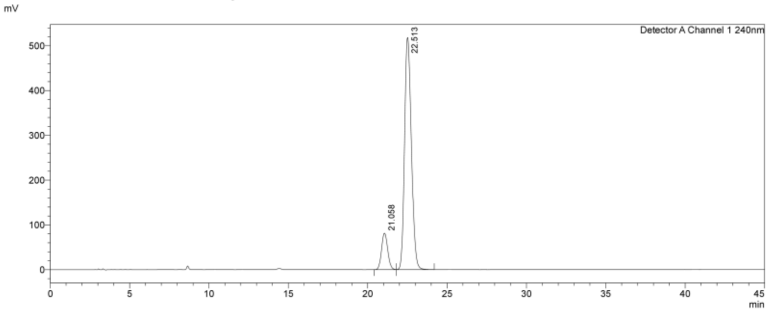

Detector A Channel 1 240nm

Peak#	Ret. Time	Area	Height	Area%
1	21.058	2193682	81398	12.436
2	22.513	15446065	518071	87.564
Total		17639747	599468	100.000

Figure S32. HPLC chromatogram of product **10t** (enantioselective synthesis).

Analysis Report

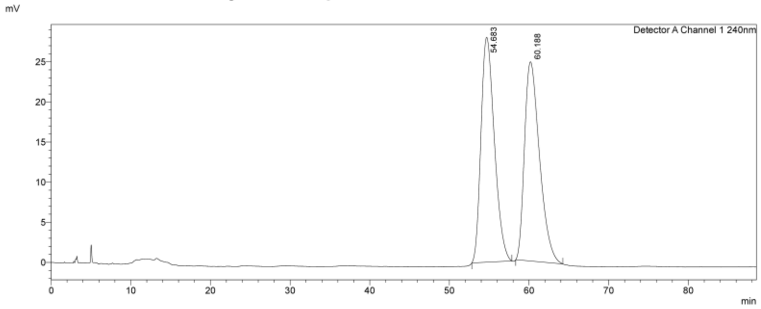

Detector A Channel 1 240nm

Peak#	Ret. Time	Area	Height	Area%
1	54.683	3272911	28031	50.128
2	60.188	3256139	24827	49.872
Total		6529051	52858	100.000

Figure S33. HPLC chromatogram of product **10u** (racemic synthesis).

Analysis Report

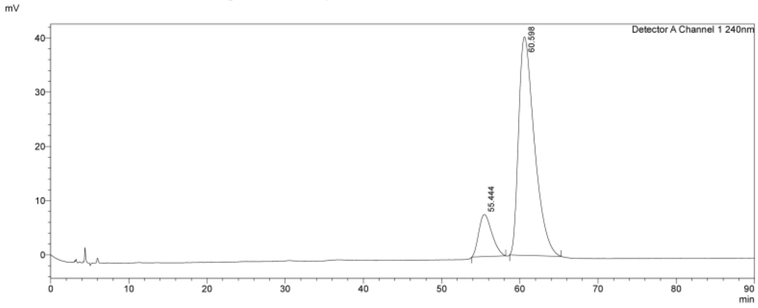

Detector A Channel 1 240nm

Peak#	Ret. Time	Area	Height	Area%
1	55.444	905530	7770	13.730
2	60.598	5689858	40303	86.270
Total		6595388	48073	100.000

Figure S34. HPLC chromatogram of product **10u** (enantioselective synthesis).

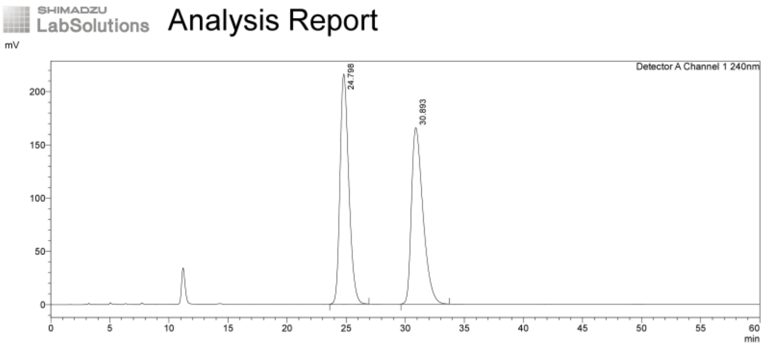

Detector A Channel 1 240nm

Peak#	Ret. Time	Area	Height	Area%
1	24.798	11248774	216141	50.022
2	30.893	11239067	165642	49.978
Total		22487841	381782	100.000

Figure S35. HPLC chromatogram of product **10v** (racemic synthesis).

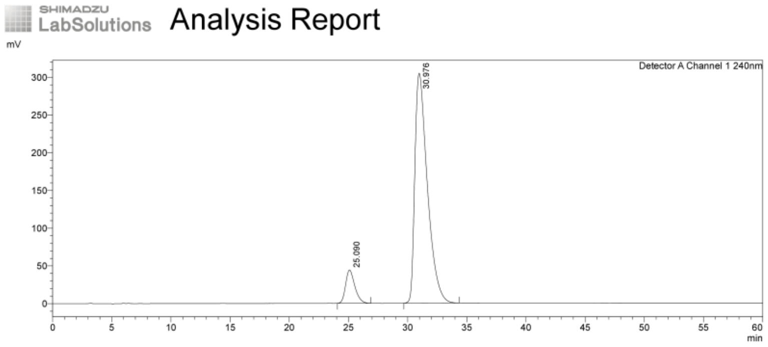

Detector A Channel 1 240nm

Peak#	Ret. Time	Area	Height	Area%
1	25.090	2375529	43991	9.584
2	30.976	22409838	304807	90.416
Total		24785368	348798	100.000

Figure S36. HPLC chromatogram of product **10v** (enantioselective synthesis).

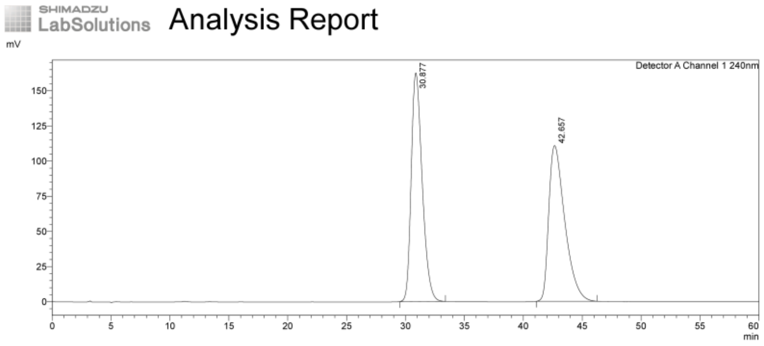

Detector A Channel 1 240nm

Peak#	Ret. Time	Area	Height	Area%
1	30.877	10502415	162533	50.090
2	42.657	10464797	110679	49.910
Total		20967212	273212	100.000

Figure S37. HPLC chromatogram of product **10w** (racemic synthesis).

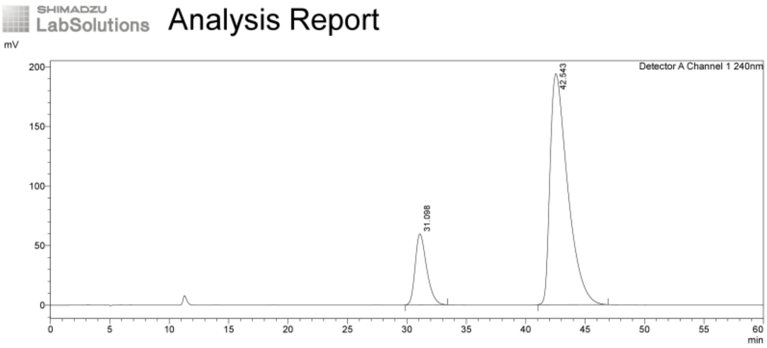

Detector A Channel 1 240nm

Peak#	Ret. Time	Area	Height	Area%
1	31.098	3998166	59507	16.664
2	42.543	19994254	193801	83.336
Total		23992420	253308	100.000

Figure S38. HPLC chromatogram of product **10w** (enantioselective synthesis).

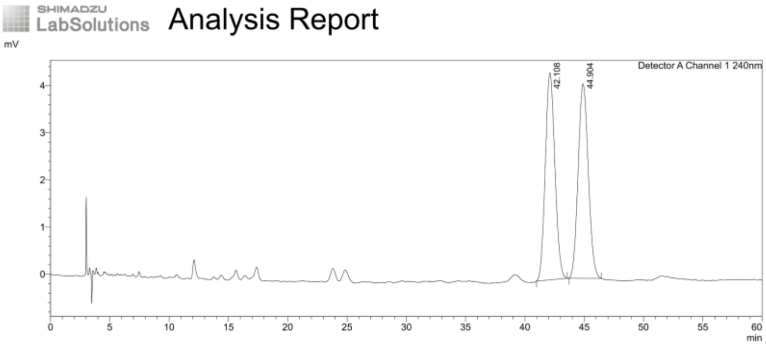

Detector A Channel 1 240nm

Peak#	Ret. Time	Area	Height	Area%
1	42.108	246917	4387	50.084
2	44.904	246089	4126	49.916
Total		493007	8513	100.000

Figure S39. HPLC chromatogram of product **10x** (racemic synthesis).

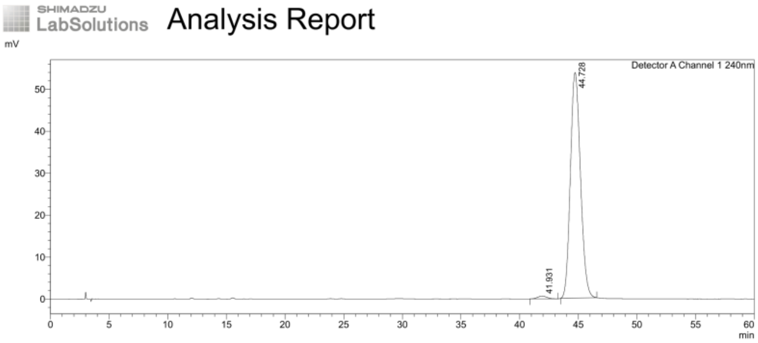

Detector A Channel 1 240nm

Peak#	Ret. Time	Area	Height	Area%
1	41.931	36256	658	1.120
2	44.728	3200838	53743	98.880
Total		3237095	54401	100.000

Figure S40. HPLC chromatogram of product **10x** (enantioselective synthesis).

SHIMADZU LabSolutions **Analysis Report**

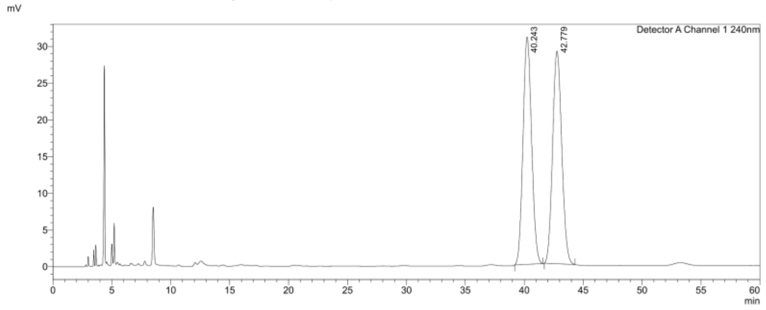

Detector A Channel 1 240nm

Peak#	Ret. Time	Area	Height	Area%
1	40.243	1572935	30975	50.087
2	42.779	1567493	28972	49.913
Total		3140428	59947	100.000

Figure S41. HPLC chromatogram of product **10y** (racemic synthesis).

SHIMADZU LabSolutions **Analysis Report**

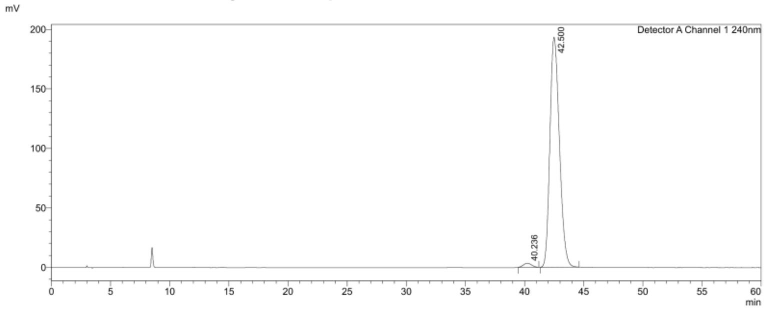

Detector A Channel 1 240nm

Peak#	Ret. Time	Area	Height	Area%
1	40.236	165689	3417	1.512
2	42.500	10790366	193142	98.488
Total		10956055	196559	100.000

Figure S42. HPLC chromatogram of product **10y** (enantioselective synthesis).

SHIMADZU LabSolutions **Analysis Report**

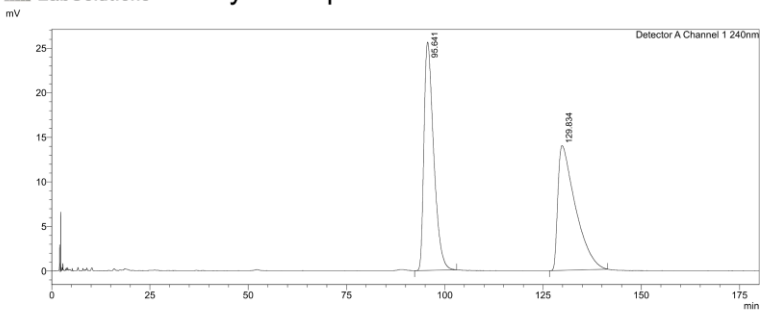

Detector A Channel 1 240nm

Peak#	Ret. Time	Area	Height	Area%
1	95.641	4397227	25609	50.276
2	129.834	4348982	14008	49.724
Total		8746209	39616	100.000

Figure S43. HPLC chromatogram of product **10z** (racemic synthesis).

Analysis Report

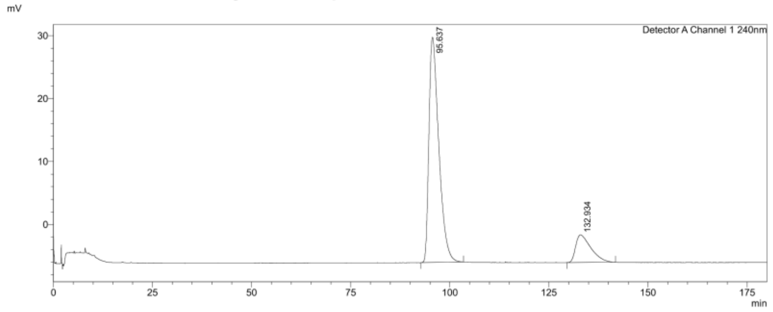

Detector A Channel 1 240nm

Peak#	Ret. Time	Area	Height	Area%
1	95.637	6195926	35779	84.087
2	132.934	1172562	4408	15.913
Total		7368488	40187	100.000

Figure S44. HPLC chromatogram of product **10z** (enantioselective synthesis).

Analysis Report

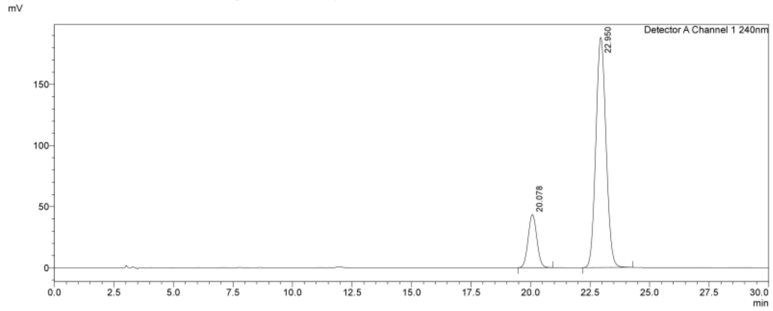

Detector A Channel 1 240nm

Peak#	Ret. Time	Area	Height	Area%
1	20.078	1107842	43487	16.578
2	22.950	5574957	188373	83.422
Total		6682799	231860	100.000

Figure S45. HPLC chromatogram of product **10a** (synthesized using $\mathbf{1}^{\mathbf{BArF}}$).

Analysis Report

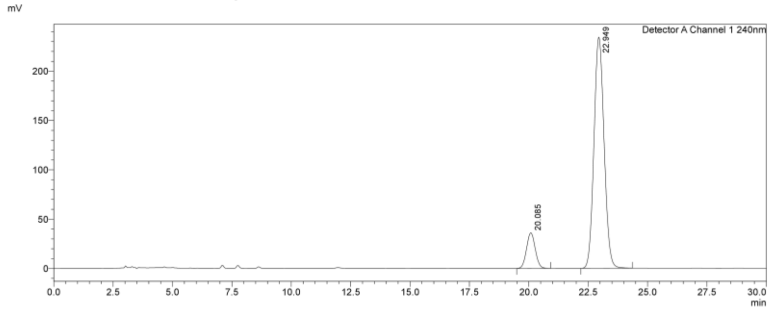

Detector A Channel 1 240nm

Peak#	Ret. Time	Area	Height	Area%
1	20.085	915899	35987	11.661
2	22.949	6938250	234193	88.339
Total		7854149	270179	100.000

Figure S46. HPLC chromatogram of product **10a** (synthesized using $\mathbf{7a}^{\mathbf{BArF}}$).

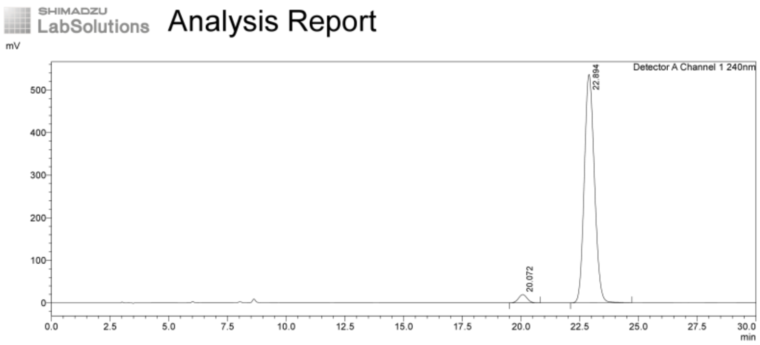

Detector A Channel 1 240nm

Peak#	Ret. Time	Area	Height	Area%
1	20.072	491537	19372	2.976
2	22.894	16022634	536173	97.024
Total		16514171	555545	100.000

Figure S47. HPLC chromatogram of product **10a** (synthesized using $\mathbf{7b}^{\mathbf{BArF}}$ (fraction 1)).

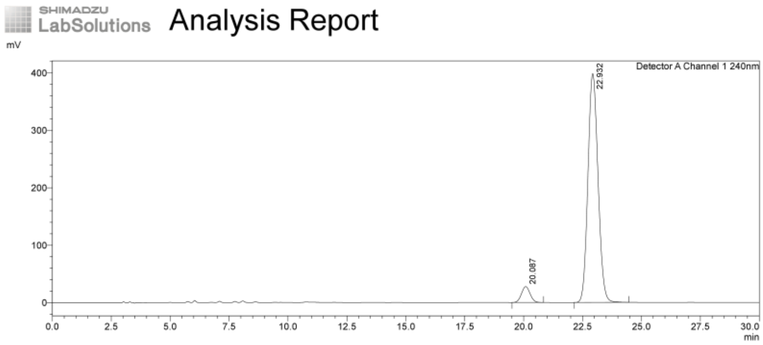

Detector A Channel 1 240nm

Peak#	Ret. Time	Area	Height	Area%
1	20.087	703730	27673	5.600
2	22.932	11862571	398331	94.400
Total		12566301	426004	100.000

Figure S48. HPLC chromatogram of product **10a** (synthesized using $\mathbf{7c}^{\mathbf{BArF}}$ (fraction 1)).

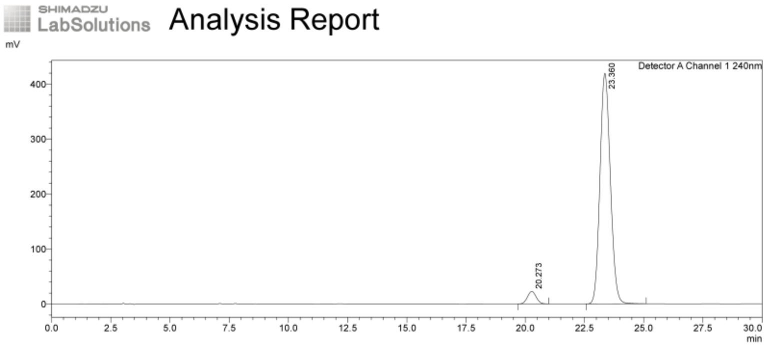

Detector A Channel 1 240nm

Peak#	Ret. Time	Area	Height	Area%
1	20.273	579278	22690	4.347
2	23.360	12746618	419248	95.653
Total		13325896	441938	100.000

Figure S49. HPLC chromatogram of product **10a** (synthesized using $\mathbf{7c}^{\mathbf{BArF}}$ (fraction 2)).

SHIMADZU LabSolutions **Analysis Report**

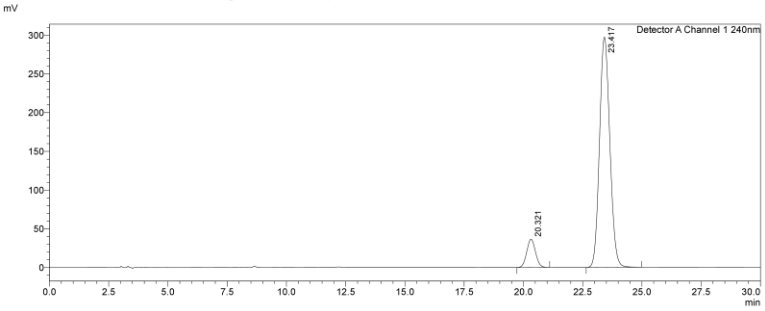

Detector A Channel 1 240nm

Peak#	Ret. Time	Area	Height	Area%
1	20.321	934485	36433	9.401
2	23.417	9005363	297223	90.599
Total		9939848	333656	100.000

Figure S50. HPLC chromatogram of product **10a** (synthesized using **7d$^{\mathbf{BArF}}$** (fraction 1)).

SHIMADZU LabSolutions **Analysis Report**

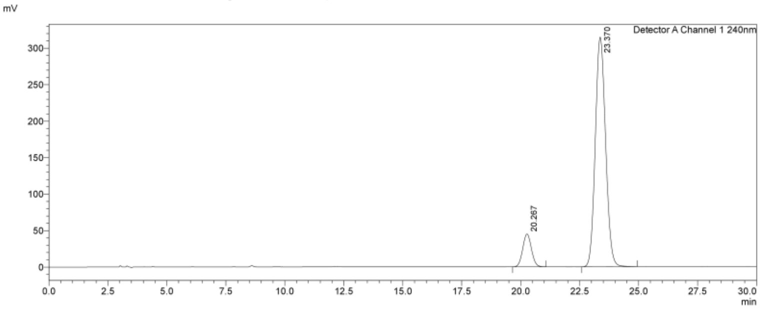

Detector A Channel 1 240nm

Peak#	Ret. Time	Area	Height	Area%
1	20.267	1149915	44874	10.764
2	23.370	9532996	314518	89.236
Total		10682910	359392	100.000

Figure S51. HPLC chromatogram of product **10a** (synthesized using **7d$^{\mathbf{BArF}}$** (fraction 2)).

SHIMADZU LabSolutions **Analysis Report**

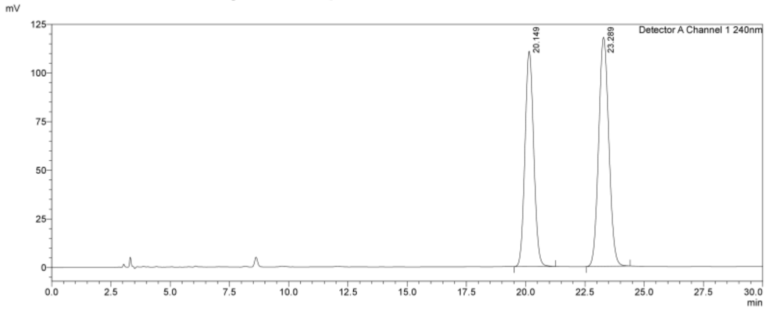

Detector A Channel 1 240nm

Peak#	Ret. Time	Area	Height	Area%
1	20.149	2835341	110668	44.660
2	23.289	3513398	117713	55.340
Total		6348739	228380	100.000

Figure S52. HPLC chromatogram of product **10a** (synthesized using **7e$^{\mathbf{BArF}}$**).

SHIMADZU LabSolutions **Analysis Report**

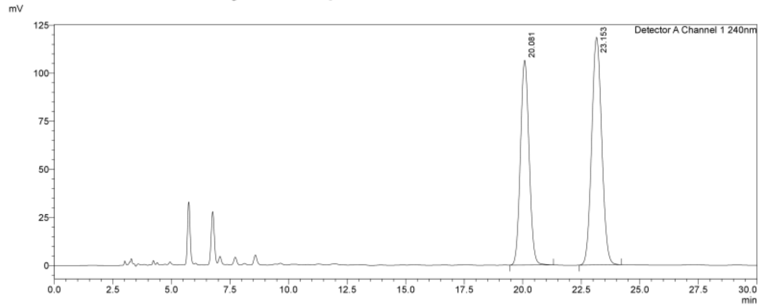

Detector A Channel 1 240nm

Peak#	Ret. Time	Area	Height	Area%
1	20.081	2737377	106442	43.685
2	23.153	3528779	118307	56.315
Total		6266156	224749	100.000

Figure S53. HPLC chromatogram of product **10a** (synthesized using **5a$^{\mathbf{BArF}}$**).

SHIMADZU LabSolutions **Analysis Report**

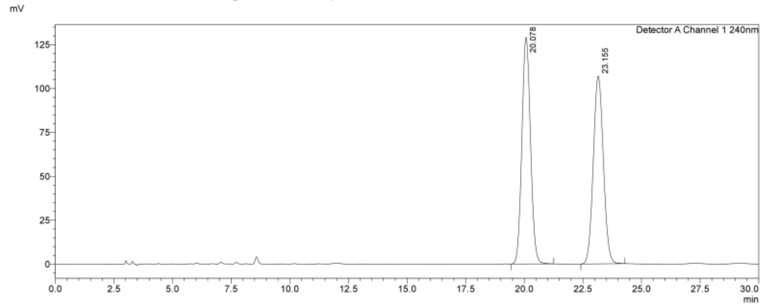

Detector A Channel 1 240nm

Peak#	Ret. Time	Area	Height	Area%
1	20.078	3317283	129106	50.970
2	23.155	3191002	106923	49.030
Total		6508285	236030	100.000

Figure S54. HPLC chromatogram of product **10a** (synthesized using **5b$^{\mathbf{BArF}}$**).

5. NMR spectra

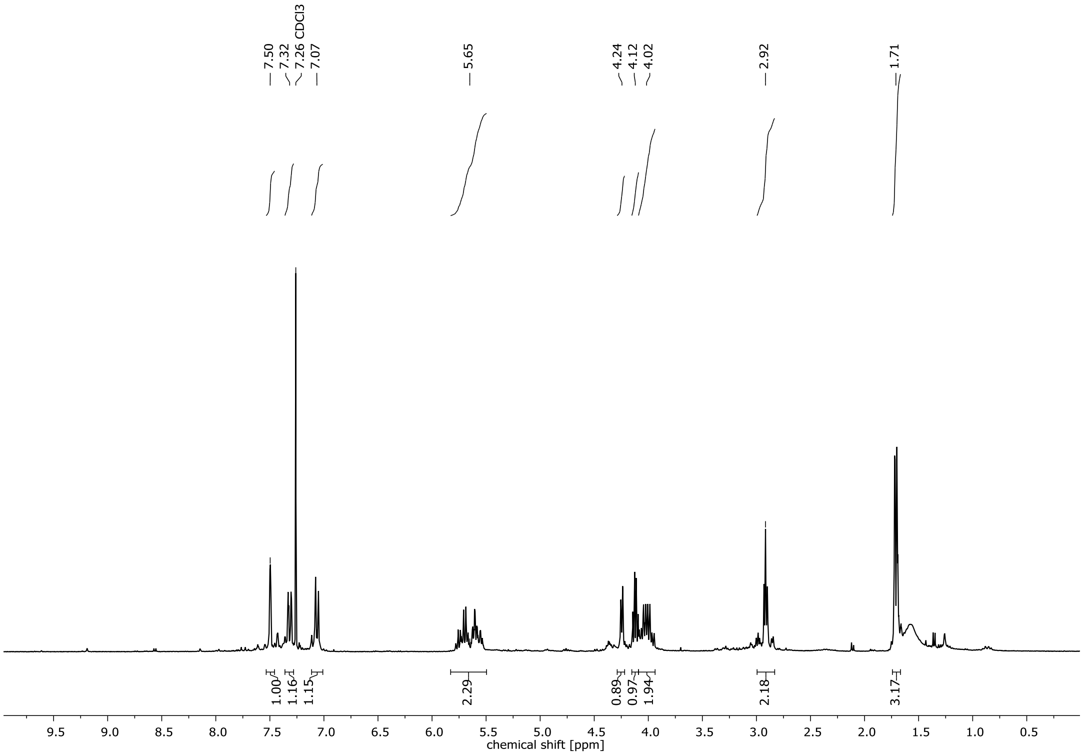

Figure S55. ^{1}H NMR spectrum of crude **SI-1** (300 MHz, chloroform-*d*).

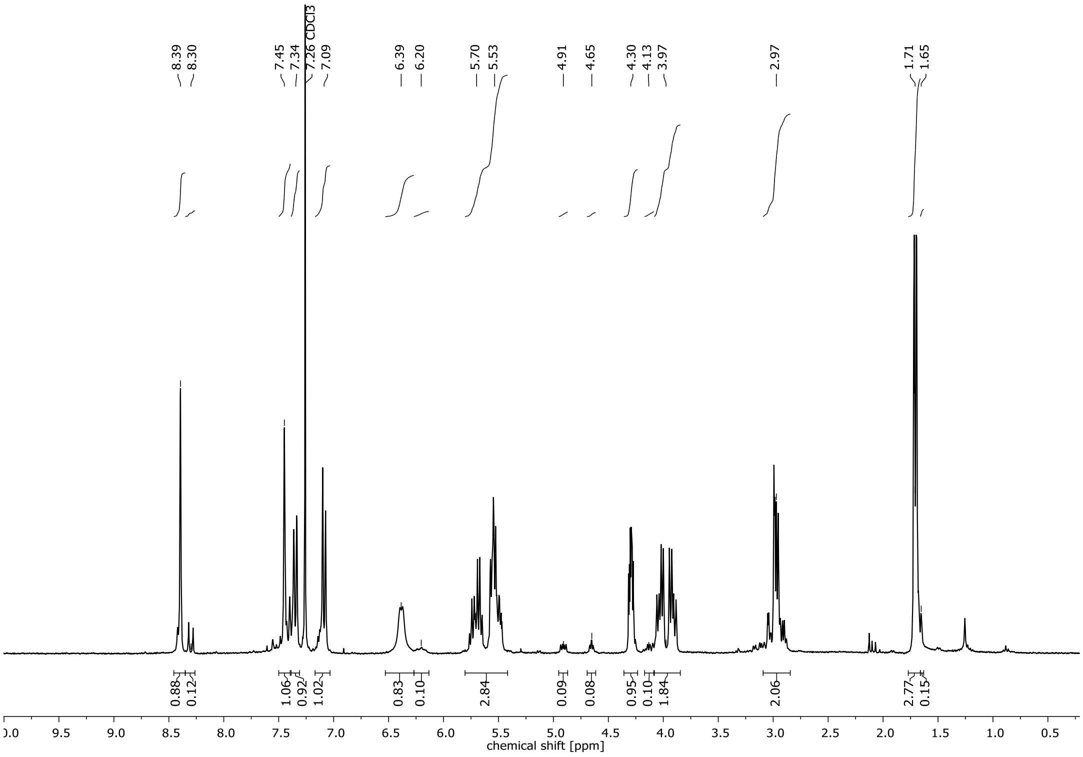

Figure S56. ^{1}H NMR spectrum of crude **SI-2** (300 MHz, chloroform-*d*).

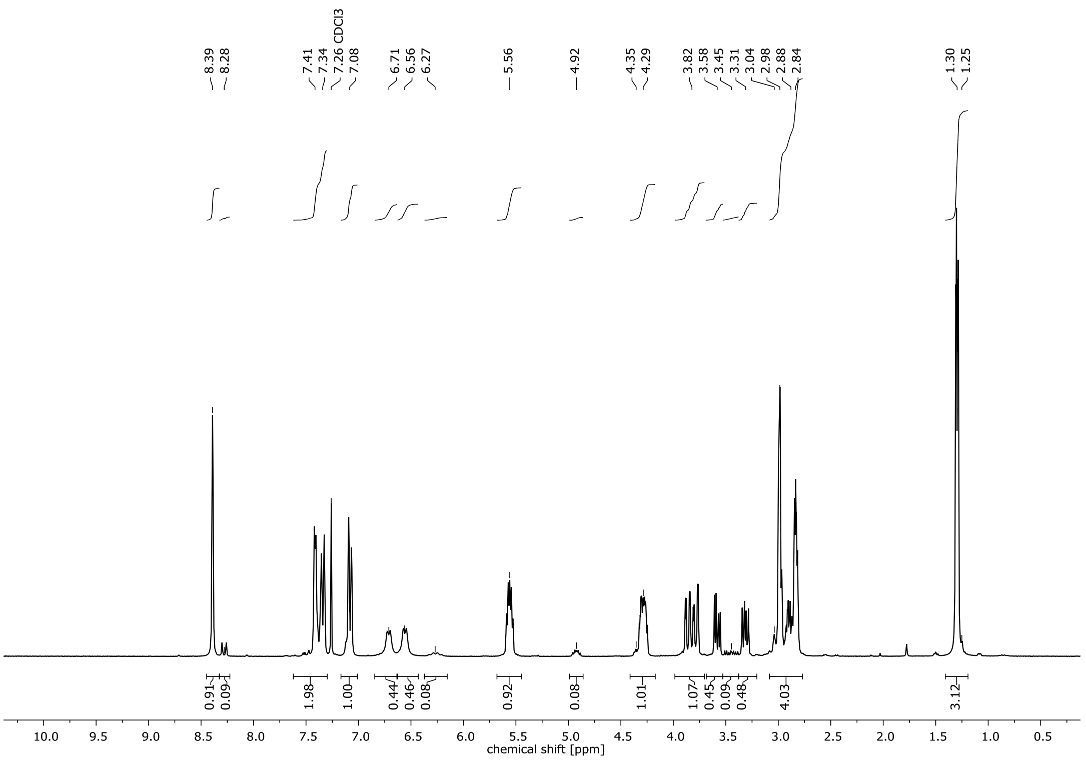

Figure S57. ^{1}H NMR spectrum of **SI-3** (300 MHz, chloroform-*d*).

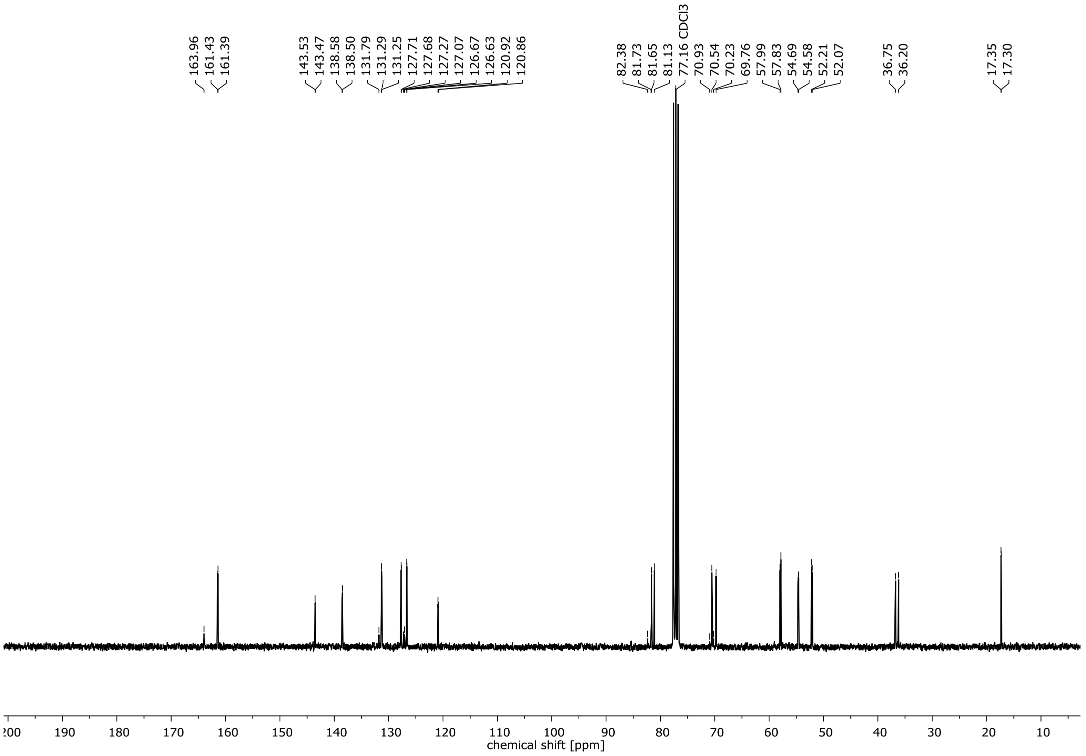

Figure S58. ^{13}C NMR spectrum of **SI-3** (75 MHz, chloroform-*d*).

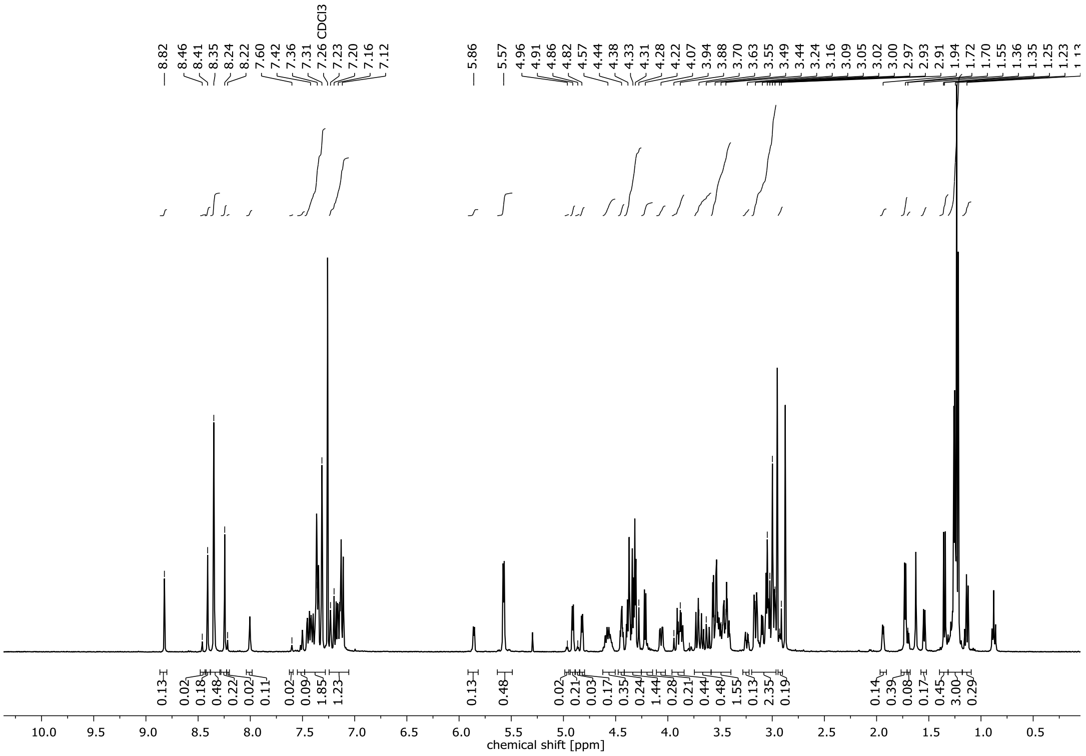

Figure S59. ^{1}H NMR spectrum of crude **SI-4** with residual dimethylformamide (300 MHz, chloroform-*d*).

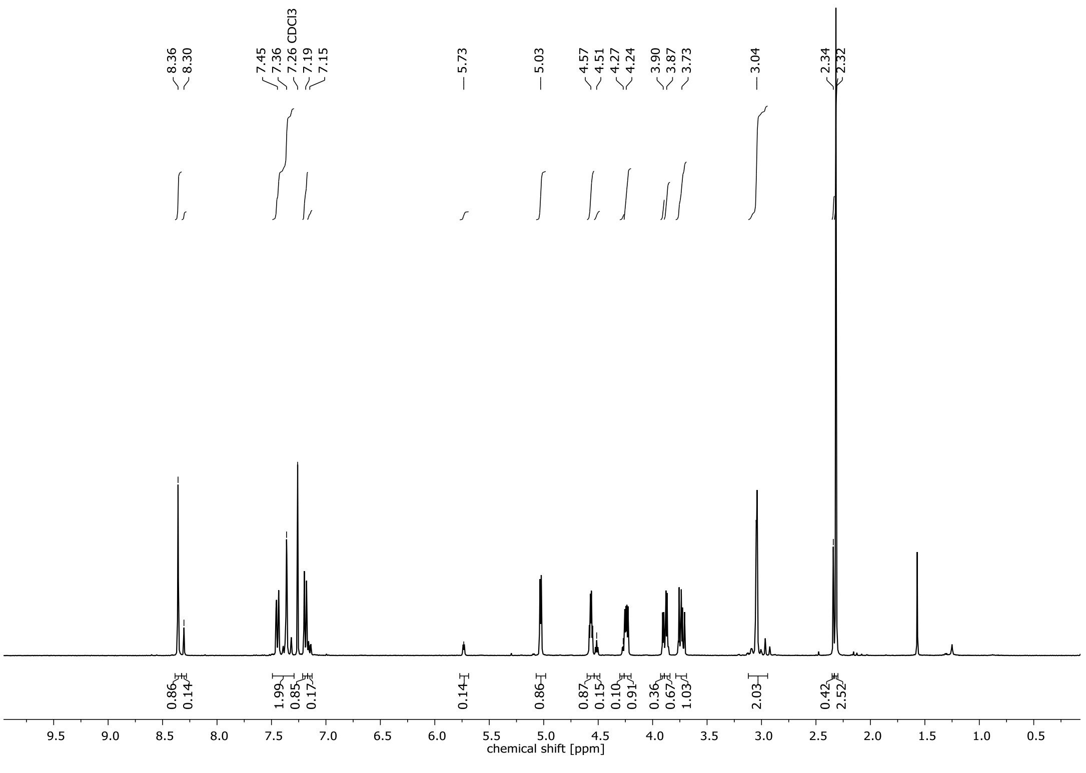

Figure S60. ^{1}H NMR spectrum of **3a** (400 MHz, chloroform-*d*).

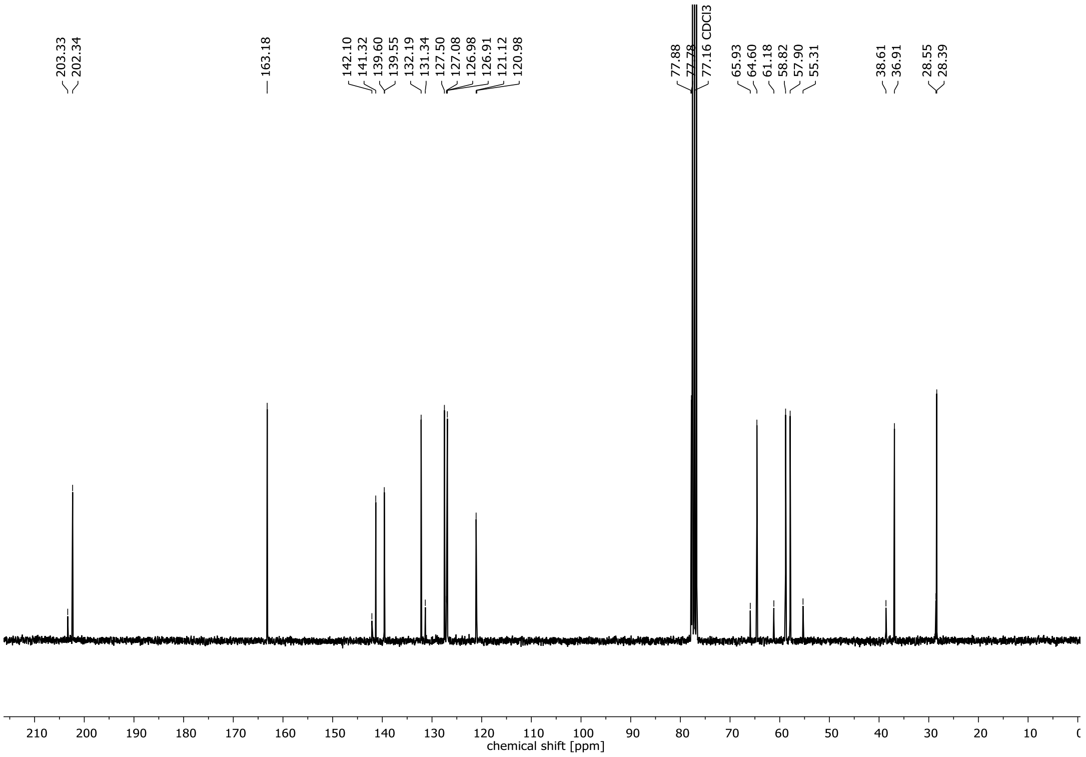

Figure S61. ^{13}C NMR spectrum of **3a** (75 MHz, chloroform-*d*).

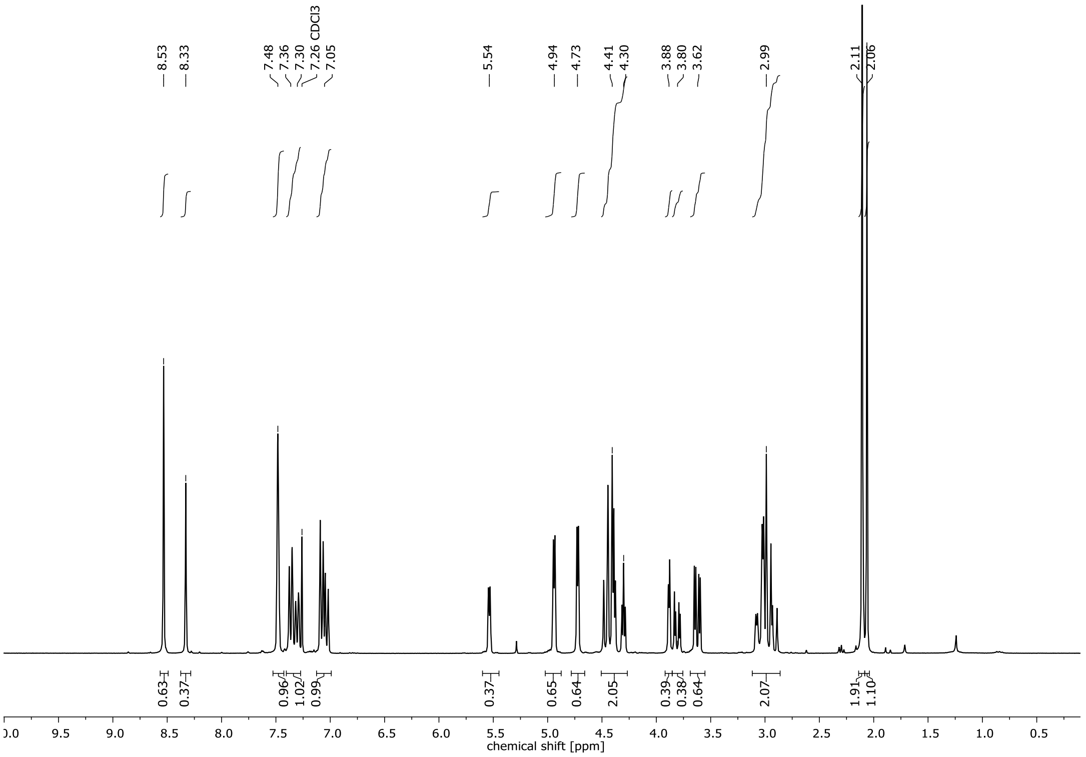

Figure S62. ^{1}H NMR spectrum of **3b** (300 MHz, chloroform-*d*).

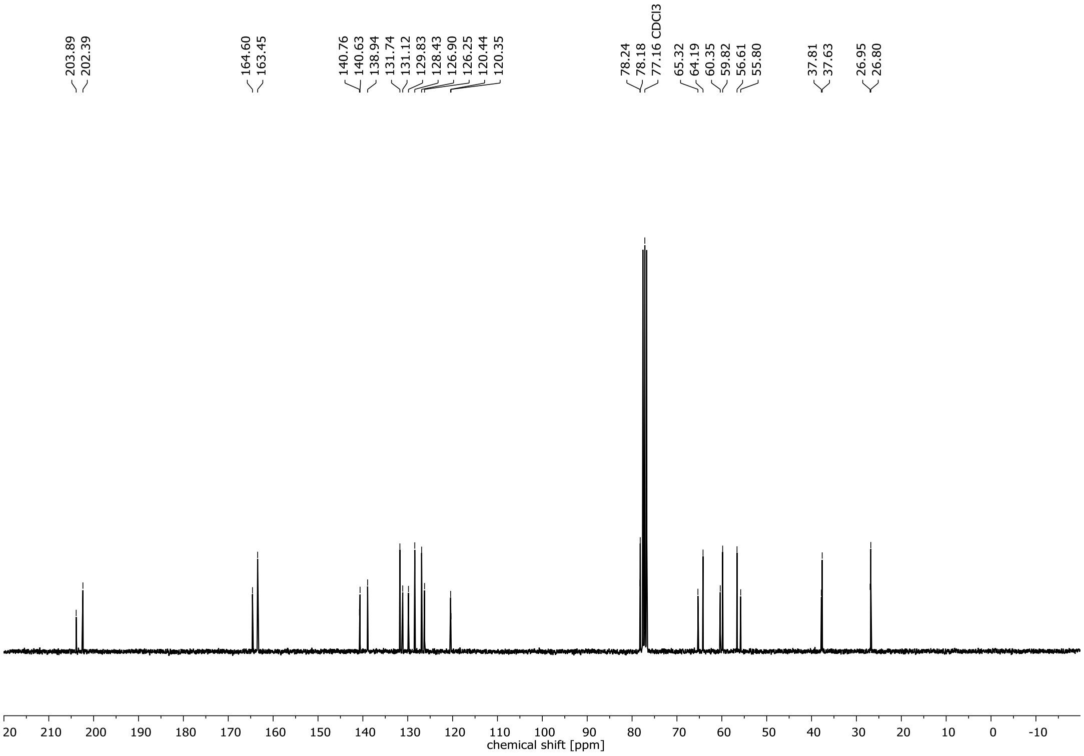

Figure S63. ^{13}C NMR spectrum of **3b** (75 MHz, chloroform-*d*).

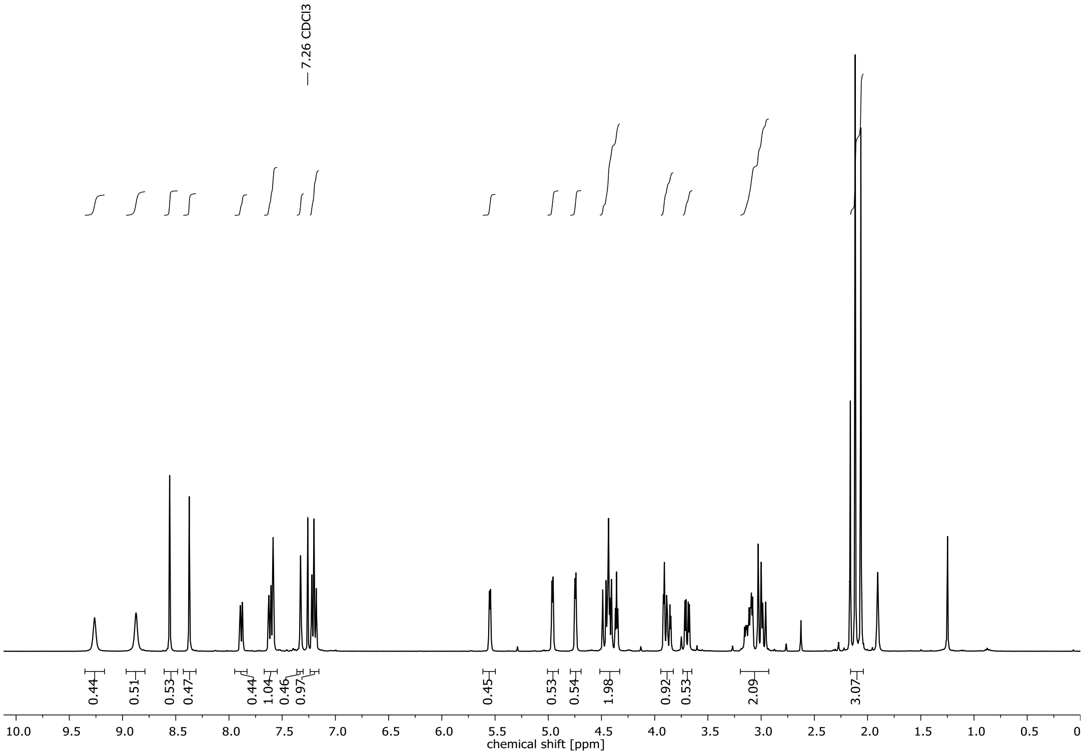

Figure S64. ^{1}H NMR spectrum of **4** (400 MHz, chloroform-*d*).

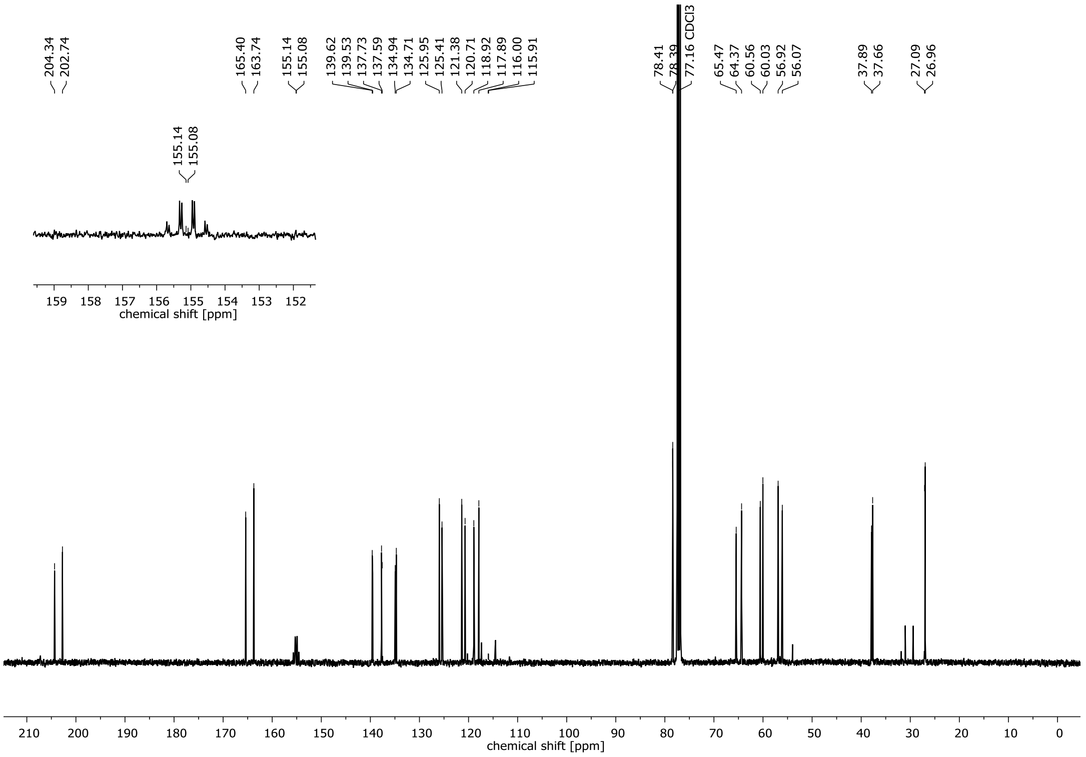

Figure S65. ^{13}C NMR spectrum of **4** (101 MHz, chloroform-*d*).

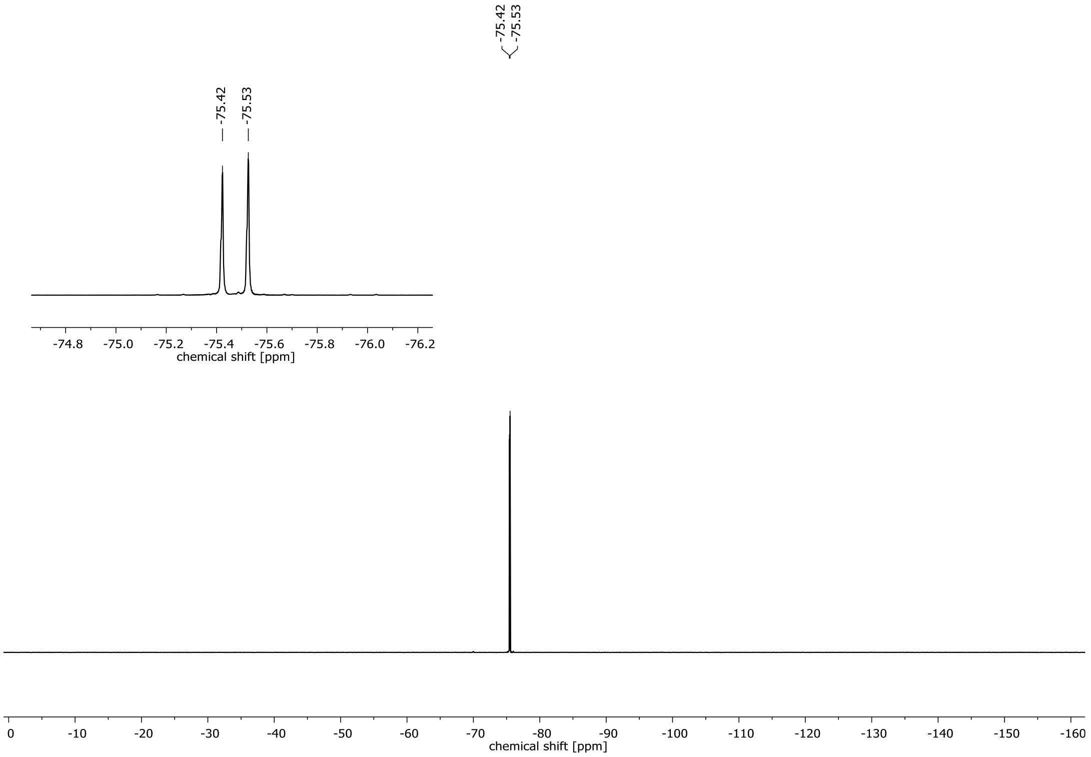

Figure S66. ^{19}F NMR spectrum of **4** (376 MHz, chloroform-*d*).

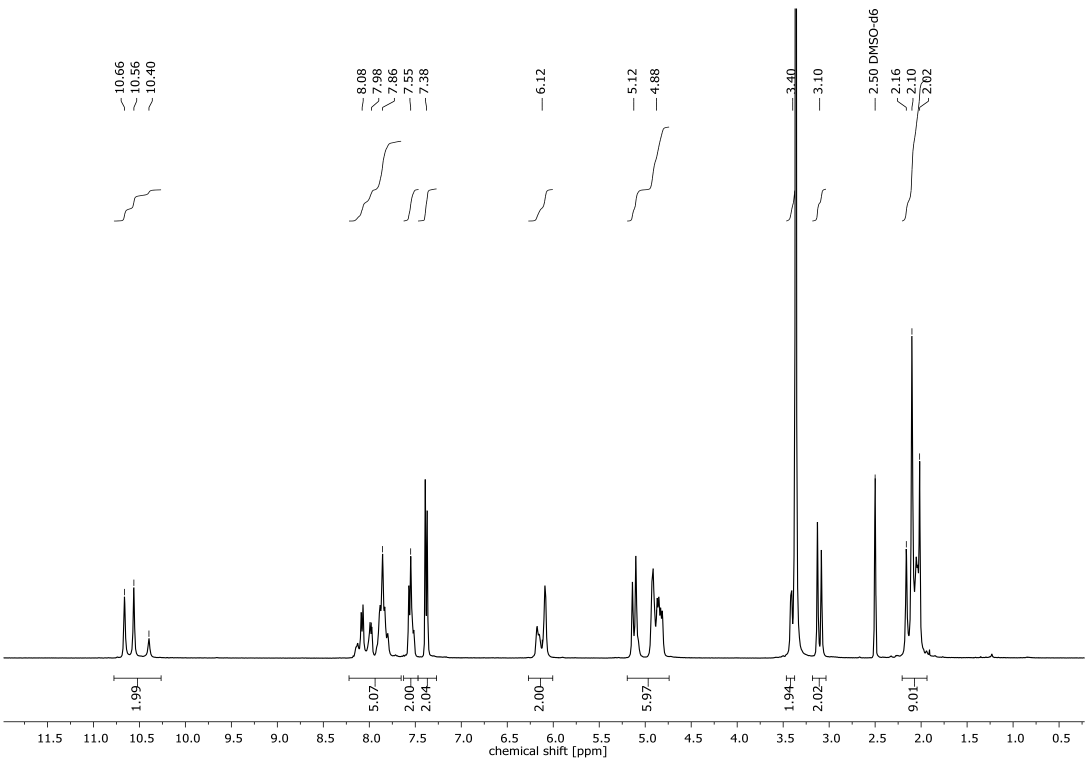

Figure S67. ^{1}H NMR spectrum of **5a^{Cl}** (400 MHz, DMSO-d_6).

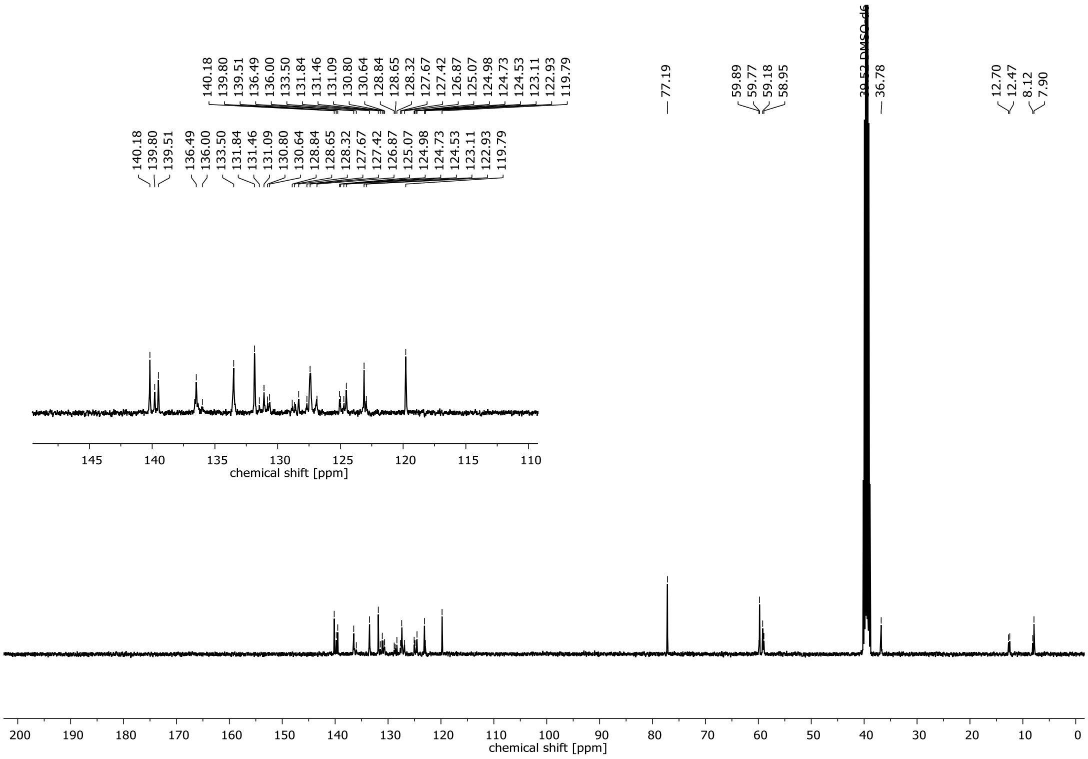

Figure S68. ^{13}C NMR spectrum of **5a^{Cl}** (101 MHz, DMSO-d_6).

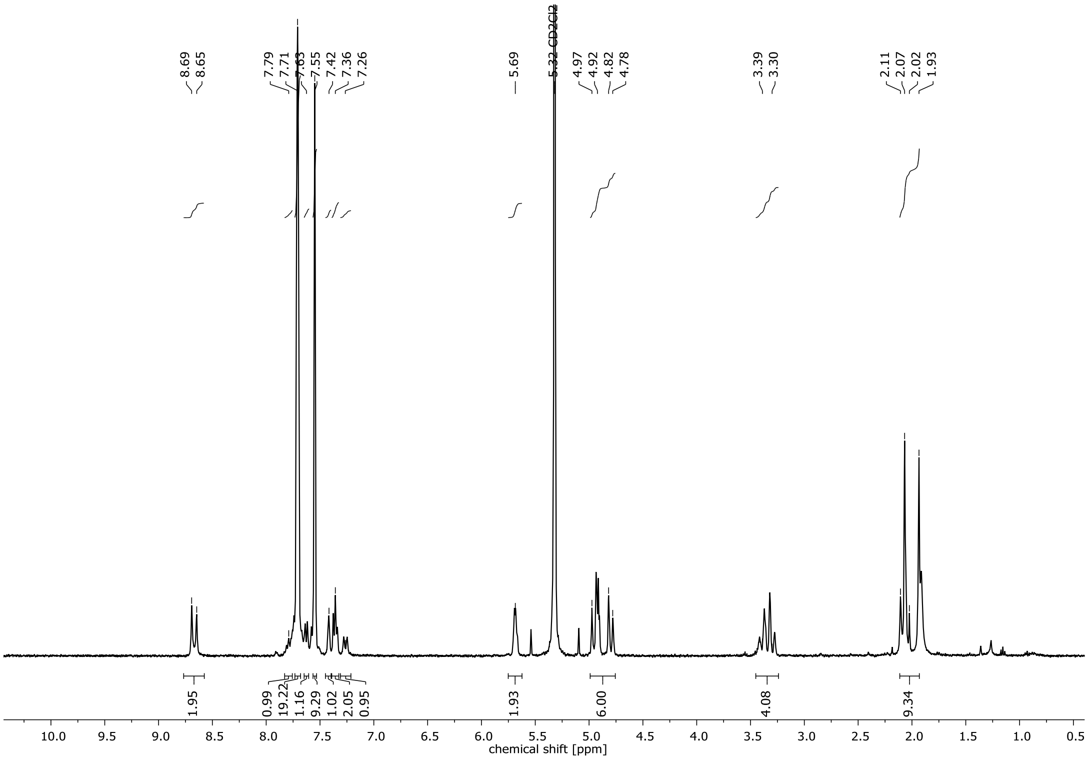

Figure S69. ^{1}H NMR spectrum of **5a^{BArF}** (400 MHz, DCM-d_2).

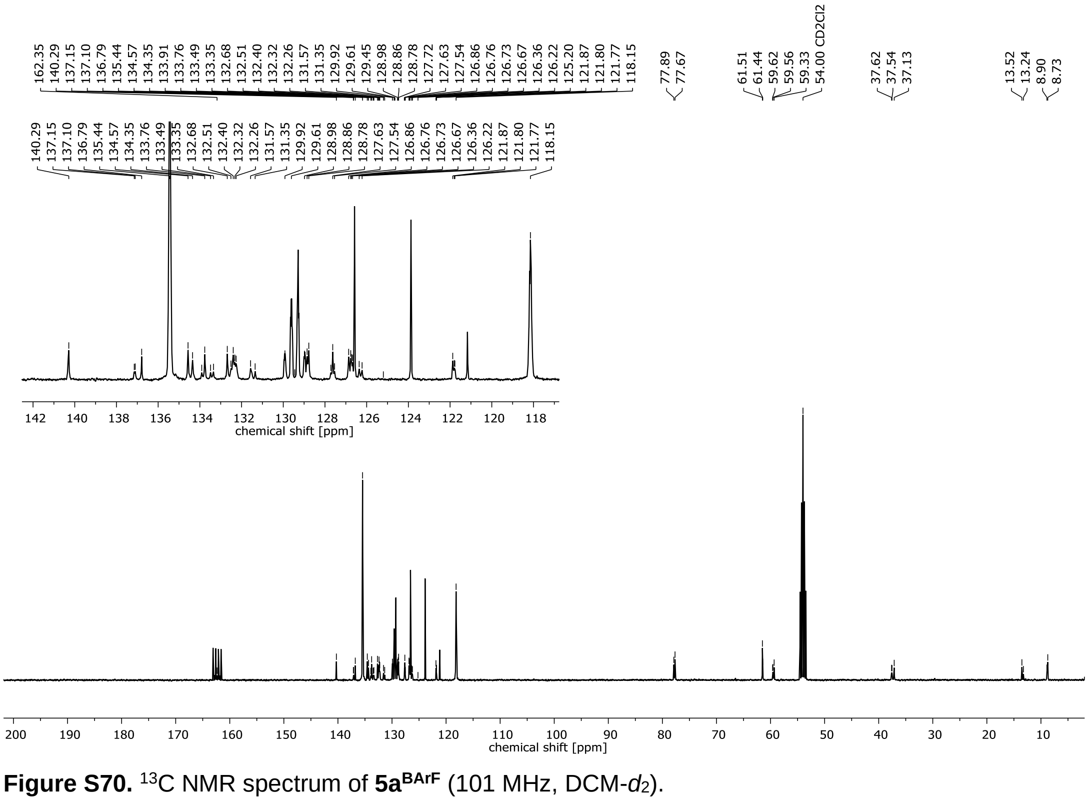

Figure S70. ^{13}C NMR spectrum of **5a^{BArF}** (101 MHz, DCM-d_2).

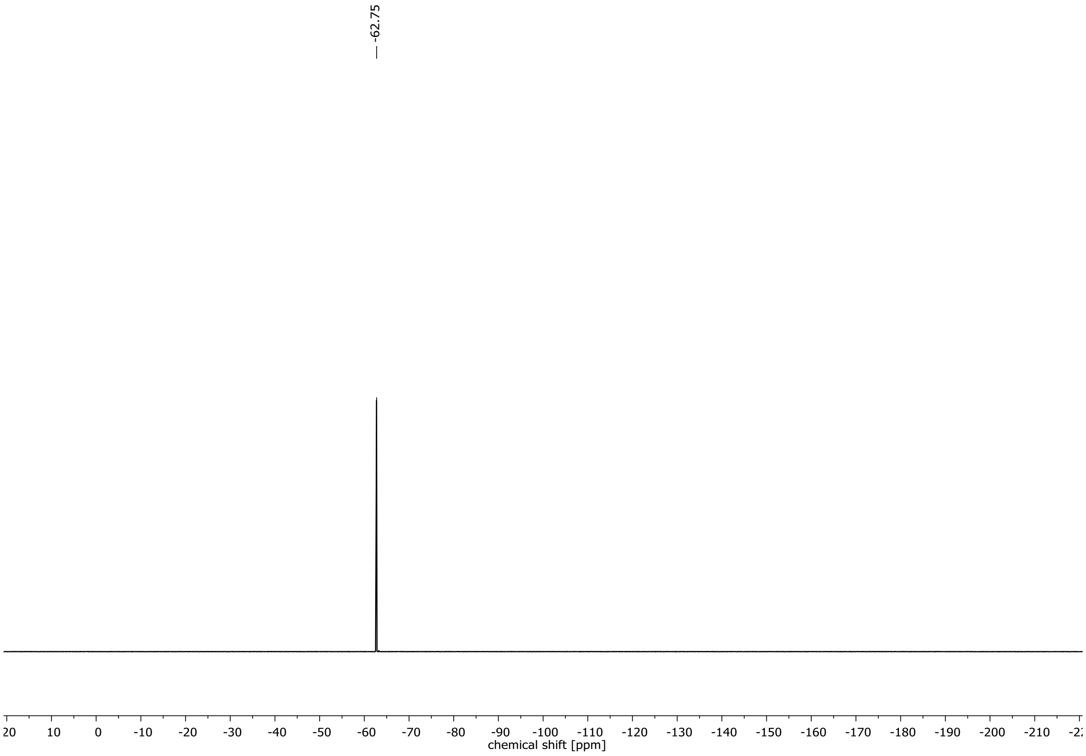

Figure S71. ^{19}F NMR spectrum of **5a$^{\mathbf{BArF}}$** (376 MHz, DCM-d_2).

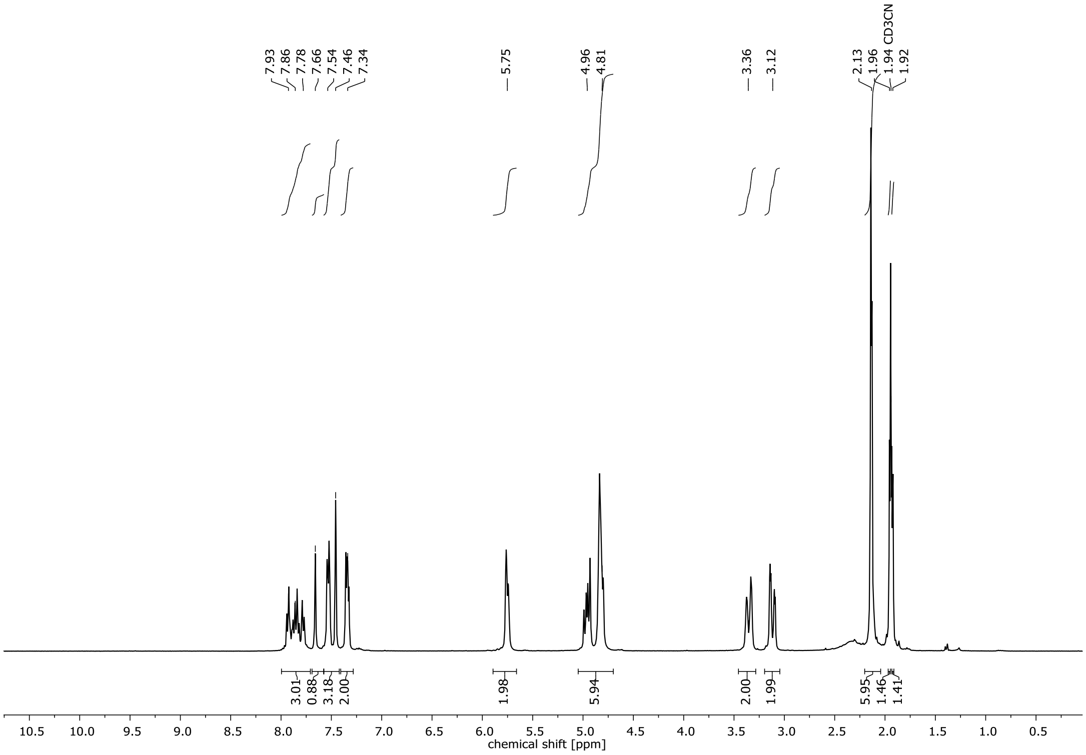

Figure S72. ^{1}H NMR spectrum of **7a$^{\mathbf{OTf}}$** (fraction 1) (400 MHz, acetonitrile-d_3).

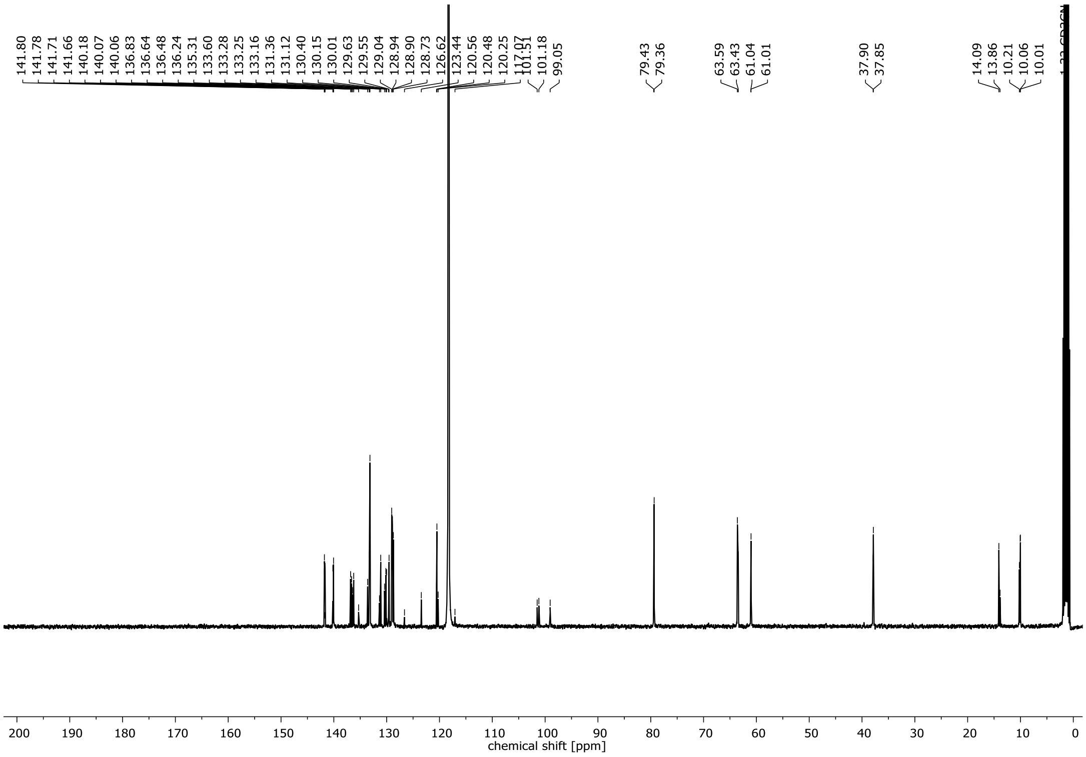

Figure S73. ^{13}C NMR spectrum of **7a^{OTf}** (fraction 1) (101 MHz, acetonitrile-d_3).

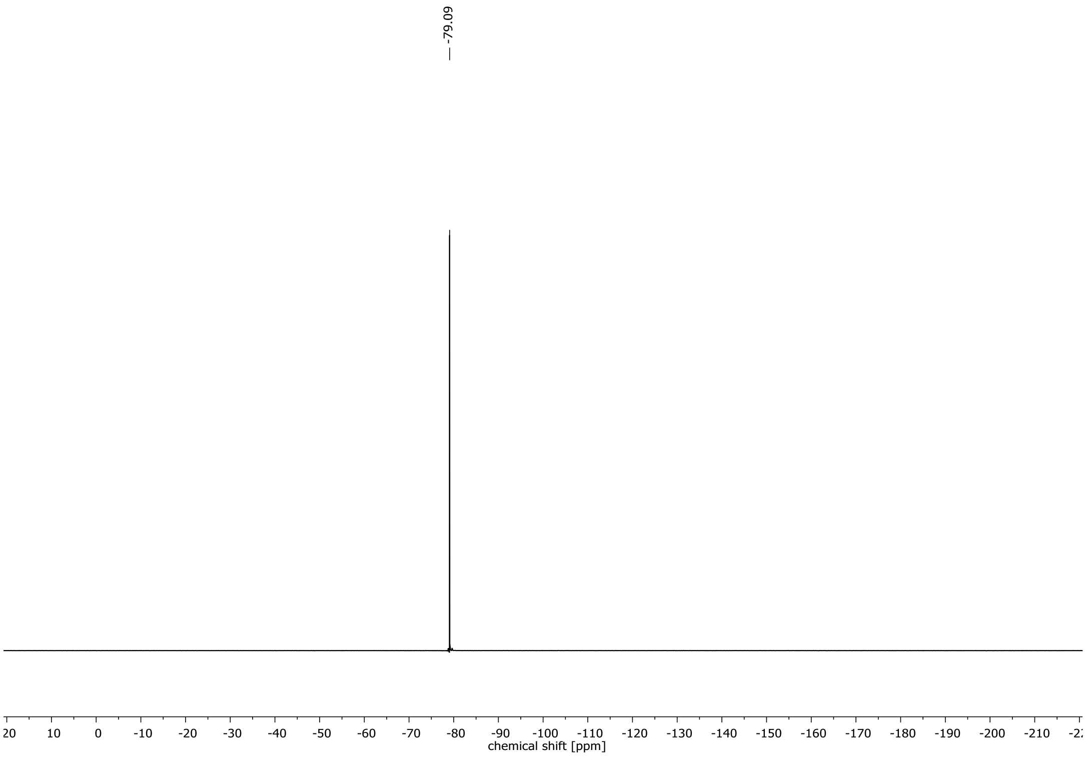

Figure S74. ^{19}F NMR spectrum of **7a^{OTf}** (fraction 1) (376 MHz, acetonitrile-d_3).

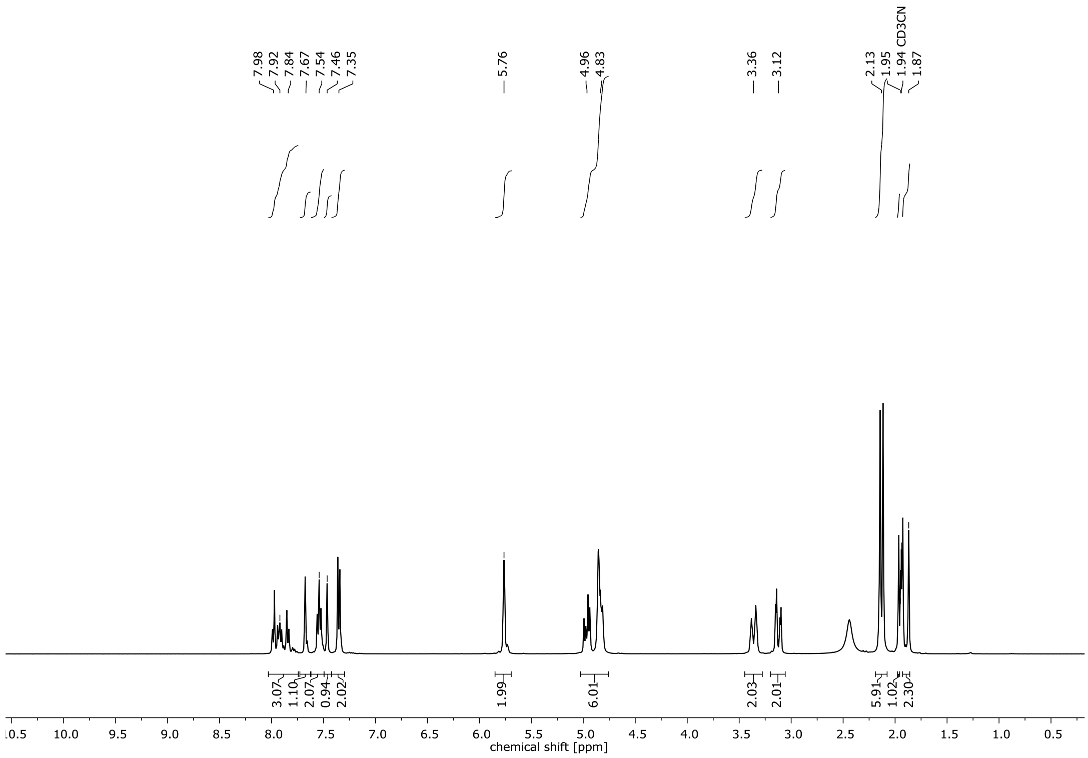

Figure S75. ^{1}H NMR spectrum of **7a^{OTf}** (fraction 2) (400 MHz, acetonitrile-d_3).

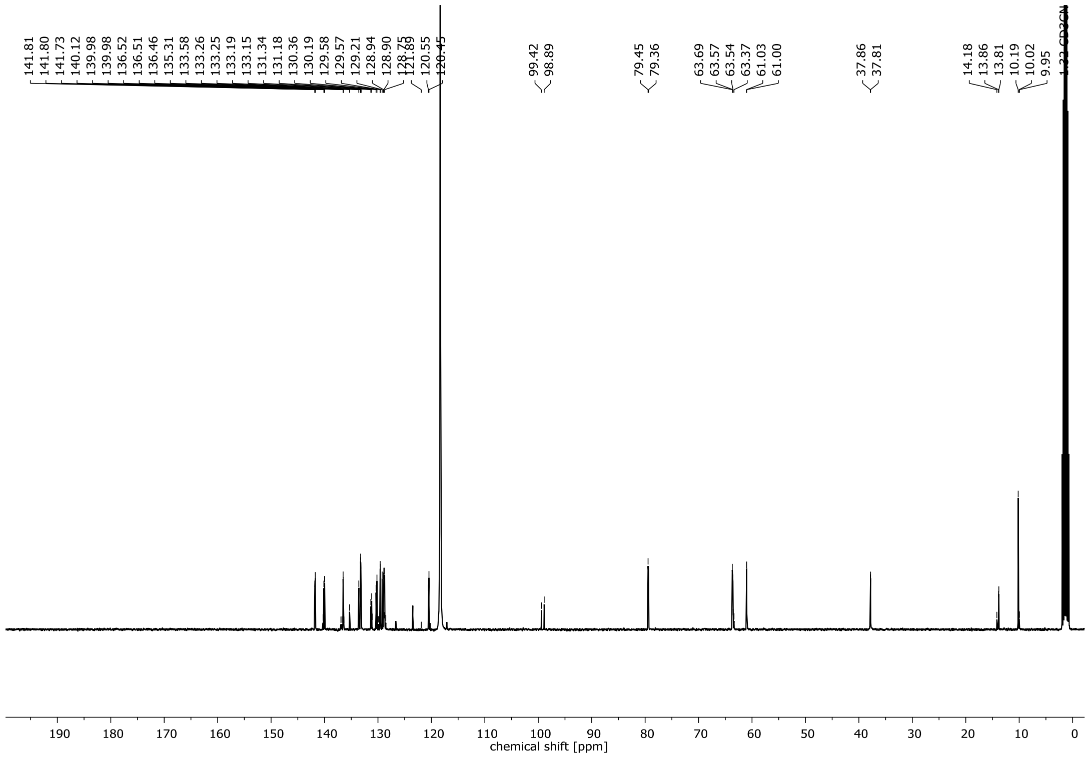

Figure S76. ^{13}C NMR spectrum of **7a^{OTf}** (fraction 2) (101 MHz, acetonitrile-d_3).

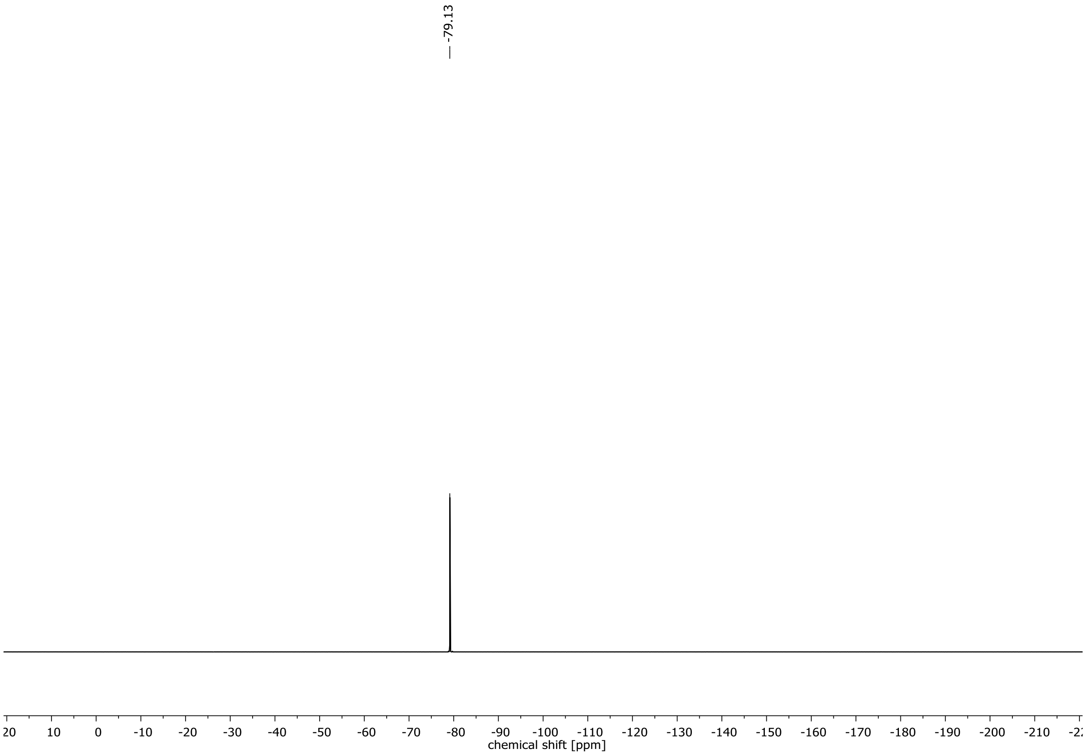

Figure S77. ^{19}F NMR spectrum of **7a^{OTf}** (fraction 2) (376 MHz, acetonitrile-d_3).

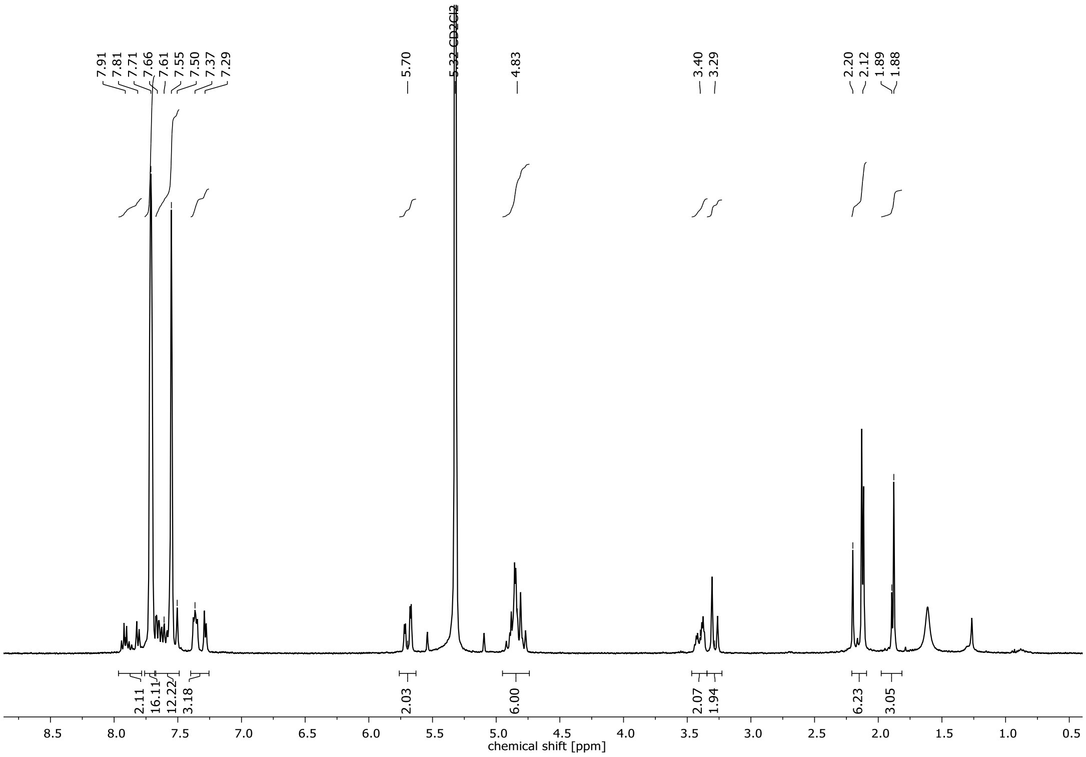

Figure S78. ^{1}H NMR spectrum of **7a^{BArF}** (fraction 1) (400 MHz, DCM-d_2).

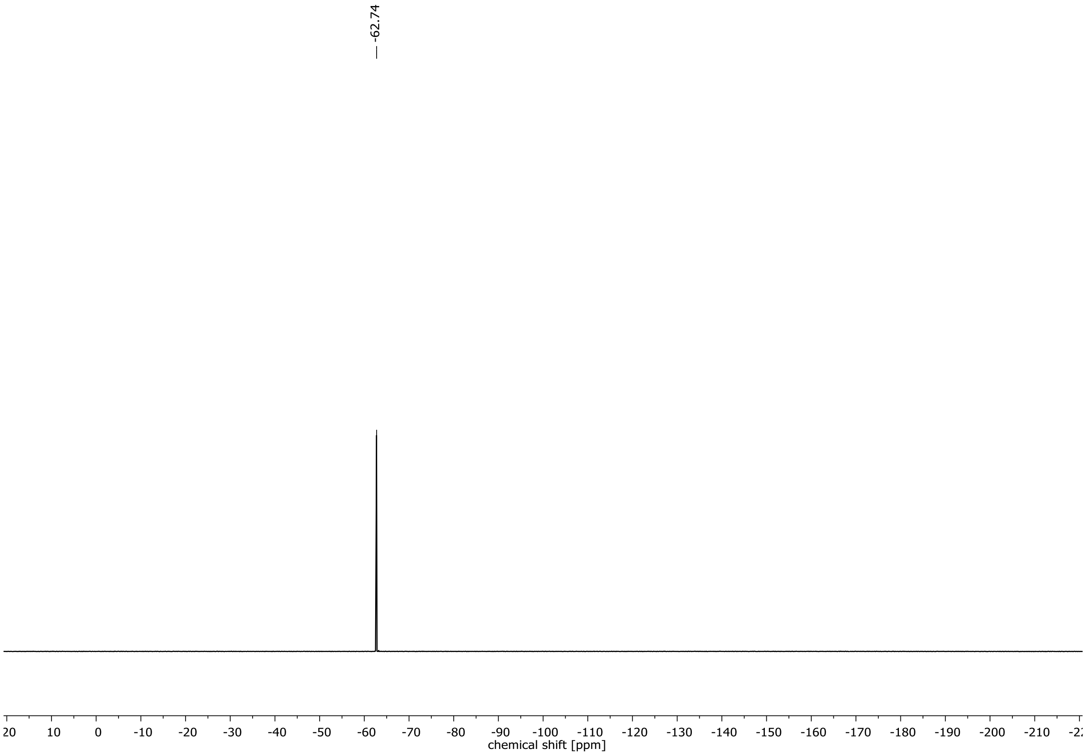

Figure S79. ^{19}F NMR spectrum of **7a$^{\mathbf{BArF}}$** (fraction 1) (376 MHz, DCM-d_2).

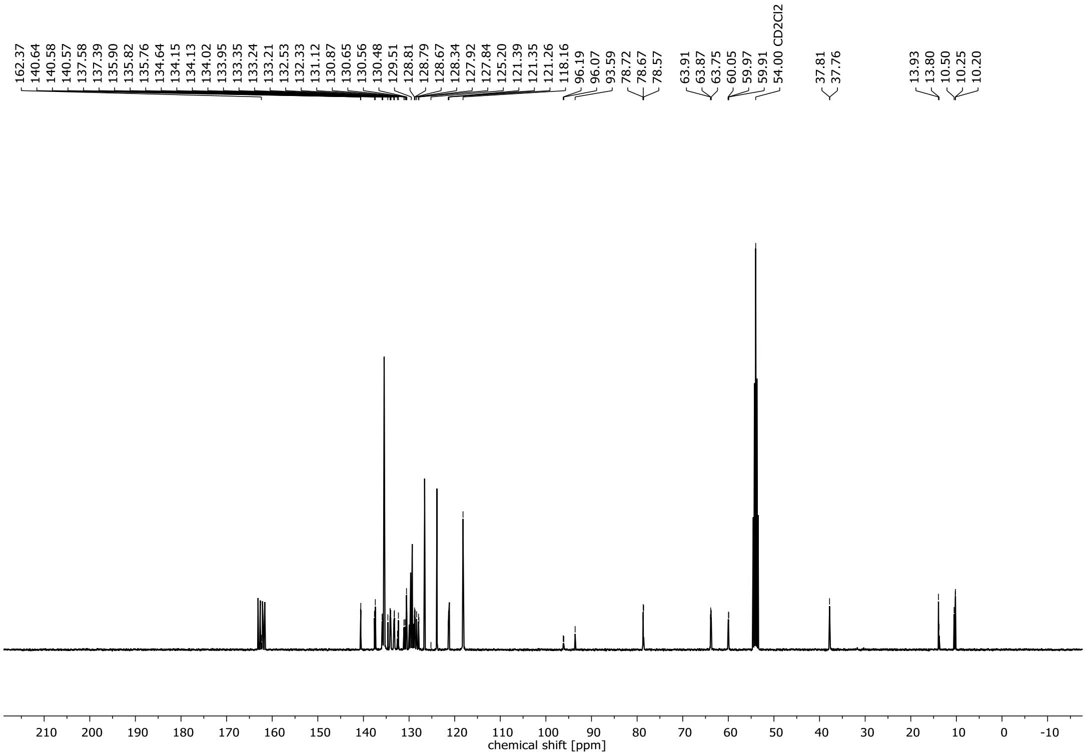

Figure S80. ^{13}C NMR spectrum of **7a$^{\mathbf{BArF}}$** (fraction 1) (101 MHz, DCM-d_2).

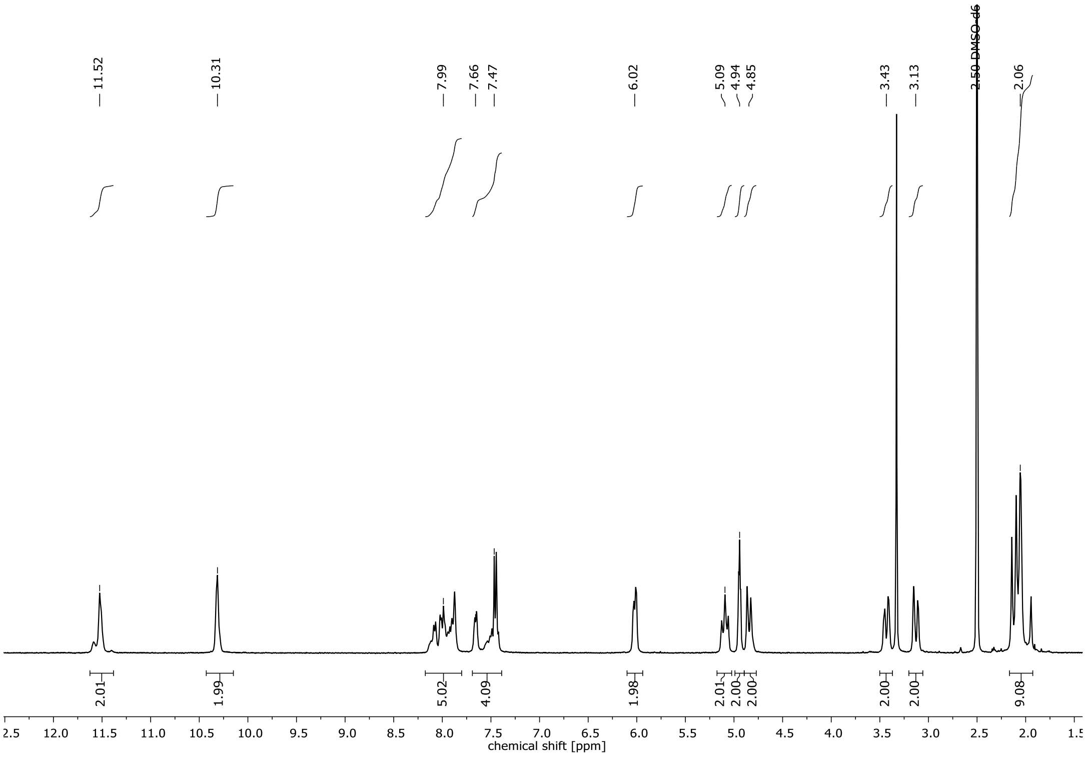

Figure S81. ^{1}H NMR spectrum of **5b^{Cl}** (400 MHz, DMSO-d_6).

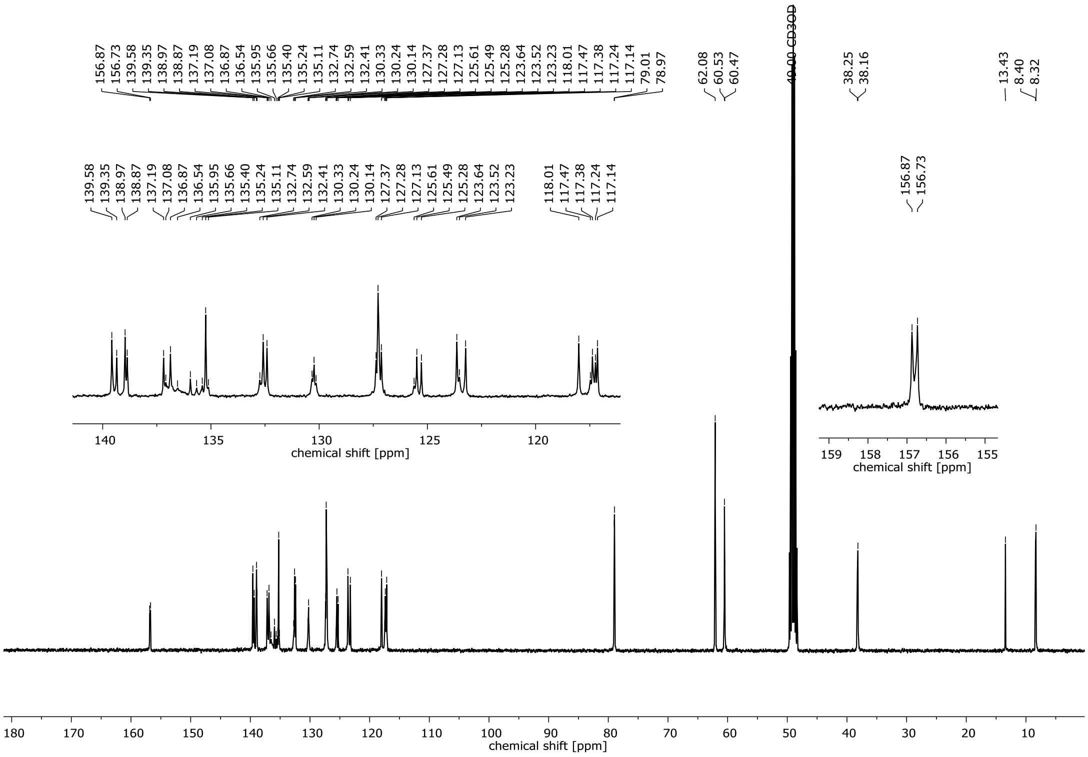

Figure S82. ^{13}C {^{1}H, ^{19}F} NMR spectrum of **5b^{Cl}** (101 MHz, methanol-d_4).

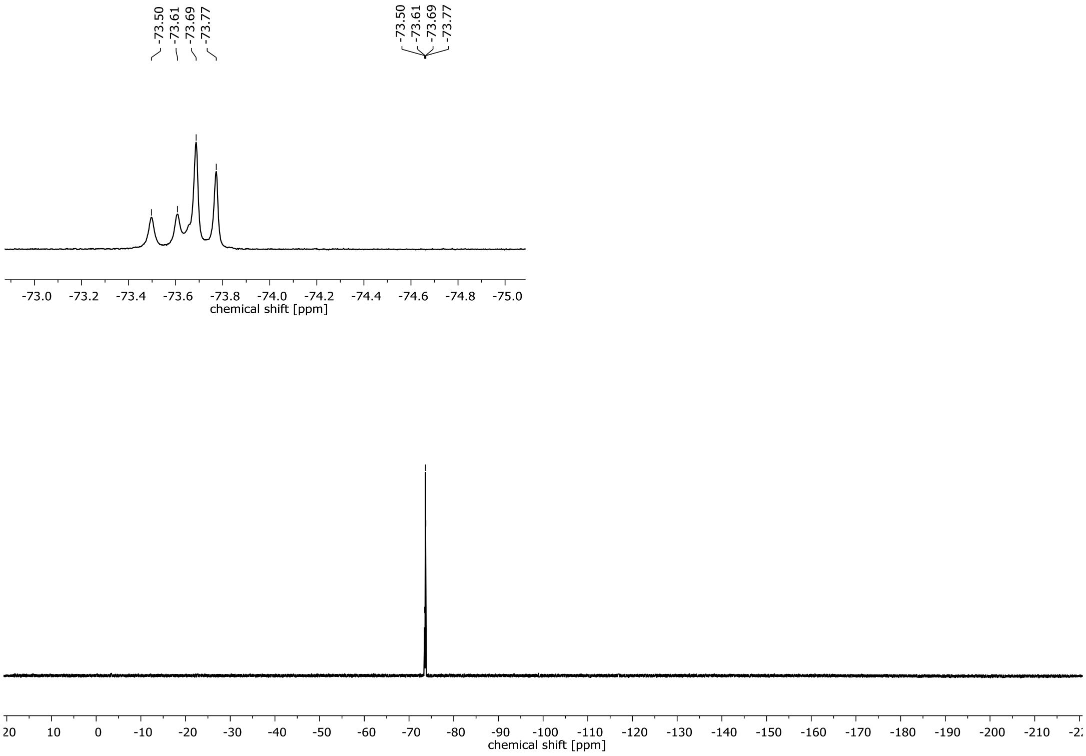

Figure S83. ^{19}F NMR spectrum of **5b$^{\mathbf{Cl}}$** (376 MHz, DMSO-d_6).

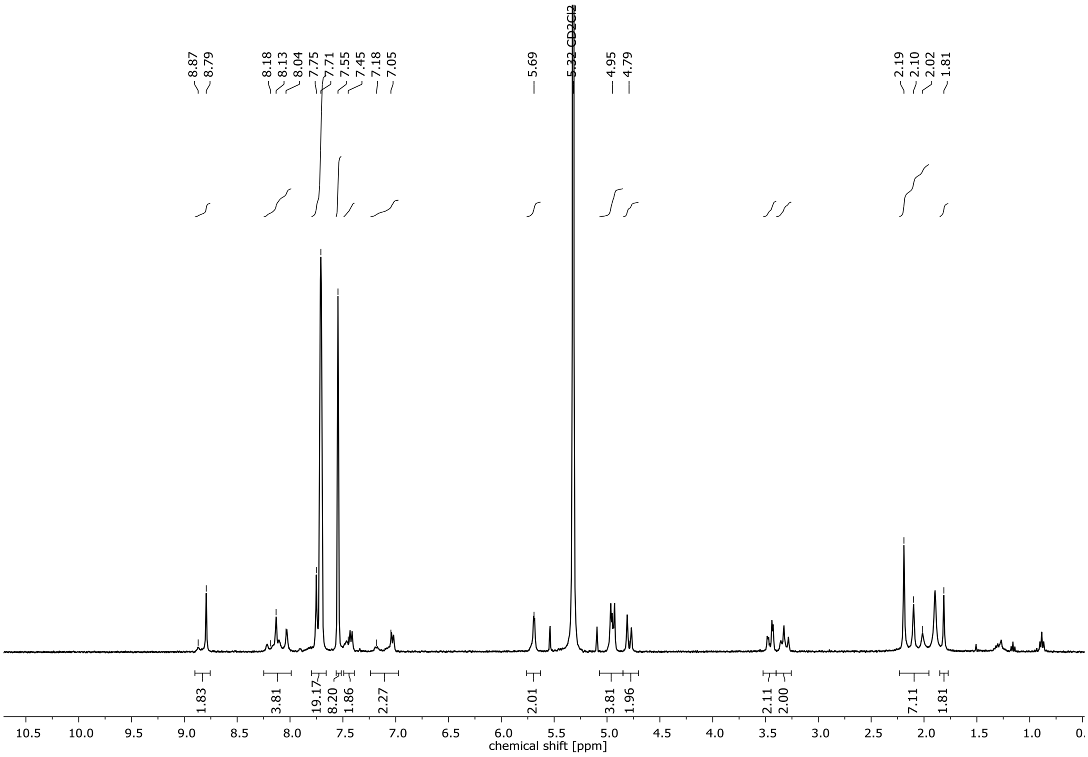

Figure S84. ^{1}H NMR spectrum of **5b$^{\mathbf{BArF}}$** (400 MHz, DCM-d_2).

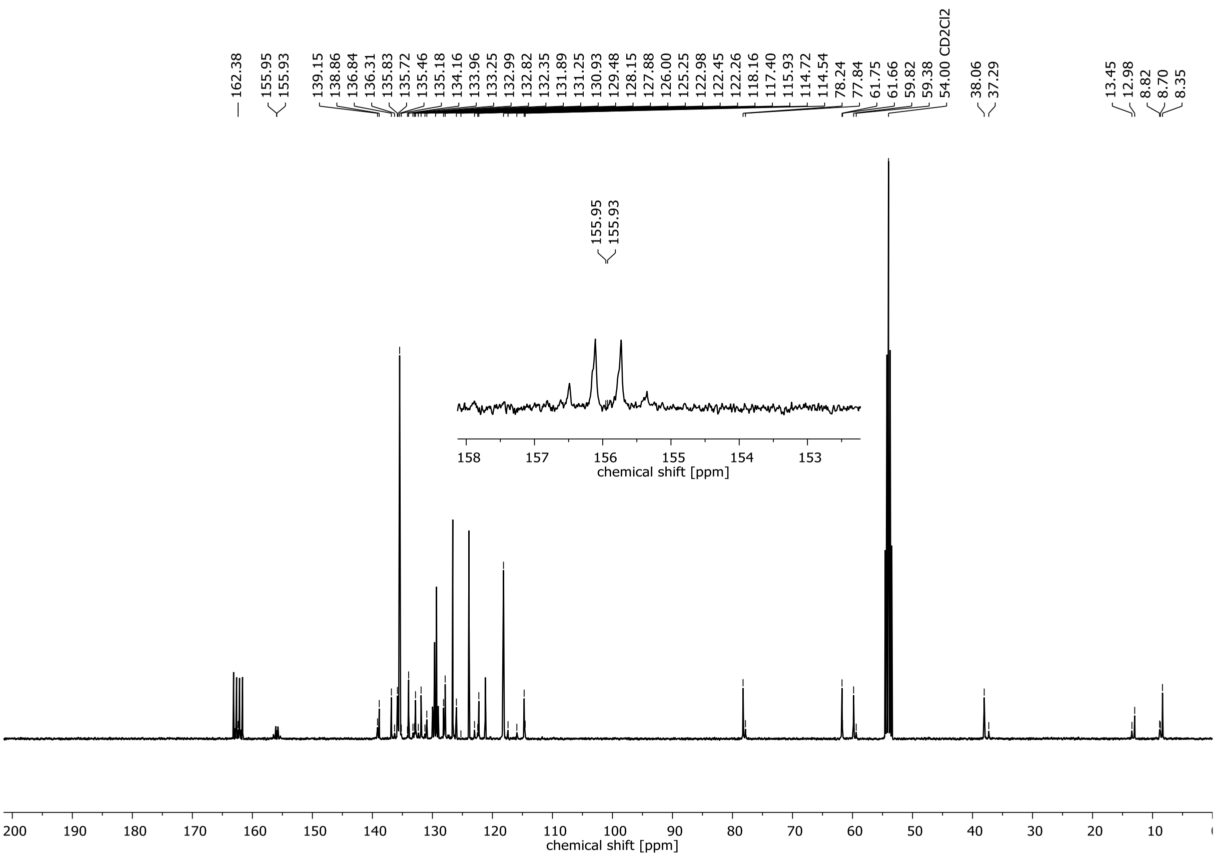

Figure S85. ^{13}C NMR spectrum of **5b**$^{\mathbf{BArF}}$ (101 MHz, DCM-d_2).

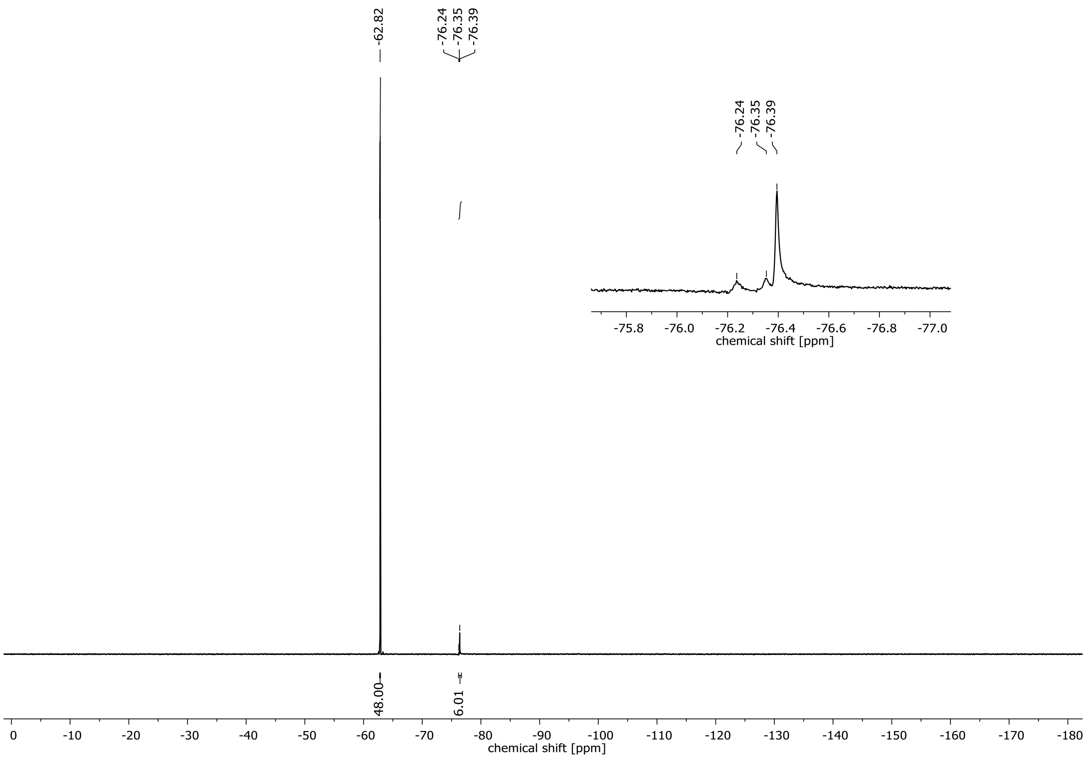

Figure S86. ^{19}F NMR spectrum of **5b**$^{\mathbf{BArF}}$ (376 MHz, DCM-d_2).

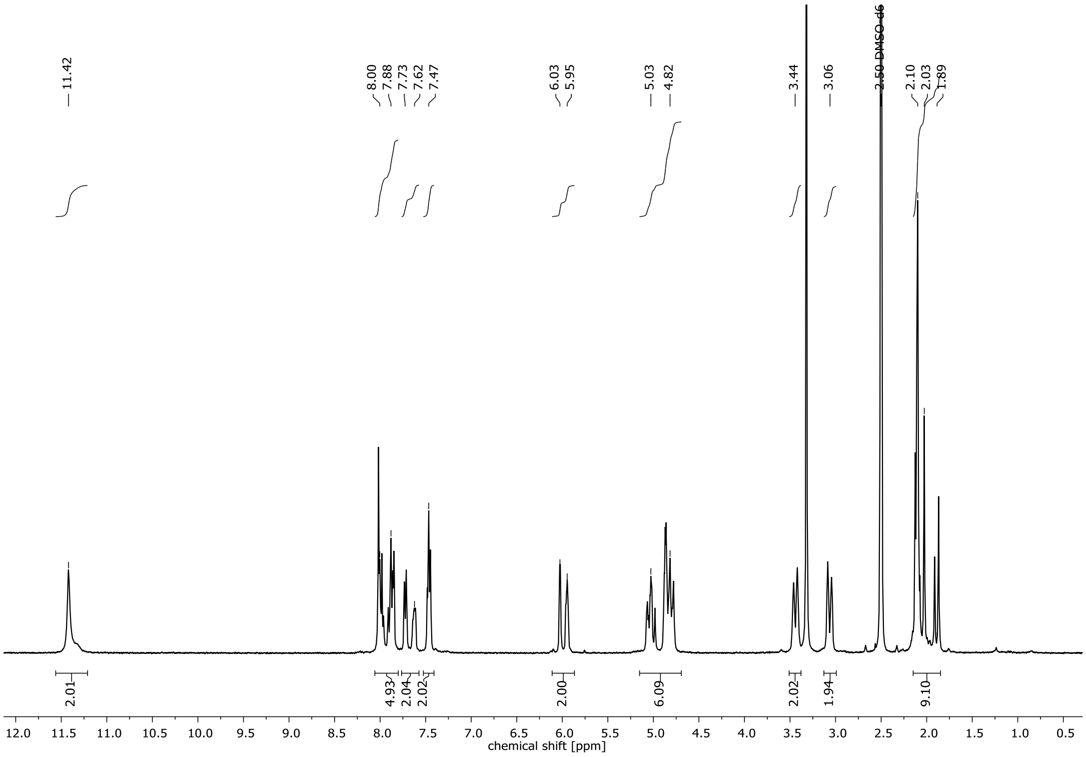

Figure S87. ^{1}H NMR spectrum of **7b^{Cl}** (400 MHz, DMSO-d_6).

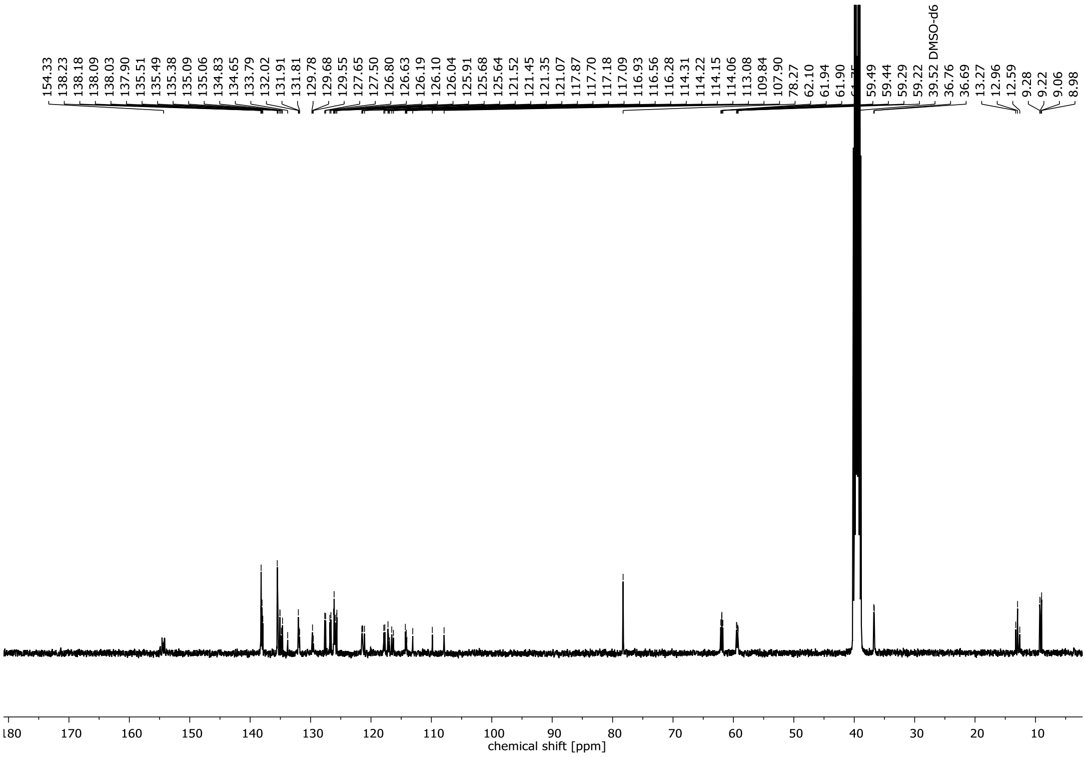

Figure S88. ^{13}C NMR spectrum of **7b^{Cl}** (101 MHz, DMSO-d_6).

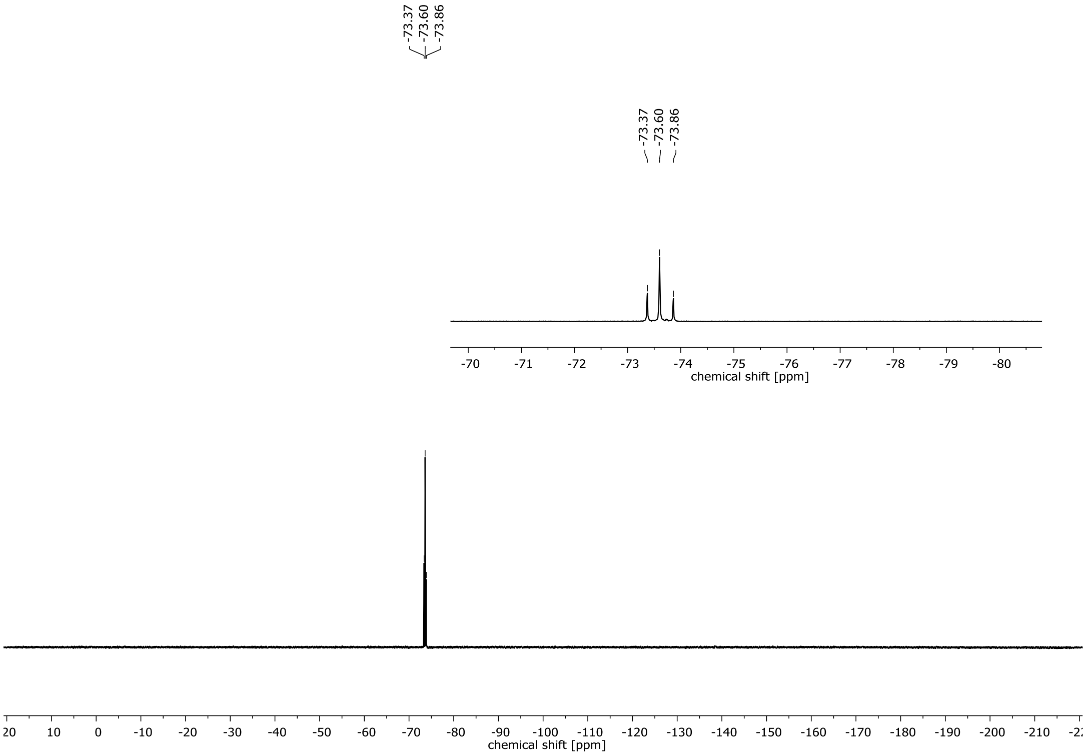

Figure S89. ^{19}F NMR spectrum of **7b^{Cl}** (376 MHz, DMSO-d_6).

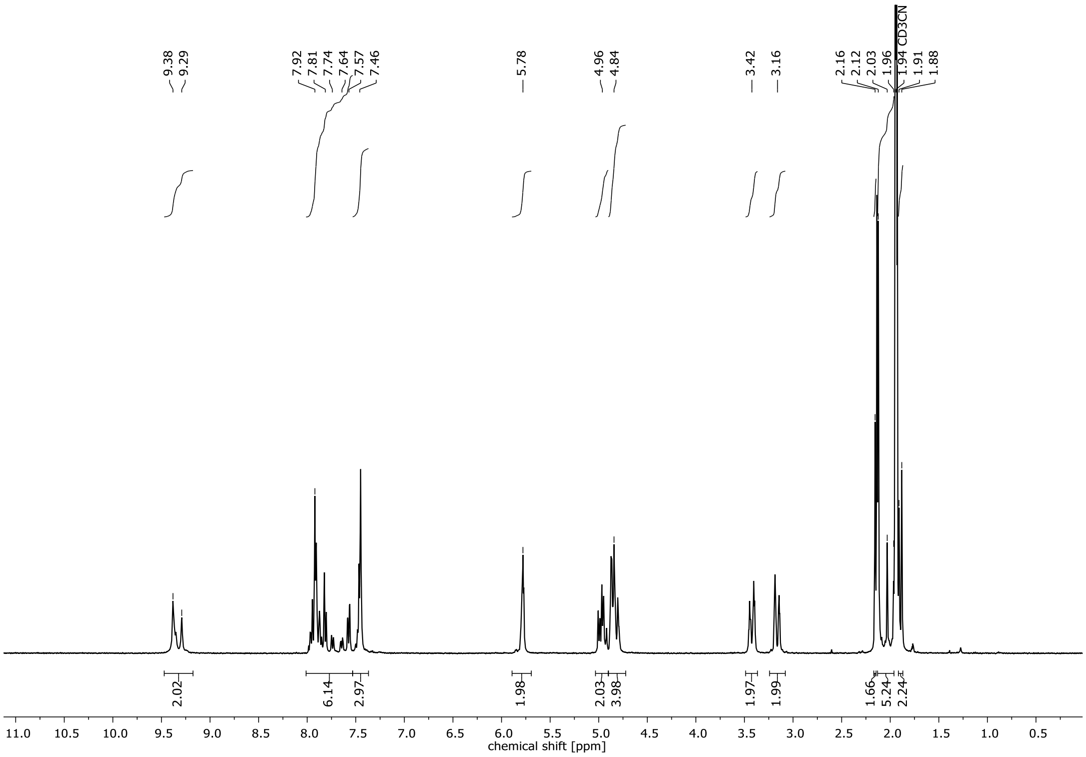

Figure S90. ^{1}H NMR spectrum of **7b^{OTf}** (fraction 1) (400 MHz, acetonitrile-d_3).

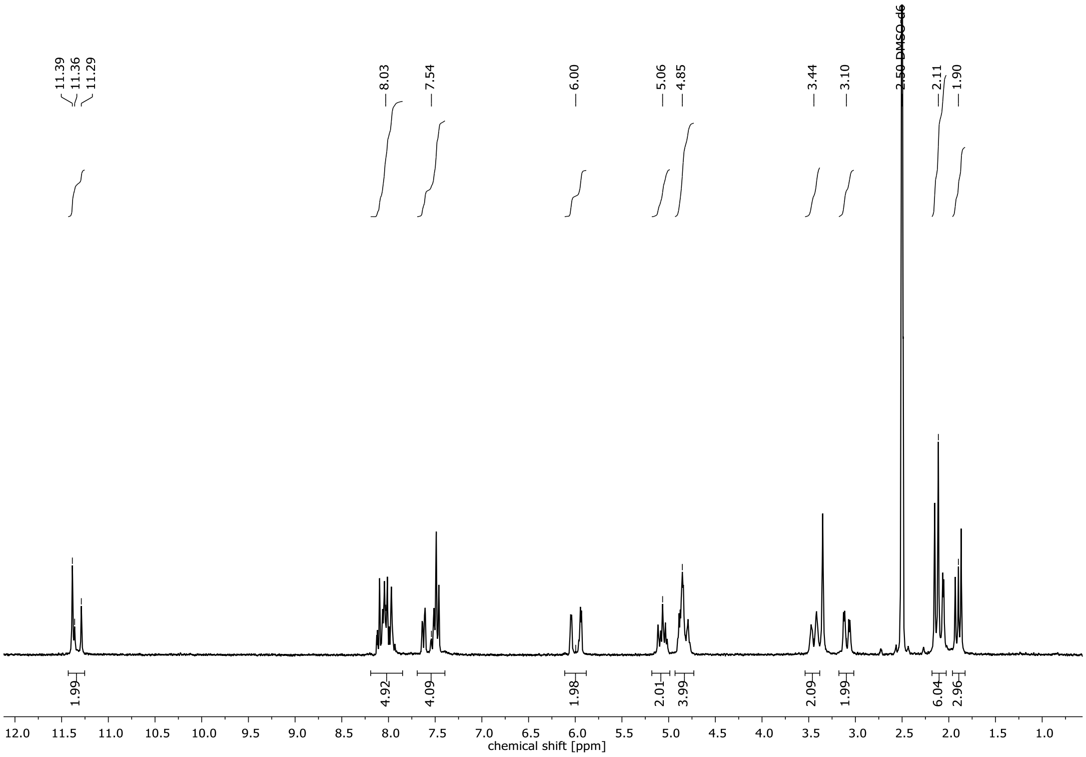

Figure S91. ^{1}H NMR spectrum of $\mathbf{7b^{OTf}}$ (fraction 1) (300 MHz, DMSO-d_6).

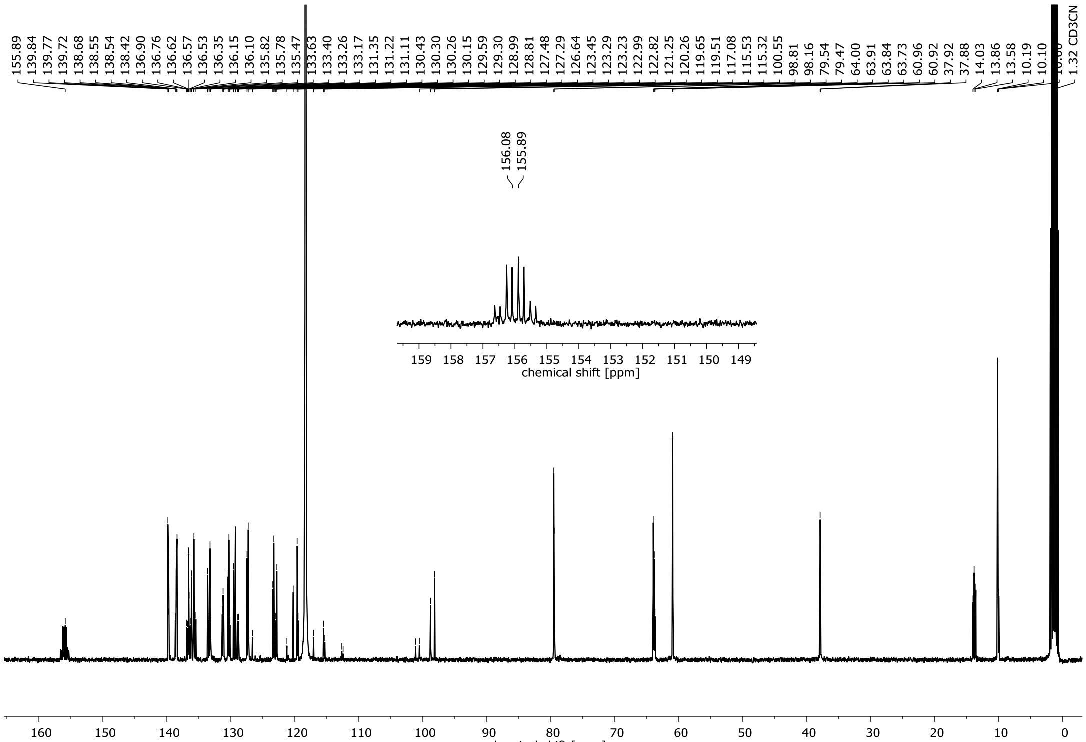

Figure S92. ^{13}C NMR spectrum of $\mathbf{7b^{OTf}}$ (fraction 1) (101 MHz, acetonitrile-d_3).

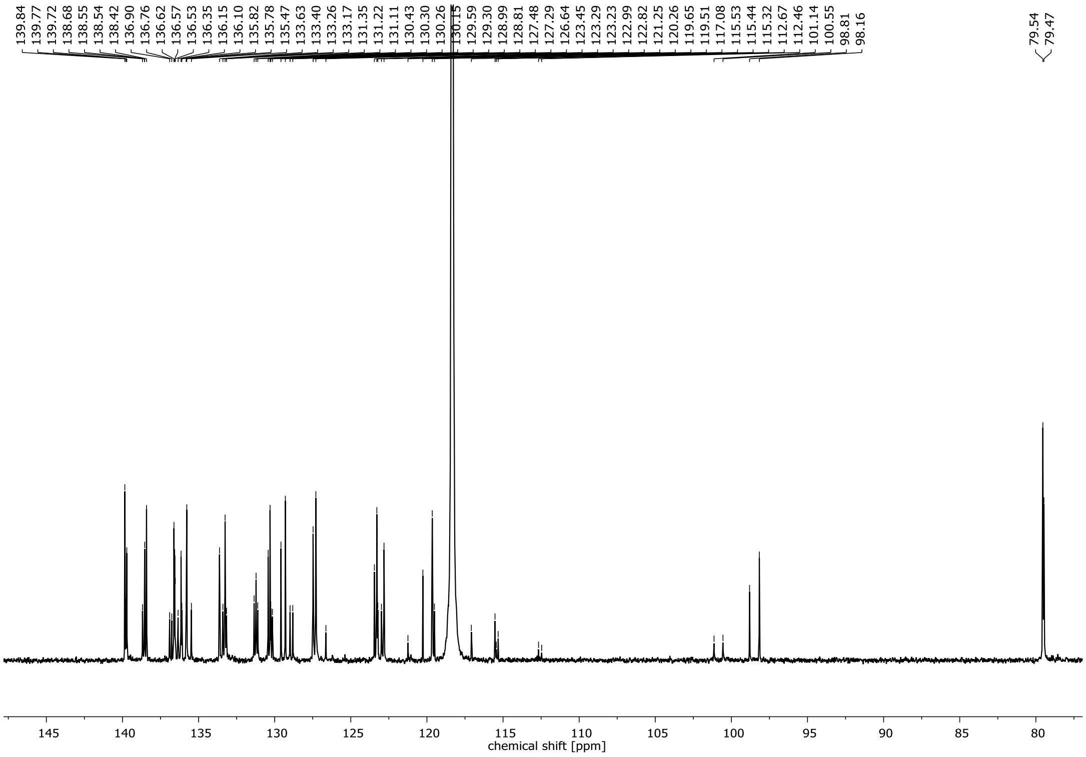

Figure S93. ^{13}C NMR spectrum of **7b$^{\mathbf{OTf}}$** (fraction 1), zoom into aromatic region (101 MHz, acetonitrile-d_3).

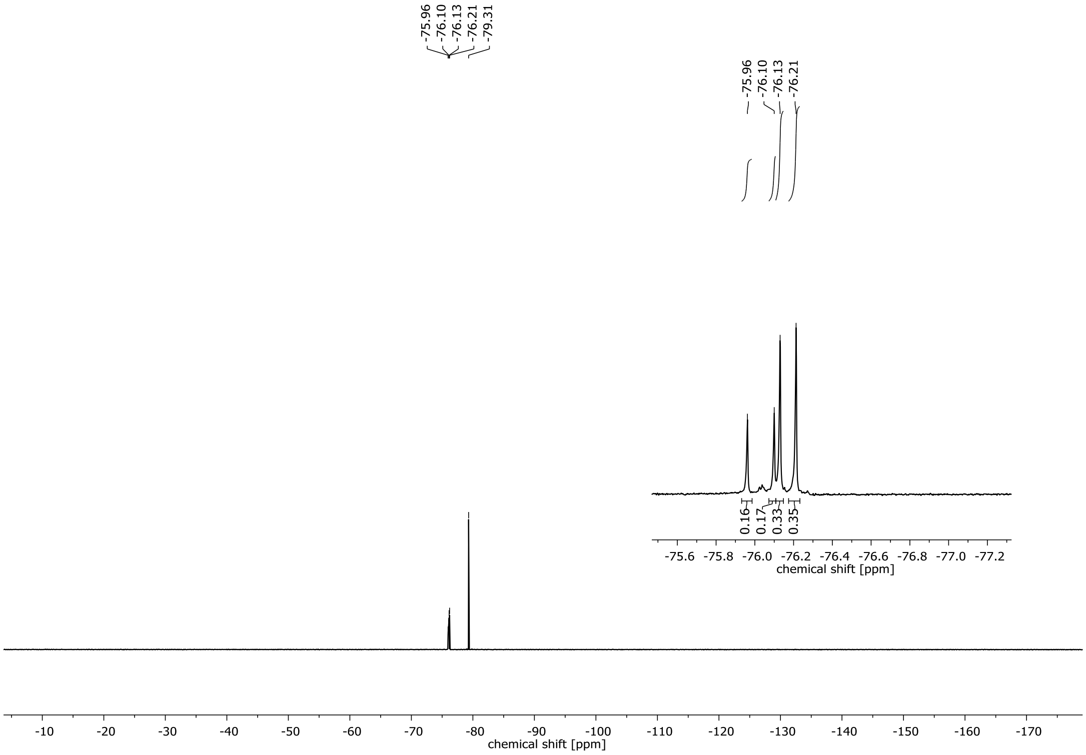

Figure S94. ^{19}F NMR spectrum of **7b$^{\mathbf{OTf}}$** (fraction 1) (376 MHz, acetonitrile-d_3).

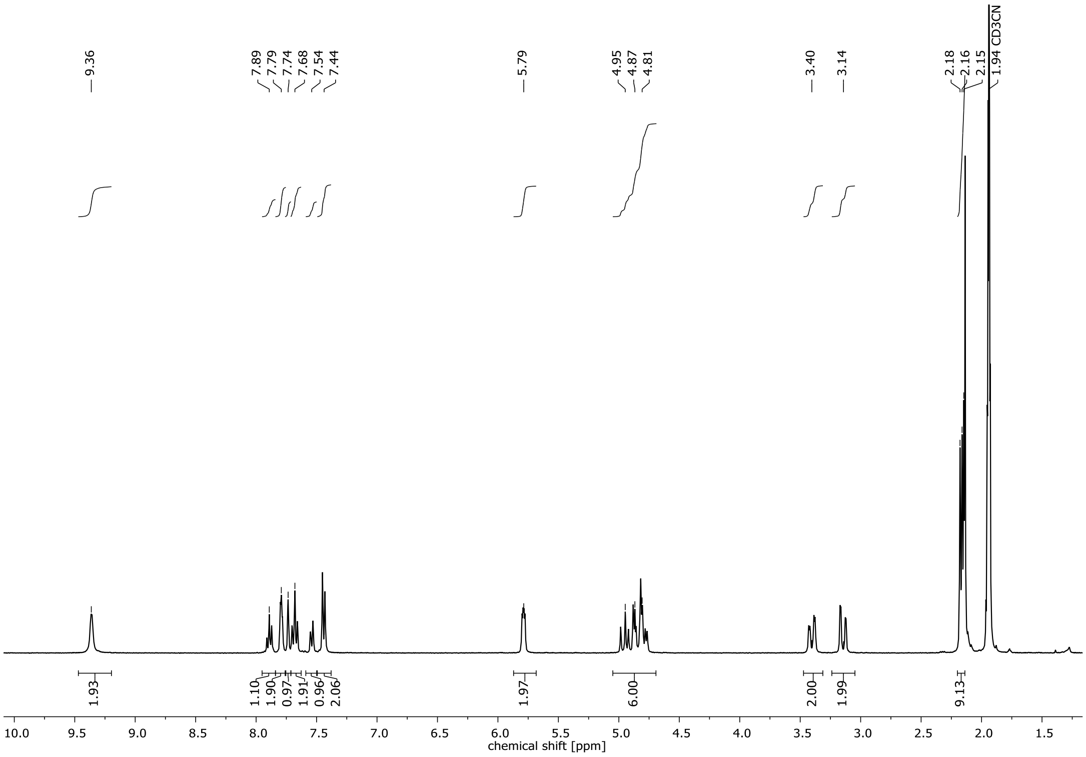

Figure S95. ^{1}H NMR spectrum of **7b^{OTf}** (fraction 2) (400 MHz, acetonitrile-d_3).

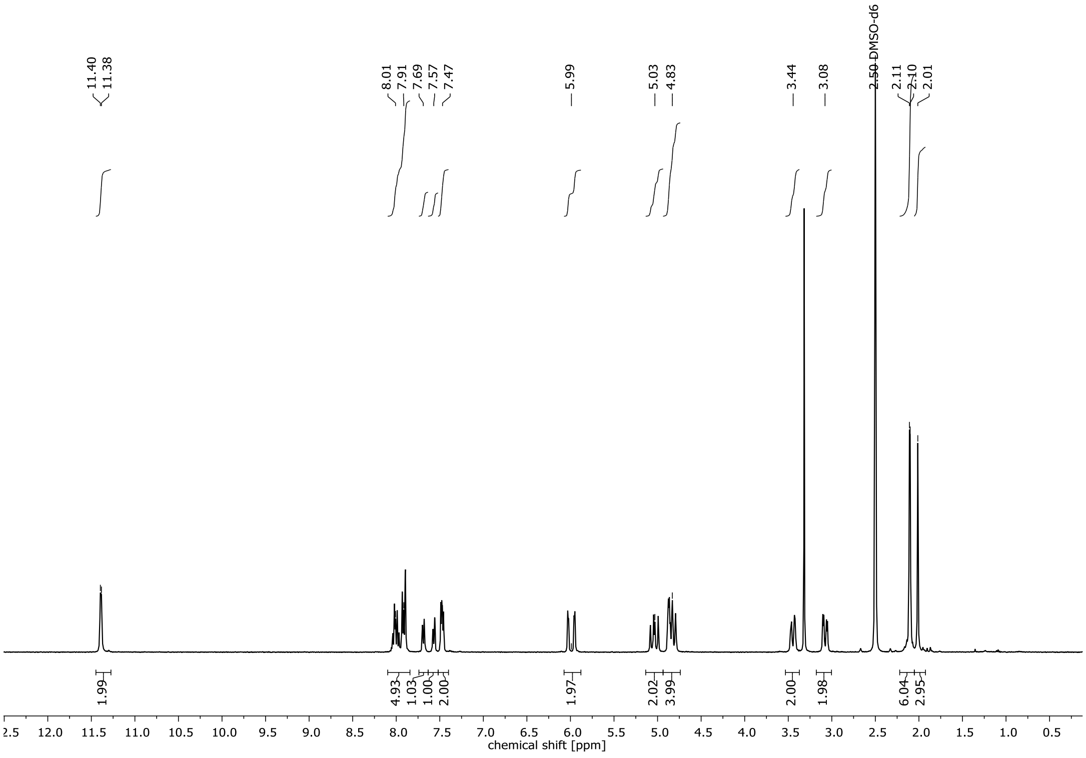

Figure S96. ^{1}H NMR spectrum of **7b^{OTf}** (fraction 2) (400 MHz, DMSO-d_6).

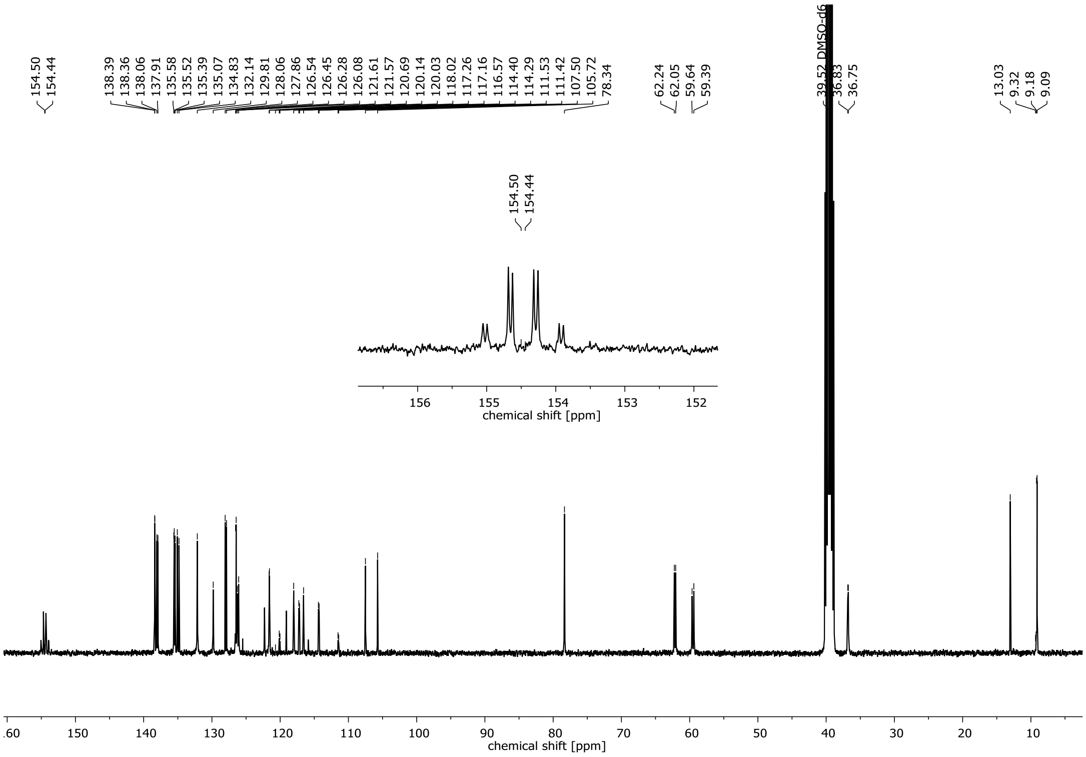

Figure S97. ^{13}C NMR spectrum of **7b^{OTf}** (fraction 2) (101 MHz, DMSO-d_6).

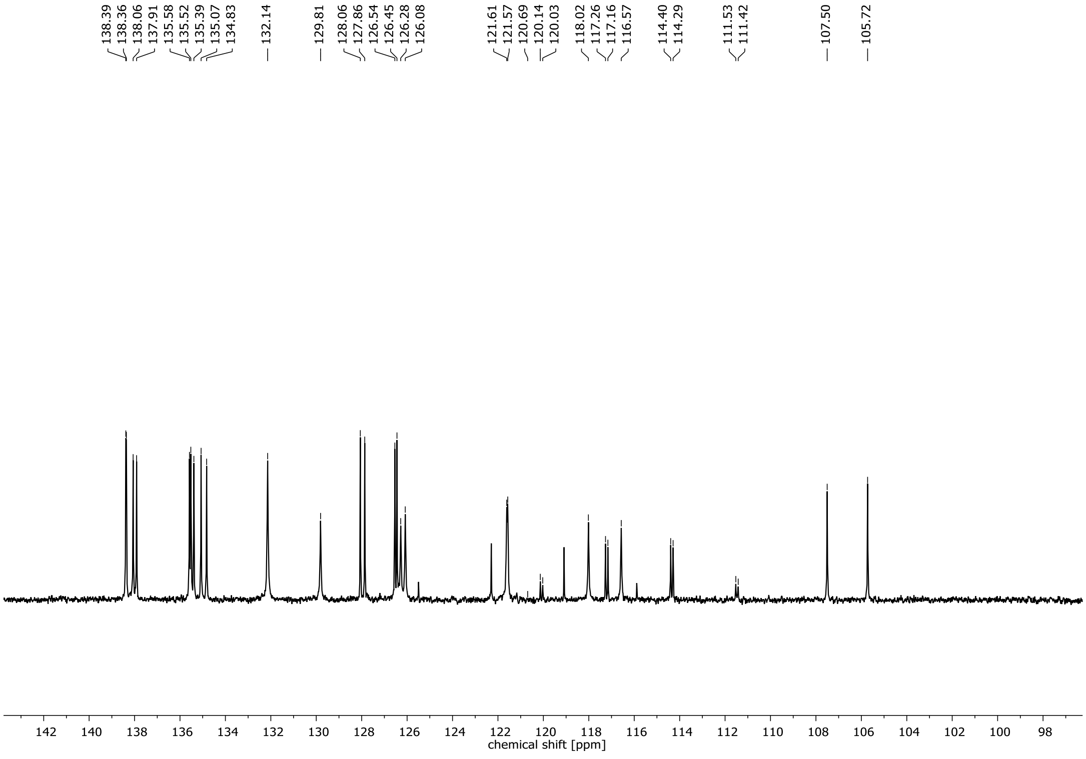

Figure S98. ^{13}C NMR spectrum of **7b^{OTf}** (fraction 2), zoom into aromatic region (101 MHz, acetonitrile-d_3).

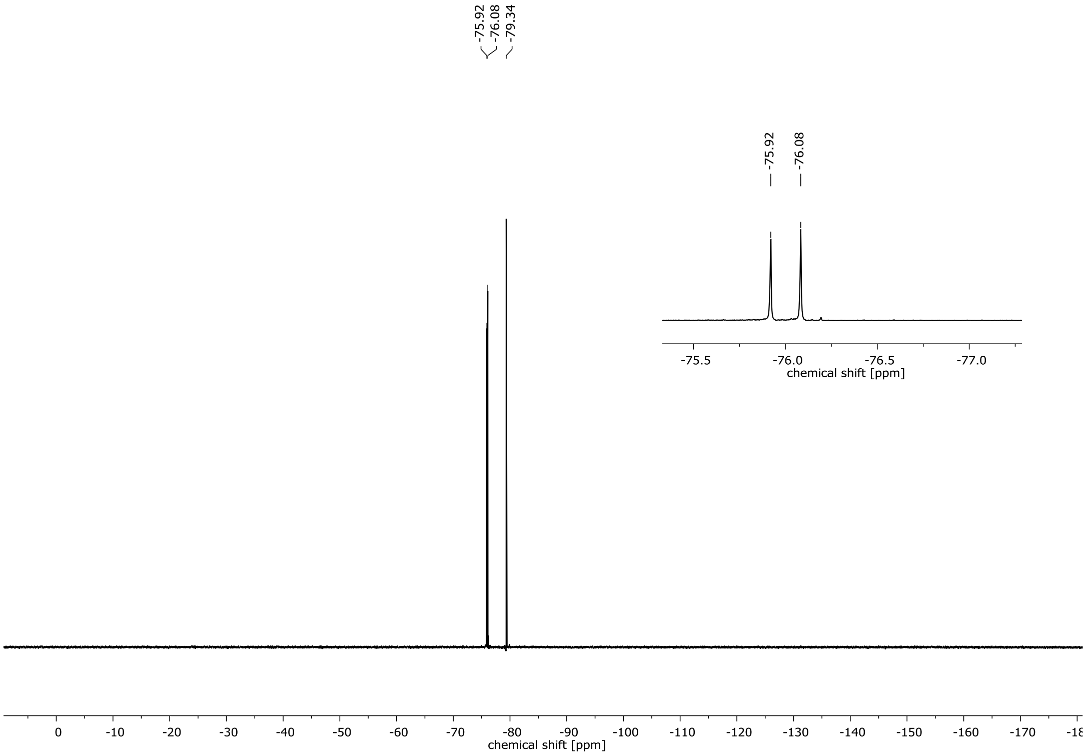

Figure S99. ^{19}F NMR spectrum of **7b^{OTf}** (fraction 2) (376 MHz, acetonitrile-d_3).

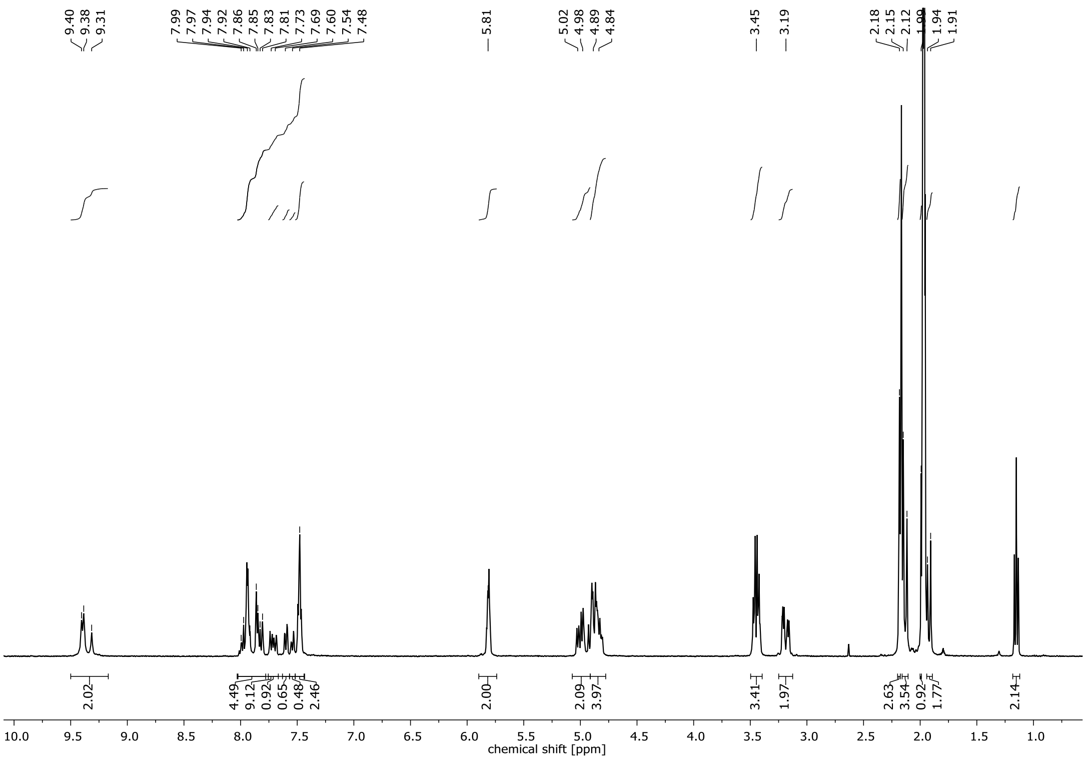

Figure S100. ^{1}H NMR spectrum of **7b^{OTf}** (atropisomer mixture, before separation) (400 MHz, acetonitrile-d_3). Residual diethyl ether is present (leads to deviating integral for signal at 3.5 ppm).

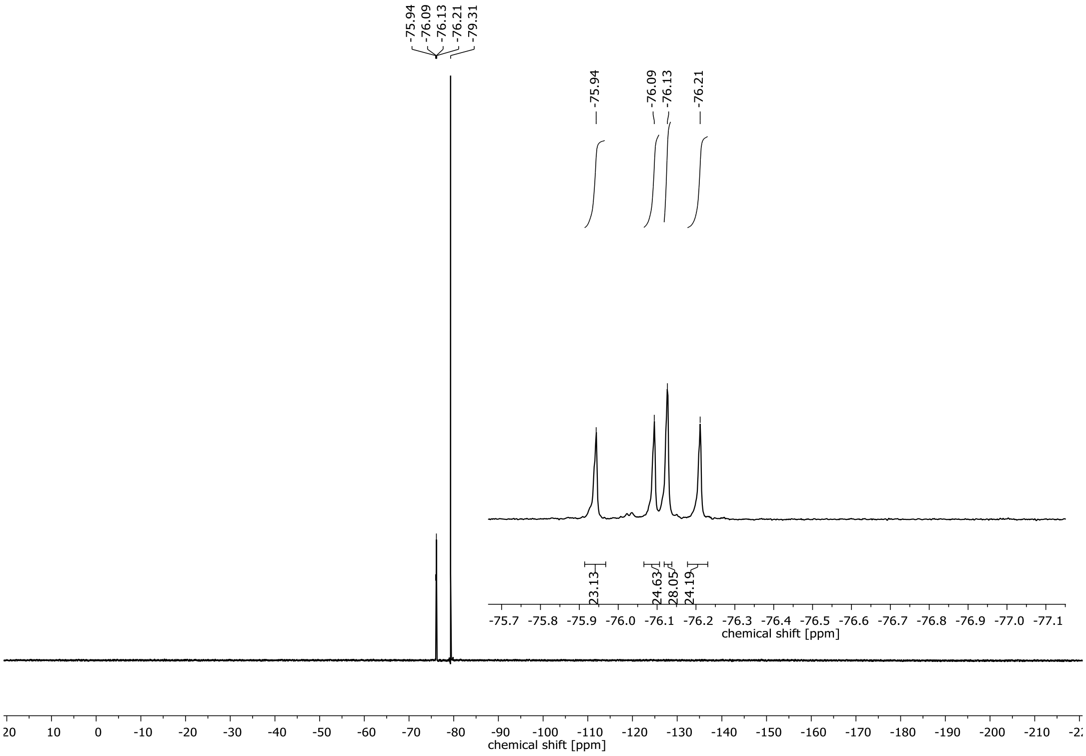

Figure S101. ^{19}F NMR spectrum of **7b^{OTf}** (atropisomer mixture, before separation) (376 MHz, acetonitrile-d_3).

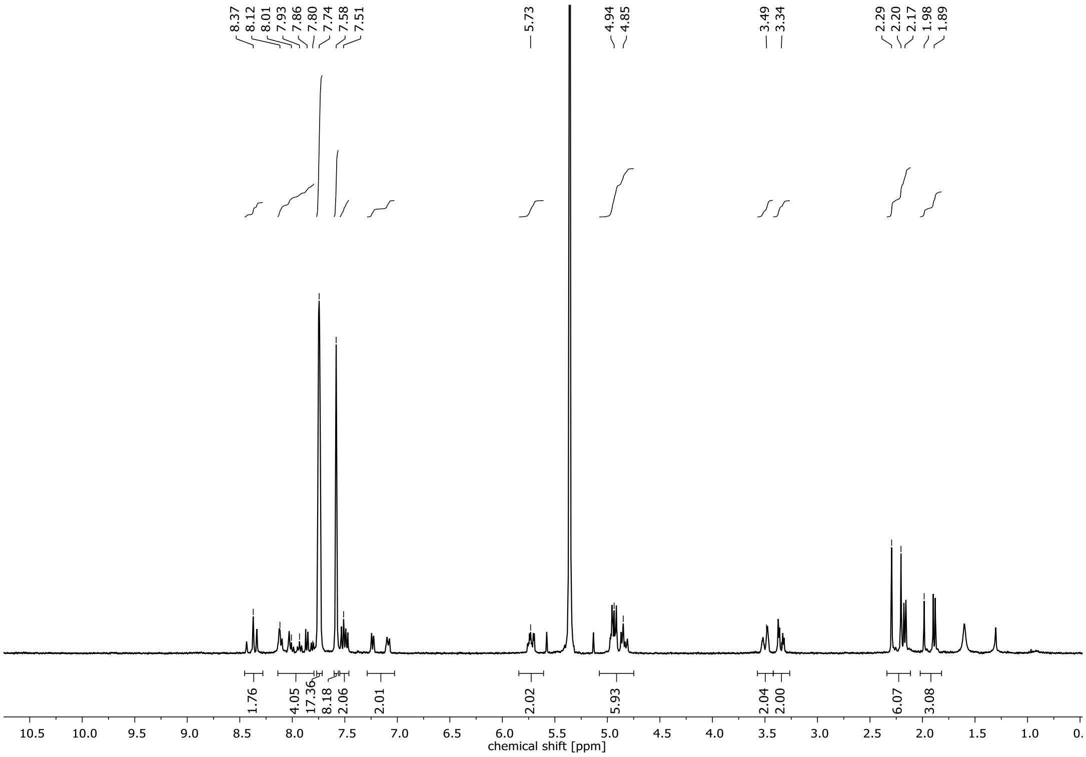

Figure S102. ^{1}H NMR spectrum of **7b^{BArF}** (fraction 1) (400 MHz, DCM-d_2).

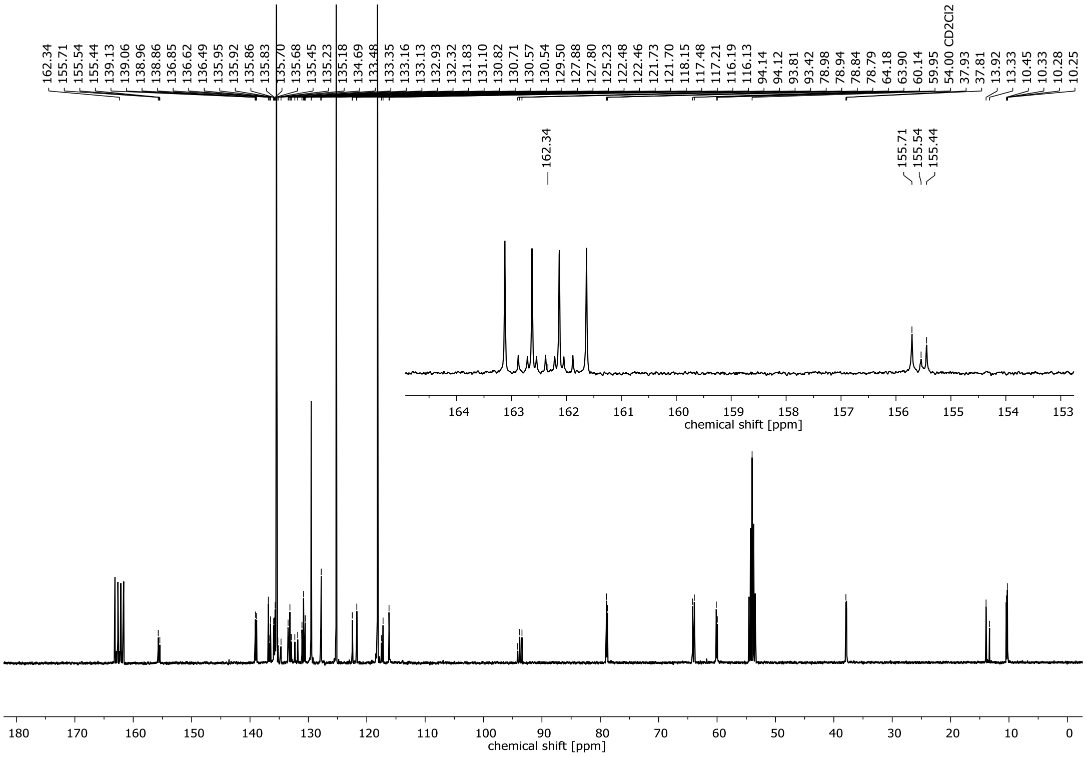

Figure S103. $^{13}C\ \{^{1}H,^{19}F\}$ NMR spectrum of **7b^{BArF}** (fraction 1) (101 MHz, DCM-d_2).

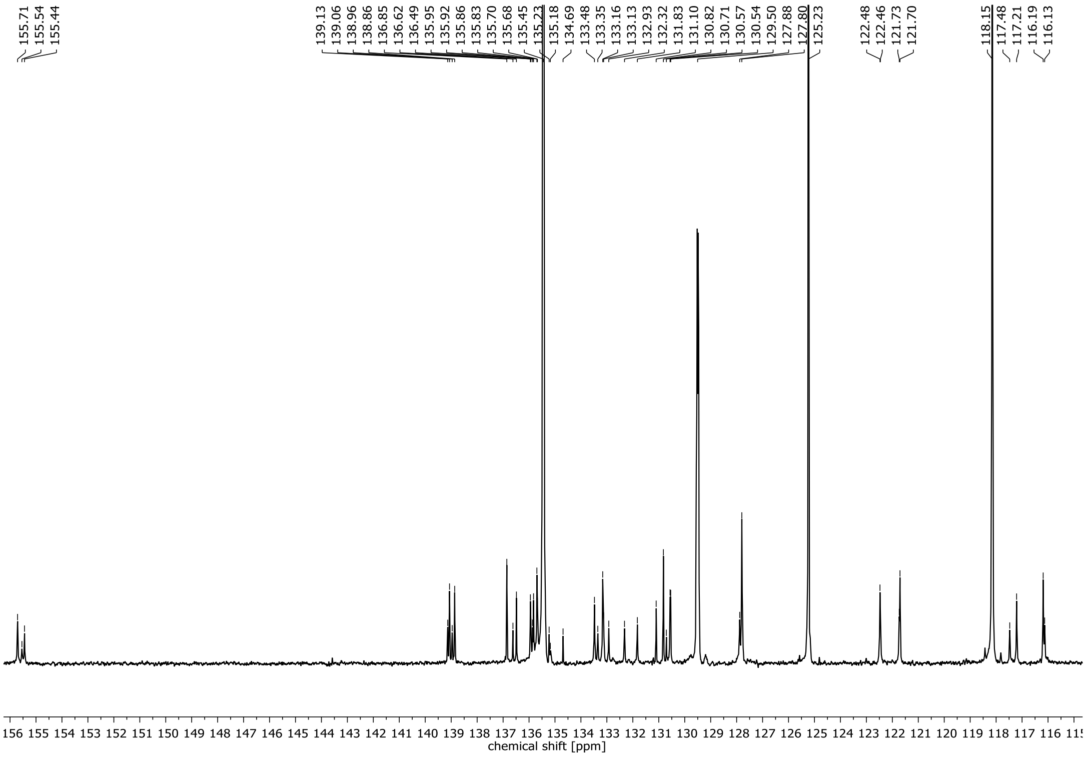

Figure S104. $^{13}C\ \{^{1}H,^{19}F\}$ NMR spectrum of **7b^{BArF}** (fraction 1), zoom into aromatic region (101 MHz, DCM-d_2).

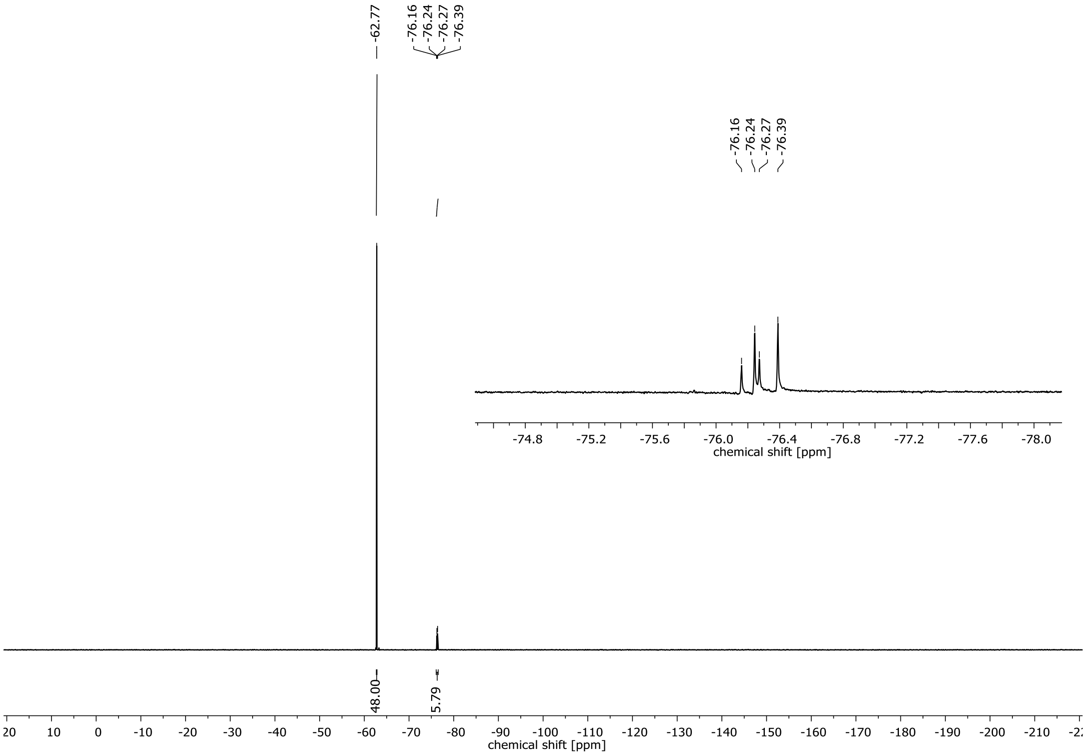

Figure S105. ^{19}F NMR spectrum of **7b^{BArF}** (fraction 1) (376 MHz, DCM-d_2).

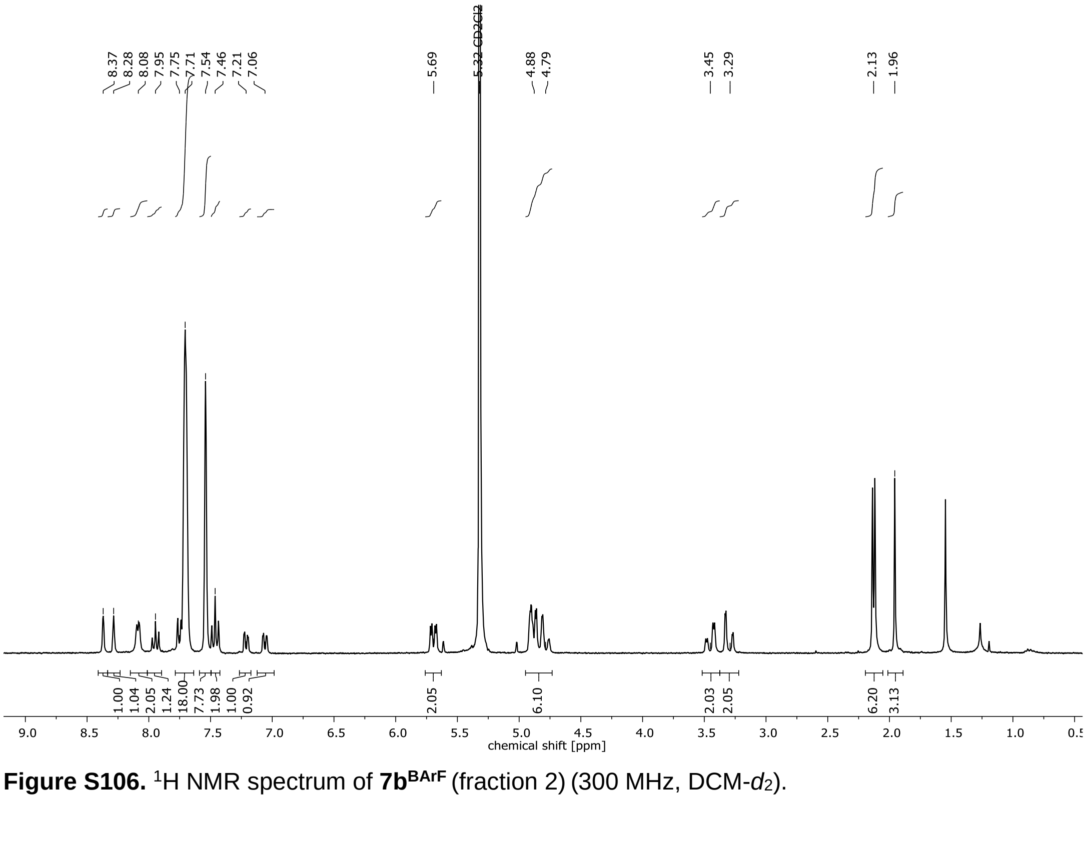

Figure S106. ^{1}H NMR spectrum of **7b^{BArF}** (fraction 2) (300 MHz, DCM-d_2).

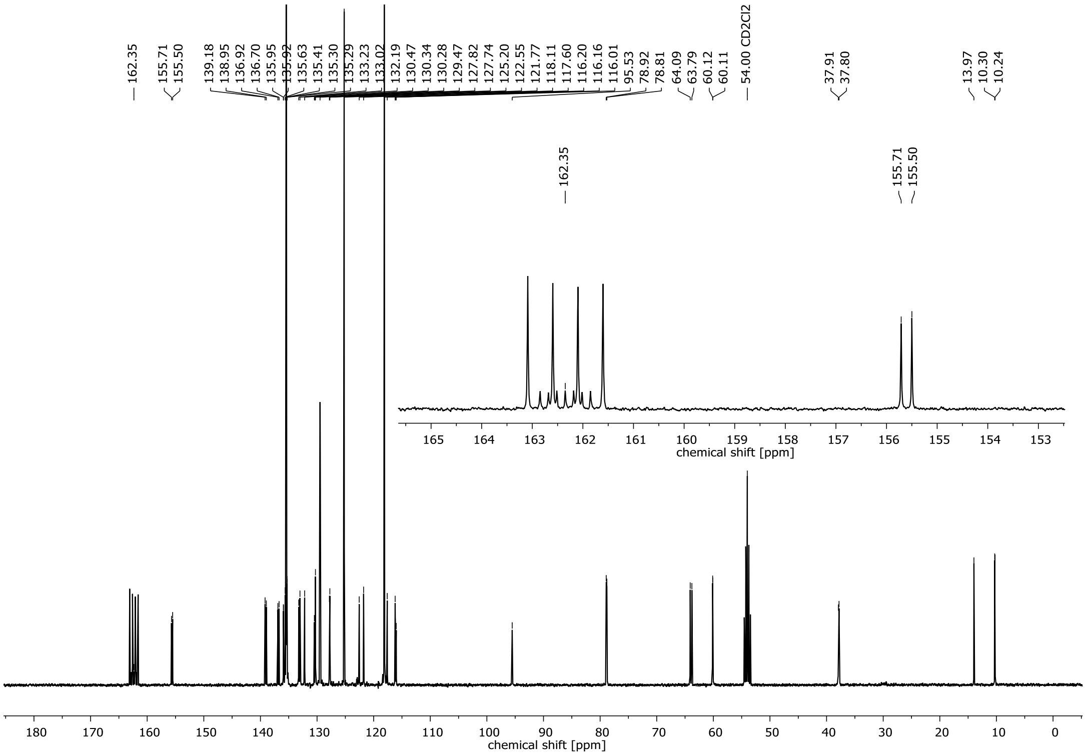

Figure S107. ^{13}C {^{1}H,^{19}F} NMR spectrum of **7b^{BArF}** (fraction 2) (101 MHz, DCM-d_2).

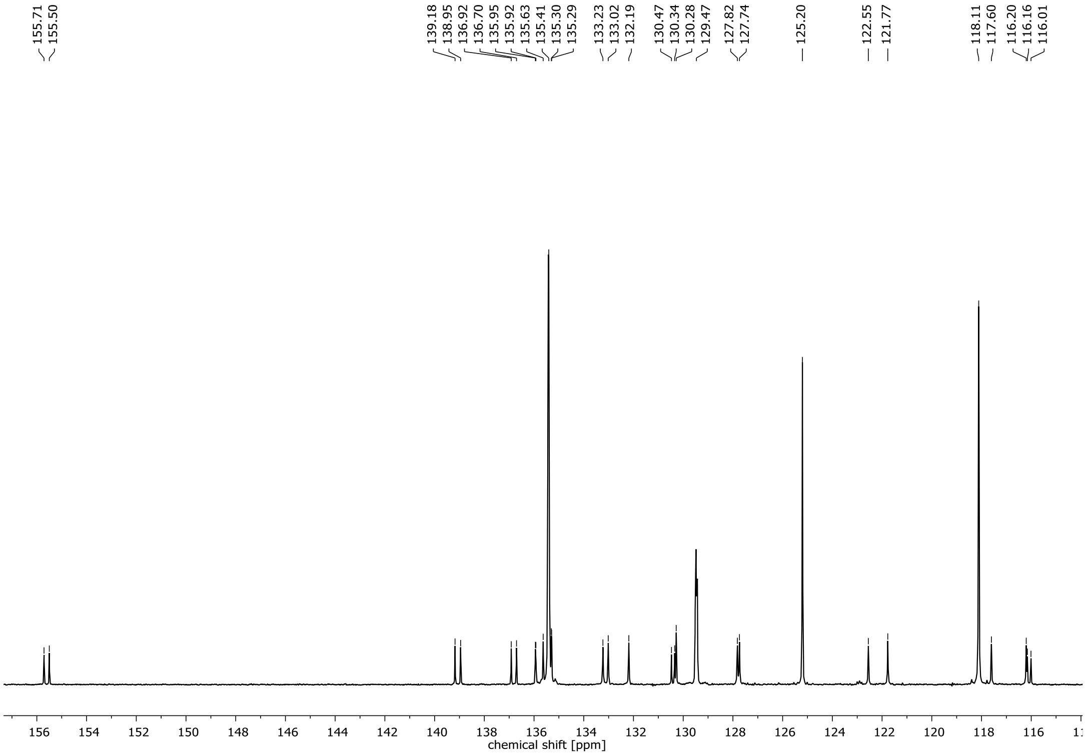

Figure S108. ^{13}C {^{1}H,^{19}F} NMR spectrum of **7b^{BArF}** (fraction 2), zoom into aromatic region (101 MHz, DCM-d_2).

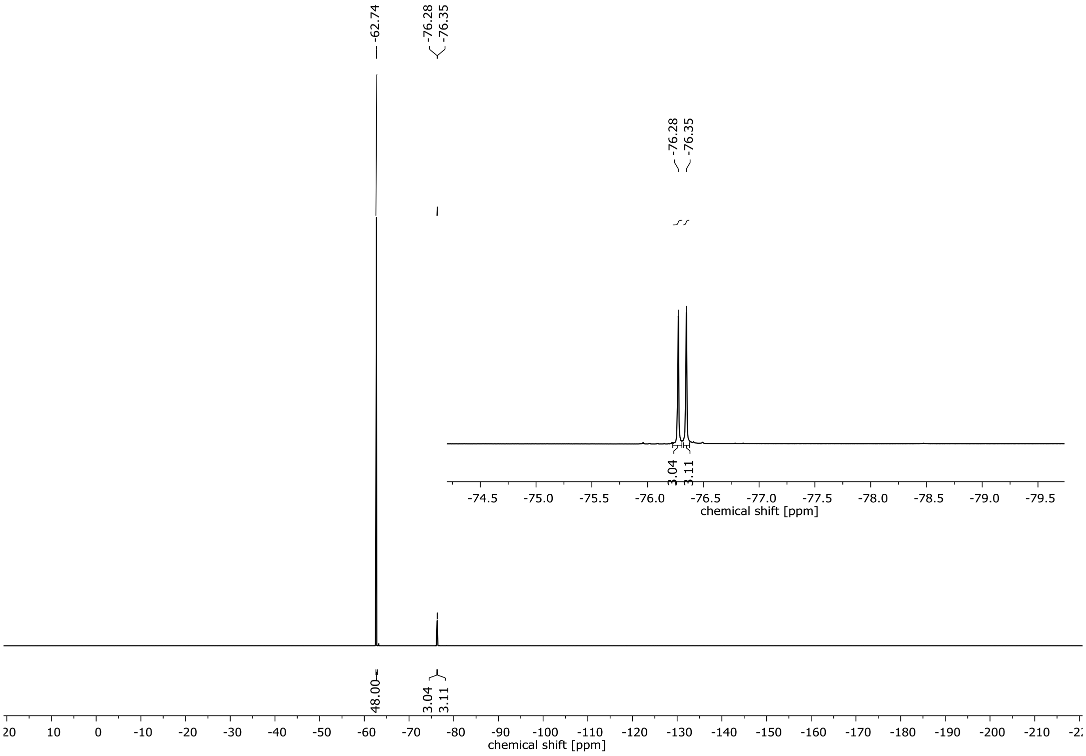

Figure S109. ^{19}F NMR spectrum of **7b$^{\mathbf{BArF}}$** (fraction 2) (376 MHz, DCM-d_2).

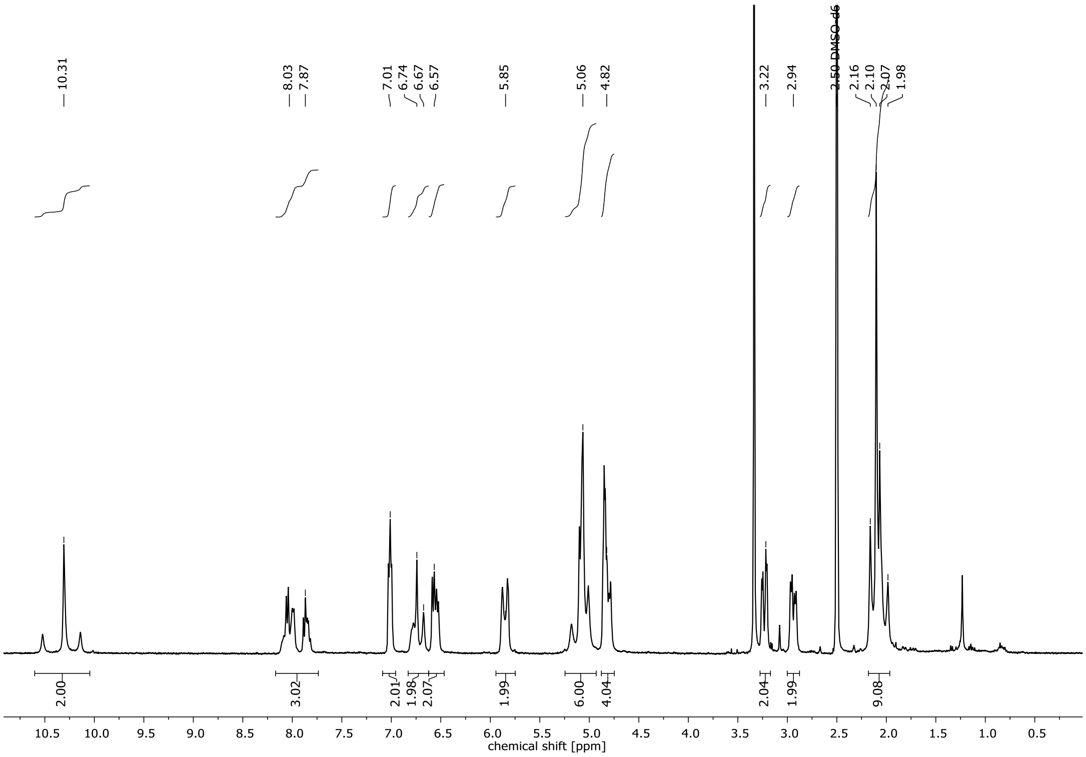

Figure S110. ^{1}H NMR spectrum of **6$^{\mathbf{Cl}}$** (400 MHz, DMSO-d_6).

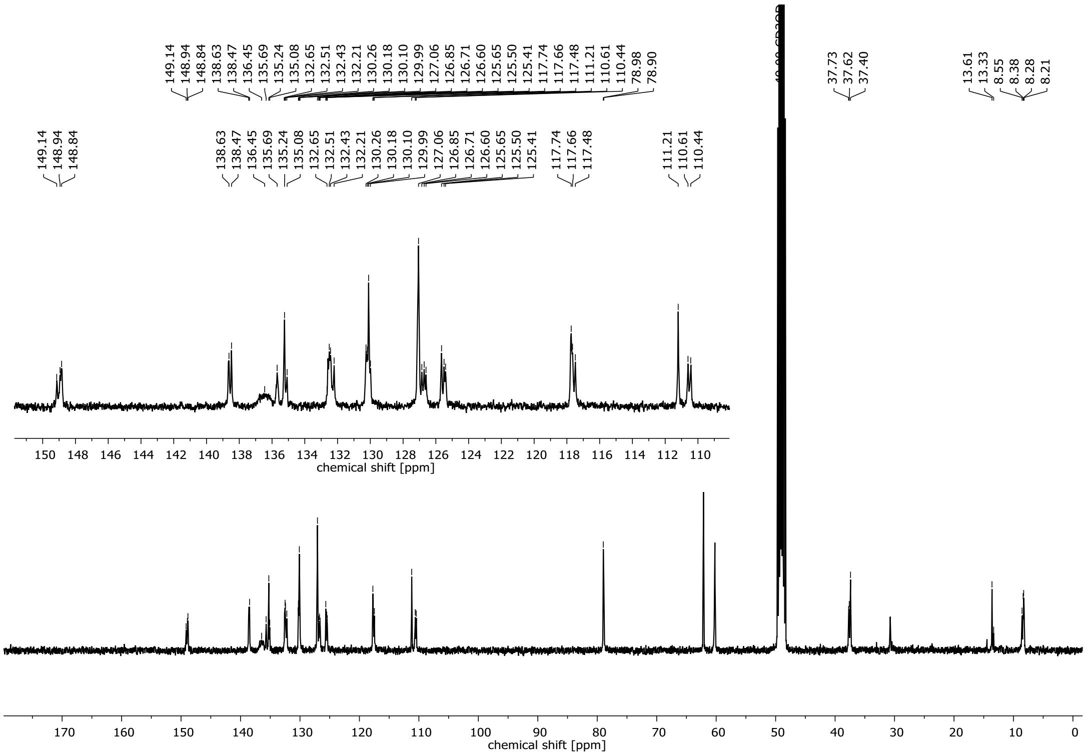

Figure S111. ^{13}C NMR spectrum of **6^{Cl}** (101 MHz, methanol-d_4).

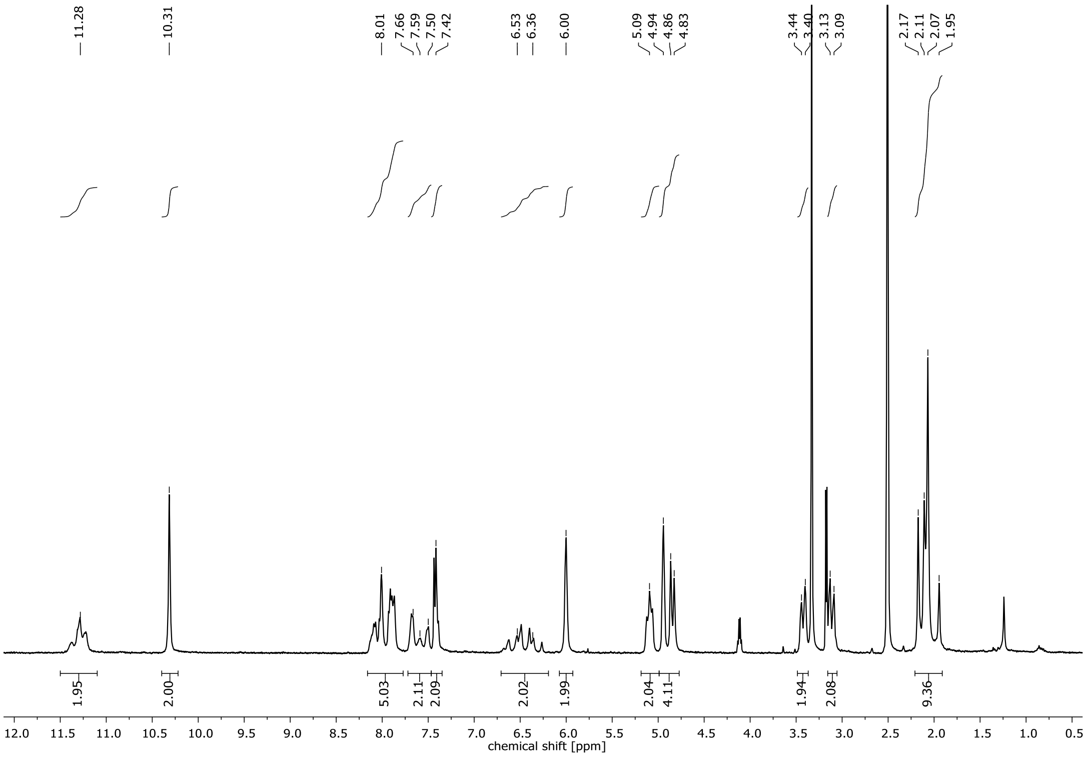

Figure S112. ^{1}H NMR spectrum of **5c^{Cl}**, containing residual MeOH (400 MHz, DMSO-d_6).

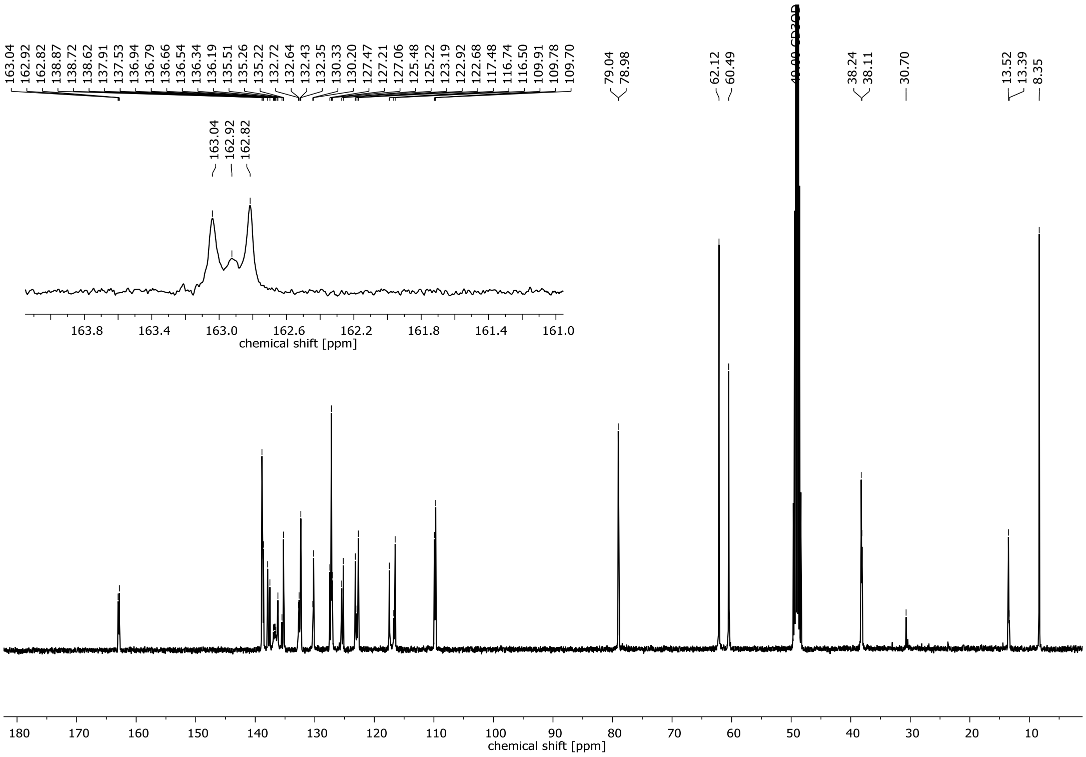

Figure S113. $^{13}C\ \{^{1}H,\ ^{19}F\}$ NMR spectrum of **5c^{Cl}** (101 MHz, methanol-d_4).

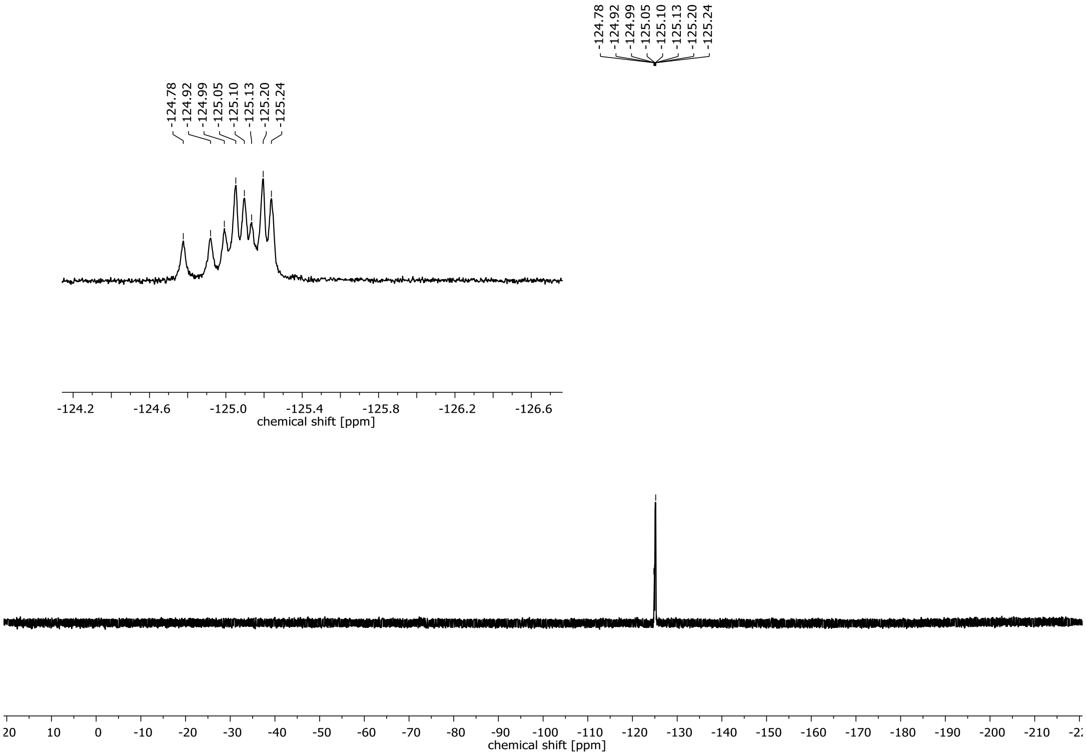

Figure S114. ^{19}F NMR spectrum of **5c^{Cl}** (376 MHz, DMSO-d_6).

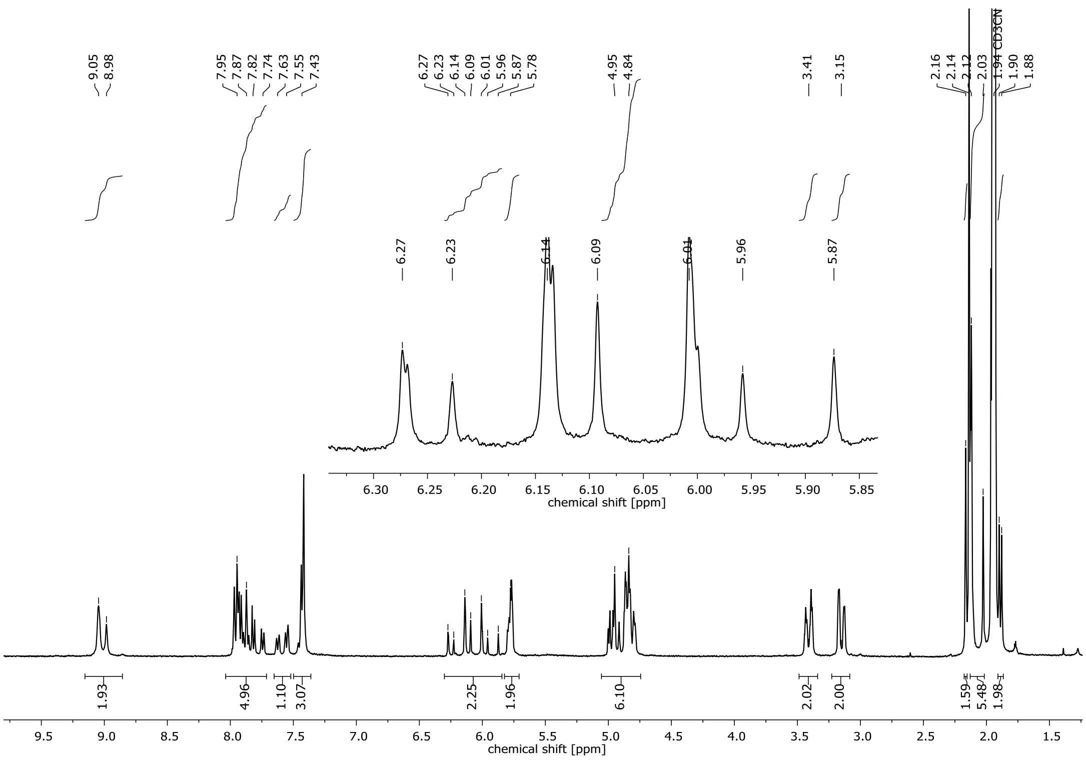

Figure S115. ^{1}H NMR spectrum of **7c^{OTf}** (fraction 1) (400 MHz, acetonitrile-d_3).

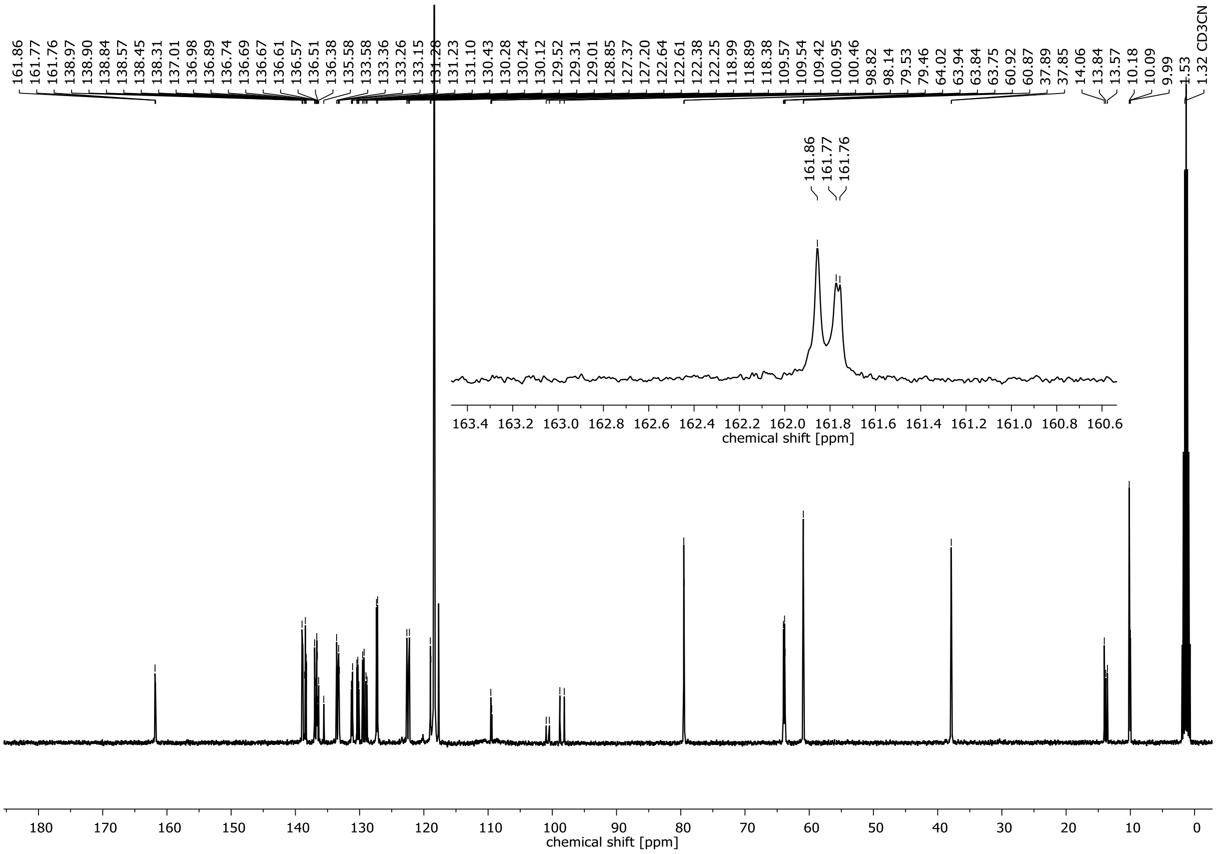

Figure S116. ^{13}C {^{1}H, ^{19}F} NMR spectrum of **7c^{OTf}** (fraction 1) (101 MHz, acetonitrile-d_3).

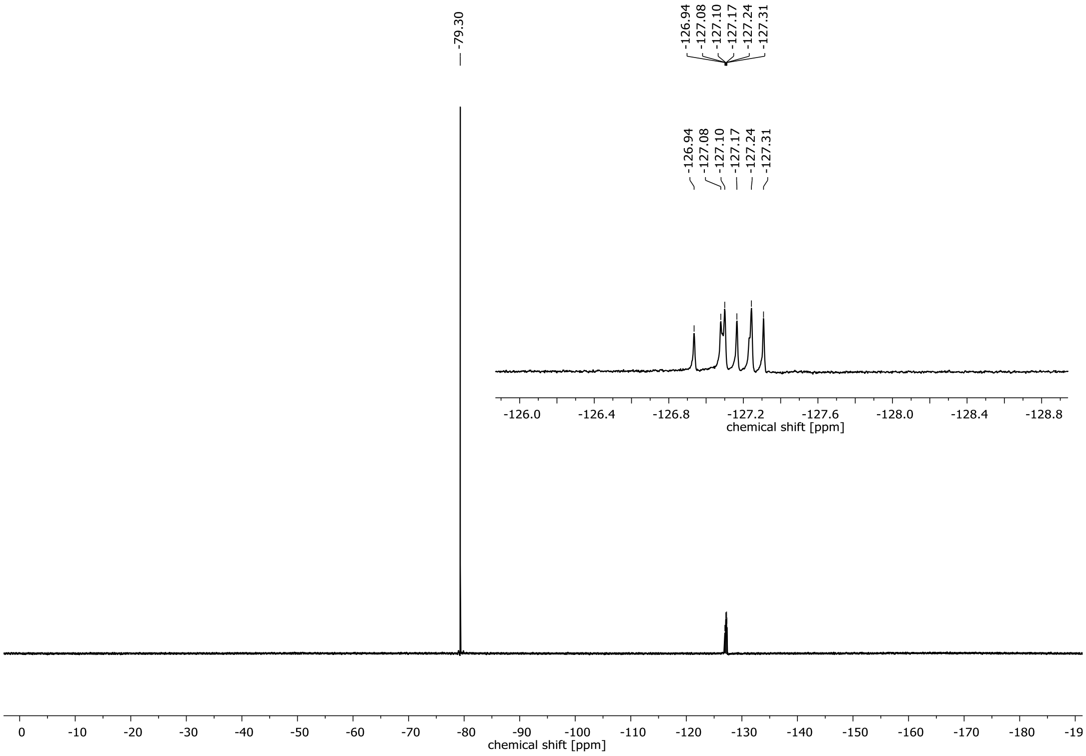

Figure S117. ^{19}F NMR spectrum of **7c^{OTf}** (fraction 1) (376 MHz, acetonitrile-d_3).

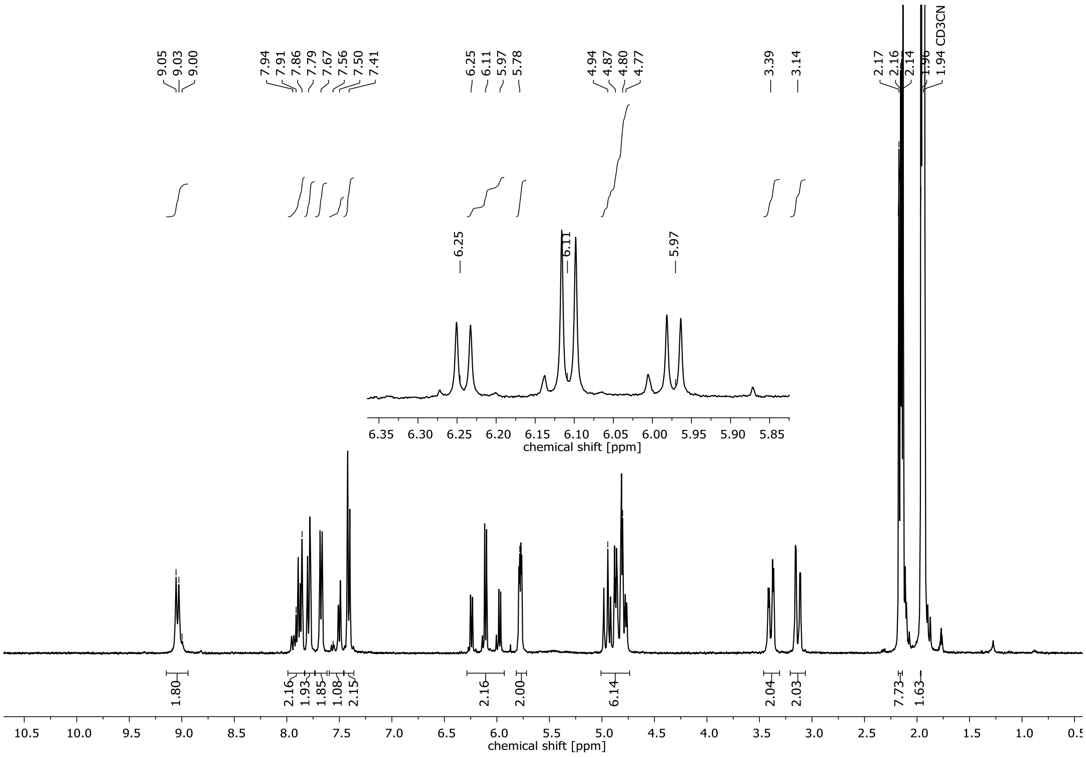

Figure S118. ^{1}H NMR spectrum of **7c^{OTf}** (fraction 2) (400 MHz, acetonitrile-d_3).

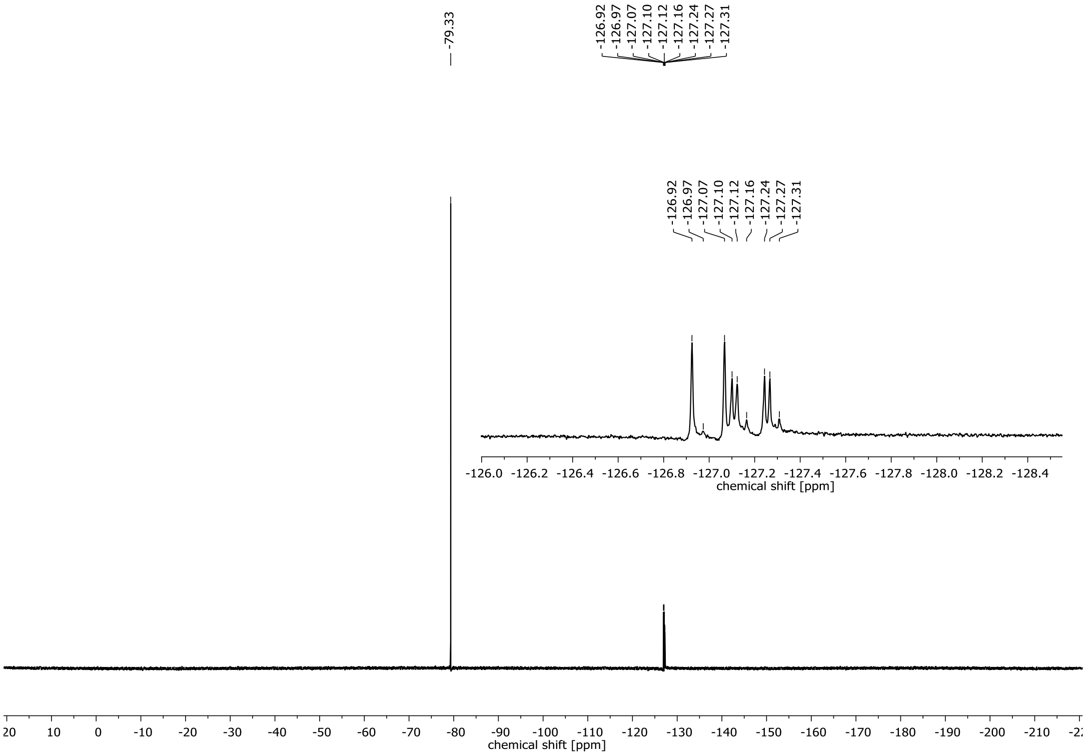

Figure S119. ^{19}F NMR spectrum of **7c^{OTf}** (fraction 2) (376 MHz, acetonitrile-d_3).

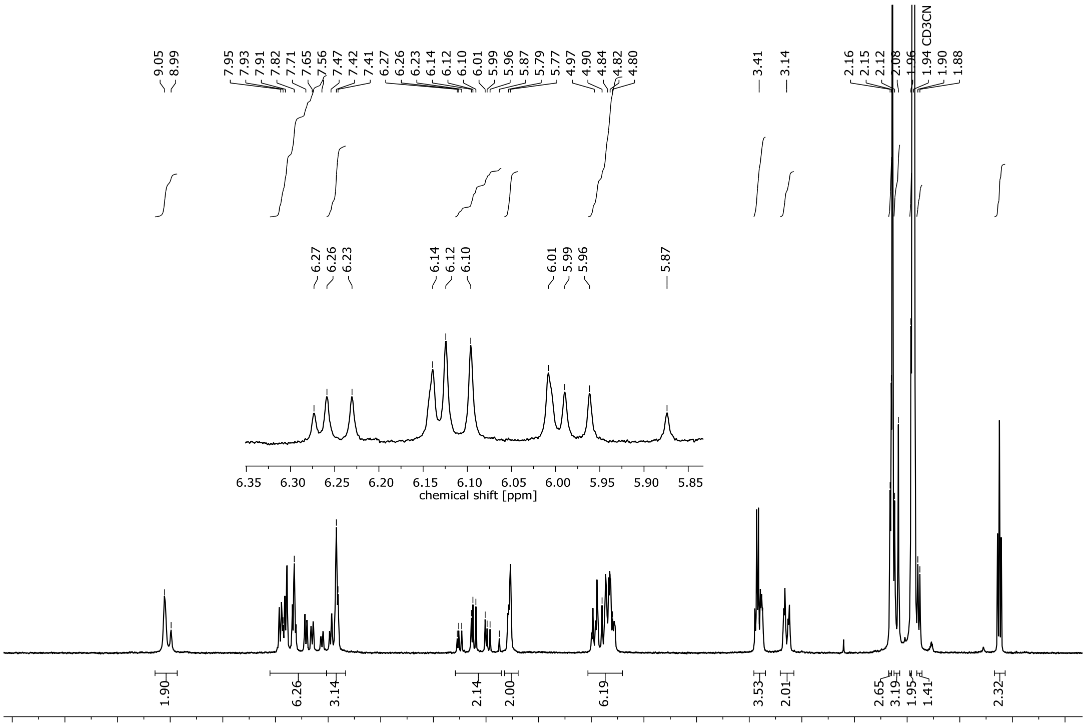

Figure S120. ^{1}H NMR spectrum of **7c^{OTf}** (mixture of atropisomers, before separation) (400 MHz, acetonitrile-d_3). Residual Et_2O is present, leading to a deviation in the signal at 3.4 ppm.

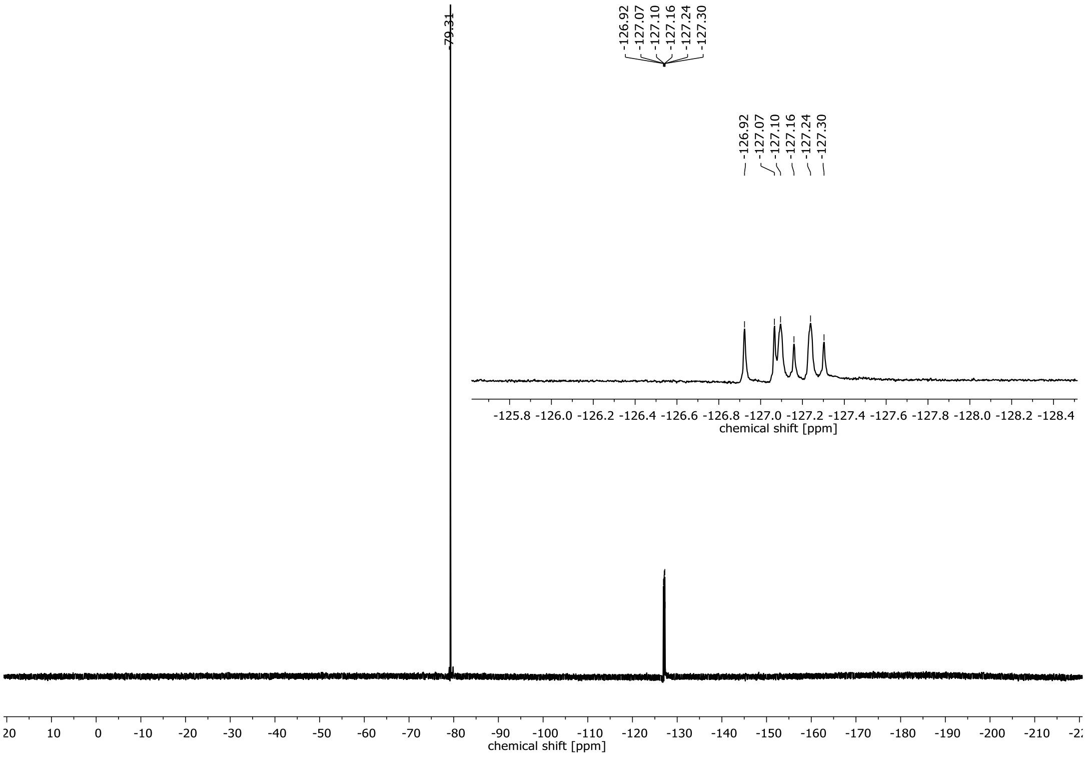

Figure S121. ^{19}F NMR spectrum of **7c^{OTf}** (mixture of atropisomers, before separation) (376 MHz, acetonitrile-d_3).

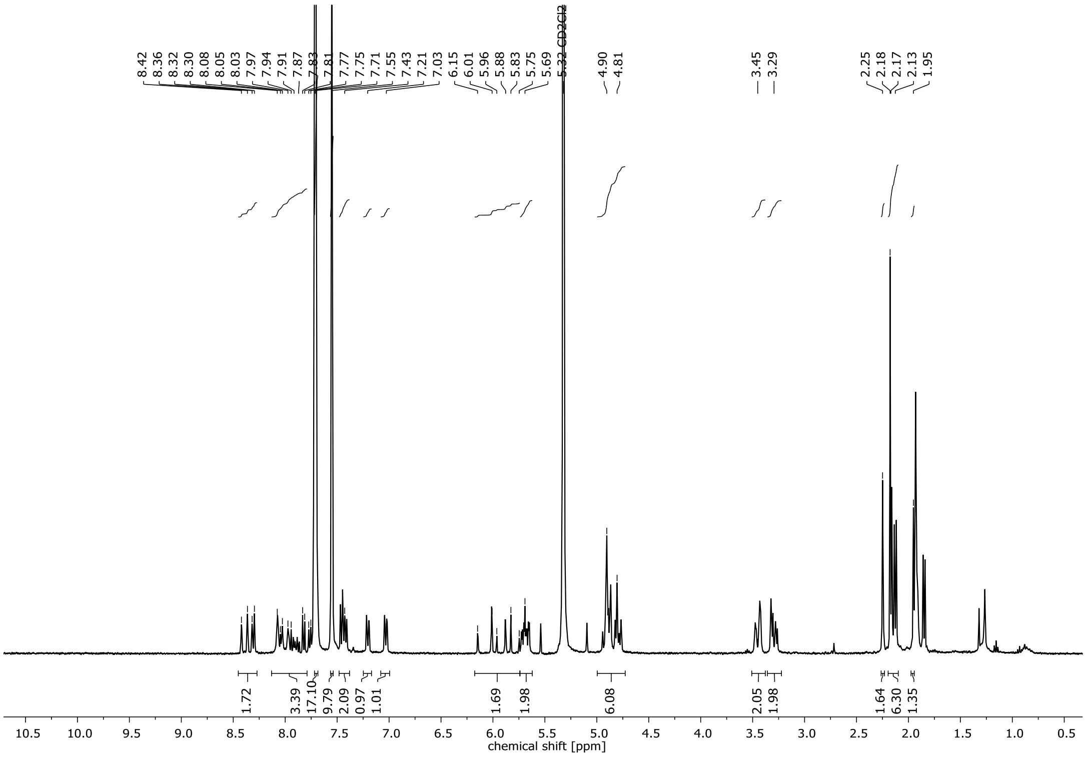

Figure S122. ^{1}H NMR spectrum of **7c^{BArF}** (fraction 1) (400 MHz, DCM-d_2).

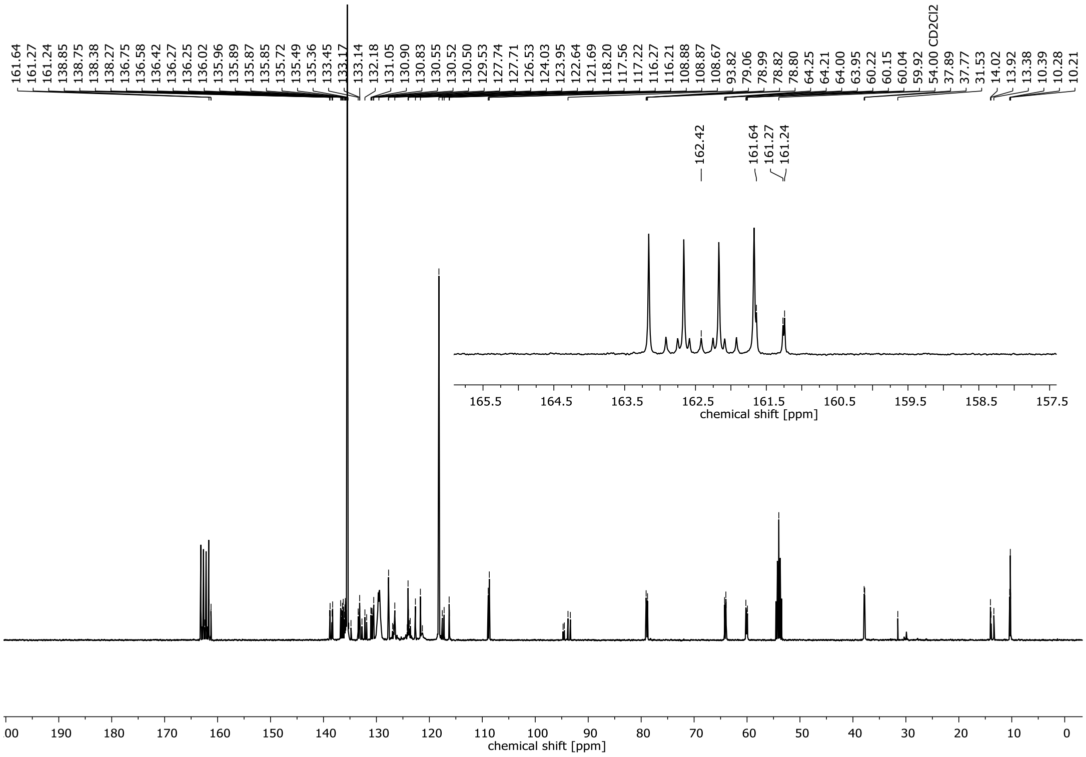

Figure S123. ^{13}C {^{1}H, ^{19}F} NMR spectrum of **7c^{BArF}** (fraction 1) (101 MHz, DCM-*d*$_2$).

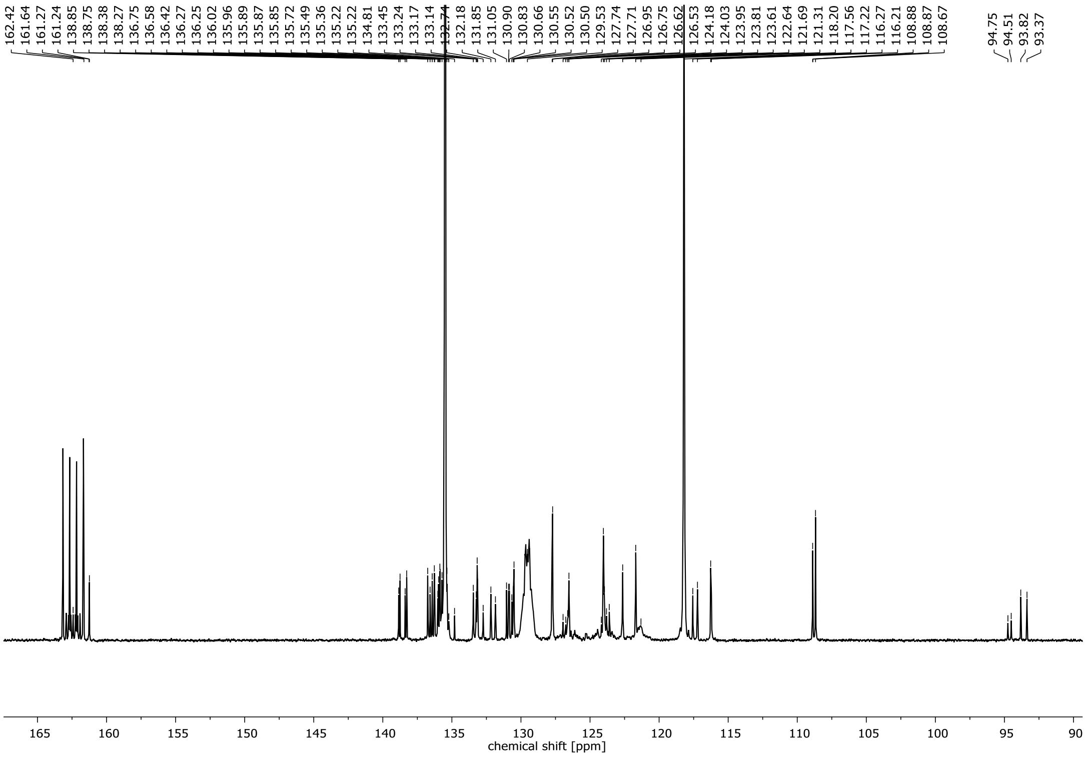

Figure S124. ^{13}C {^{1}H, ^{19}F} NMR spectrum of **7c^{BArF}** (fraction 1), zoom into aromatic region (101 MHz, DCM-*d*$_2$).

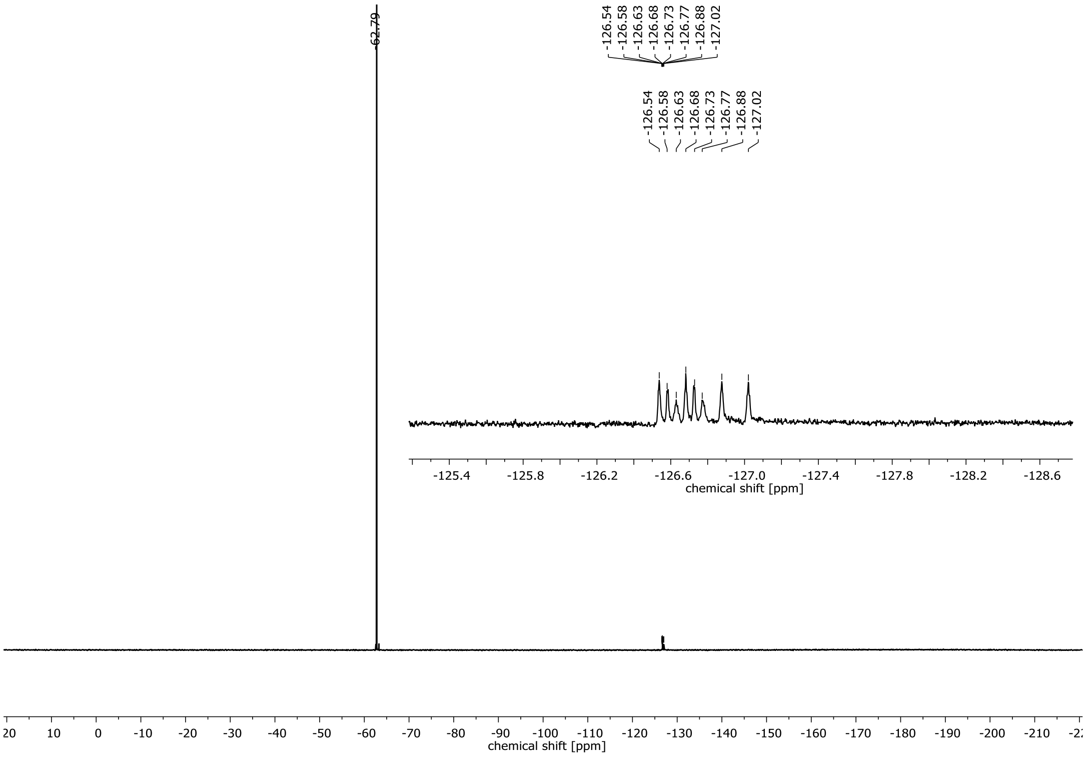

Figure S125. ^{19}F NMR spectrum of **7c**$^{\mathbf{BArF}}$ (fraction 1) (376 MHz, DCM-d_2).

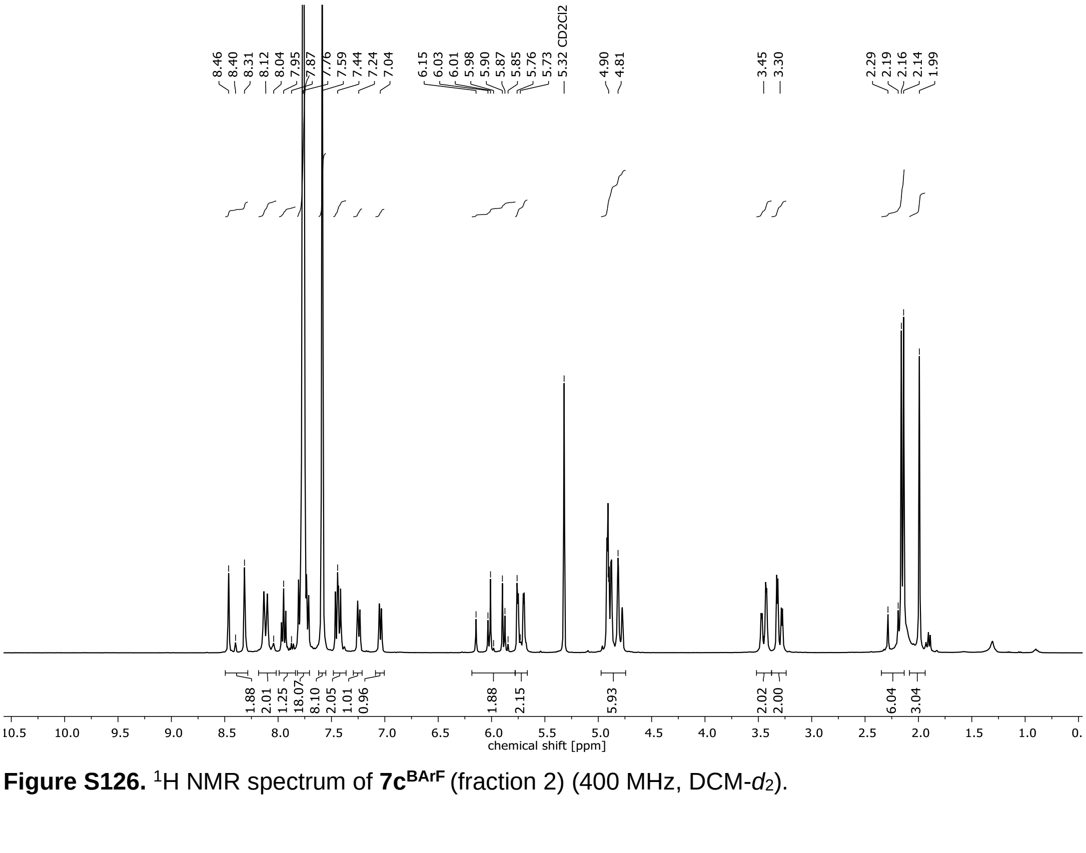

Figure S126. ^{1}H NMR spectrum of **7c**$^{\mathbf{BArF}}$ (fraction 2) (400 MHz, DCM-d_2).

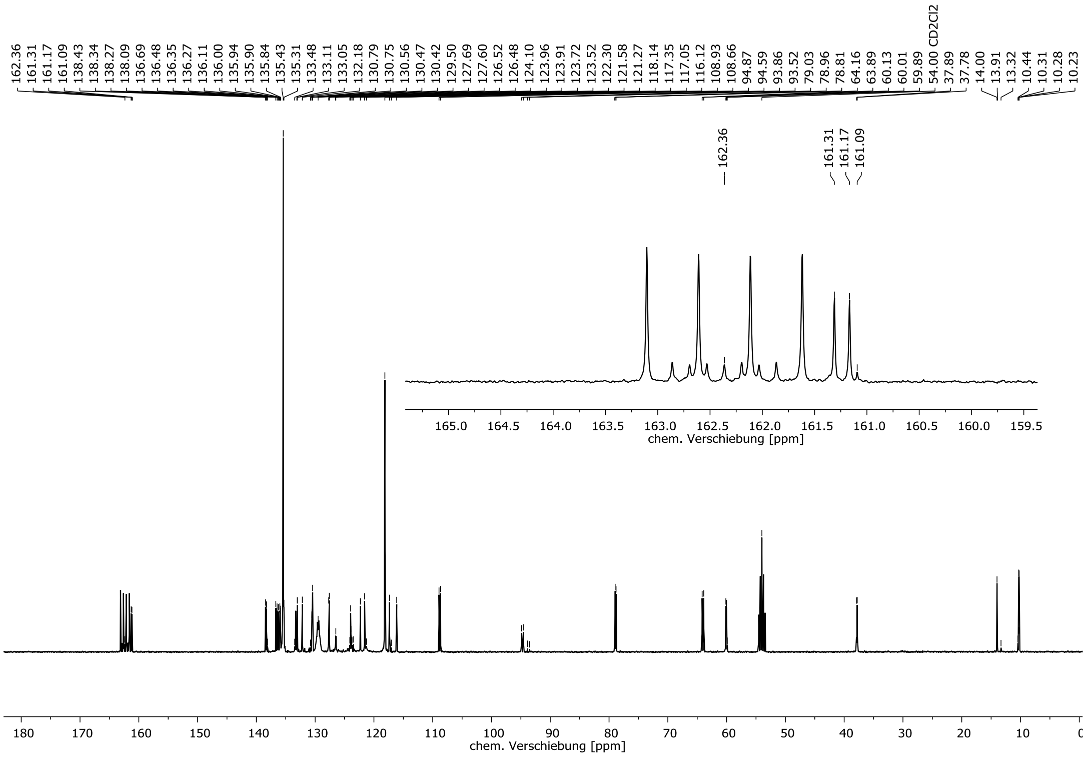

Figure S127. ^{13}C {^1H, ^{19}F} NMR spectrum of **7c^{BArF}** (fraction 2) (101 MHz, DCM-d_2).

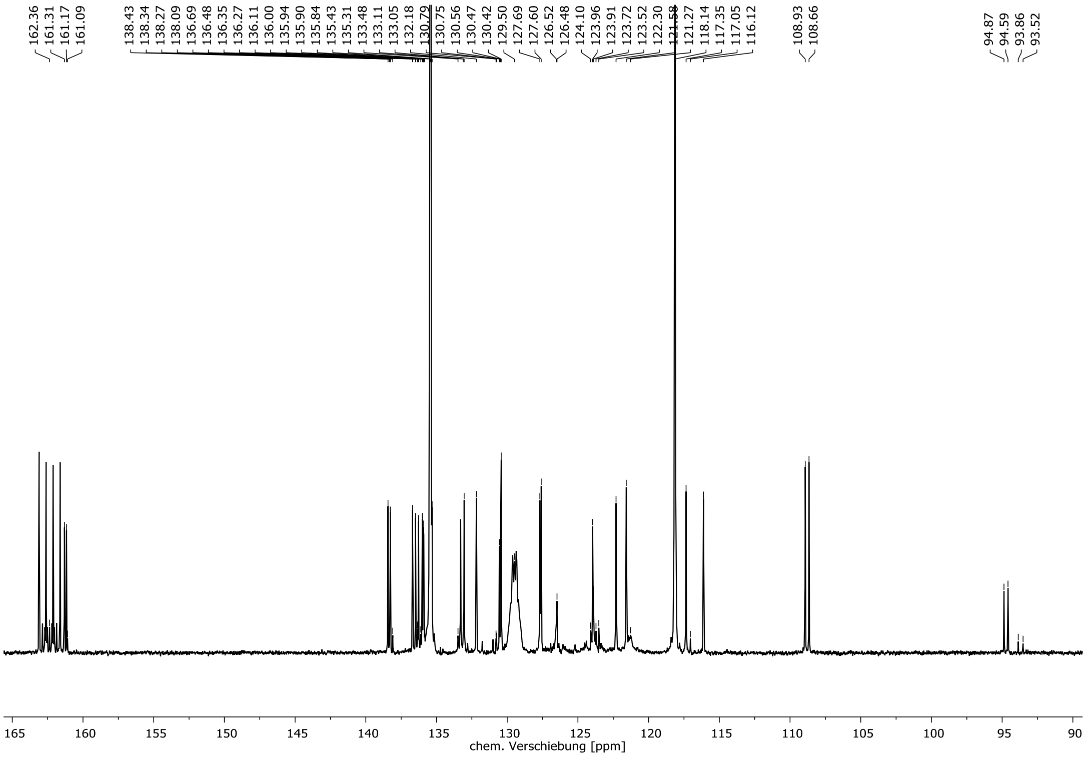

Figure S128. ^{13}C {^1H, ^{19}F} NMR spectrum of **7c^{BArF}** (fraction 2), zoom into aromatic region (101 MHz, DCM-d_2).

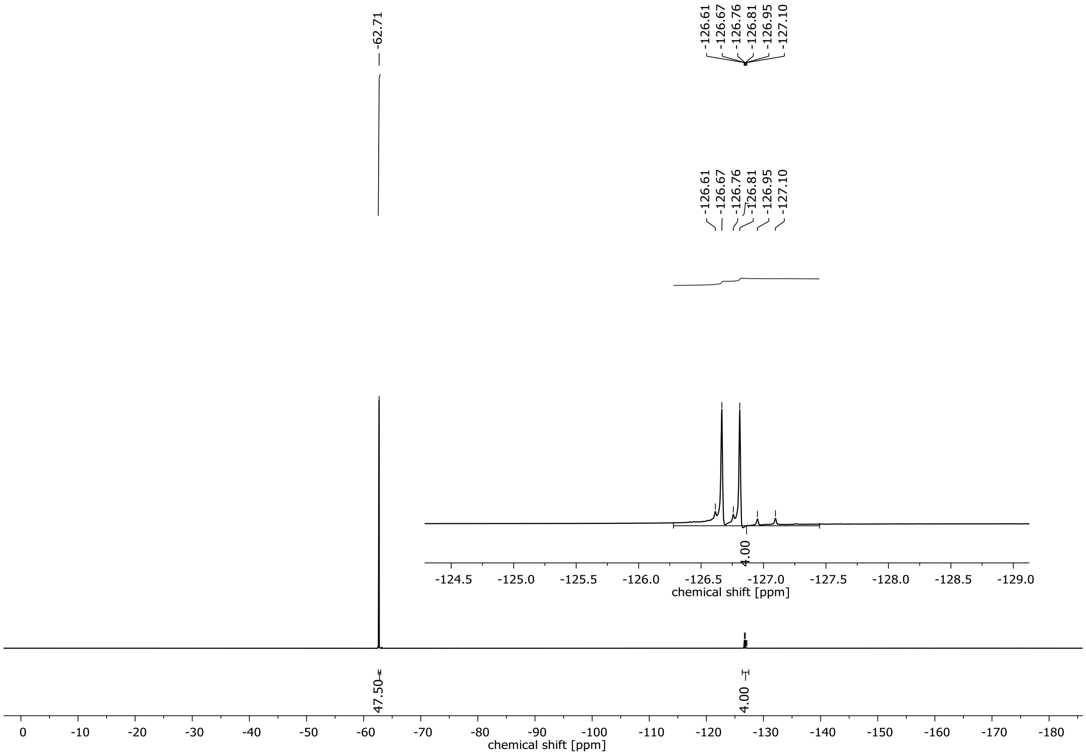

Figure S129. ^{19}F NMR spectrum of **7c^{BArF}** (fraction 2) (376 MHz, DCM-d_2).

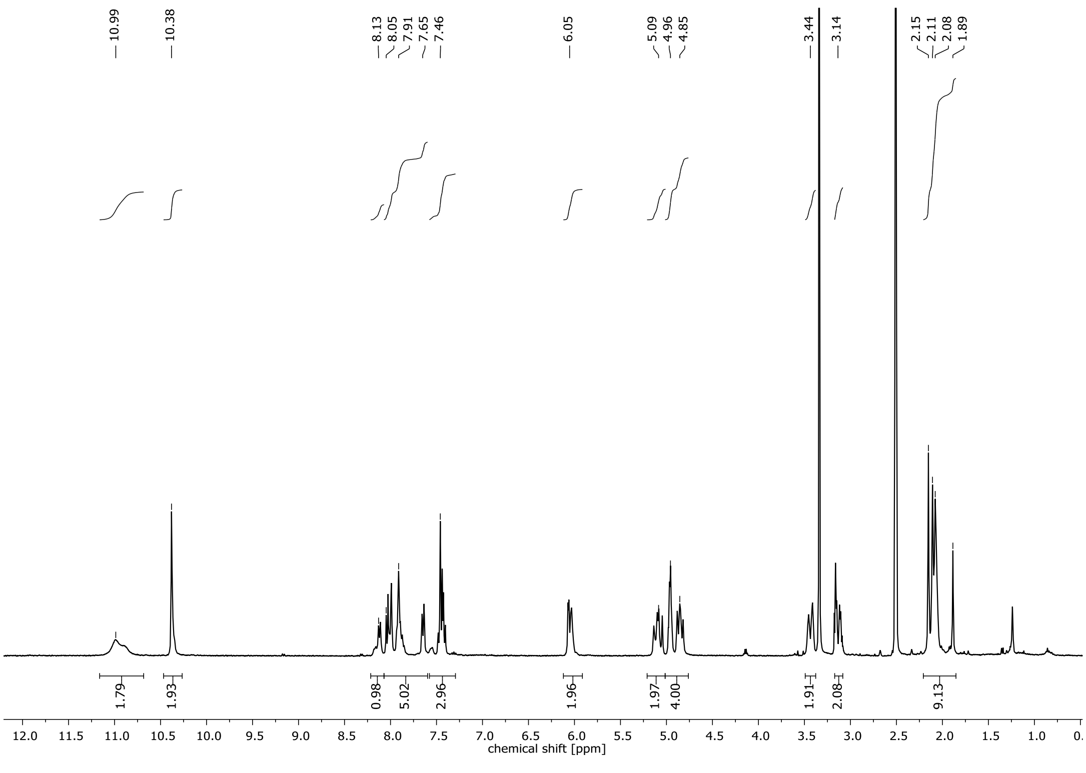

Figure S130. ^{1}H NMR spectrum of **5d^{Cl}** (400 MHz, DMSO-d_6).

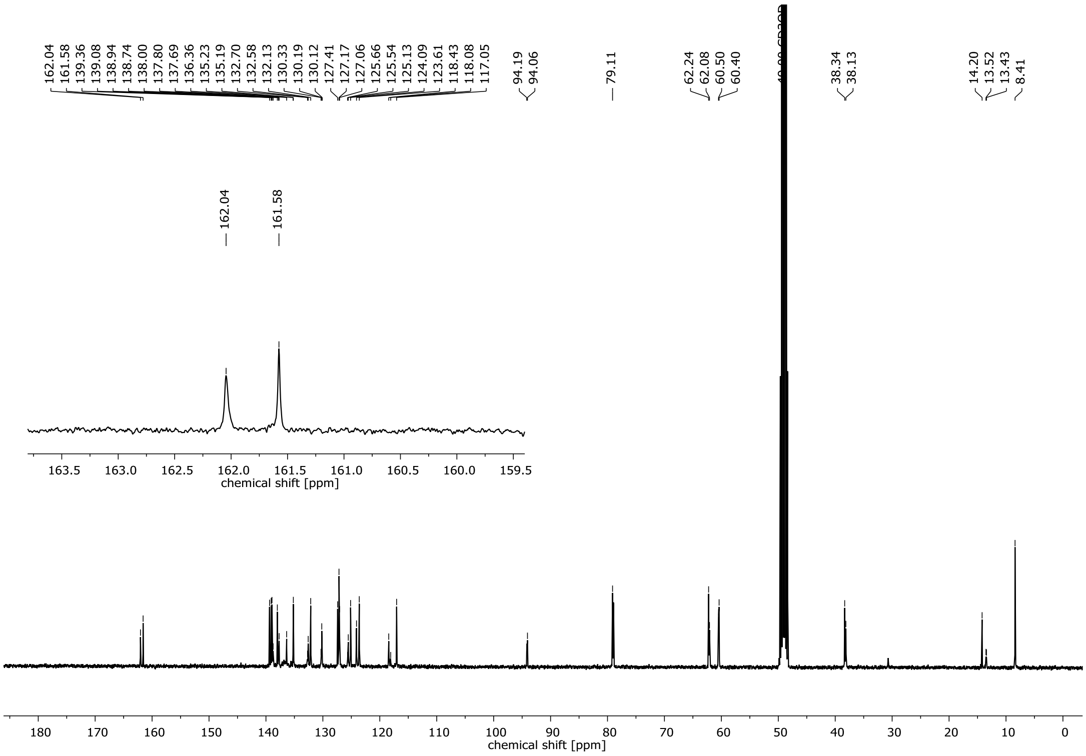

Figure S131. ^{13}C NMR spectrum of **5d^{Cl}** (101 MHz, methanol-d_4).

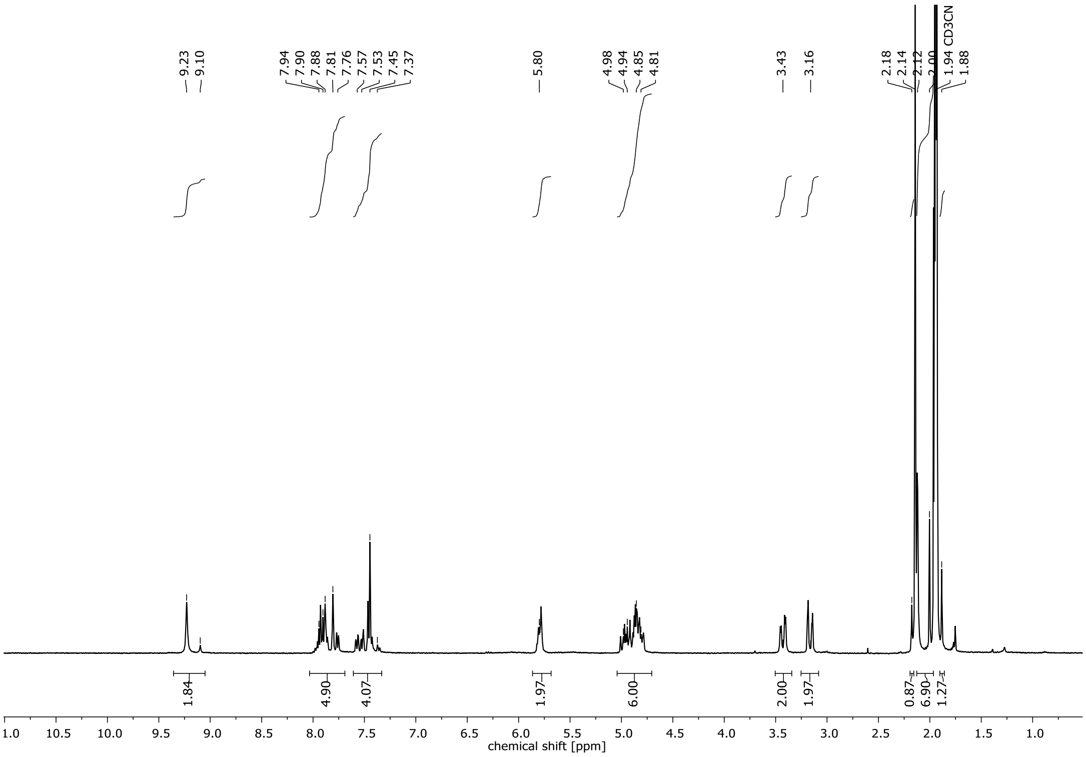

Figure S132. ^{1}H NMR spectrum of **7d^{OTf}** (fraction 1) (400 MHz, acetonitrile-d_3).

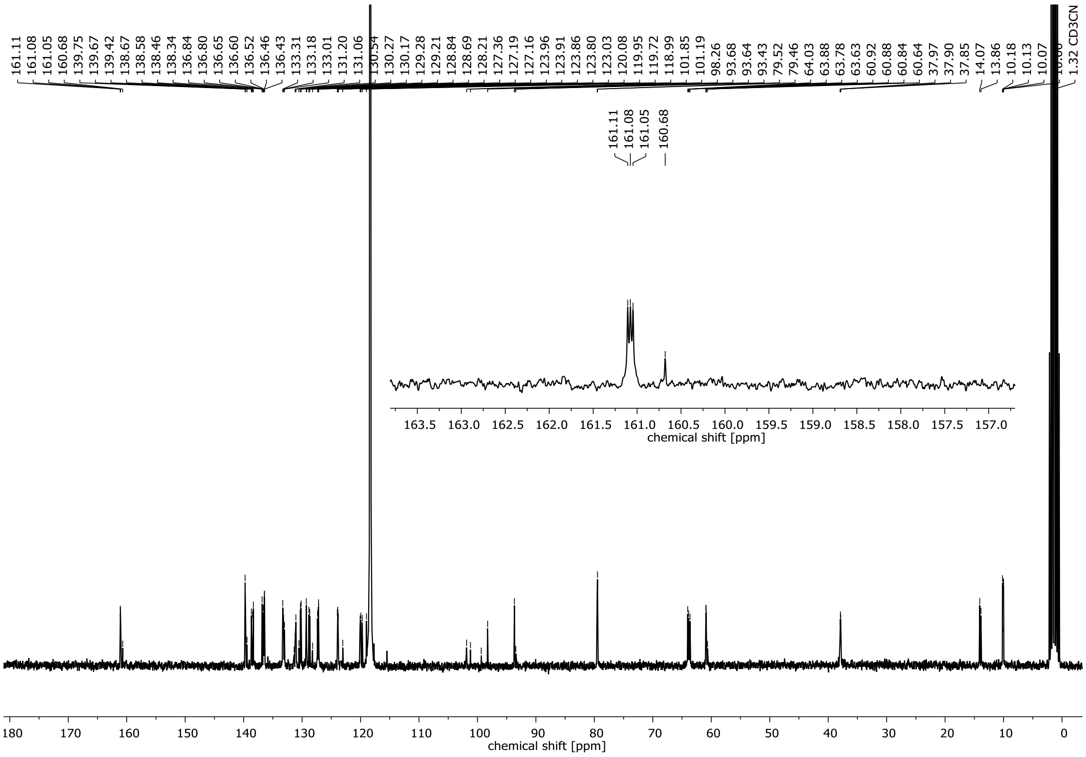

Figure S133. ^{13}C NMR spectrum of **7d$^{\mathbf{OTf}}$** (fraction 1) (75 MHz, acetonitrile-*d*$_3$).

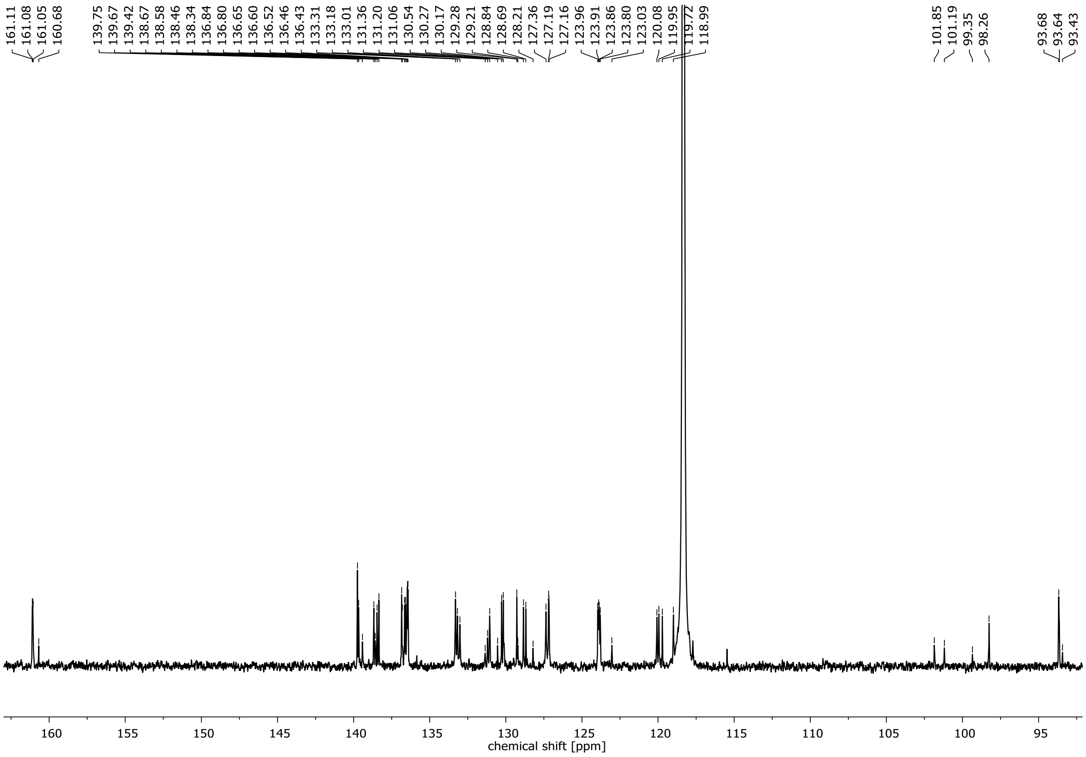

Figure S134. ^{13}C NMR spectrum of **7d$^{\mathbf{OTf}}$** (fraction 1), zoom into aromatic region (75 MHz, acetonitrile-*d*$_3$).

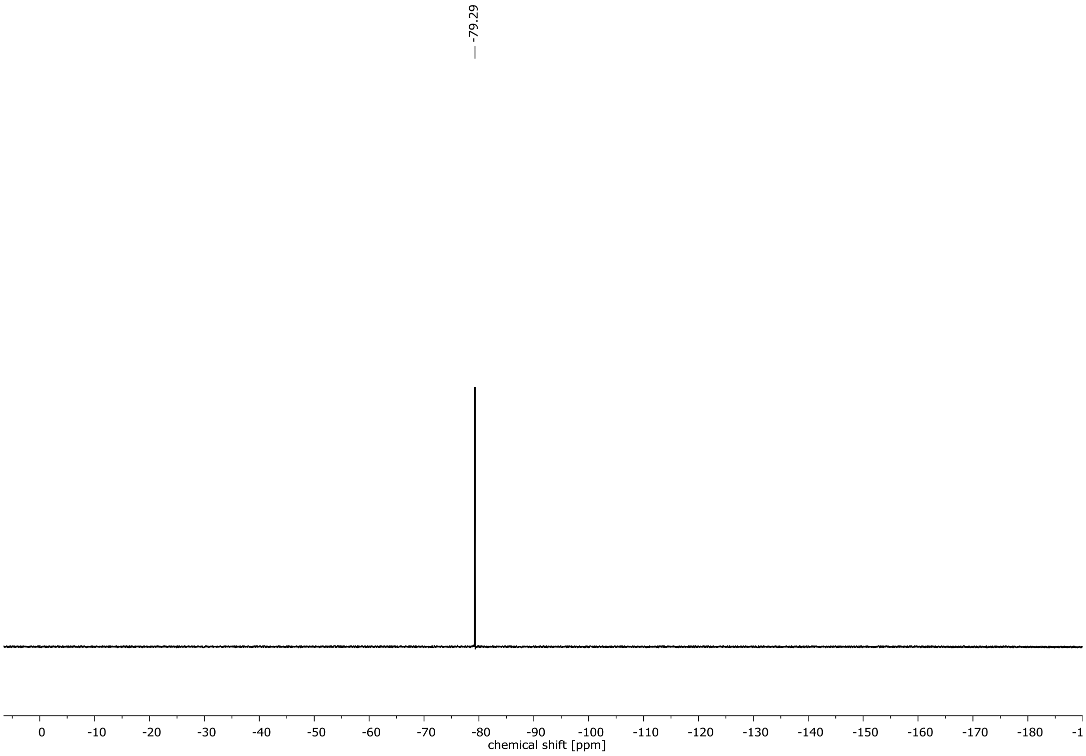

Figure S135. ^{19}F NMR spectrum of **7d$^{\mathbf{OTf}}$** (fraction 1) (376 MHz, acetonitrile-*d*$_3$).

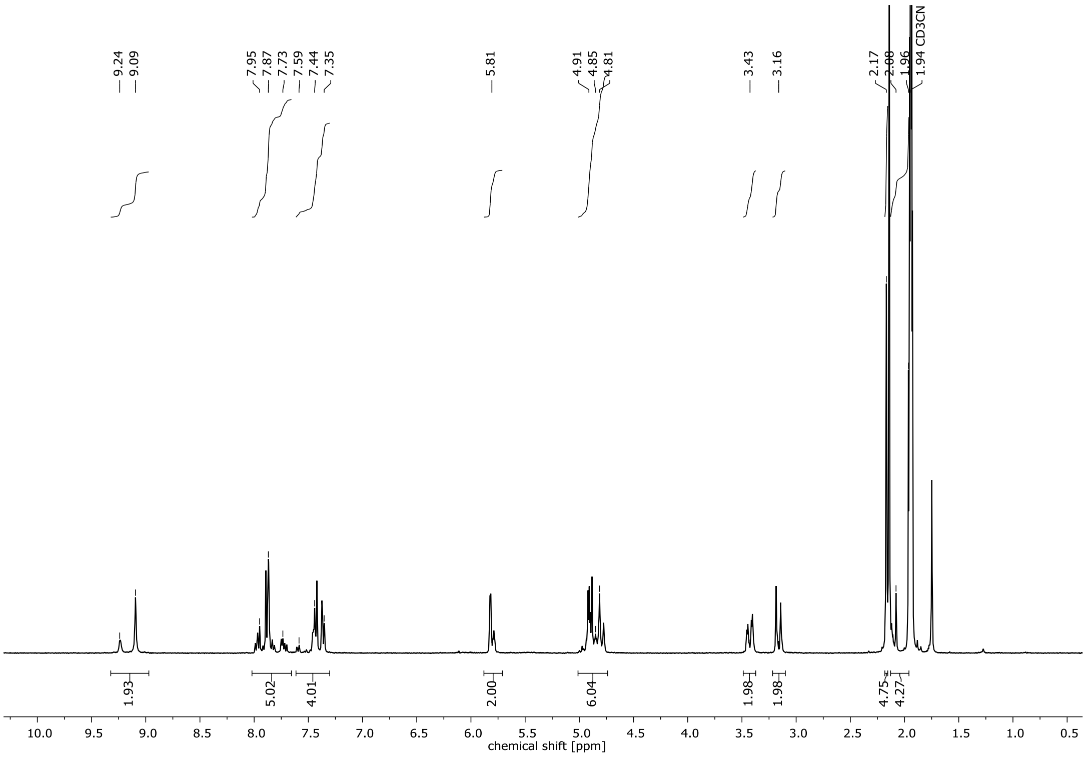

Figure S136. ^{1}H NMR spectrum of **7d$^{\mathbf{OTf}}$** (fraction 2) (400 MHz, acetonitrile-*d*$_3$).

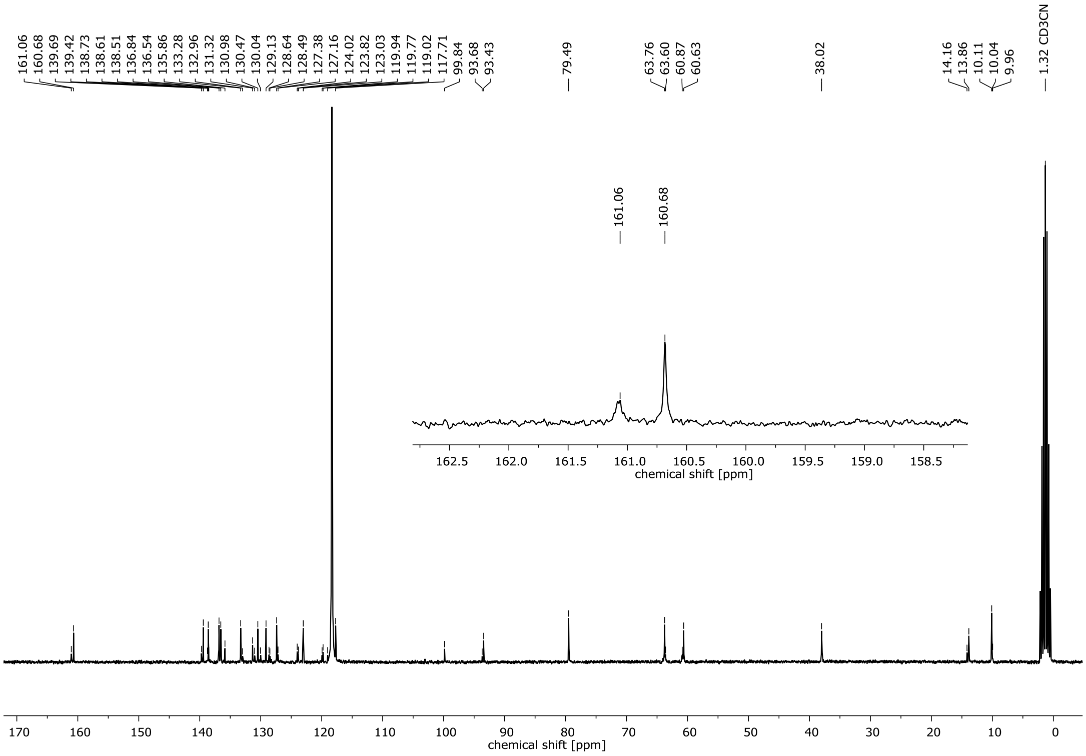

Figure S137. ^{13}C NMR spectrum of **7d$^{\mathbf{OTf}}$** (fraction 2) (75 MHz, acetonitrile-d_3).

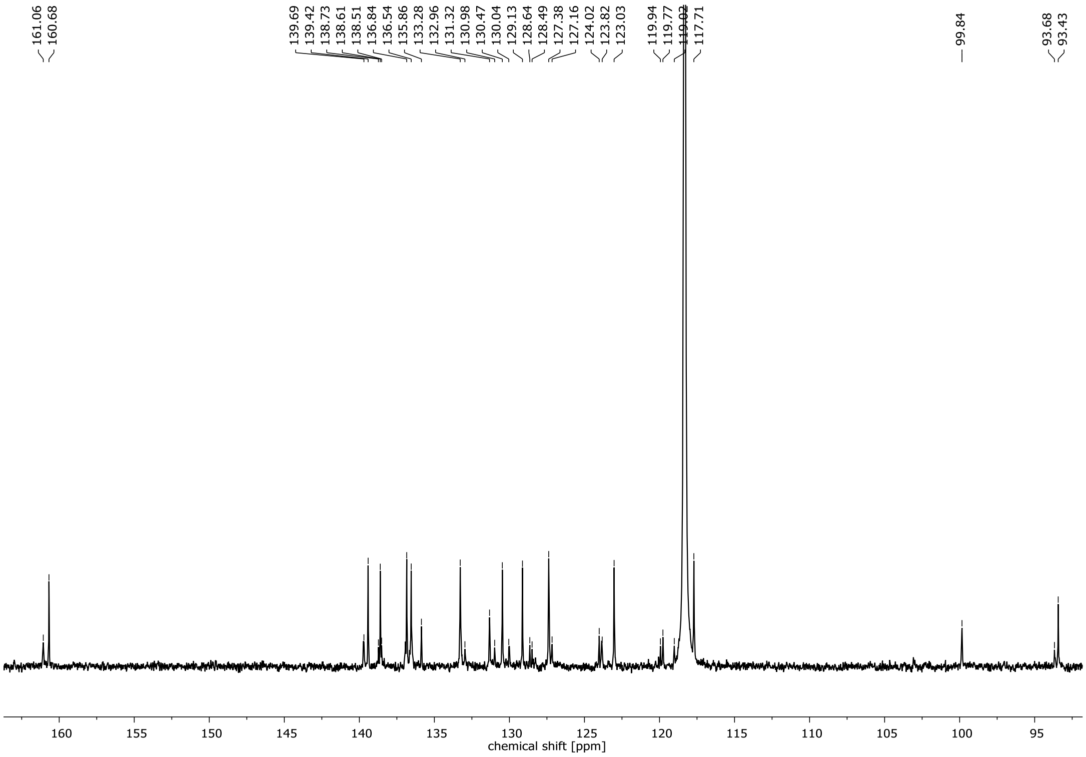

Figure S138. ^{13}C NMR spectrum of **7d$^{\mathbf{OTf}}$** (fraction 2), zoom into aromatic region (75 MHz, acetonitrile-d_3).

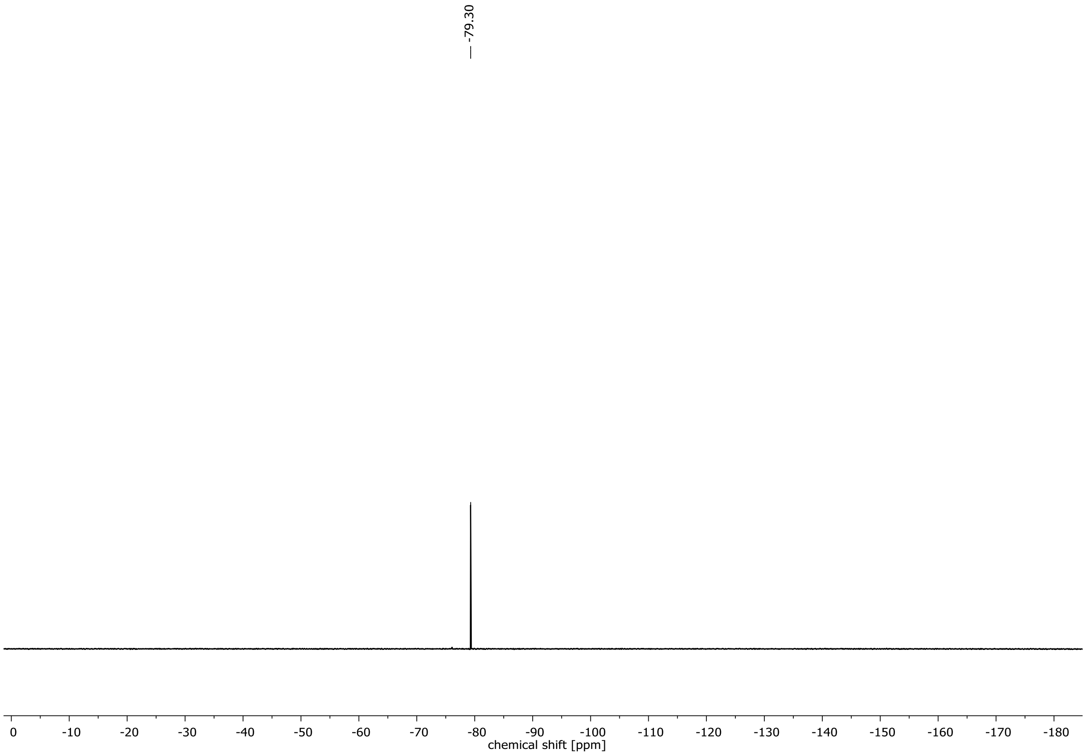

Figure S139. ^{19}F NMR spectrum of **7d^{OTf}** (fraction 2) (376 MHz, acetonitrile-d_3).

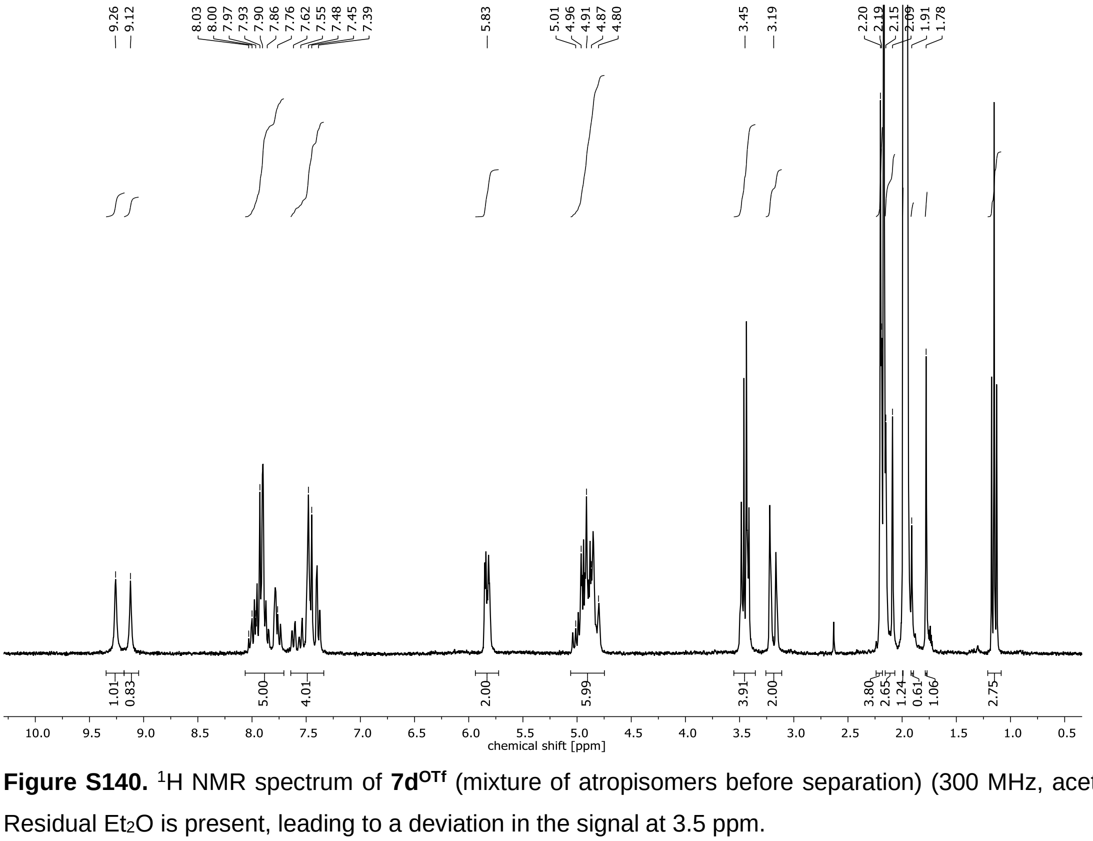

Figure S140. ^{1}H NMR spectrum of **7d^{OTf}** (mixture of atropisomers before separation) (300 MHz, acetonitrile-d_3). Residual Et_2O is present, leading to a deviation in the signal at 3.5 ppm.

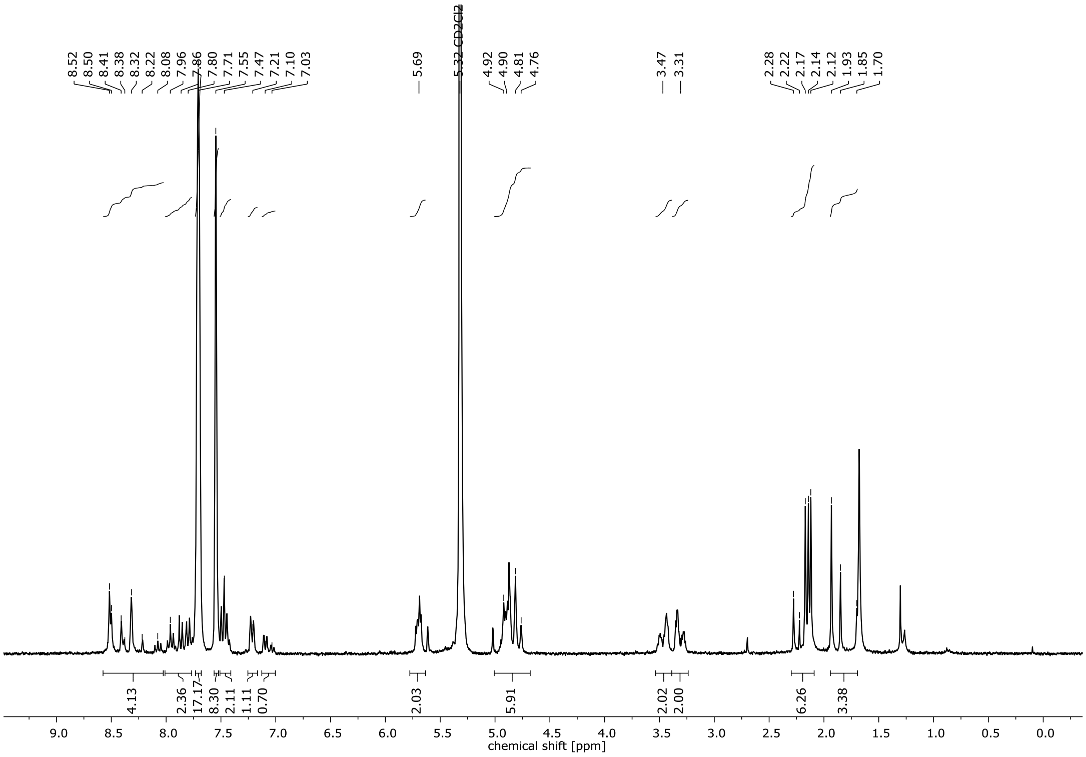

Figure S141. ^{1}H NMR spectrum of **7d$^{\mathbf{BArF}}$** (fraction 1) (300 MHz, DCM-d_2).

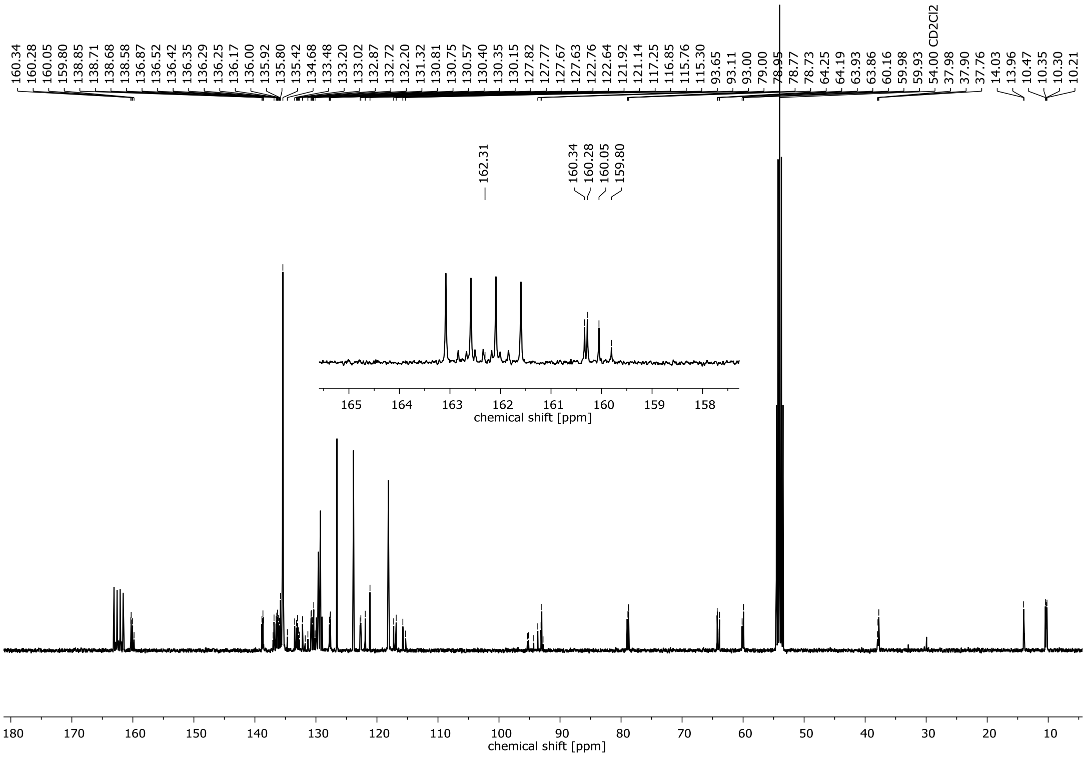

Figure S142. ^{13}C NMR spectrum of **7d$^{\mathbf{BArF}}$** (fraction 1) (101 MHz, DCM-d_2).

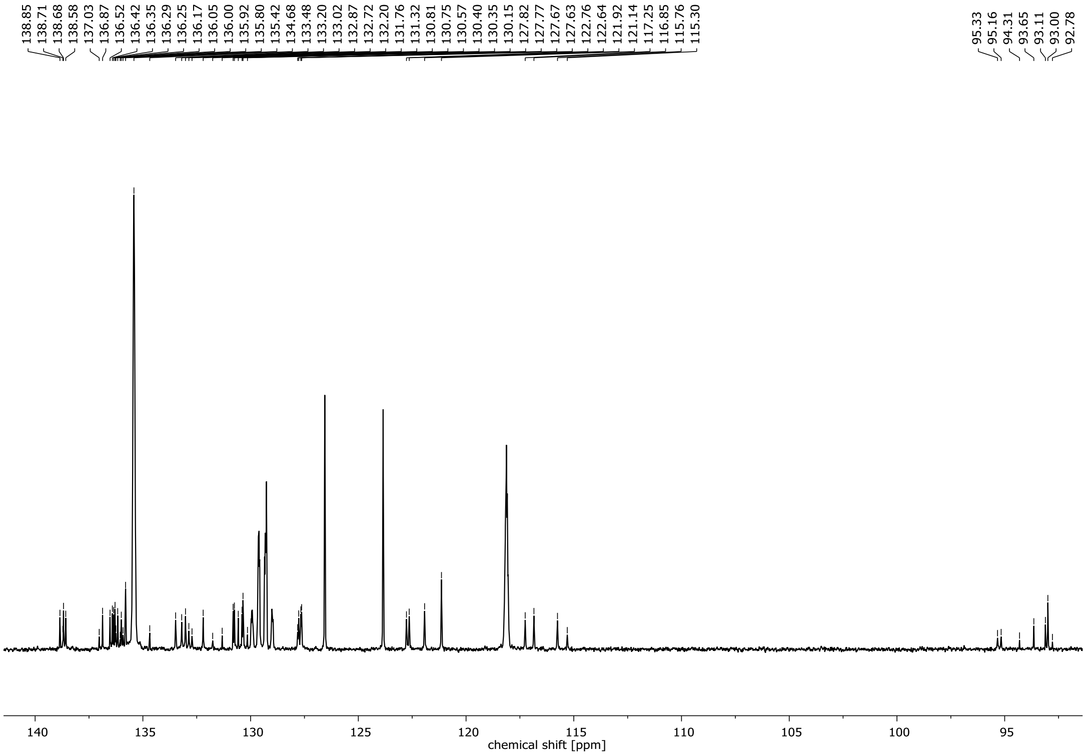

Figure S143. ^{13}C NMR spectrum of **7d$^{\mathbf{BArF}}$** (fraction 1), zoom into aromatic region (101 MHz, DCM-d_2).

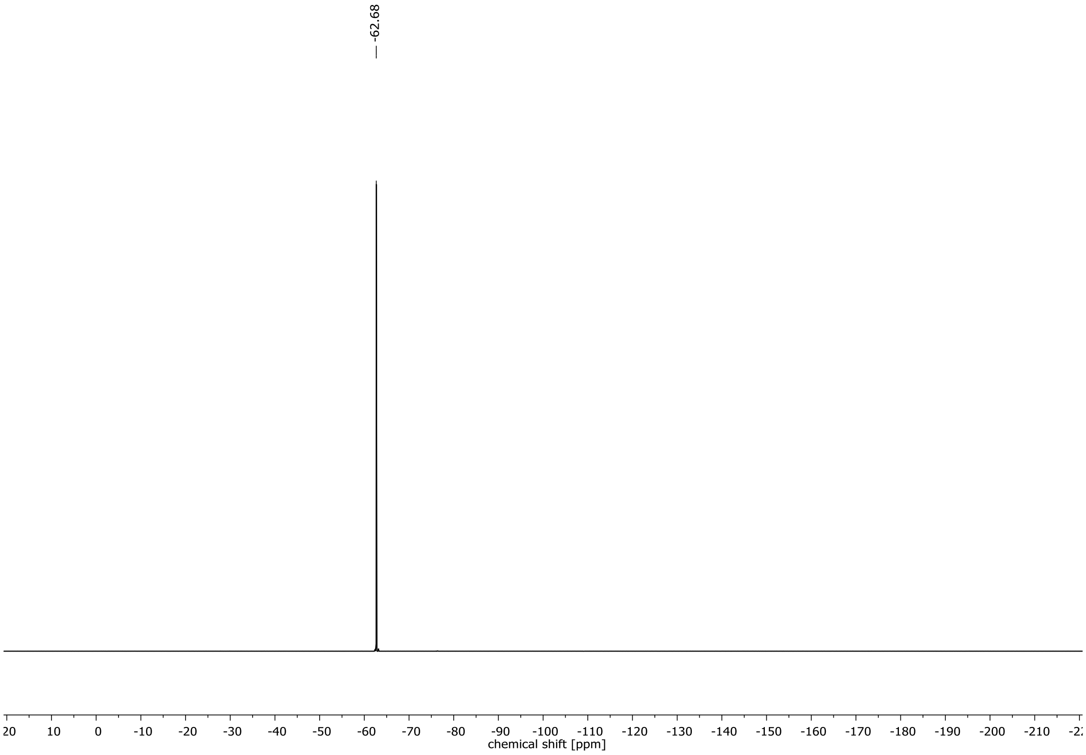

Figure S144. ^{19}F NMR spectrum of **7d$^{\mathbf{BArF}}$** (fraction 1) (376 MHz, DCM-d_2).

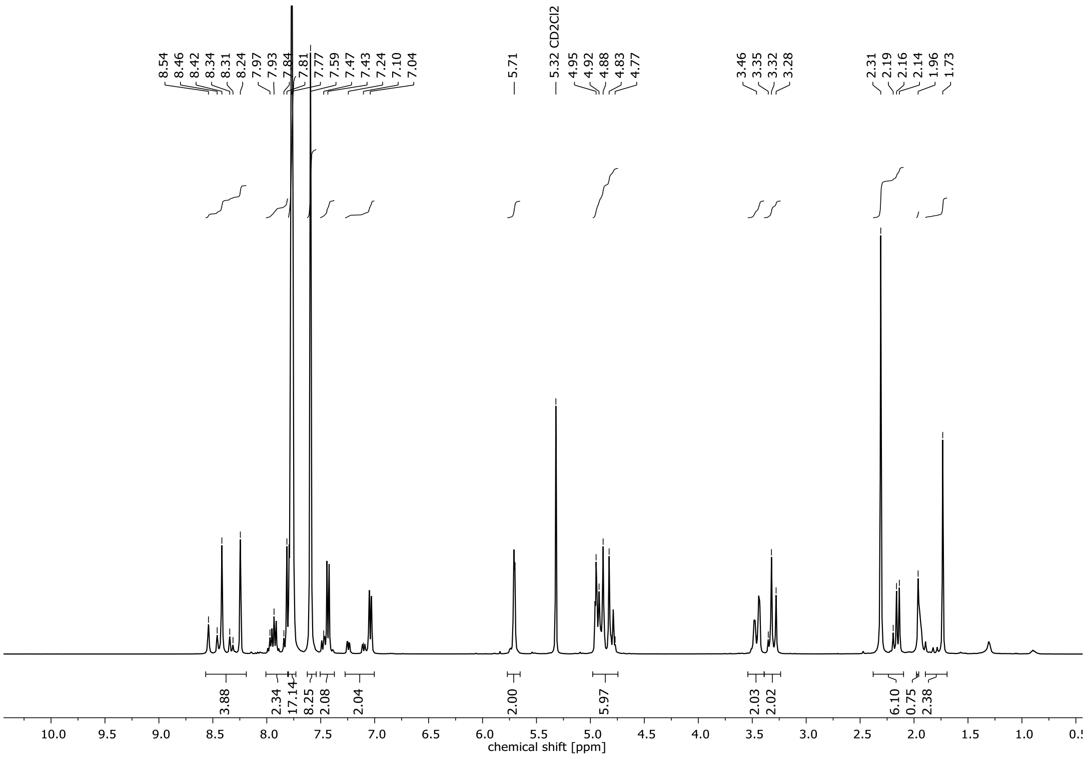

Figure S145. ^{1}H NMR spectrum of **7d^{BArF}** (fraction 2) (400 MHz, DCM-d_2).

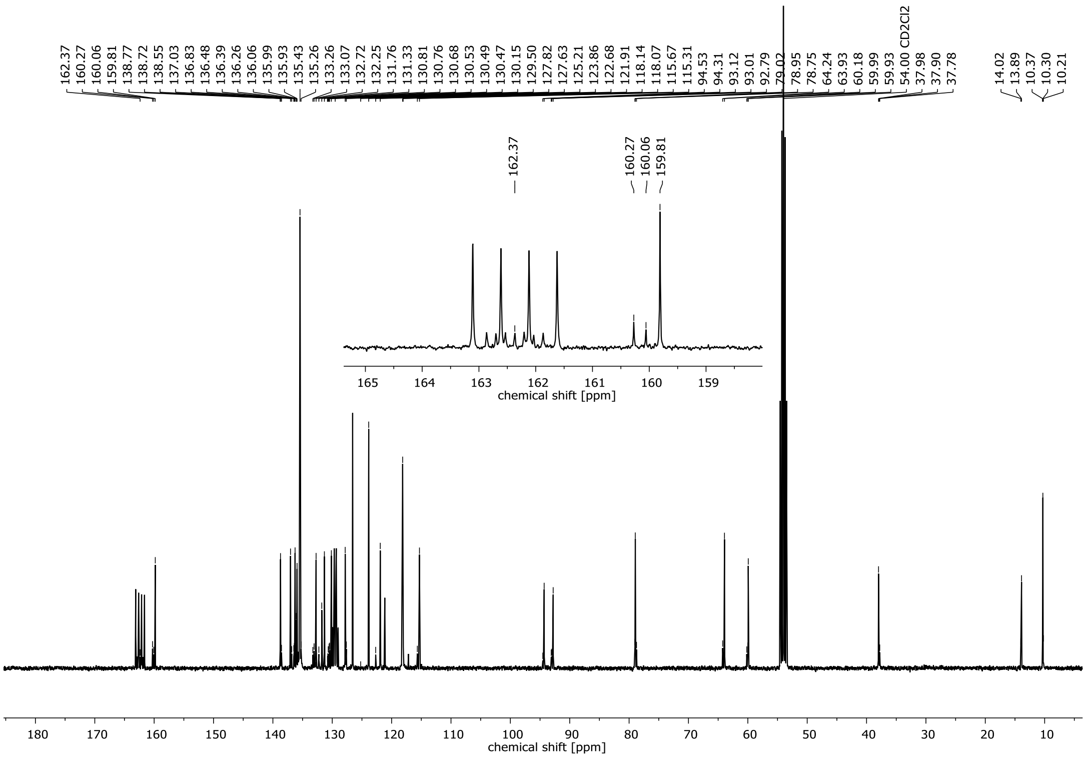

Figure S146. ^{13}C NMR spectrum of **7d^{BArF}** (fraction 2) (101 MHz, DCM-d_2).

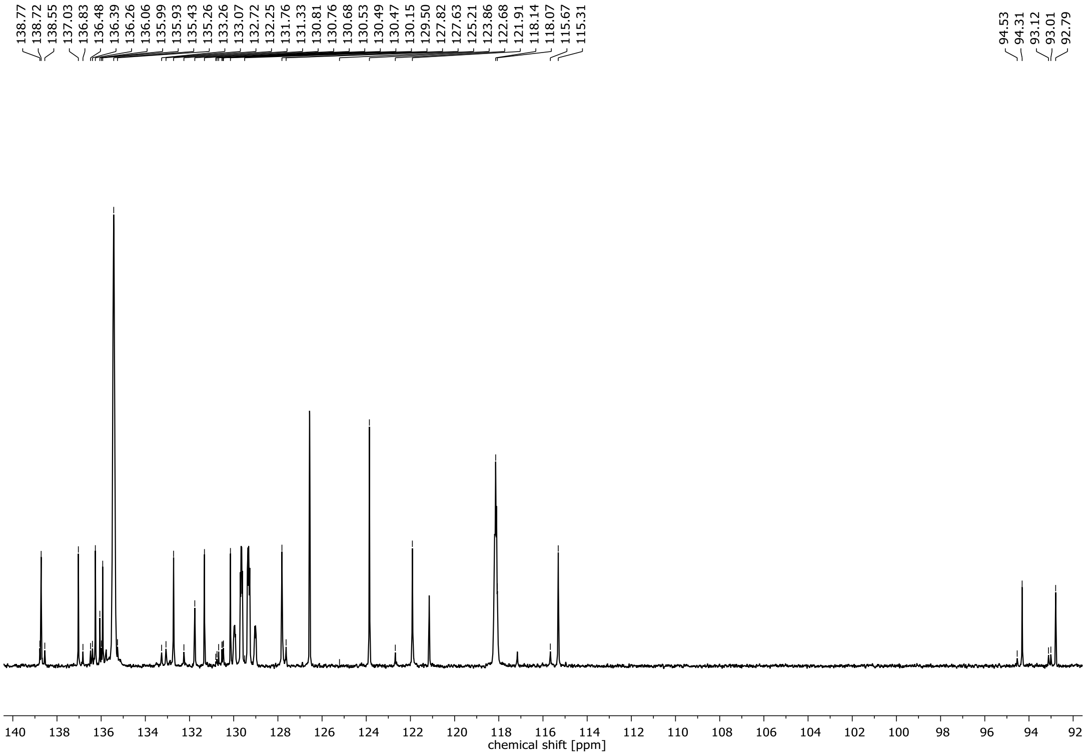

Figure S147. ^{13}C NMR spectrum of **7d$^{\mathbf{BArF}}$** (fraction 2), zoom into aromatic region (101 MHz, DCM-d_2).

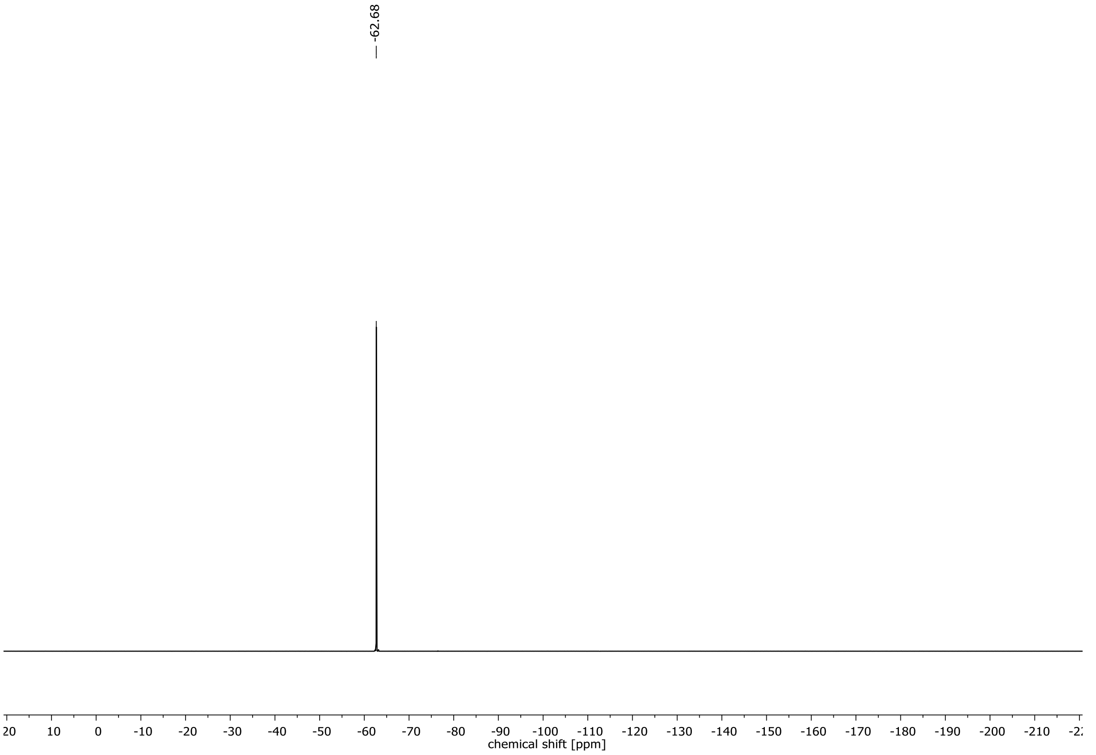

Figure S148. ^{19}F NMR spectrum of **7d$^{\mathbf{BArF}}$** (fraction 2) (376 MHz, DCM-d_2).

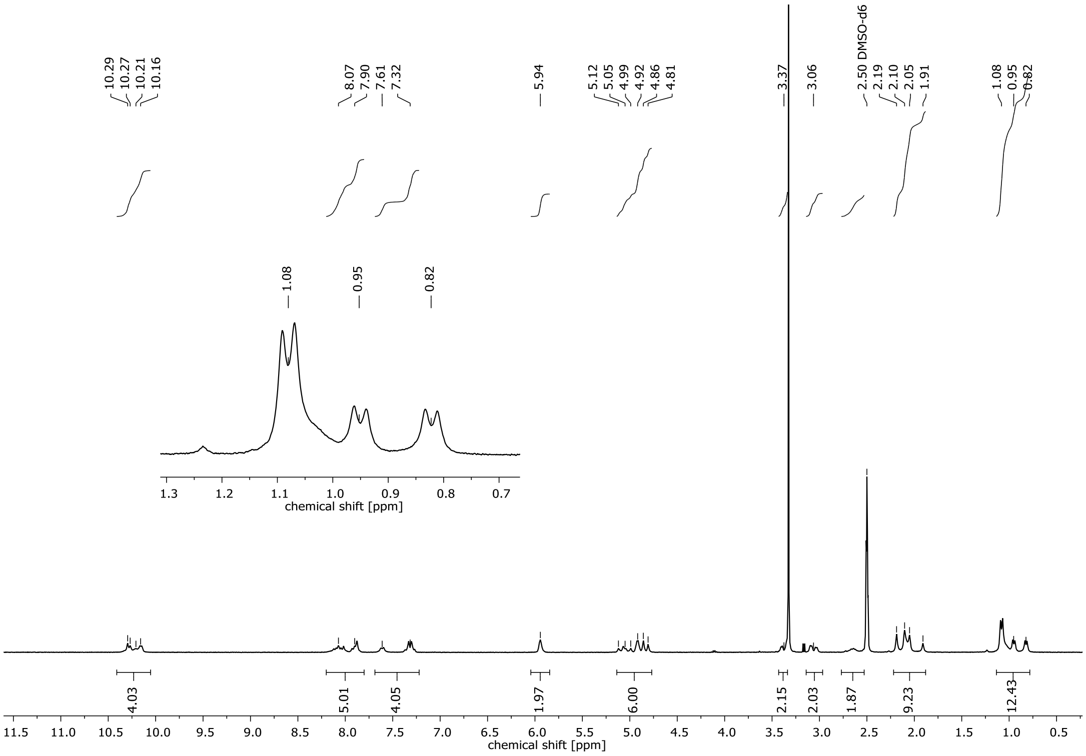

Figure S149. ^{1}H NMR spectrum of **5e^{Cl}** (300 MHz, DMSO-d_6). Cut-out detailing overlapping signals for inequivalent methyl groups shown.

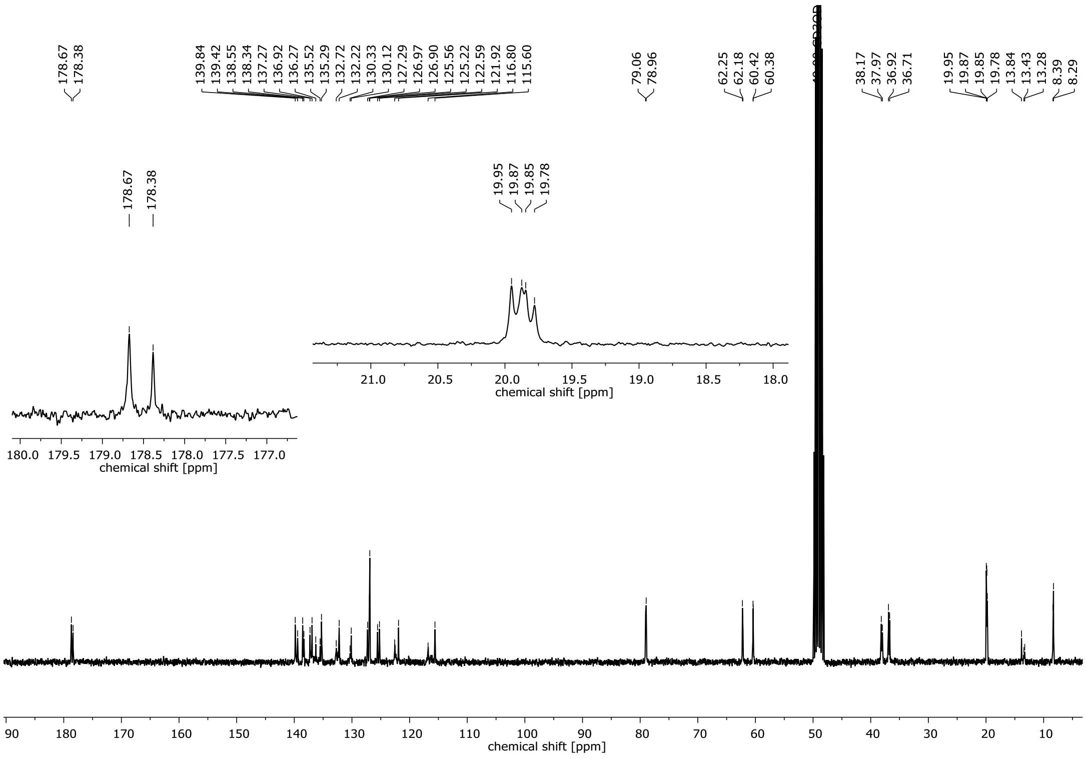

Figure S150. ^{13}C NMR spectrum of **5e^{Cl}** (75 MHz, methanol-d_4). Cut-outs detailing two signals for carbonyl carbons and inequivalent isopropyl methyl groups are highlighted.

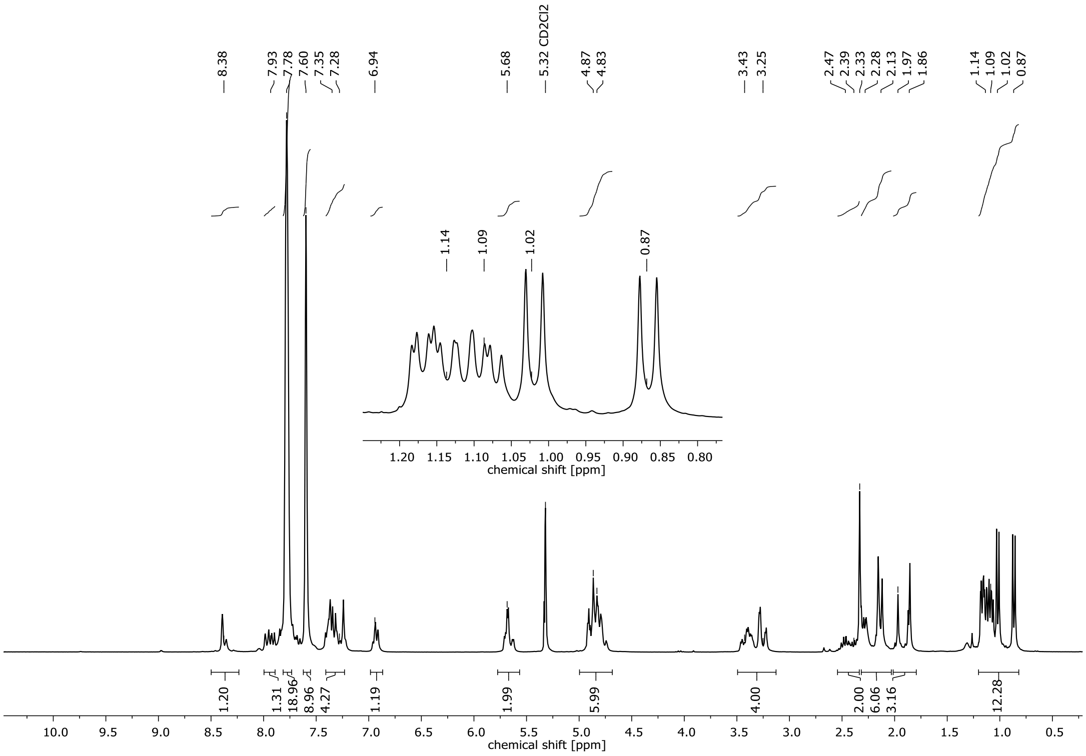

Figure S151. ^{1}H NMR spectrum of $\mathbf{7e^{BArF}}$ (400 MHz, DCM-d_2). Cut-out detailing complex signals for isopropyl methyl groups shown.

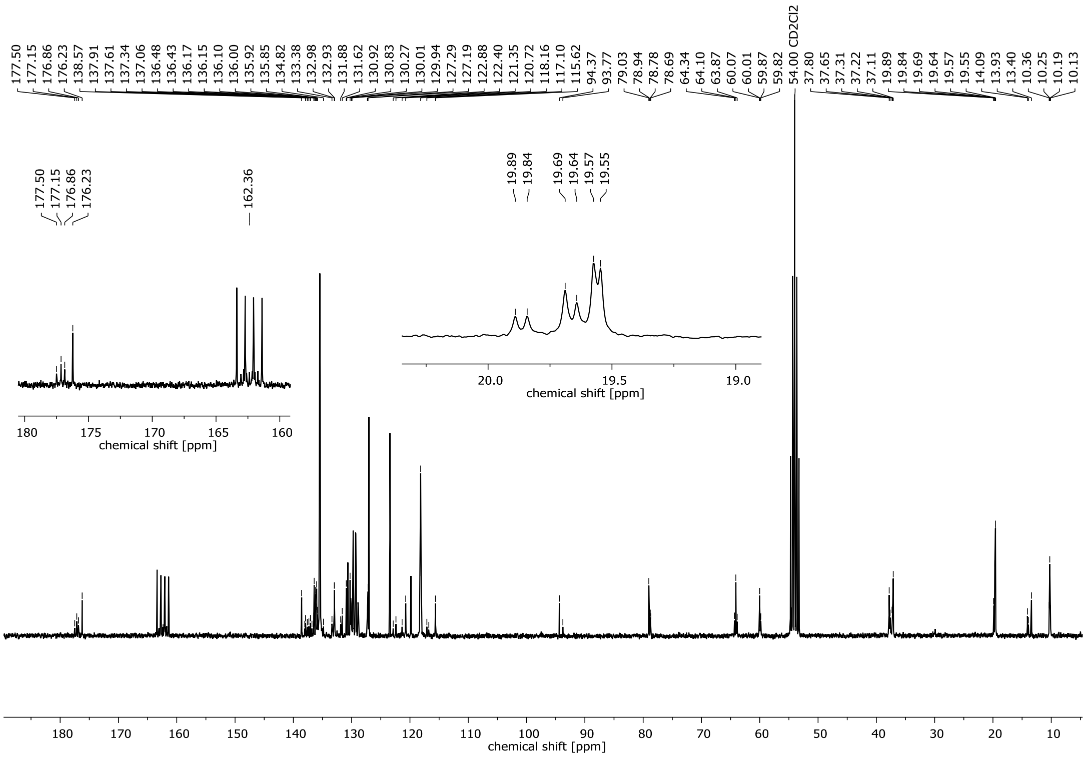

Figure S152. ^{13}C NMR spectrum of $\mathbf{7e^{BArF}}$ (75 MHz, DCM-d_2).

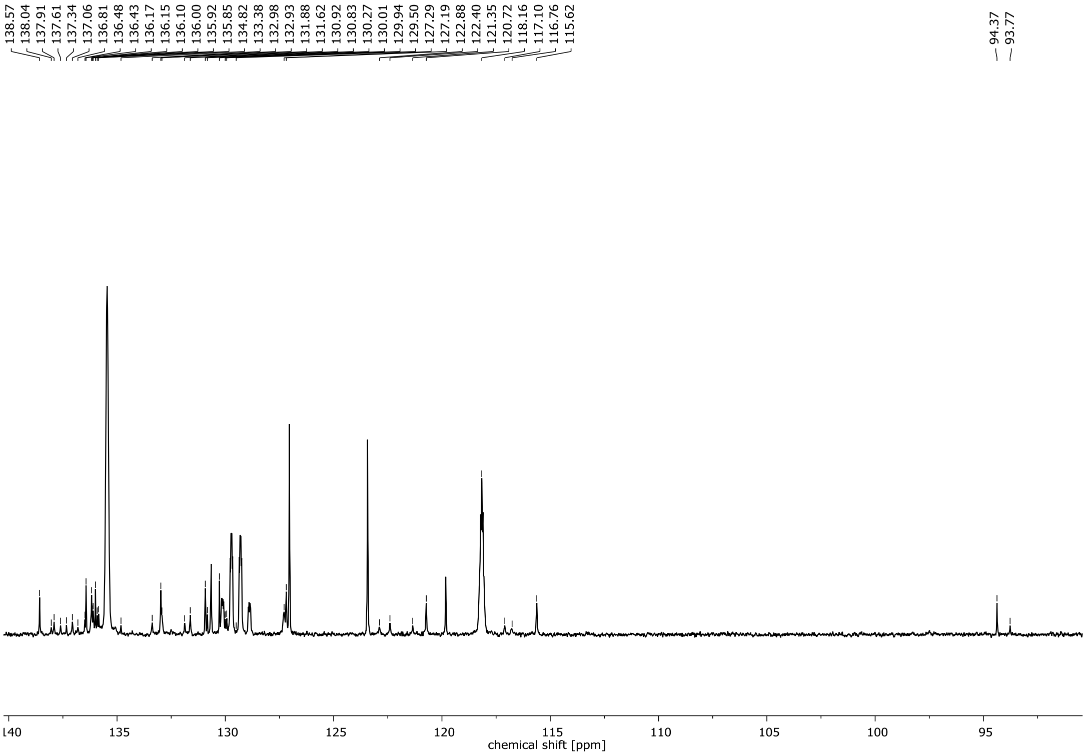

Figure S153. ^{13}C NMR spectrum of **$7e^{BArF}$**, zoom into aromatic region (75 MHz, DCM-d_2).

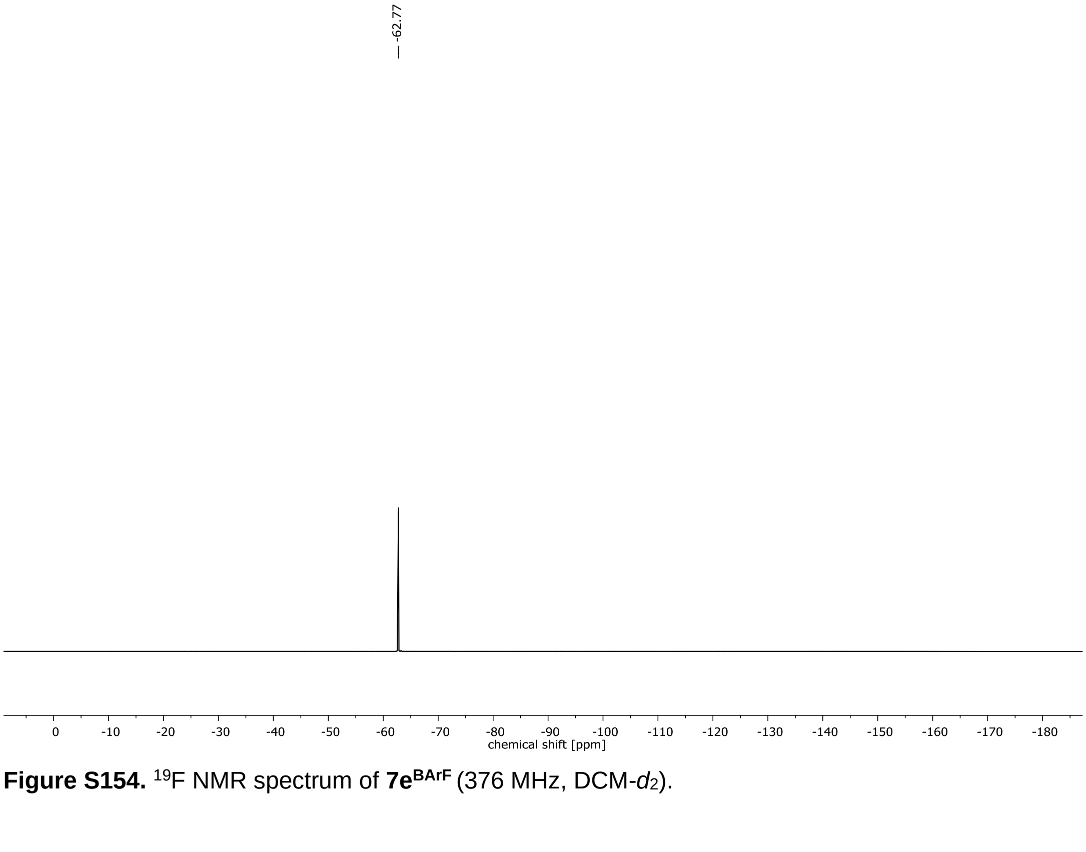

Figure S154. ^{19}F NMR spectrum of **$7e^{BArF}$** (376 MHz, DCM-d_2).

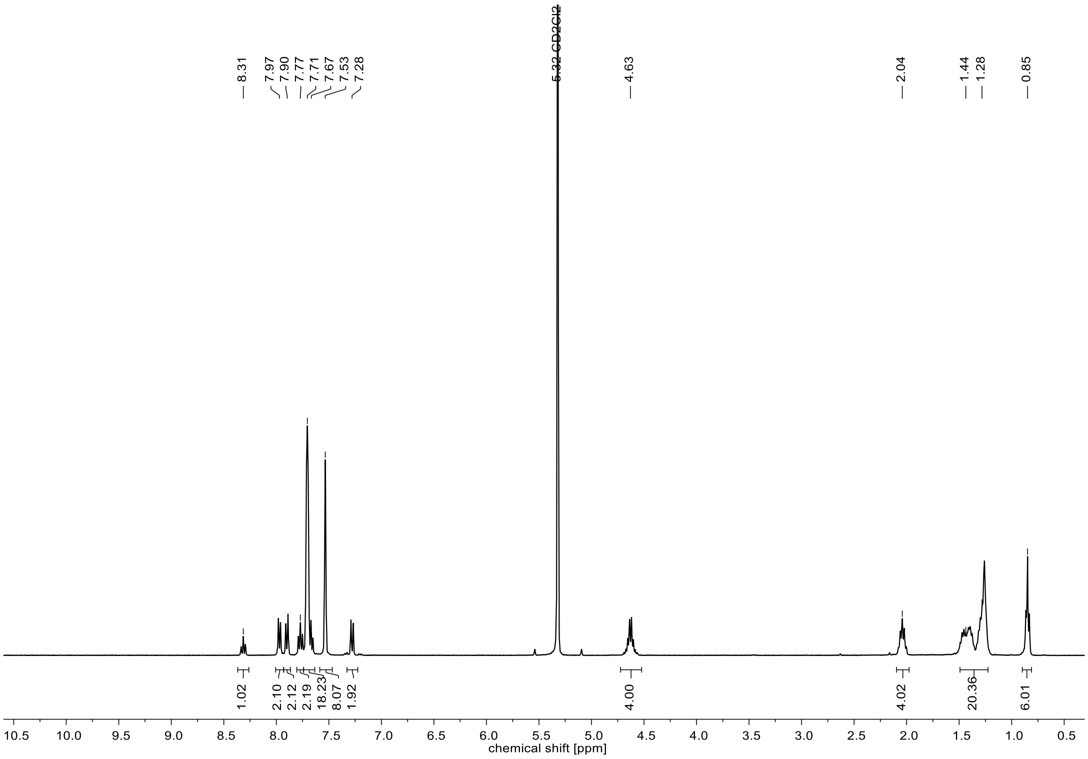

Figure S155. ^{1}H NMR spectrum of **SI-9** (400 MHz, DCM-d_2).

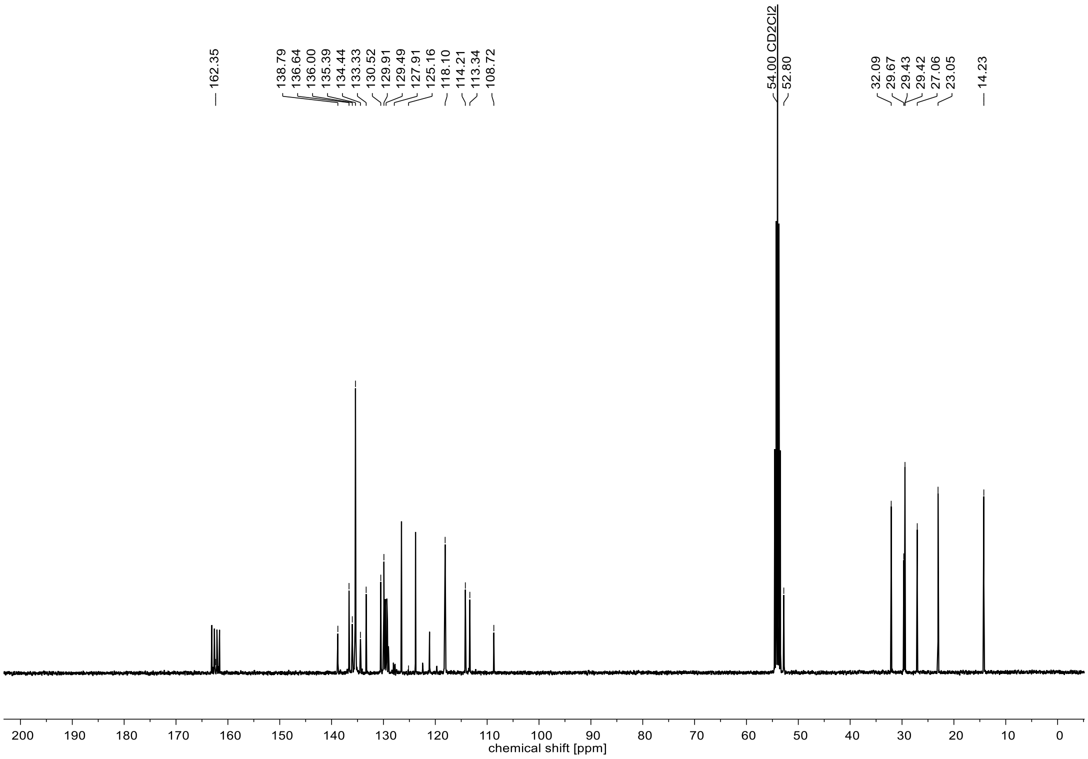

Figure S156. ^{13}C NMR spectrum of **SI-9** (101 MHz, DCM-d_2).

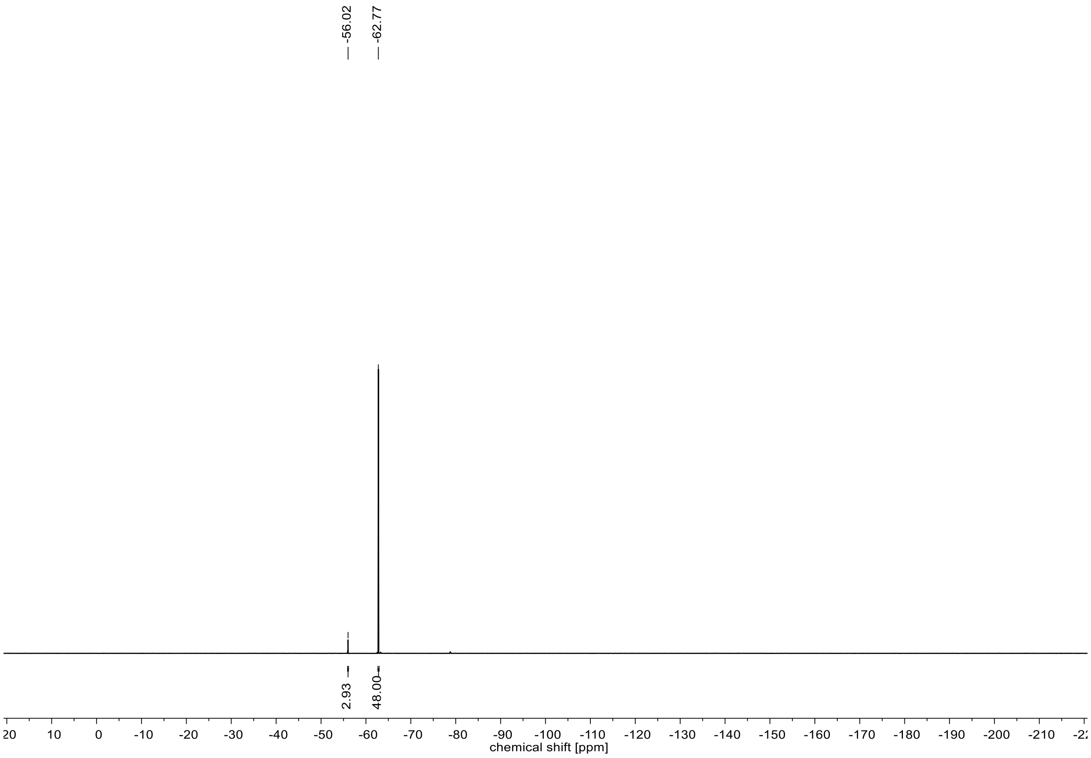

Figure S157. ^{19}F NMR spectrum of **SI-9** (376 MHz, DCM-d_2).

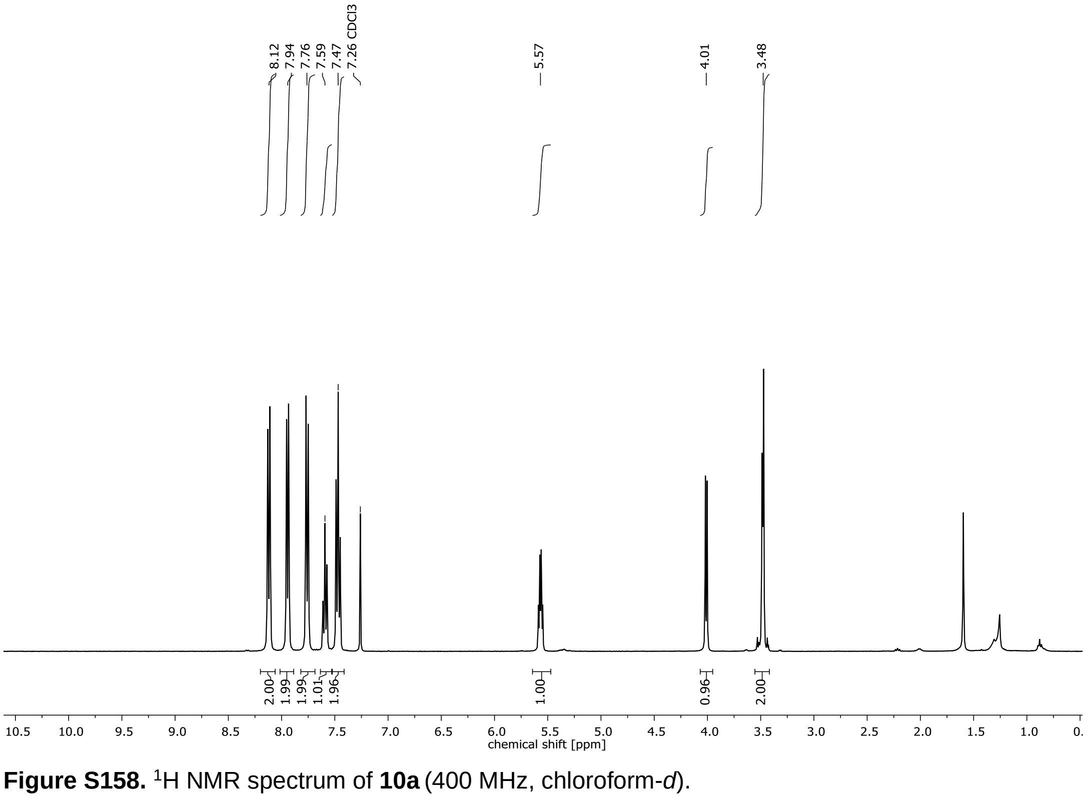

Figure S158. ^{1}H NMR spectrum of **10a** (400 MHz, chloroform-d).

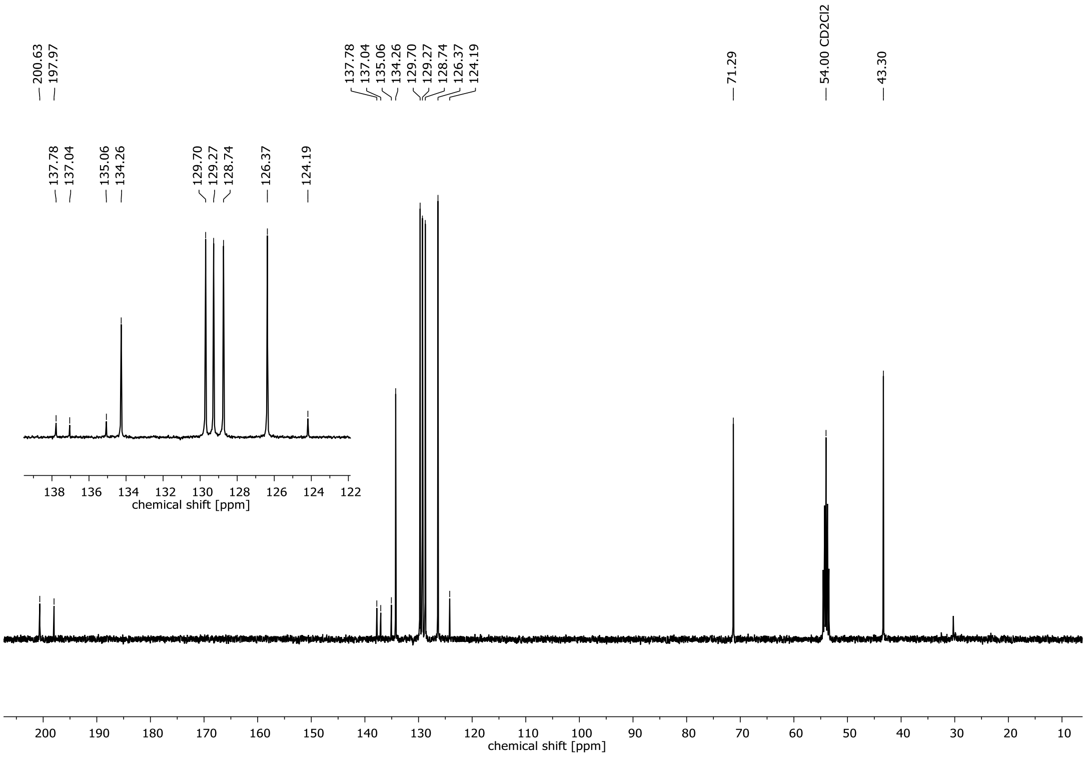

Figure S159. ^{13}C {^{1}H, ^{19}F} NMR spectrum of **10a** (101 MHz, DCM-d_2).

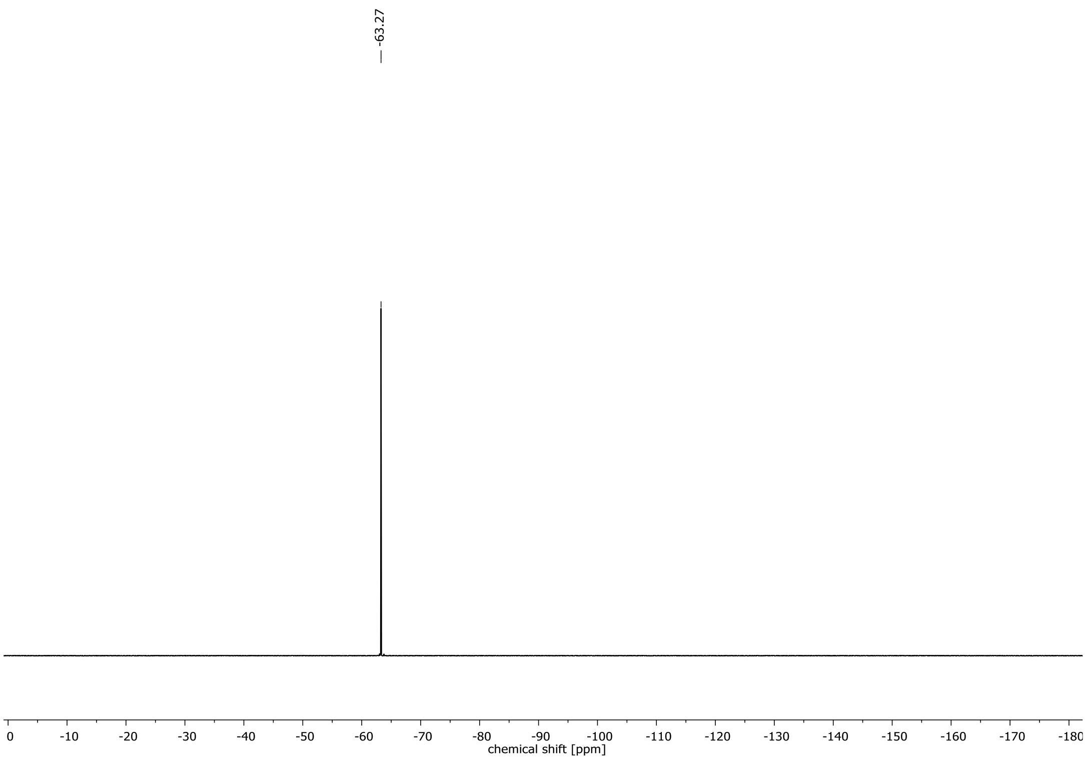

Figure S160. ^{19}F NMR spectrum of **10a** (376 MHz, chloroform-*d*).

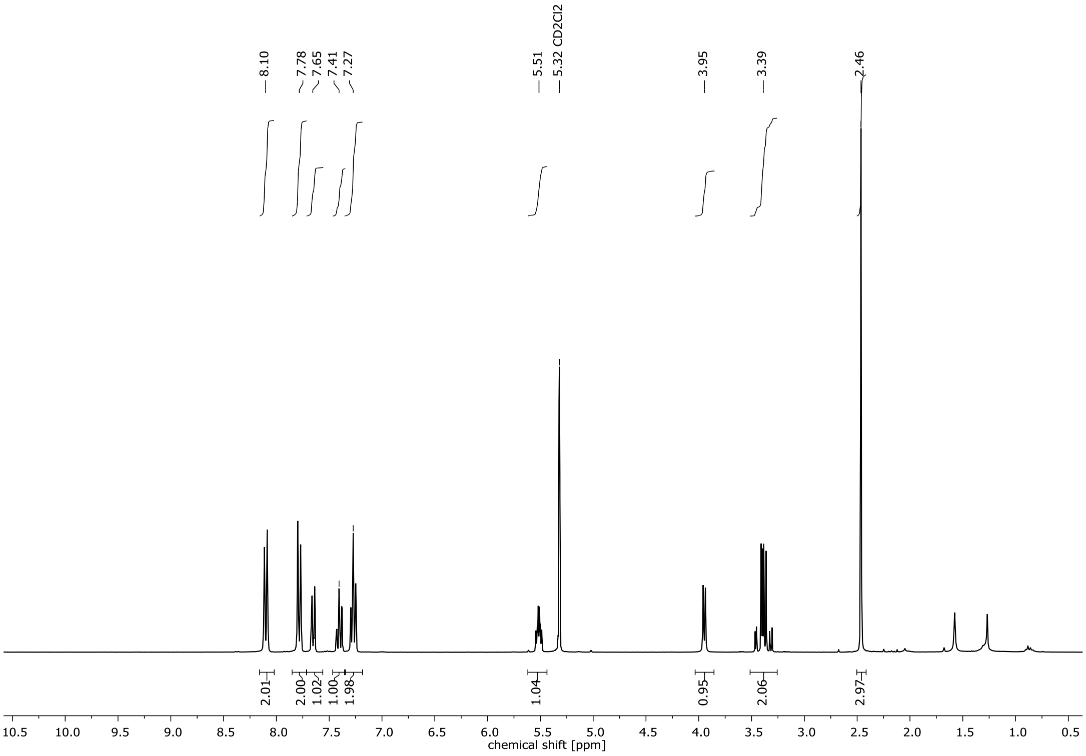

Figure S161. ^{1}H NMR spectrum of **10b** (300 MHz, DCM-d_2).

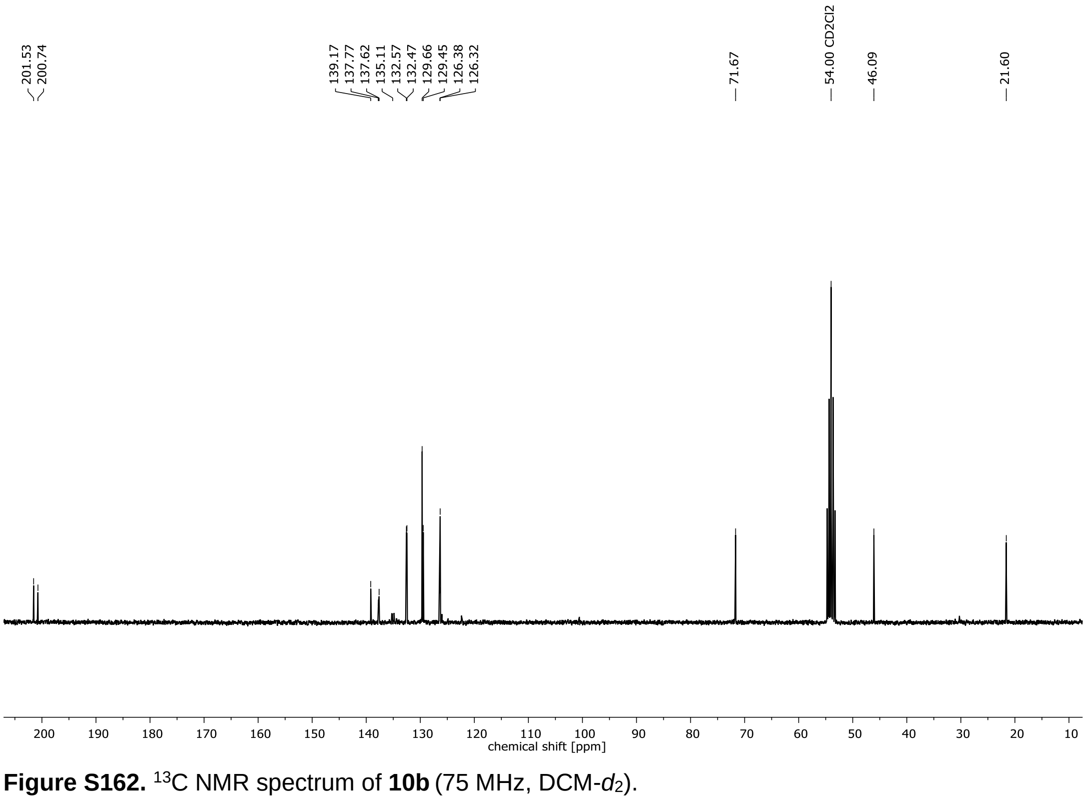

Figure S162. ^{13}C NMR spectrum of **10b** (75 MHz, DCM-d_2).

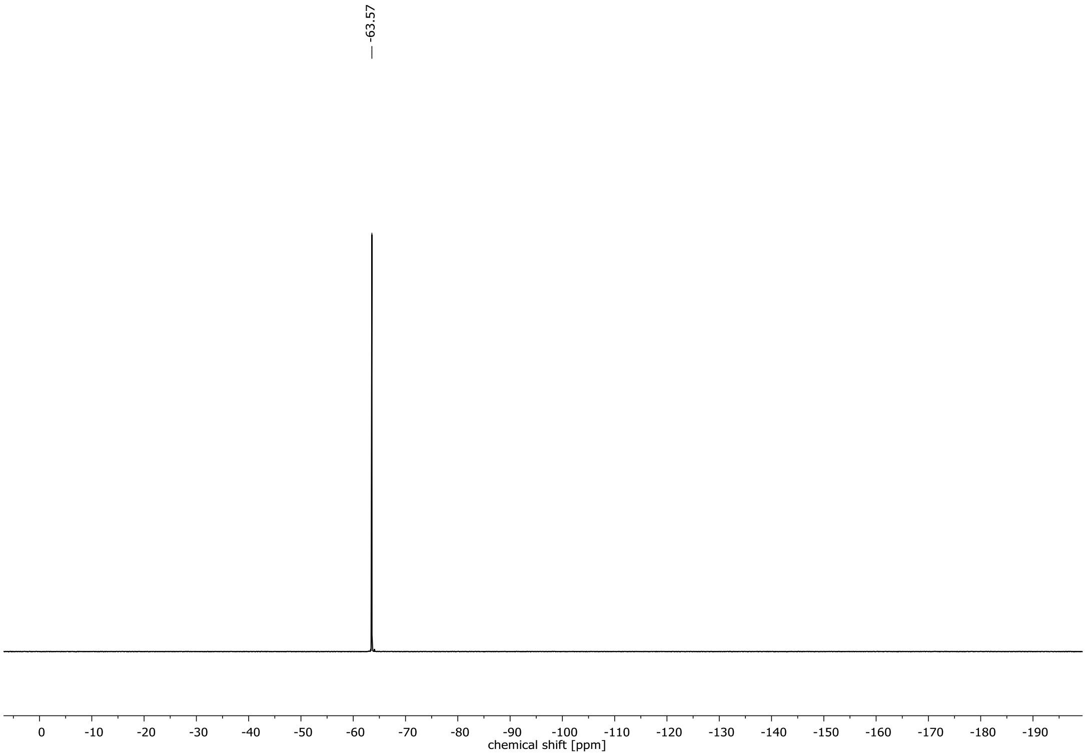

Figure S163. ^{19}F NMR spectrum of **10b** (376 MHz, DCM-d_2).

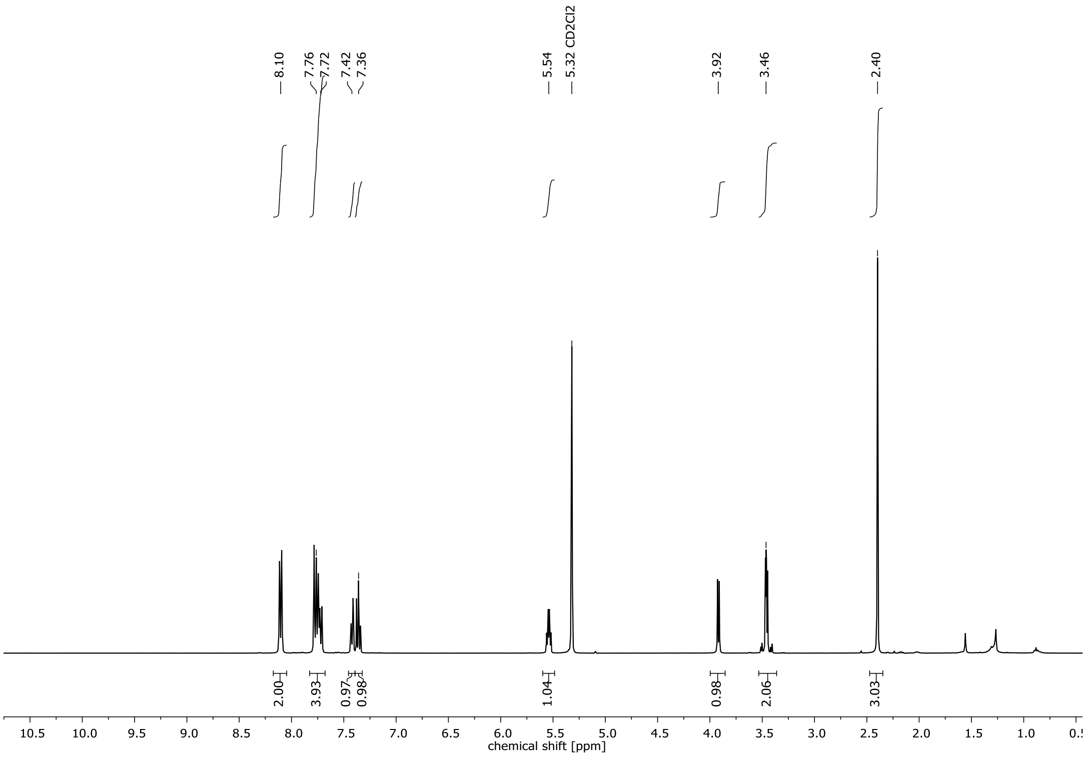

Figure S164. ^{1}H NMR spectrum of **10c** (400 MHz, DCM-d_2).

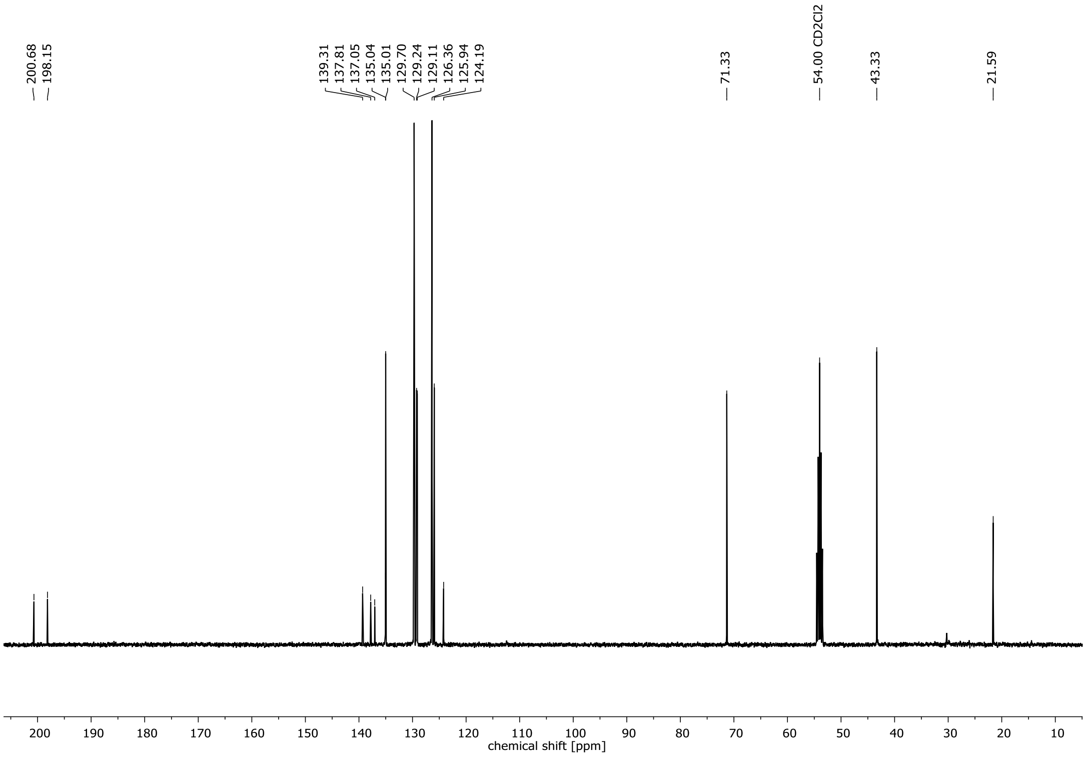

Figure S165. ^{13}C {^{1}H, ^{19}F} NMR spectrum of **10c** (101 MHz, DCM-d_2).

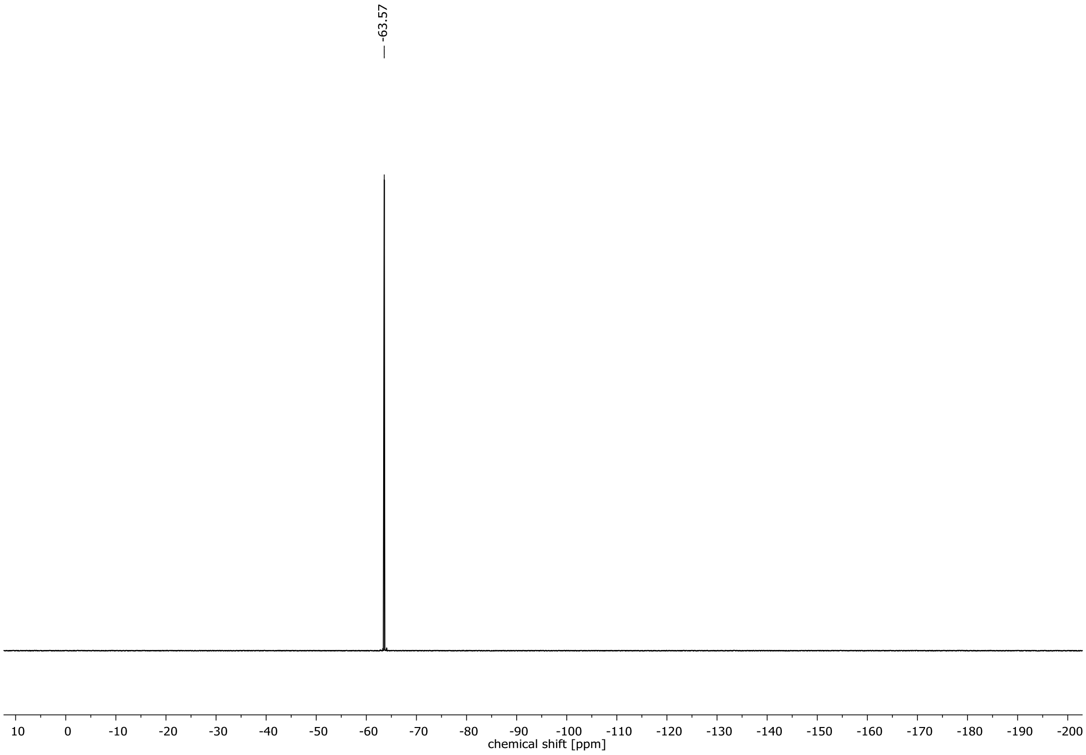

Figure S166. ^{19}F NMR spectrum of **10c** (376 MHz, DCM-d_2).

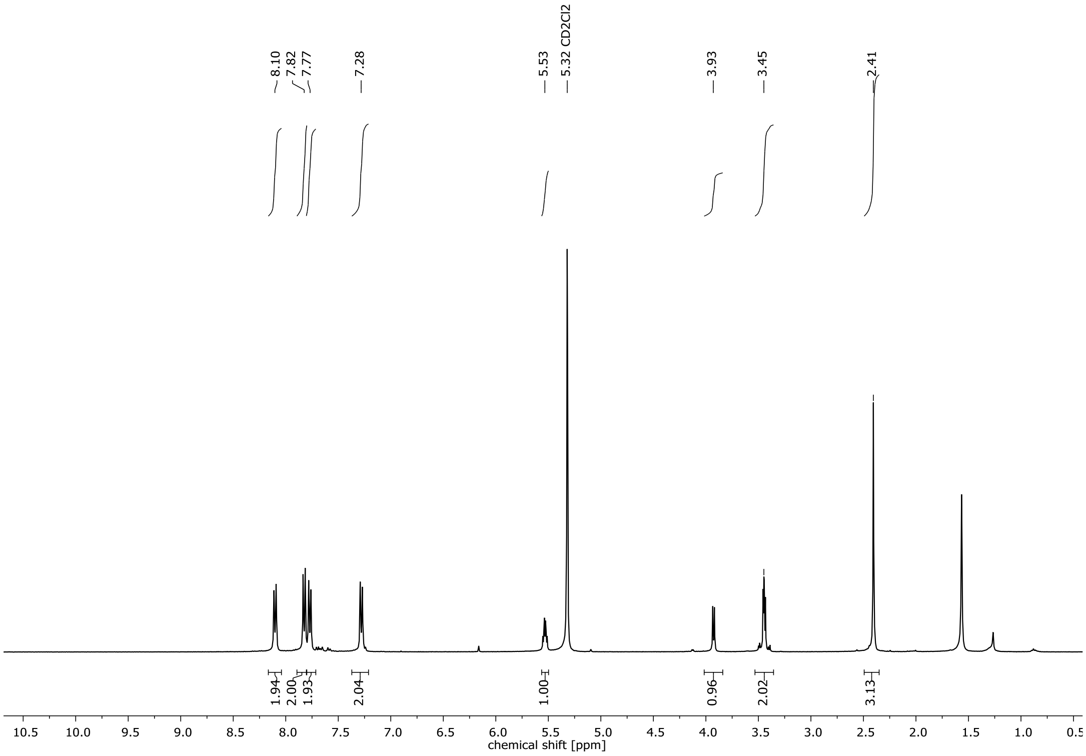

Figure S167. ^{1}H NMR spectrum of **10d** (400 MHz, DCM-d_2).

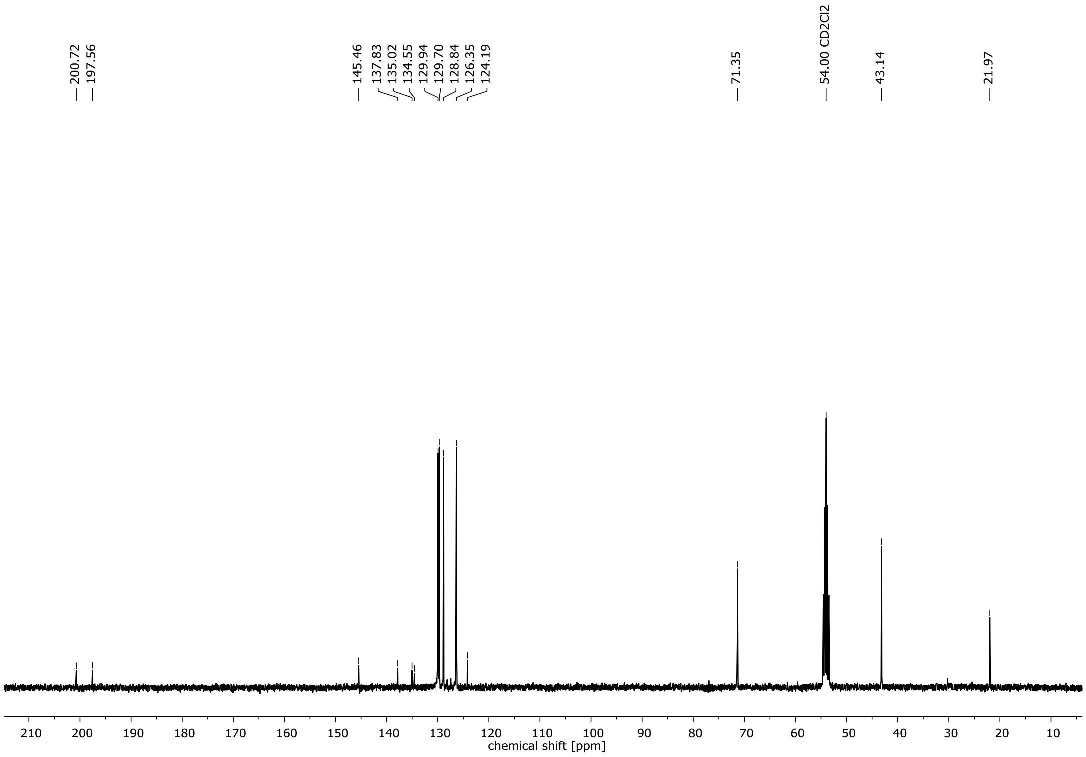

Figure S168. ^{13}C {^{1}H, ^{19}F} NMR spectrum of **10d** (101 MHz, DCM-d_2).

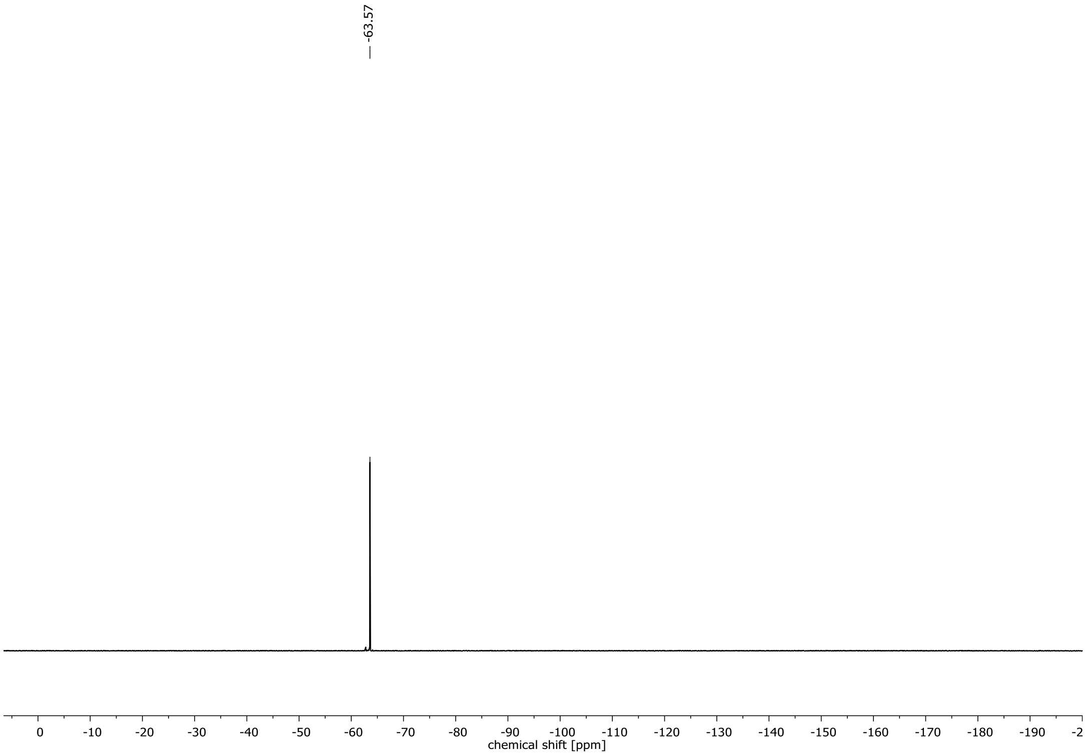

Figure S169. ^{19}F NMR spectrum of **10d** (376 MHz, DCM-d_2).

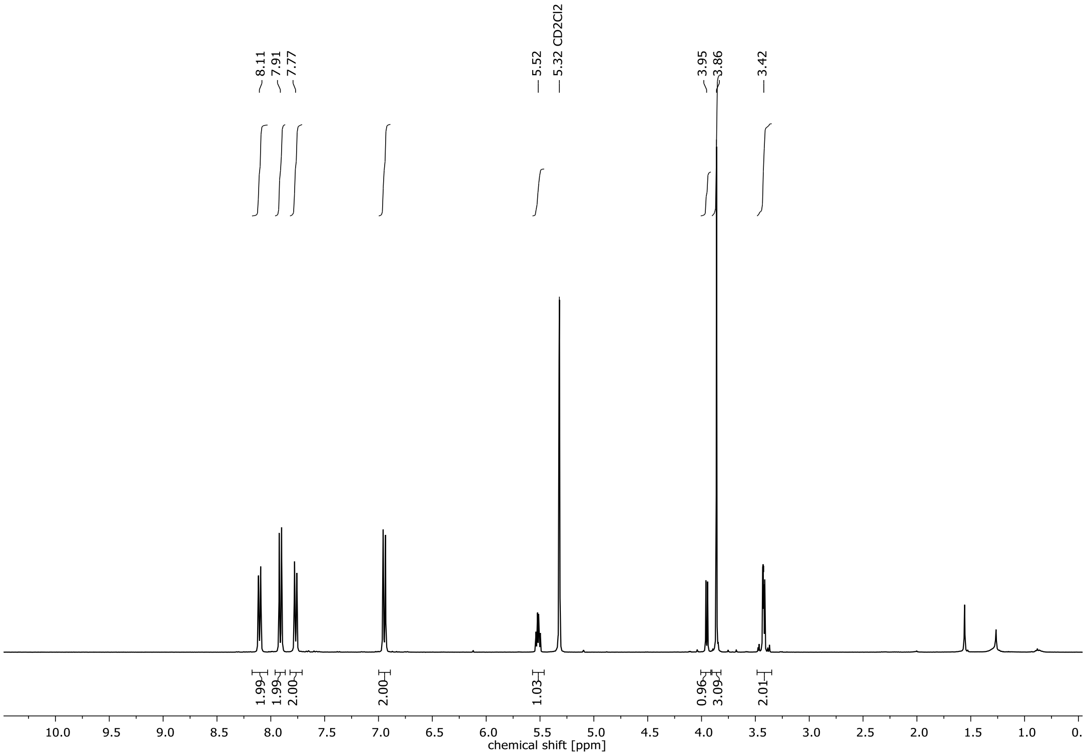

Figure S170. ^{1}H NMR spectrum of **10e** (400 MHz, DCM-d_2).

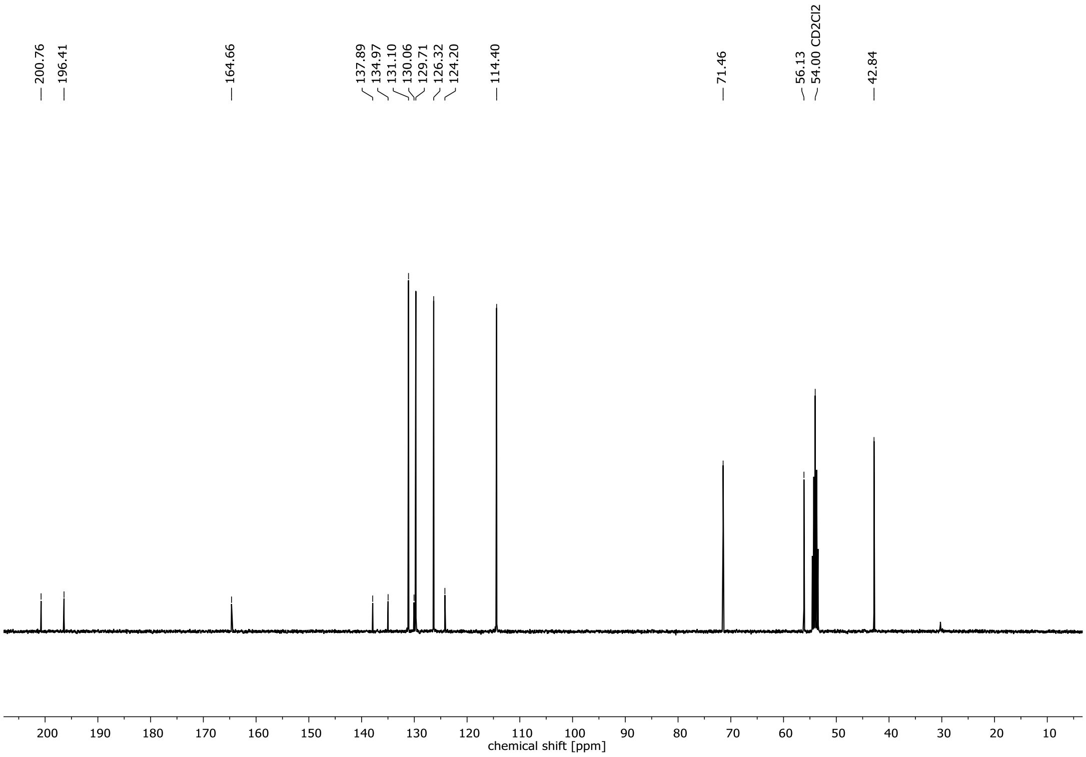

Figure S171. ^{13}C {^{1}H, ^{19}F} NMR spectrum of **10e** (101 MHz, DCM-d_2).

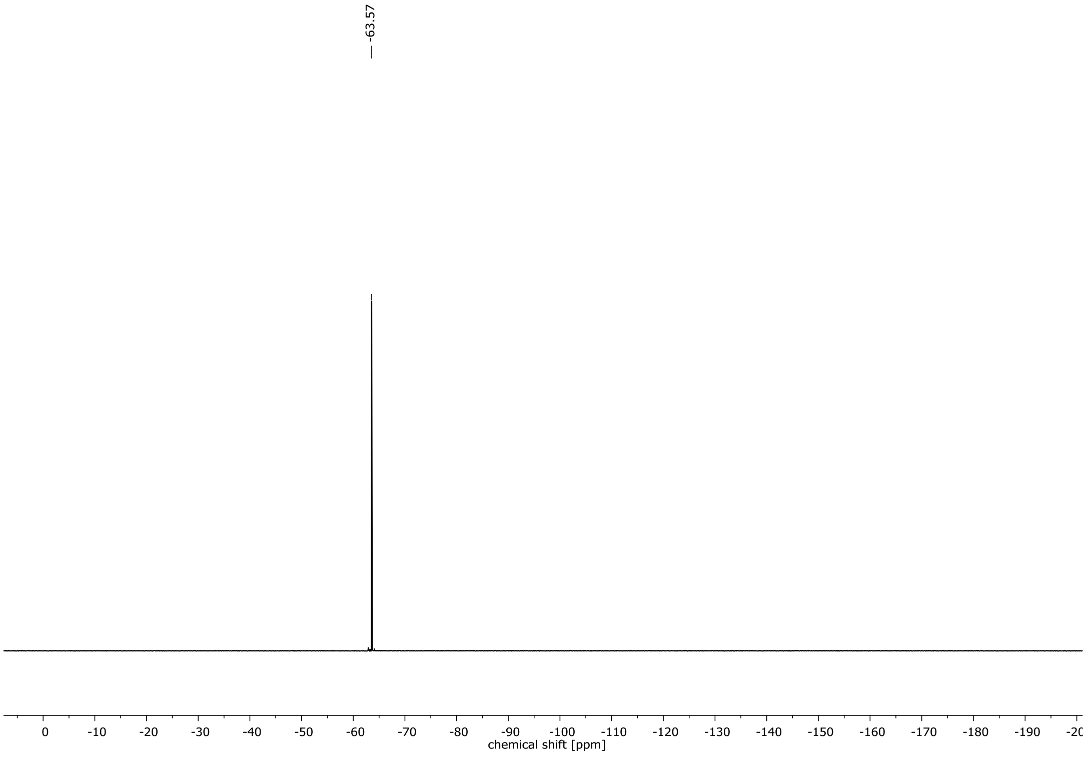

Figure S172. ^{19}F NMR spectrum of **10e** (376 MHz, DCM-d_2).

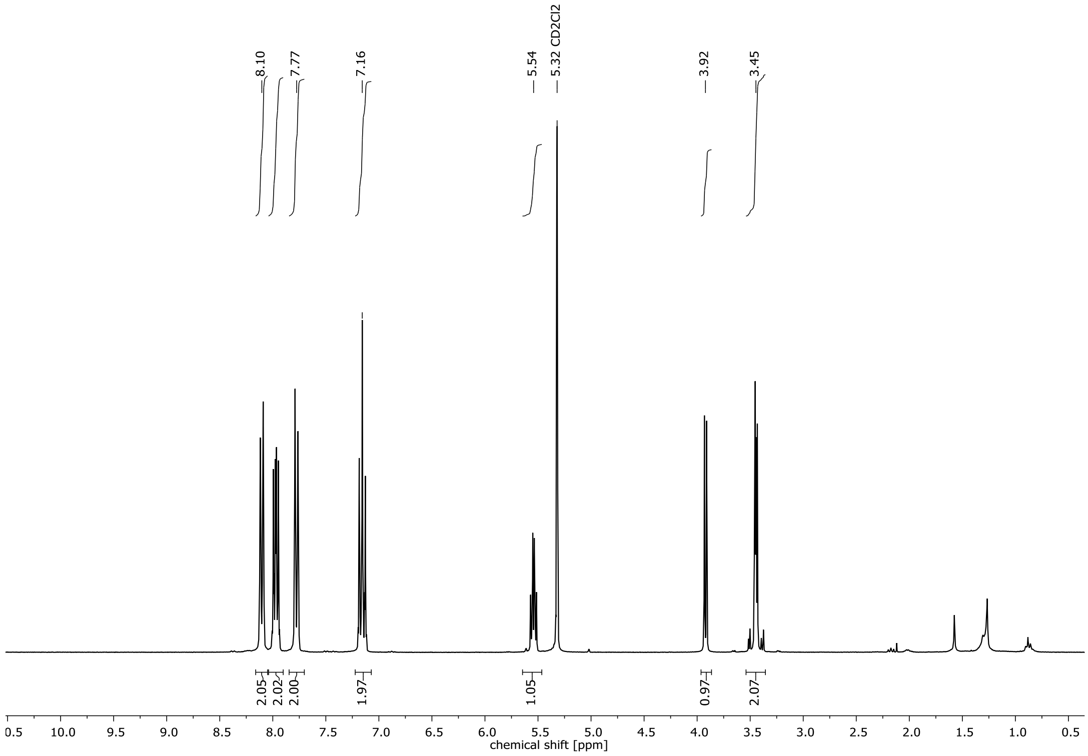

Figure S173. ^{1}H NMR spectrum of **10f** (300 MHz, DCM-d_2).

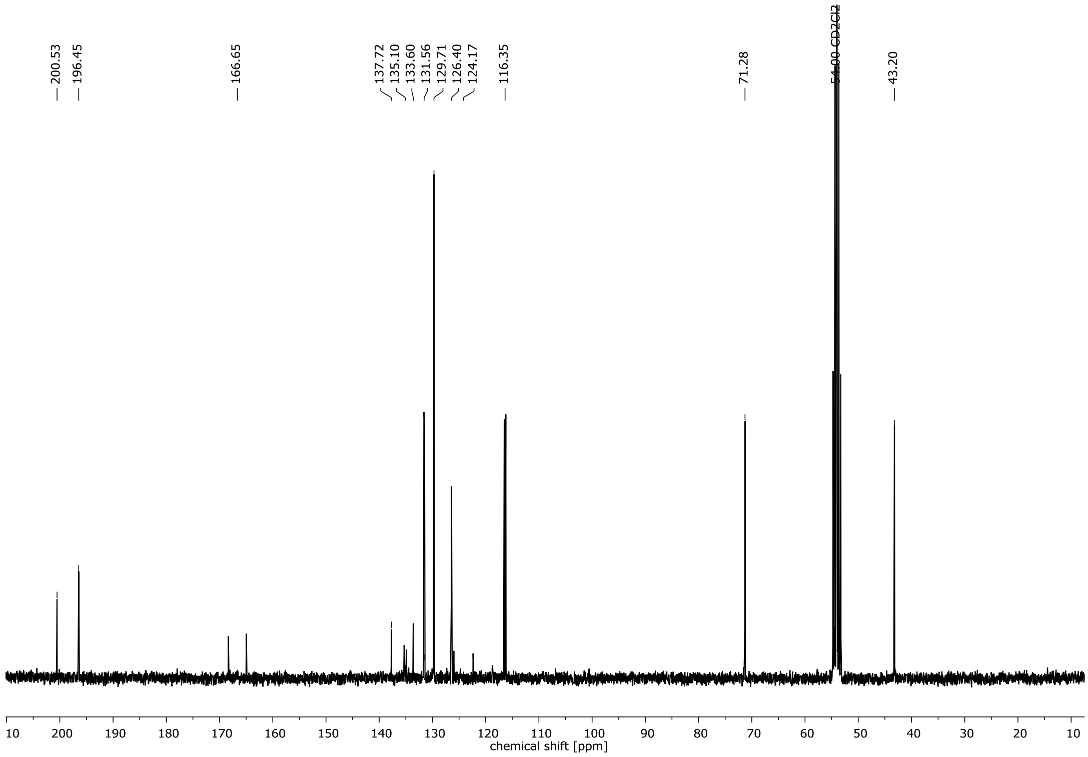

Figure S174. ^{13}C NMR spectrum of **10f** (75 MHz, DCM-d_2).

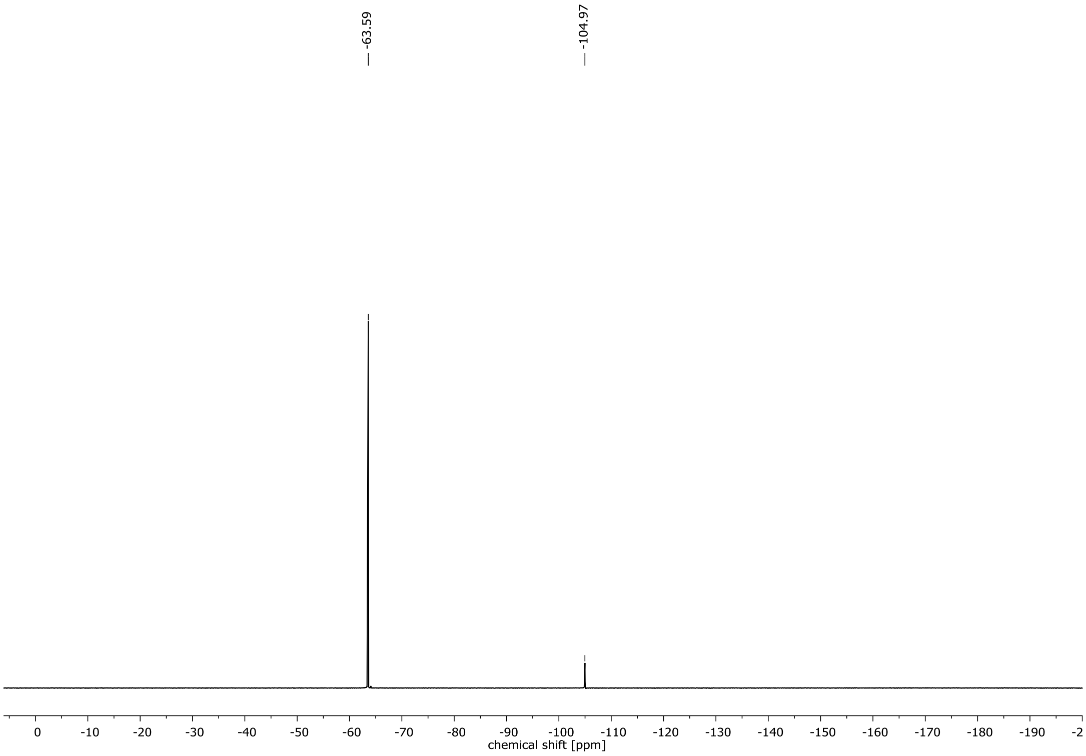

Figure S175. ^{19}F NMR spectrum of **10f** (376 MHz, DCM-d_2).

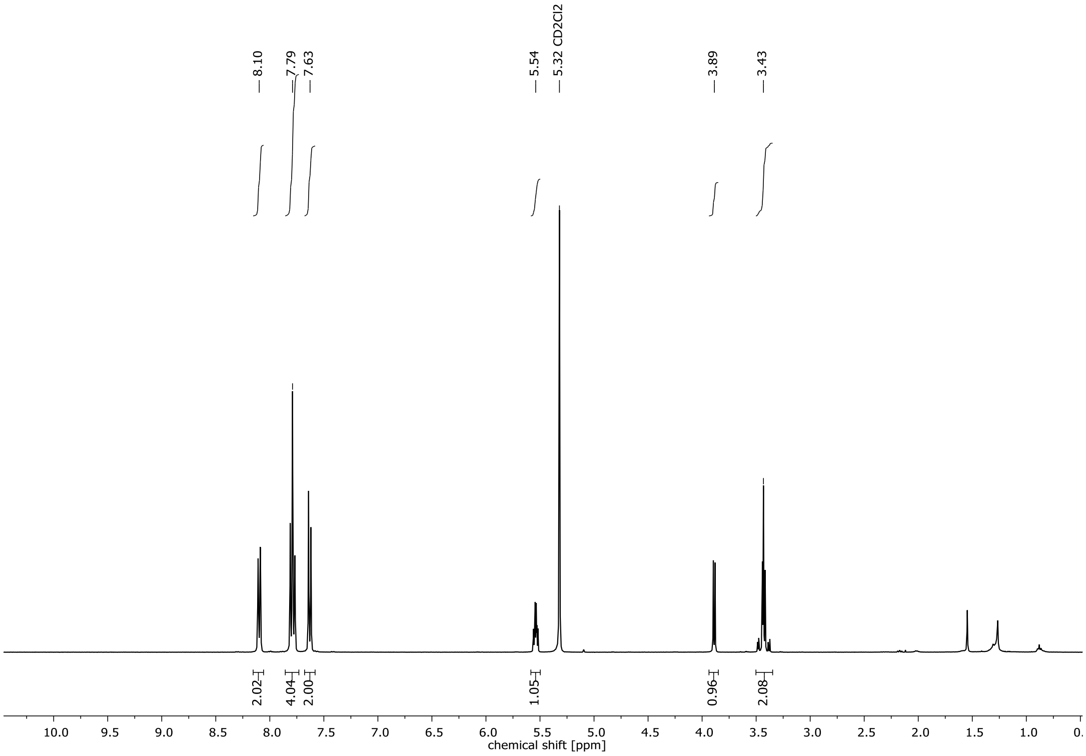

Figure S176. ^{1}H NMR spectrum of **10g** (400 MHz, DCM-d_2).

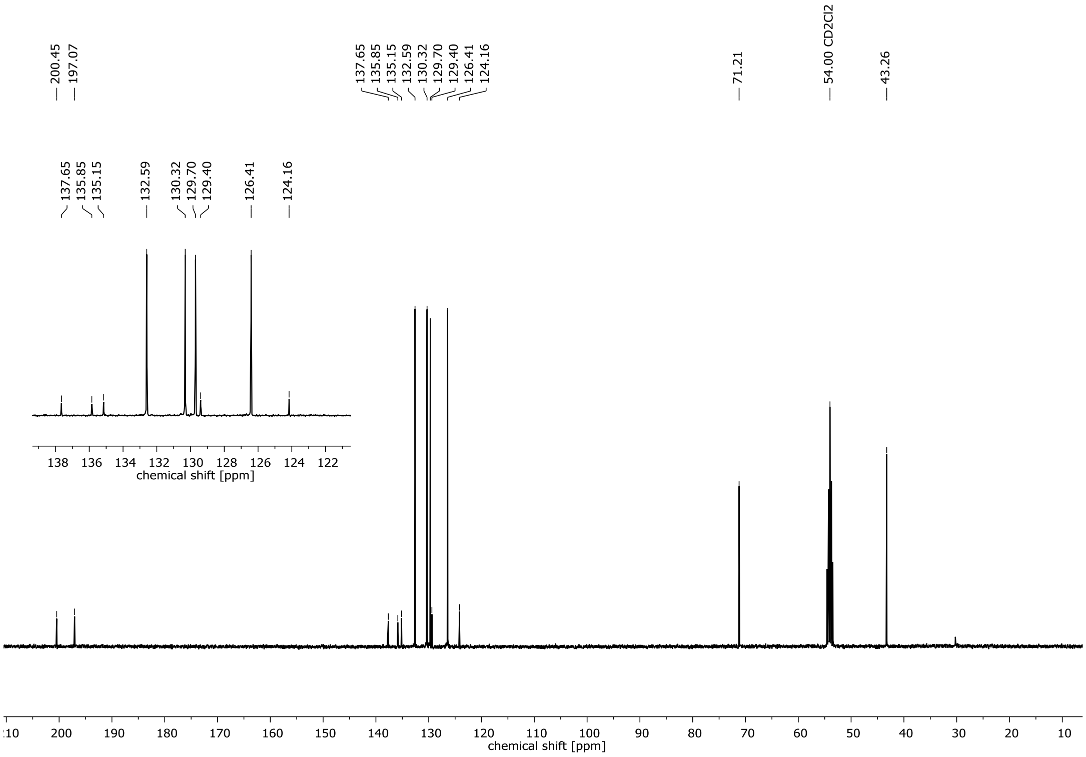

Figure S177. ^{13}C {^{1}H, ^{19}F} NMR spectrum of **10g** (101 MHz, DCM-d_2).

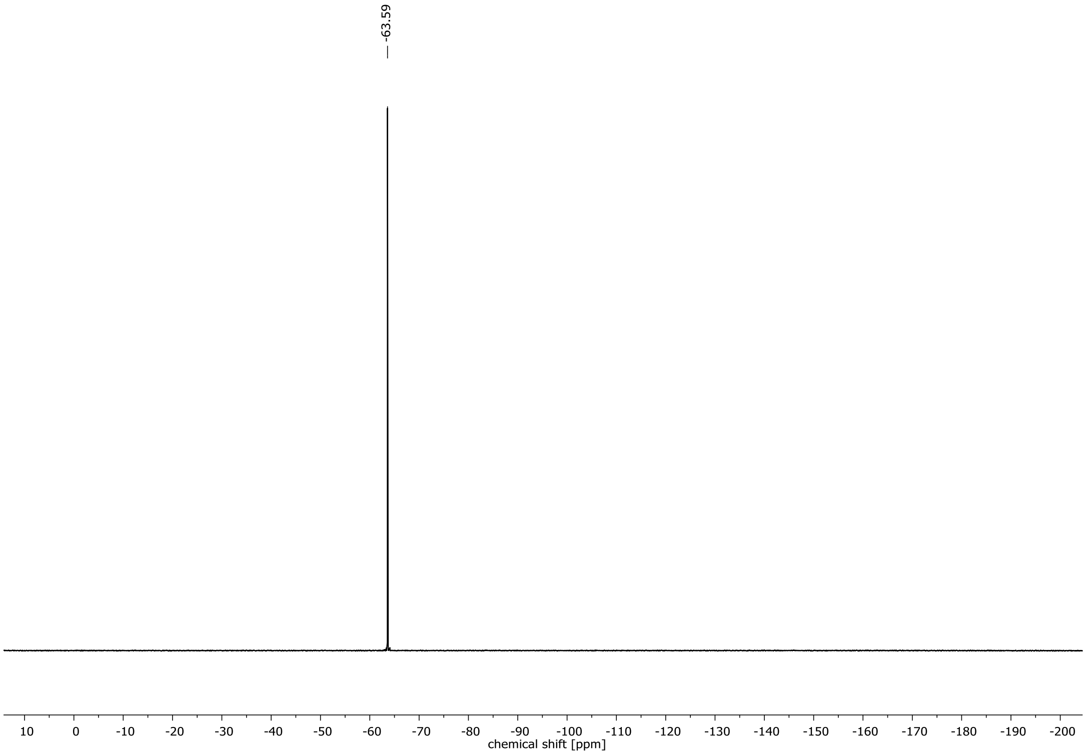

Figure S178. ^{19}F NMR spectrum of **10g** (376 MHz, DCM-d_2).

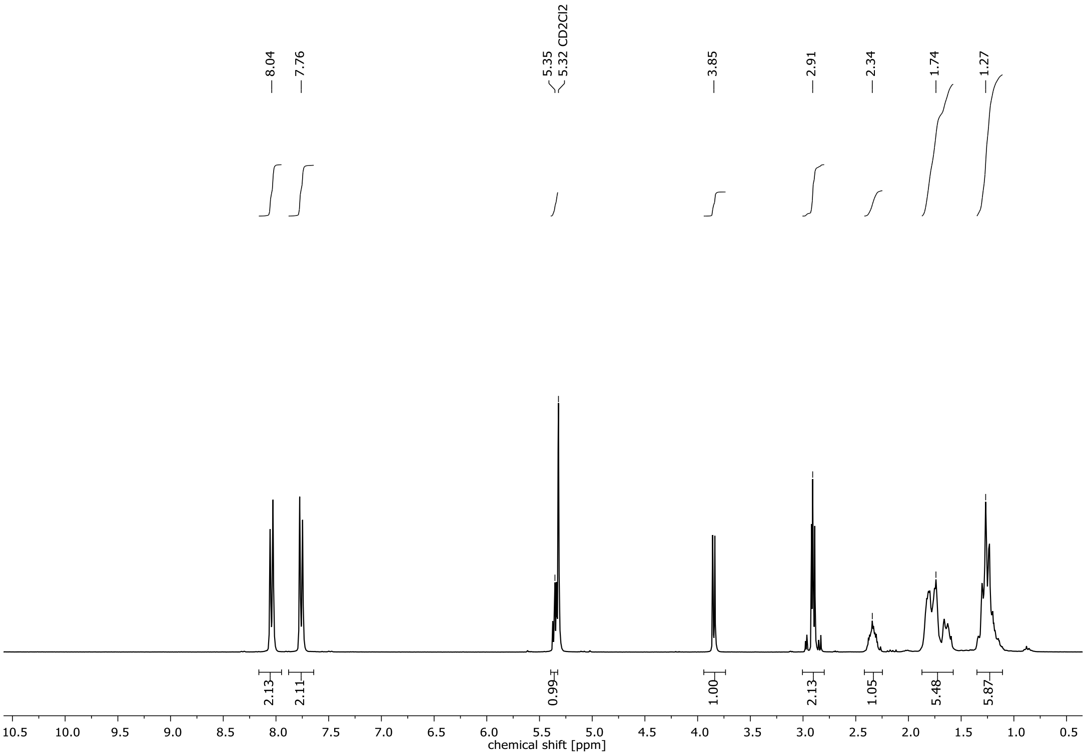

Figure S179. ^{1}H NMR spectrum of **10h** (300 MHz, DCM-d_2). Grease residue present in the sample led to slight deviation of the integral for the signal centered at 1.3 ppm.

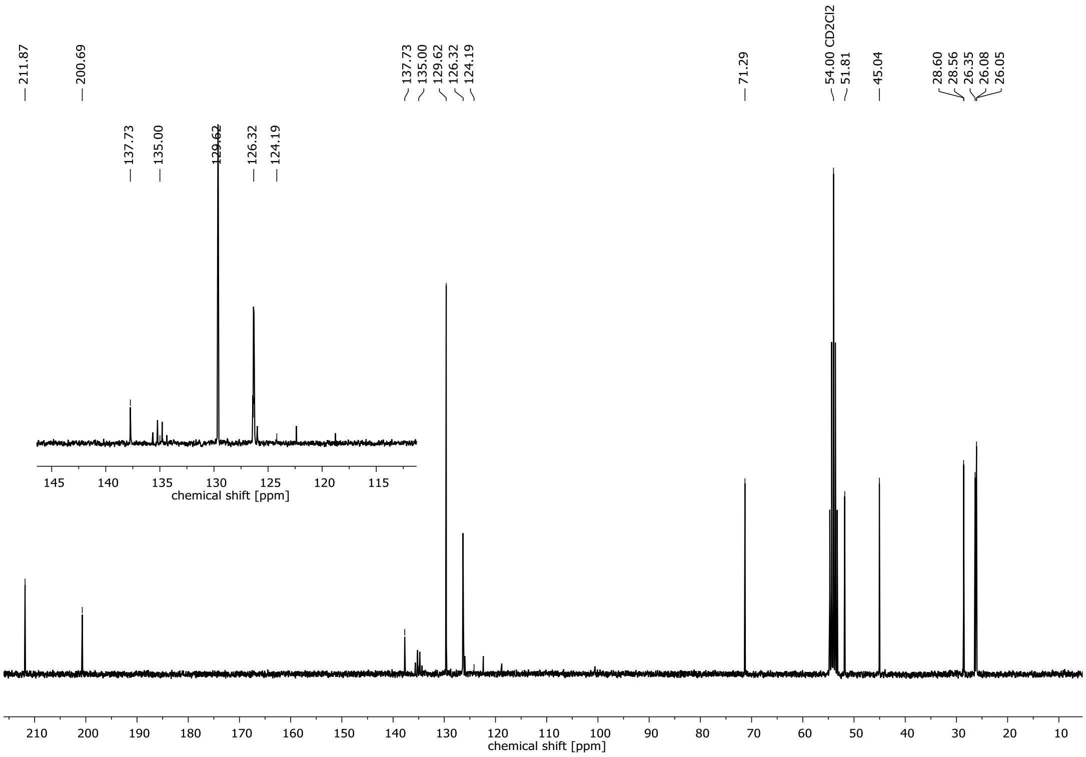

Figure S180. ^{13}C NMR spectrum of **10h** (75 MHz, DCM-d_2).

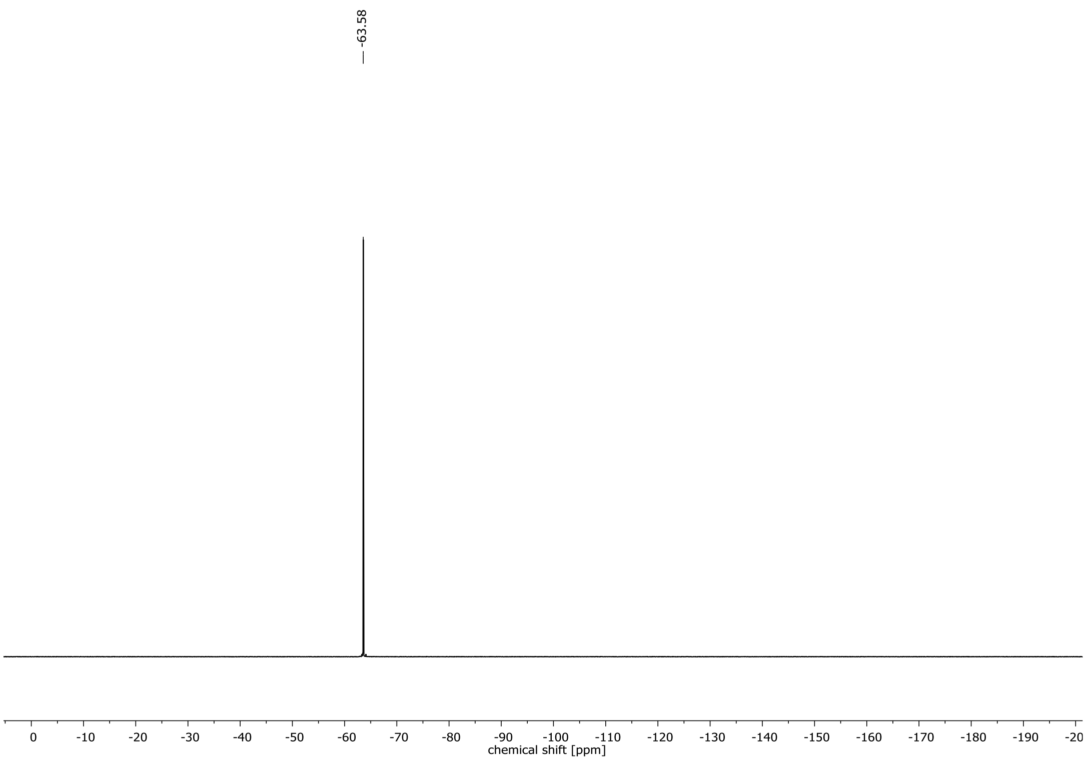

Figure S181. ^{19}F NMR spectrum of **10h** (376 MHz, DCM-d_2).

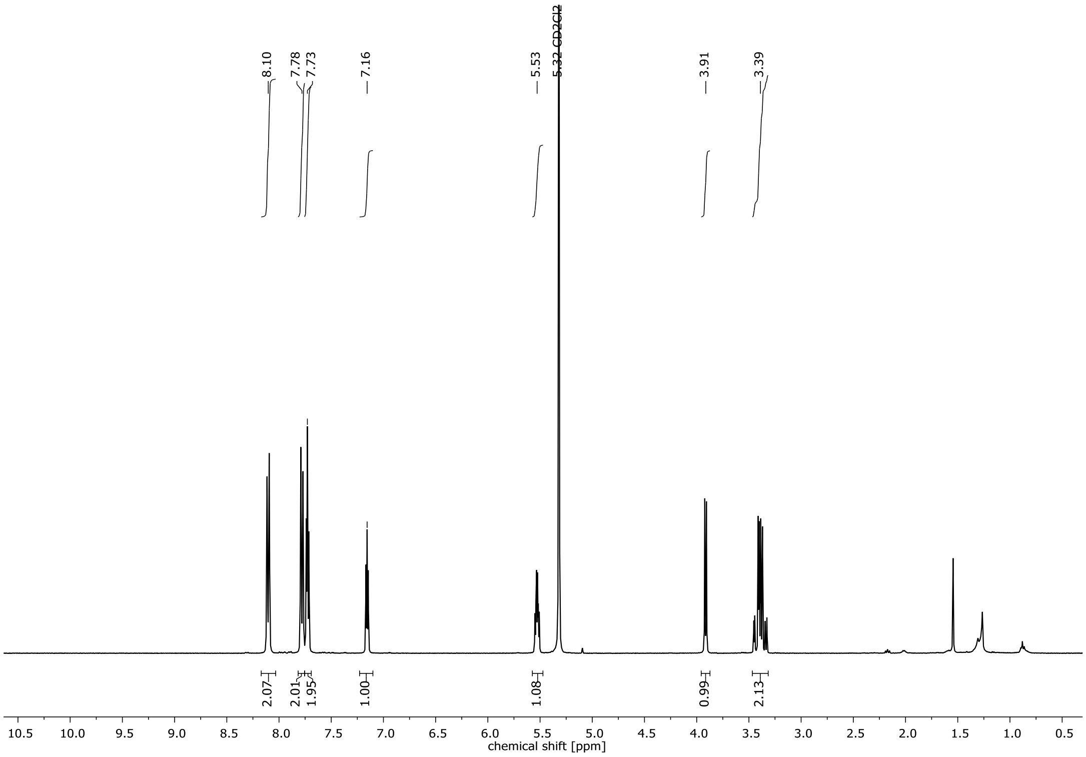

Figure S182. ^{1}H NMR spectrum of **10i** (400 MHz, DCM-d_2).

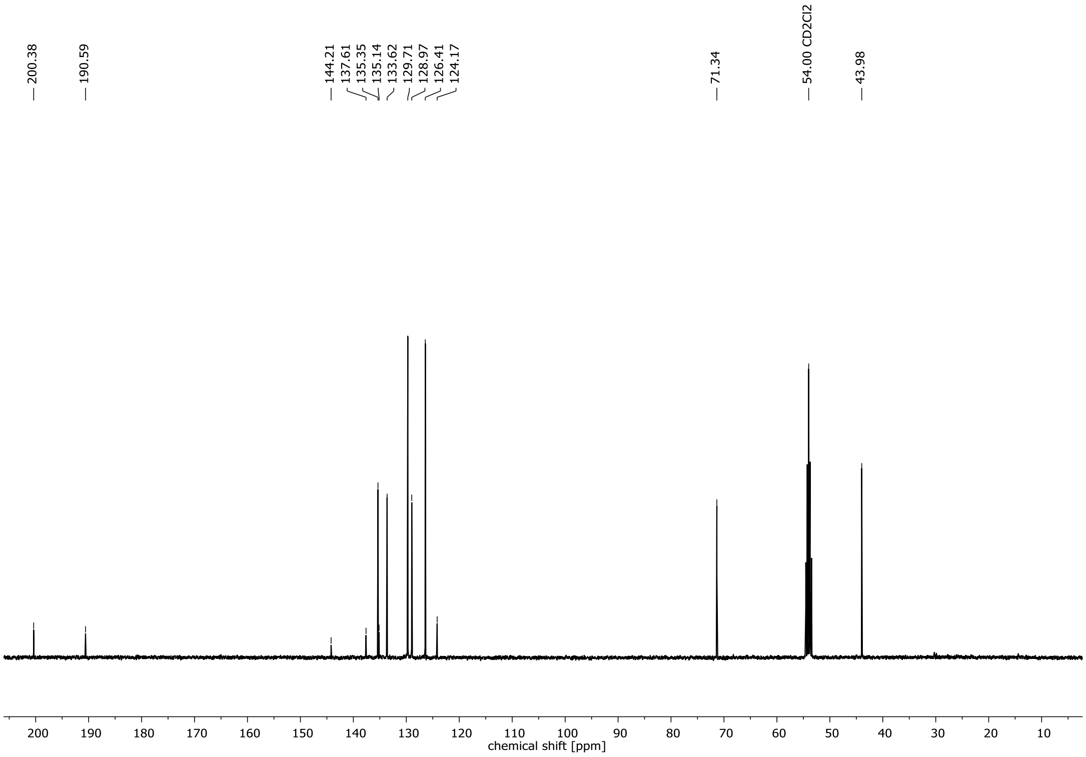

Figure S183. ^{13}C {^{1}H, ^{19}F} NMR spectrum of **10i** (101 MHz, DCM-d_2).

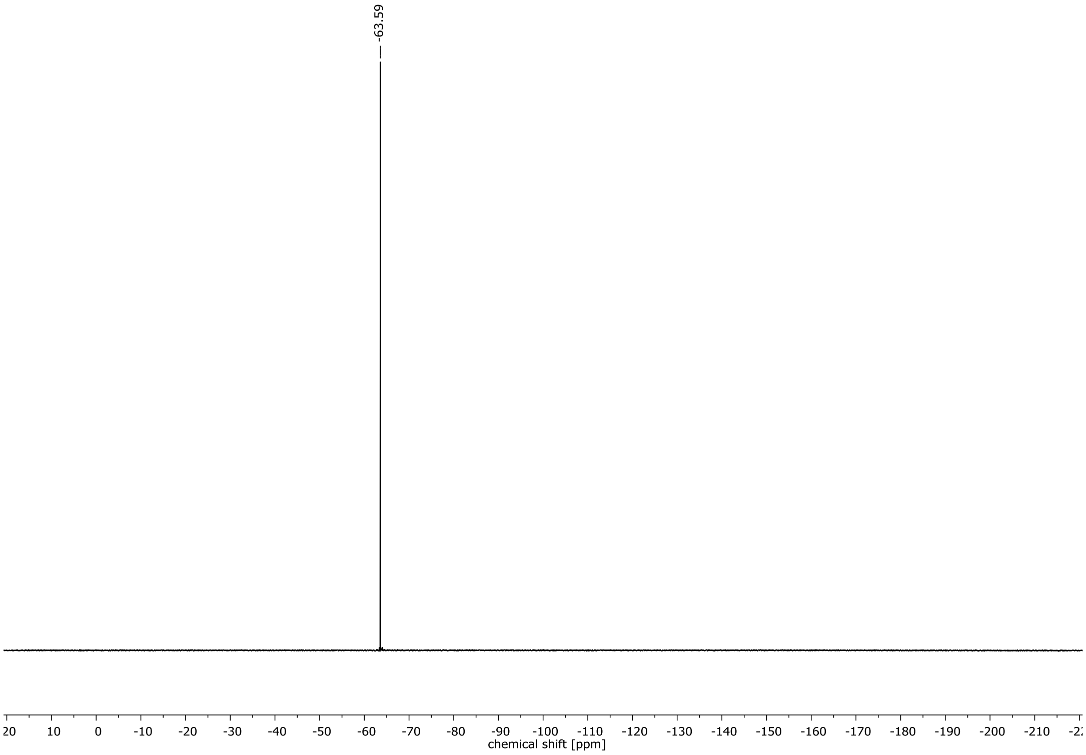

Figure S184. ^{19}F NMR spectrum of **10i** (376 MHz, DCM-d_2).

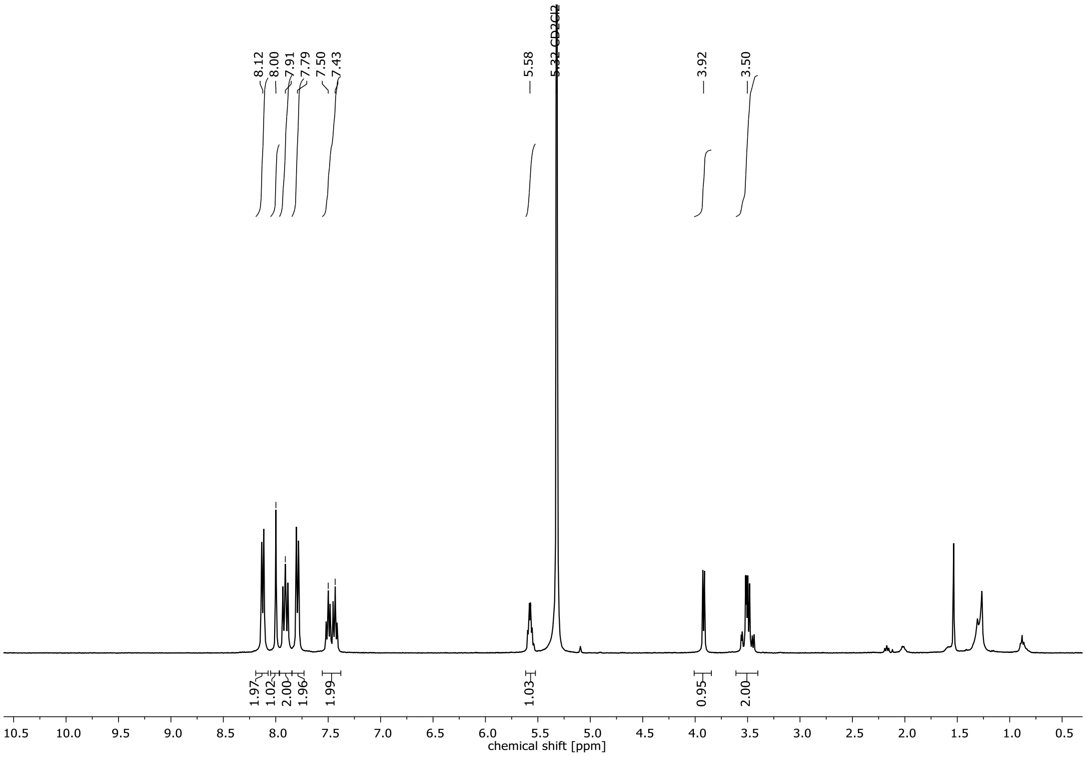

Figure S185. ^{1}H NMR spectrum of **10j** (400 MHz, DCM-d_2).

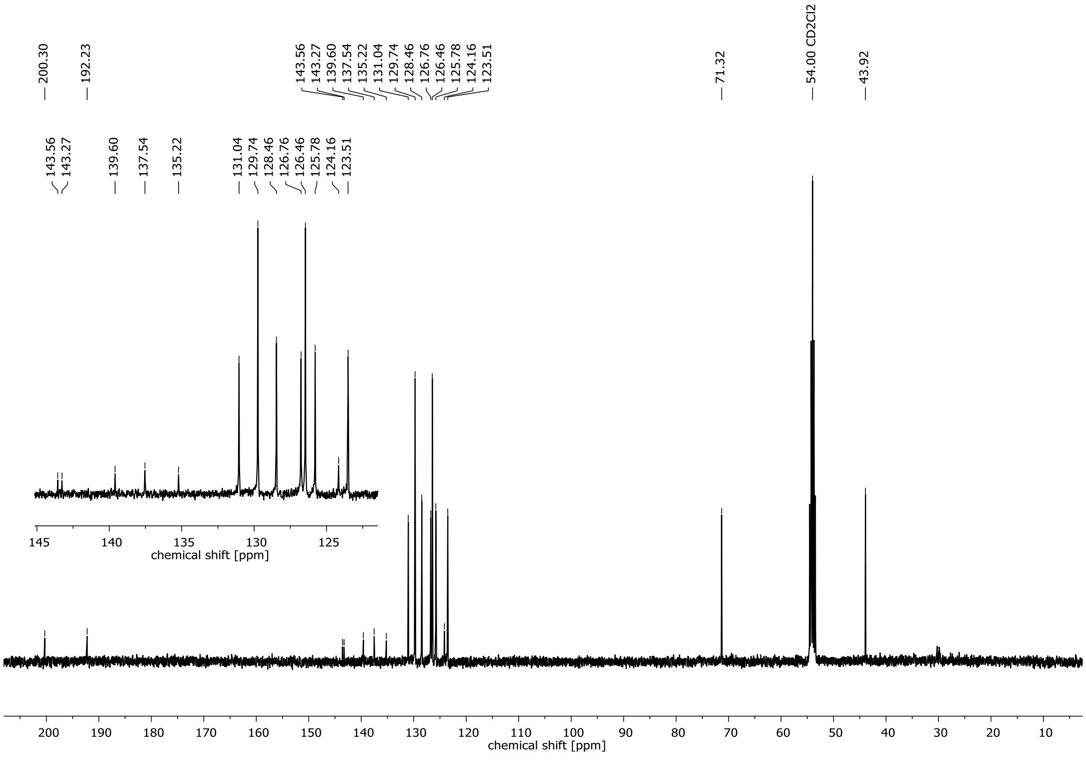

Figure S186. ^{13}C {^{1}H, ^{19}F} NMR spectrum of **10j** (101 MHz, DCM-d_2).

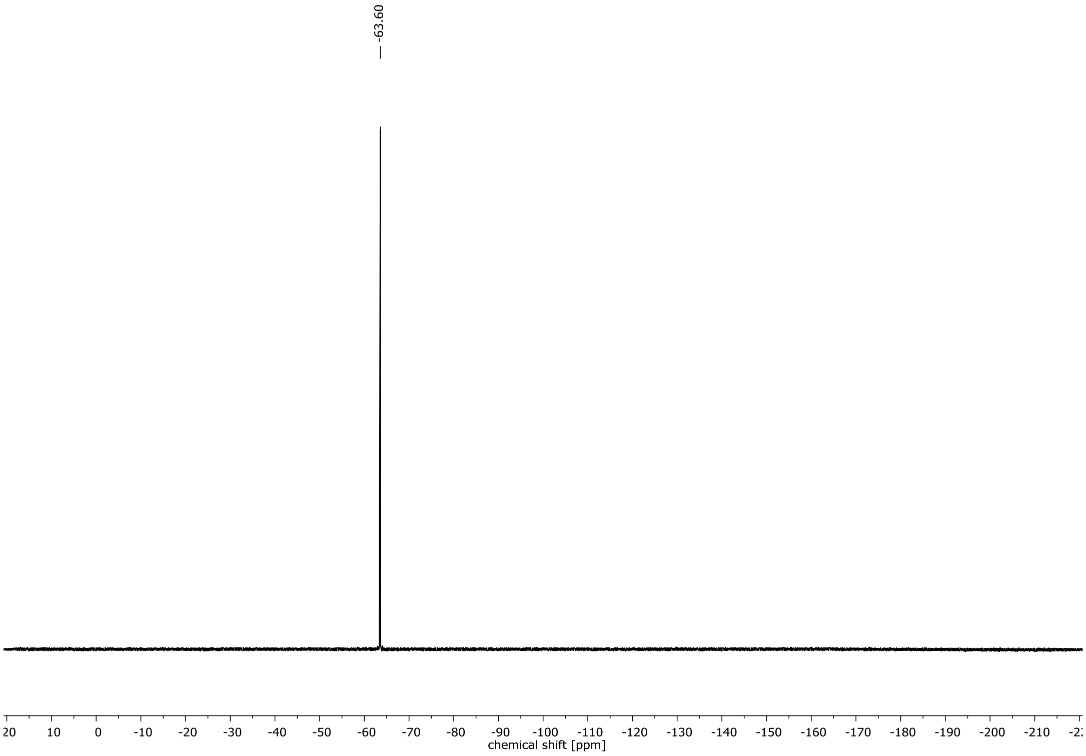

Figure S187. ^{19}F NMR spectrum of **10j** (376 MHz, DCM-d_2).

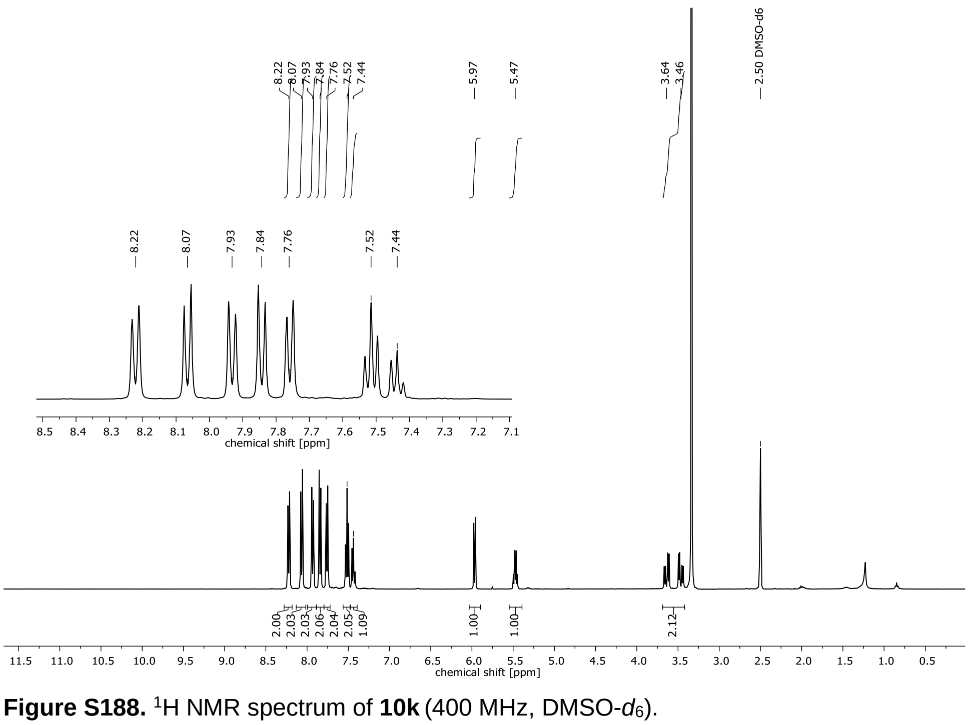

Figure S188. ^{1}H NMR spectrum of **10k** (400 MHz, DMSO-d_6).

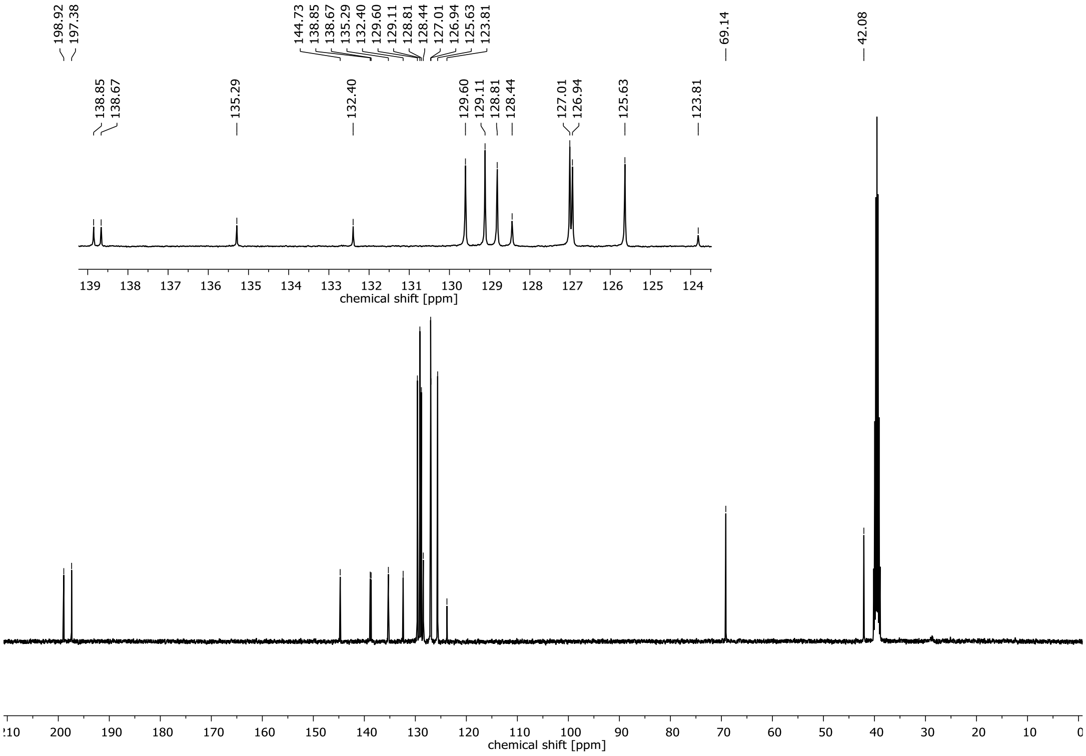

Figure S189. ^{13}C {^{1}H, ^{19}F} NMR spectrum of **10k** (101 MHz, DMSO-d_6).

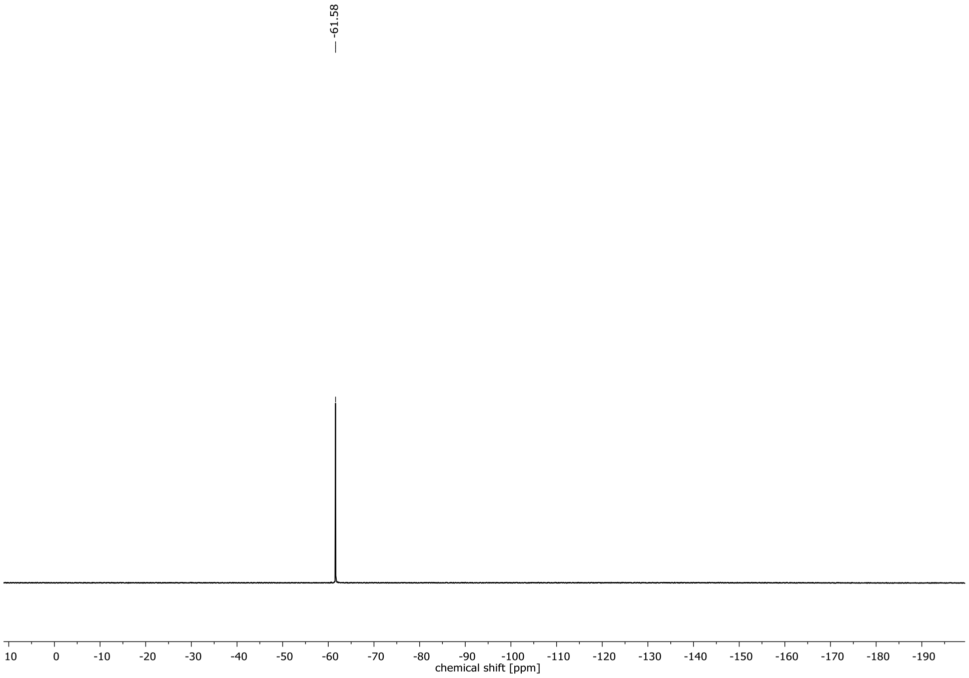

Figure S190. ^{19}F NMR spectrum of **10k** (376 MHz, DMSO-d_6).

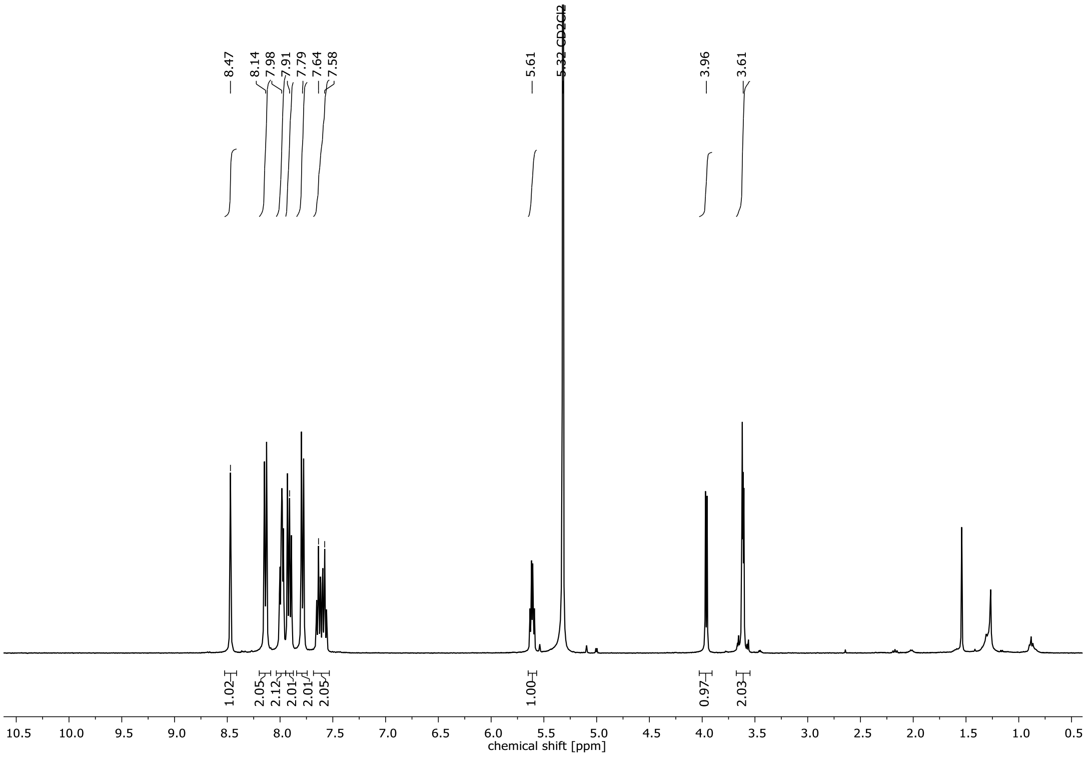

Figure S191. ^{1}H NMR spectrum of **10l** (400 MHz, DCM-d_2).

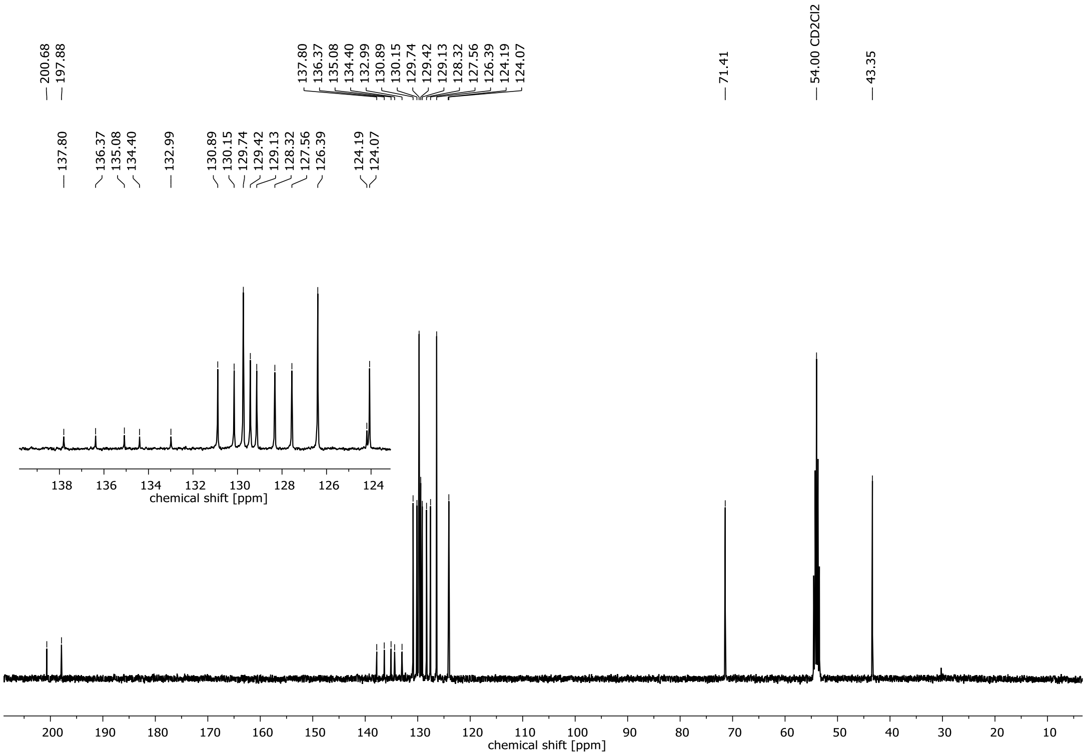

Figure S192. ^{13}C {^{1}H, ^{19}F} NMR spectrum of **10l** (101 MHz, DCM-d_2).

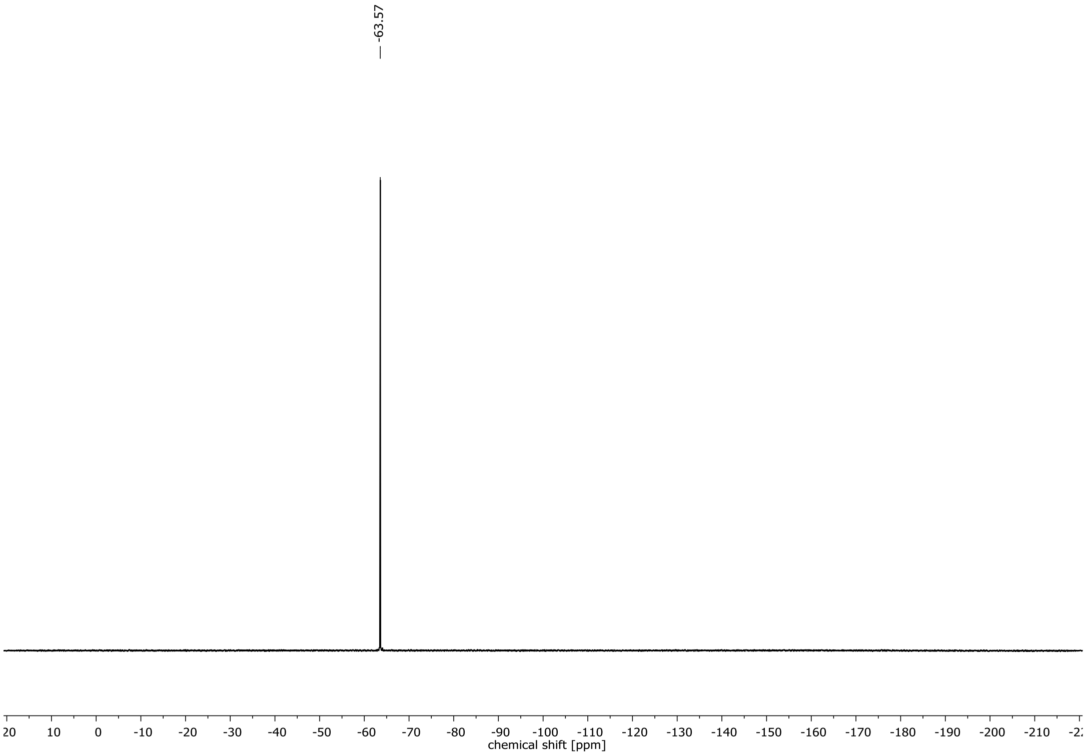

Figure S193. ^{19}F NMR spectrum of **10l** (376 MHz, DCM-d_2).

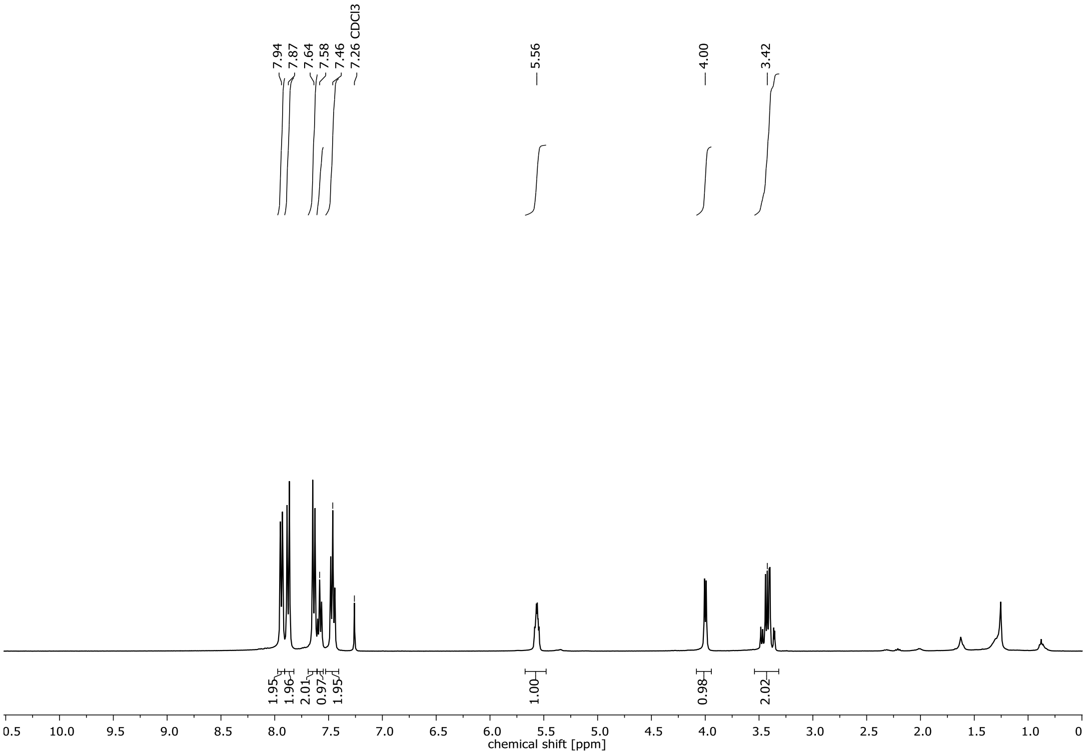

Figure S194. ^{1}H NMR spectrum of **10s** (400 MHz, chloroform-*d*).

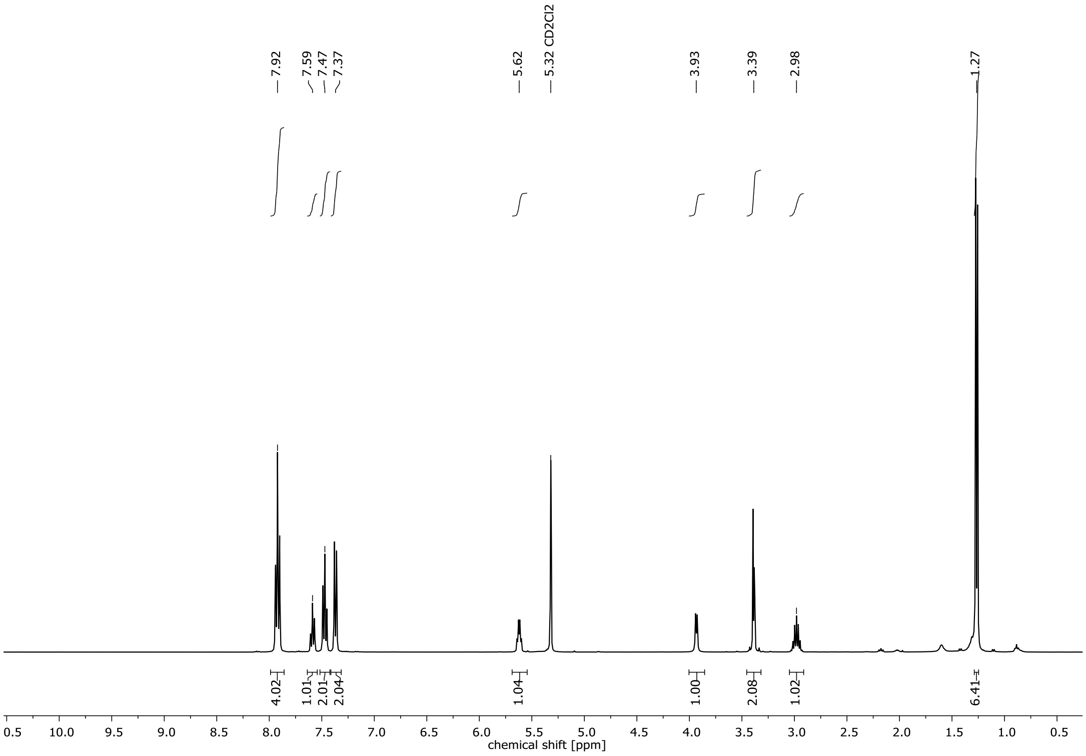

Figure S195. ^{1}H NMR spectrum of **10t** (400 MHz, DCM-d_2).

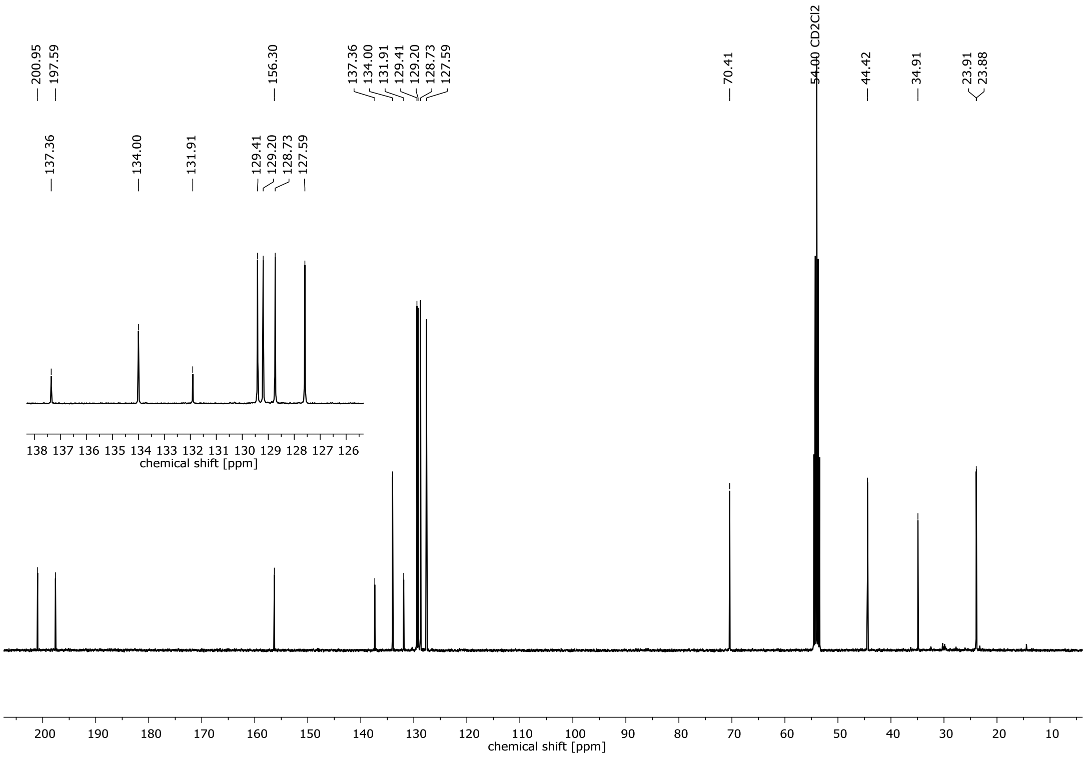

Figure S196. ^{13}C NMR spectrum of **10t** (101 MHz, DCM-d_2).

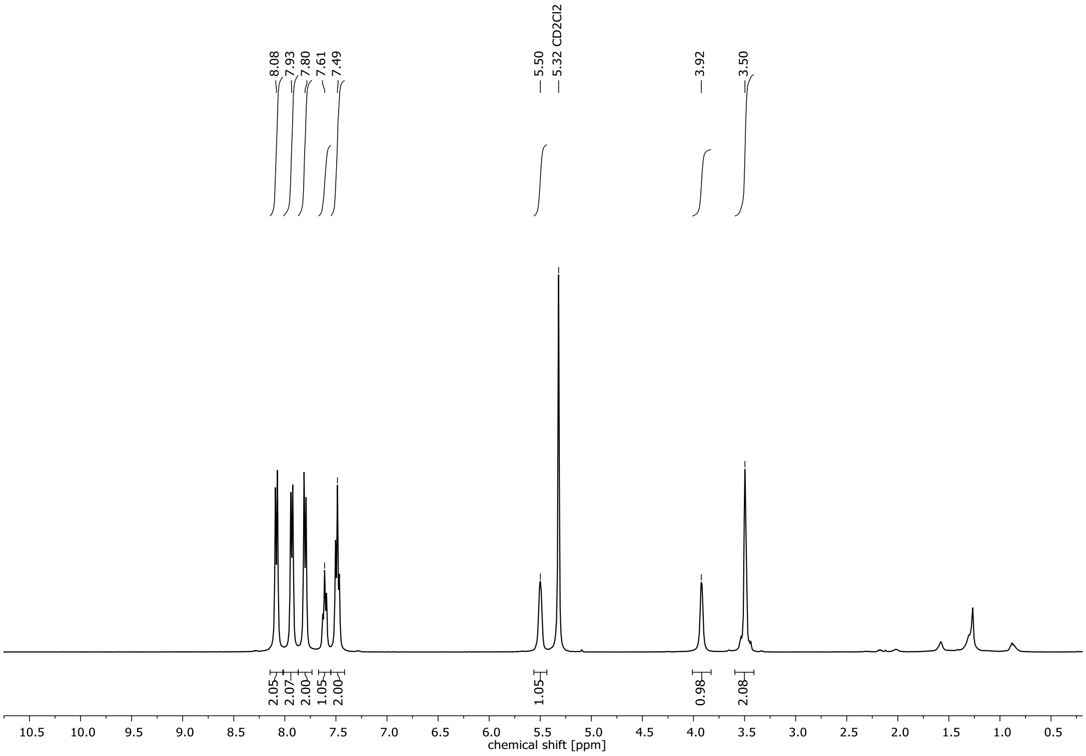

Figure S197. ^{1}H NMR spectrum of **10u** (400 MHz, DCM-d_2).

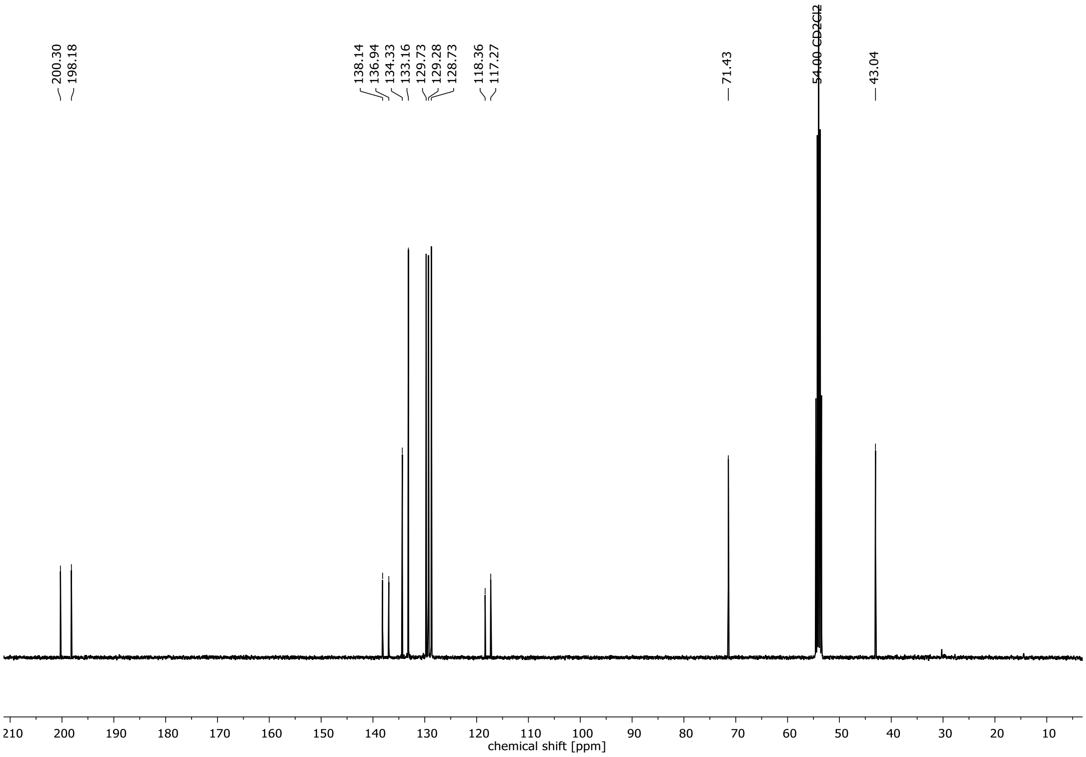

Figure S198. ^{13}C NMR spectrum of **10u** (101 MHz, DCM-d_2).

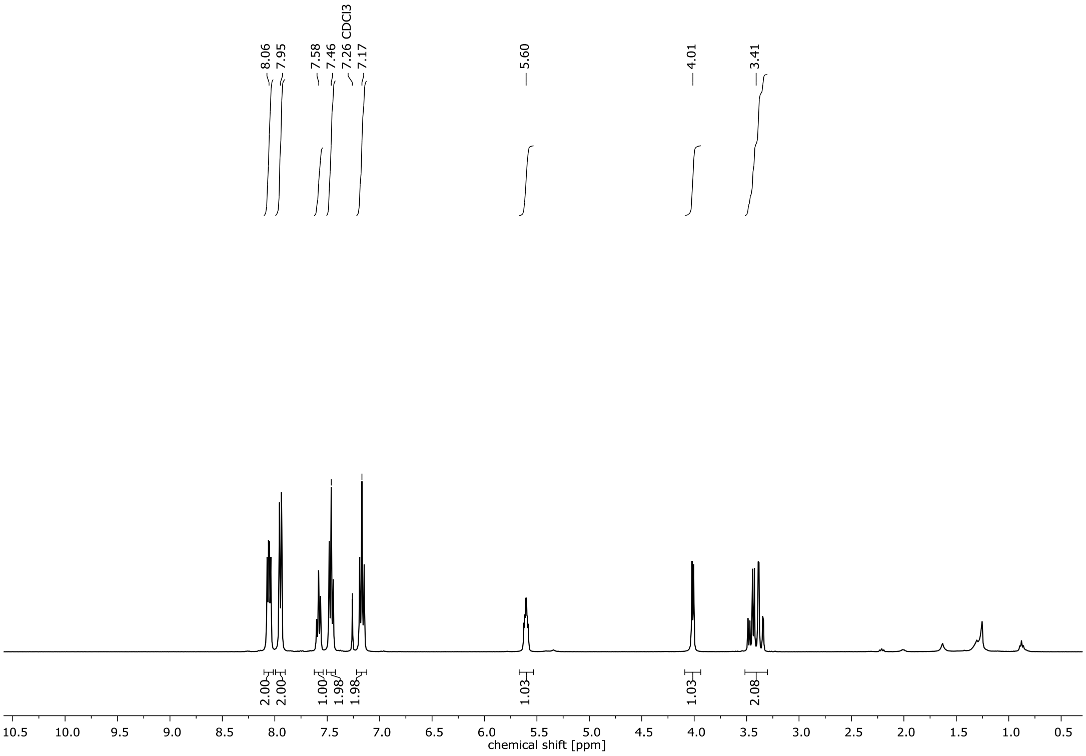

Figure S199. ^{1}H NMR spectrum of **10v** (400 MHz, chloroform-*d*).

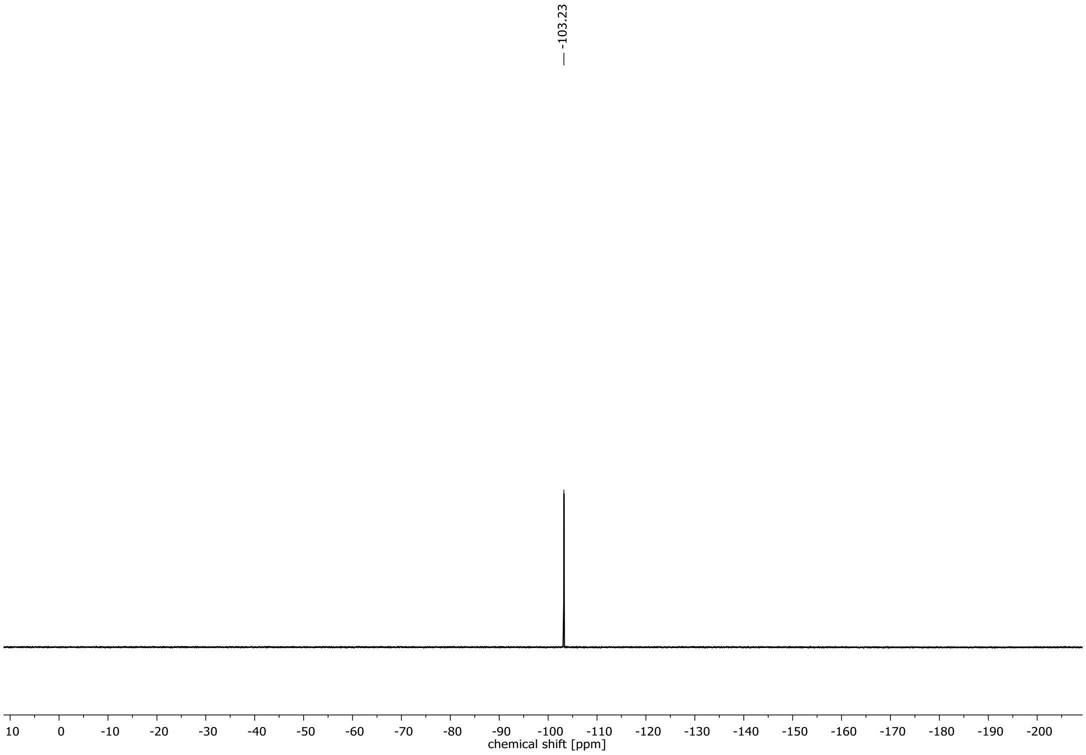

Figure S200. ^{19}F NMR spectrum of **10v** (376 MHz, chloroform-*d*).

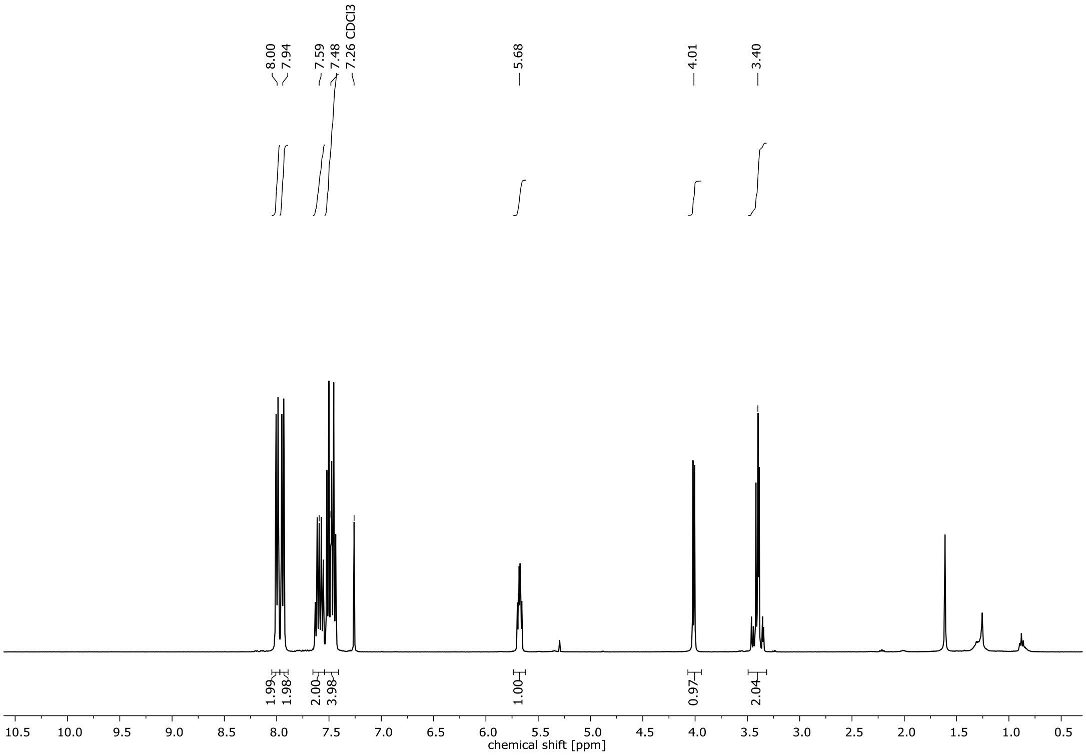

Figure S201. ^{1}H NMR spectrum of **10w** (400 MHz, chloroform-*d*).

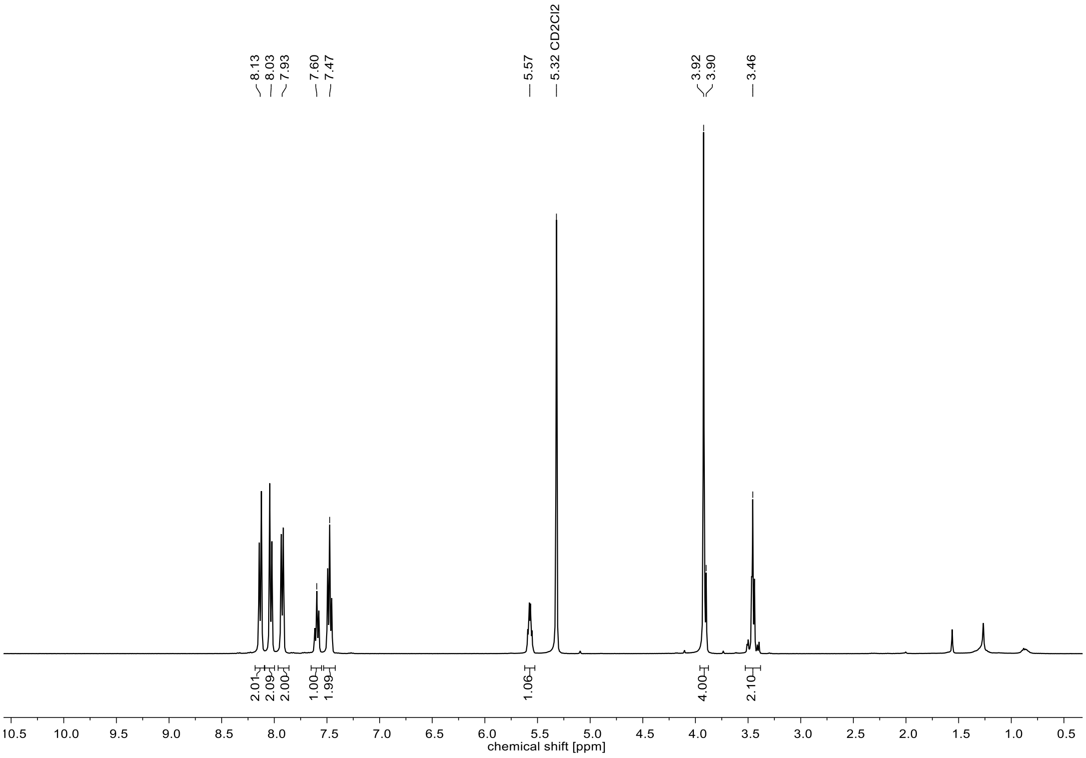

Figure S202. ^{1}H NMR spectrum of **10x** (400 MHz, chloroform-*d*).

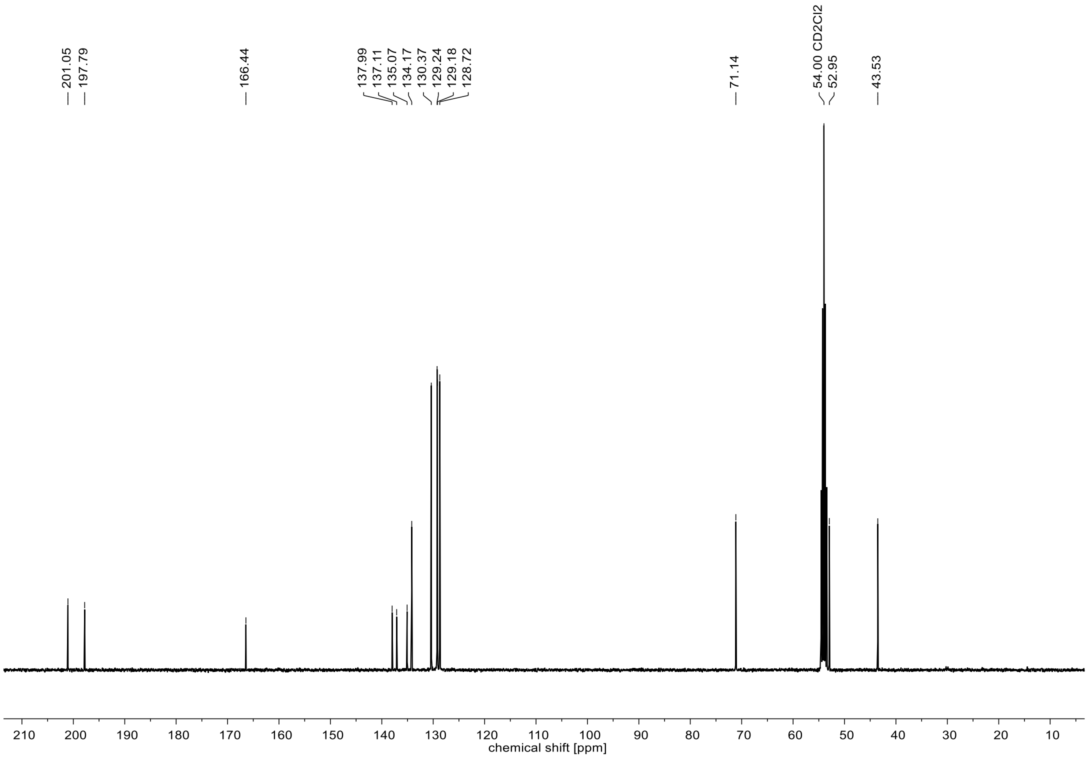

Figure S203. ^{13}C NMR spectrum of **10x** (101 MHz, chloroform-*d*).

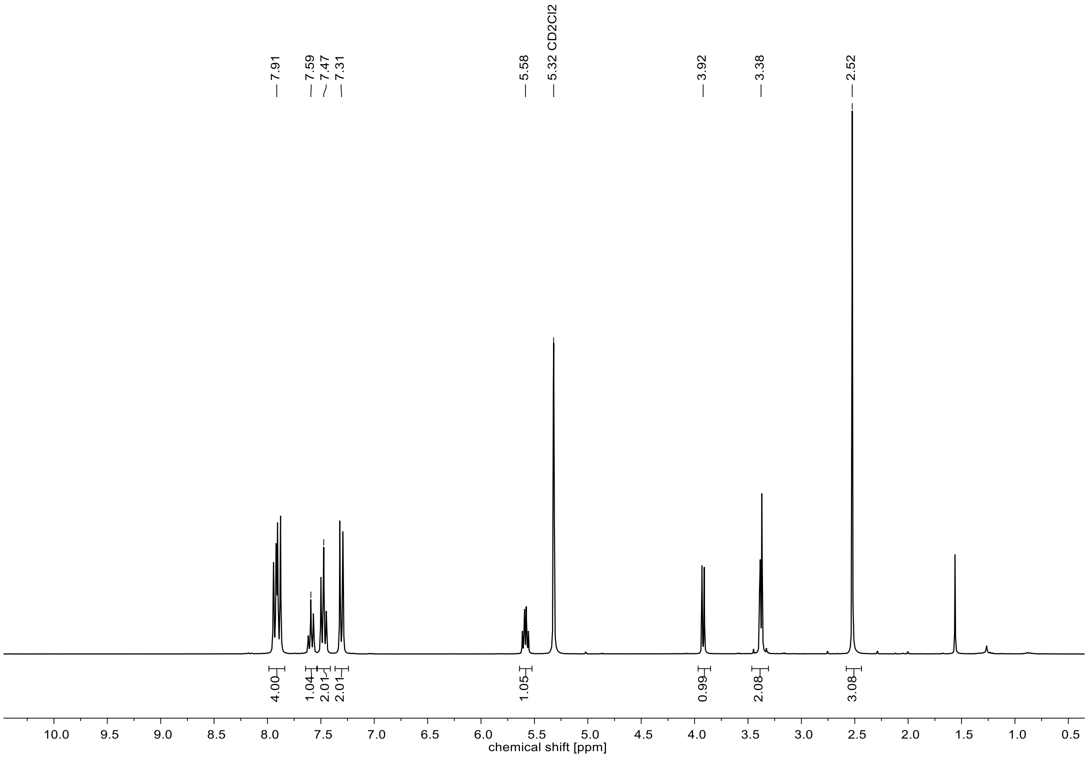

Figure S204. ^{1}H NMR spectrum of **10y** (300 MHz, chloroform-*d*).

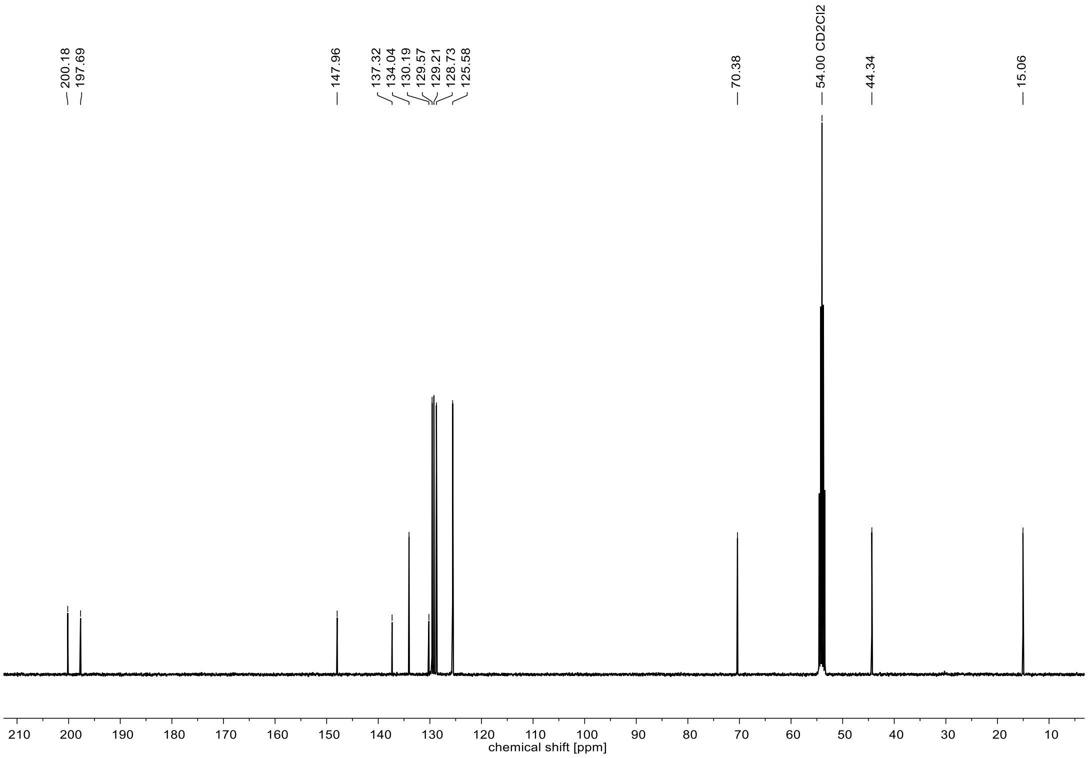

Figure S205. ^{13}C NMR spectrum of **10y** (101 MHz, chloroform-*d*).

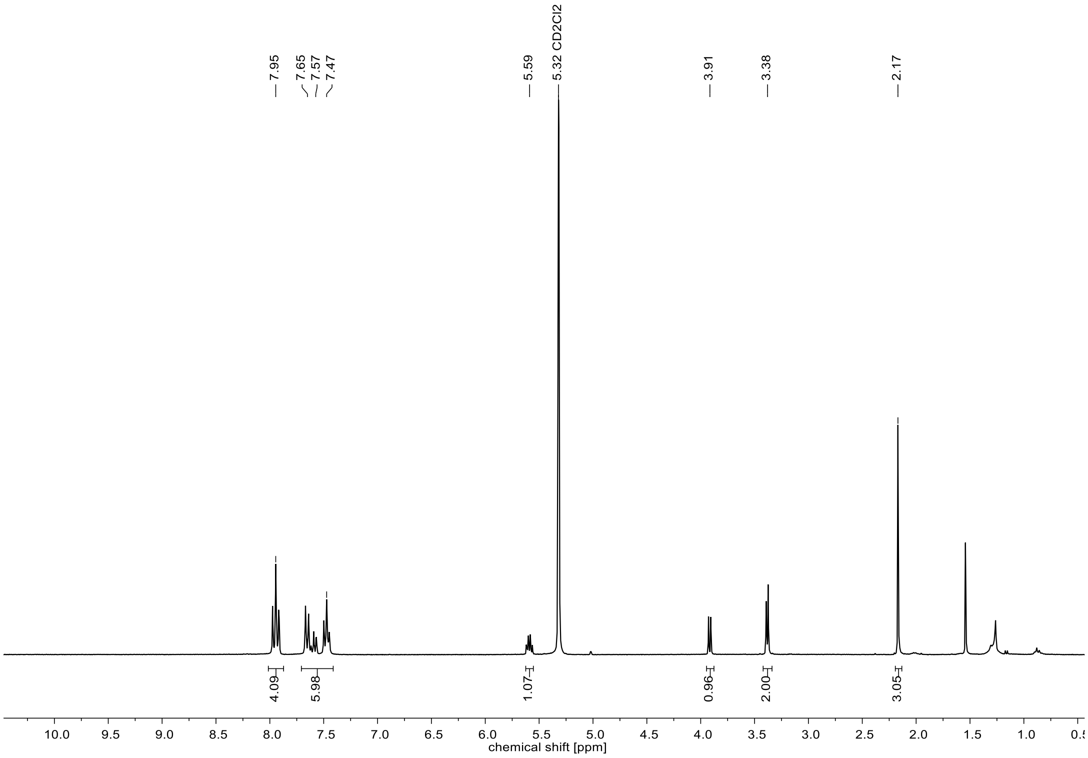

Figure S206. ^{1}H NMR spectrum of **10z** (300 MHz, chloroform-*d*).

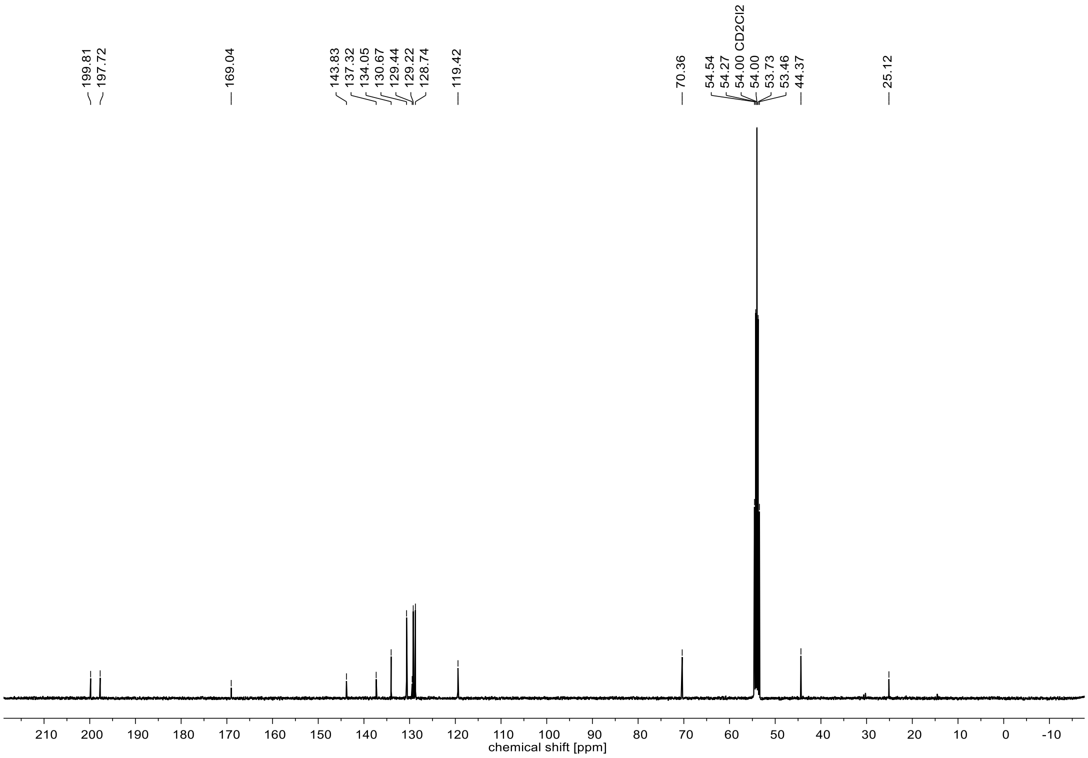

Figure S207. ^{13}C NMR spectrum of **10z** (101 MHz, DCM-*d*$_2$).

6. References

[74] C. M. Reid, C. Ebikeme, M. P. Barrett, E.-M. Patzewitz, S. Müller, D. J. Robins, A. Sutherland, *Bioorg. Med. Chem. Lett.* **2008**, *18*, 2455.

[75] C. M. Reid, C. Ebikeme, M. P. Barrett, E.-M. Patzewitz, S. Müller, D. J. Robins, A. Sutherland, *Bioorg. Med. Chem. Lett.* **2008**, *18*, 5399.

[76] P. Mahajabeen, A. Chadha, *Tetrahedron: Asymmetry* **2011**, *22*, 2156.

[77] N. Zhang, Y. Li, Z. Chen, W. Qin, *Synthesis* **2018**, *50*, 4063.

[78] I. Khan, B. G. Reed-Berendt, R. L. Melen, L. C. Morrill, *Angew. Chem. Int. Ed.* **2018**, *57*, 12356.

[79] H. Cao, S. Ma, Y. Feng, Y. Guo, P. Jiao, *Chem. Commun.* **2022**, *58*, 1780.

[80] A. Dewanji, L. van Dalsen, J. A. Rossi-Ashton, E. Gasson, G. E. M. Crisenza, D. J. Procter, *Nat. Chem.* **2023**, *15*, 43.

[81] see reference [49] and, for related systems: M. H. H. Voelkel, P. Wonner, S. M. Huber, *ChemistryOpen* **2020**, *9*, 214.

[82] *CrysAlisPro*; Rigaku Oxford Diffraction (1995-2018), 2018.

[83] L. J. Farrugia, *J. Appl. Crystallogr.* **1999**, *32*, 837.

[84] G. M Sheldrick, *Acta Cryst. A* **2008**, *64*, 112.

[85] C. B. Hübschle, G. M. Sheldrick, B. *J.* Dittrich, *Appl. Crystallogr.* **2011**, 44, 1281.

[86] Diamond - Crystal and Molecular Structure Visualization, Crystal Impact - H. Putz & K. Brandenburg GbR, Kreuzherrenstr. 102, 53227 Bonn, Germany, https://www.crystalimpact.de/diamond.

[87] S. Grimme, C. Bannwarth, P. Shushkov, *J.Chem.Theory Comput.* **2017**, *13*, 1989.

[88] C. Bannwarth, S. Ehlert, S. Grimme, *J. Chem. Theory Comput.* **2019**, *15*, 1652.

[89] S. Spicher, S. Grimme, *Angew. Chem. Int. Ed.* **2020**, *59*, 15665.

[90] C. Bannwarth, E. Caldeweyher, S. Ehlert, A. Hansen, P. Pracht, J. Seibert, S. Spicher, S. Grimme, *Wiley Interdiscip. Rev. Comput. Mol. Sci.* **2021**, *11*, e1493.

[91] F. Neese, *Wiley Interdiscip. Rev. Comput. Mol. Sci.* **2022**, *12*, e1606.

[92] Y. Zhao, D. G. Truhlar, *Theor. Chem. Acc.* **2008**, *120*, 215.

[93] F. Weigend, R. Ahlrichs, *Phys. Chem. Chem. Phys.* **2005**, *7*, 3297.

[94] F. Weigend, *Phys. Chem. Chem. Phys.* **2006**, *8*, 1057.

[95] A. V. Marenich, C. J. Cramer, D. G. Truhlar, *J. Phys. Chem. B* **2009**, *113*, 6378.

[96] S. Grimme, *Chem. Eur. J.* **2012**, *18*, 9955.

[97] N. Mardirossian, M. Head-Gordon, *Phys. Chem. Chem. Phys.* **2014**, *16*, 9904.

[98] G. Santra, N. Sylvetsky, J. M. L. Martin, *J. Phys. Chem. A* **2019**, *123*, 5129.
